# Agent-specific, histopathology-stratified hematologic malignancy risk among dpp-4 inhibitors, glp-1 receptor agonists, and SGLT2 inhibitors: a network meta-analysis of 270,471 participants

**DOI:** 10.1186/s13045-026-01788-5

**Published:** 2026-04-09

**Authors:** Pao-Yen Lin, Bing-Syuan Zeng, Jiann-Jy Chen, Bing-Yan Zeng, Mein-Woei Suen, Chao-Ming Hung, Chih-Wei Hsu, Brendon Stubbs, Yen-Wen Chen, Tien-Yu Chen, Wei-Te Lei, Shih-Pin Hsu, Yow-Ling Shiue, Kuan-Pin Su, Cheng-Ta Li, Hung-Yu Wang, Ping-Tao Tseng, Chih-Sung Liang

**Affiliations:** 1 Department of Psychiatry, Kaohsiung Chang Gung Memorial Hospital and Chang Gung University College of Medicine, Kaohsiung, Taiwan; 2 Department of Internal Medicine, E-Da Cancer Hospital, I-Shou University, Kaohsiung, Taiwan; 3 Prospect Clinic for Otorhinolaryngology & Neurology, Kaohsiung, Taiwan; 4 Department of Otorhinolaryngology, E-Da Cancer Hospital, I-Shou University, Kaohsiung, Taiwan; 5 Institute of Biomedical Sciences, National Sun Yat-sen University, 70 Lienhai Rd. 80424, Kaohsiung, Taiwan; 6 Department of Internal Medicine, E-Da Dachang Hospital, I-Shou University, Kaohsiung, Taiwan; 7 Department of Psychology, College of Medical and Health Science, Asia University, Taichung, Taiwan; 8 Gender Equality Education and Research Center, Asia University, Taichung, Taiwan; 9 Department of Medical Research, Asia University Hospital, Asia University, Taichung, Taiwan; 10 Department of Medical Research, China Medical University Hospital, China Medical University, Taichung, Taiwan; 11 Division of General Surgery, Department of Surgery, E-Da Cancer Hospital, I-Shou University, Kaohsiung, Taiwan; 12 School of Medicine, College of Medicine, I-Shou University, Kaohsiung, Taiwan; 13 Psychological Medicine, Institute of Psychiatry, Psychology and Neuroscience (IoPPN), King’s College London, London, UK; 14 Comprehensive Centre for Clinical Neurosciences and Mental Health, Medical University of Vienna, Vienna, Austria; 15 Clinical Division of Social Psychiatry, Department of Psychiatry and Psychotherapy, Medical University of Vienna, Vienna, Austria; 16 Department of Psychiatry, Tri-Service General Hospital, Taipei, Taiwan; 17 Department of Psychiatry, College of Medicine, National Defense Medical University, Taipei, Taiwan; 18 Division of Pediatric Allergy, Immunology, and Rheumatology, Department of Pediatrics, Hsinchu Municipal MacKay Children’s Hospital, Hsinchu City, Taiwan; 19 Graduate Institute of Clinical Medical Sciences, College of Medicine, Chang Gung University, Taoyuan City, Taiwan; 20 Department of Neurology, E-Da hospital, I-Shou University, Kaohsiung, Taiwan; 21 Institute of Precision Medicine, National Sun Yat-sen University, 70 Lienhai Rd. 80424, Kaohsiung, Taiwan; 22 Office of Research and Development, Asia University, No. 2 Yuh-Der RoadNorth District, Taichung, Taiwan; 23 College of Medicine, China Medical University, Taichung, Taiwan; 24 An-Nan Hospital, China Medical University, Tainan, Taiwan; 25 Mind-Body Interface Research Center (MBI Lab & Care), China Medical University Hospital, Taichung, Taiwan; 26 Department of Psychiatry, Taipei Veterans General Hospital, No. 201, Sec. 2, Shipai Road, Beitou, Taipei, 11267 Taiwan; 27 Division of Psychiatry, School of Medicine, National Yang Ming Chiao Tung University, Taipei, 112 Taiwan; 28 Institute of Brain Science and Brain Research Center, School of Medicine, National Yang Ming Chiao Tung University, Taipei, 112 Taiwan; 29 Kaohsiung Municipal Kai-Syuan Psychiatric Hospital, No.130, Kaisyuan 2nd Road, Lingya, Kaohsiung, 802211 Taiwan; 30 School of Medicine, College of Medicine, National Sun Yat-sen University, No. 70, Lienhai Road, 80424 Kaohsiung, Taiwan; 31 Department of Psychiatry, Beitou Branch, Tri-Service General Hospital, National Defense Medical University, No. 60, Xinmin Road, Beitou, Taipei, 112 Taiwan; 32 Department of Psychiatry, School of Medicine, College of Medicine, National Defense Medical University, Taipei, Taiwan; 33 Lab Head, Functional Neuroimaging and Brain Stimulation Lab, Taipei Veterans General Hospital, Taipei, Taiwan

**Keywords:** Network meta-analysis, DPP-4 inhibitor, GLP-1 receptor agonist, SGLT2 inhibitor, Hematology, Malignancy, Lymphoma, Leukemia, Myeloma

## Abstract

**Abstract:**

Dipeptidyl peptidase-4 (DPP-4) inhibitors, glucagon-like peptide-1 (GLP-1) receptor agonists, and sodium–glucose cotransporter 2 (SGLT2) inhibitors are widely prescribed for their cardiometabolic benefits, yet their hematologic oncologic safety remains uncertain. Because hematologic malignancies are highly lethal and biologically heterogeneous, delineating drug-specific risks across histopathologic subtypes is clinically crucial. This histopathology-stratified network meta-analysis (NMA) evaluated and compared the hematologic malignancy risks associated with individual agents from these drug classes. Following Cochrane guidance for adverse-event synthesis, we conducted this frequentist-based NMA of randomized controlled trials (RCTs). The primary endpoint was incident hematologic malignancy, categorized a priori into leukemia (acute and chronic forms), lymphoma (Hodgkin and non-Hodgkin), and myeloma/plasma cell neoplasms. Bayesian models were used as sensitivity analyses. Seventy-five RCTs including 270,471 participants were eligible. Dulaglutide was associated with a significantly increased risk of overall hematologic malignancy (RR = 2.17, 95% CIs = 1.14–4.17). In contrast, tirzepatide (RR = 0.22, 95% CIs = 0.06–0.78) and linagliptin (RR = 0.51, 95% CIs = 0.27–0.95) were linked to a reduced overall risk. In histopathology-specific analyses, tirzepatide showed a significant protective association against non-Hodgkin’s lymphoma, whereas no agent demonstrated clear signals for leukemia or myeloma. In this histopathology-focused NMA, dulaglutide emerged as the only agent with a significantly elevated overall hematologic malignancy risk, whereas tirzepatide and linagliptin exhibited protective profiles. The lymphoma-specific benefit observed for tirzepatide underscores the value of histologic subclassification when evaluating oncologic safety of antidiabetic therapies and calls for targeted mechanistic and long-term outcome studies.

**Trial registration:**

PROSPERO CRD420251151419. The study protocol was approved by the Institutional Review Board of the Tri-Service General Hospital, National Defense Medical University (TSGHIRB E202516007).

**Supplementary Information:**

The online version contains supplementary material available at 10.1186/s13045-026-01788-5.

## To the editor:

Compared with solid tumors, hematologic malignancies have rarely been the primary focus of safety assessments for novel antidiabetic agents, such as dipeptidyl peptidase-4 (DPP-4) inhibitors, glucagon-like peptide-1 (GLP-1) receptor agonists, and sodium–glucose cotransporter 2 (SGLT2) inhibitors. This gap is clinically meaningful because many hematologic malignancies display aggressive clinical courses and poor survival [1]. In light of these considerations, we undertook a comprehensive NMA to evaluate hematologic malignancy risk associated with novel antidiabetic agents, with explicit emphasis on histopathology-based classification. This work extends our earlier preliminary NMA, which focused on GLP-1 receptor agonists and SGLT2 inhibitors and primarily assessed overall hematologic malignancy [2] by incorporating DPP-4 inhibitors and dual agonists and by aligning hematologic cancer outcomes with standardized histologic categories. Through parallel evaluation of aggregate hematologic malignancy and its major subtypes (leukemia, lymphoma, and myeloma), we aimed to determine whether distinct agents exert differential, histology-specific oncologic effects that would be obscured in overall composite endpoints.

The detailed methodology had been listed in Appendix. In brief, we performed a frequentist random-effects NMA using the network suite in STATA version 16.0 (StataCorp, College Station, TX) [3]. Effect estimates were summarized as risk ratios (RRs) with 95% confidence intervals (CIs). Between-study heterogeneity was assessed via τ^2^ estimates, and network inconsistency was evaluated using loop-specific, node-splitting, and design-by-treatment interaction approaches [4]. To test robustness, we performed Bayesian sensitivity analyses using the netmeta module in MetaInsight v4.0.2, which accommodates zero-event trials without the need for continuity corrections.

**Fig. 1:**
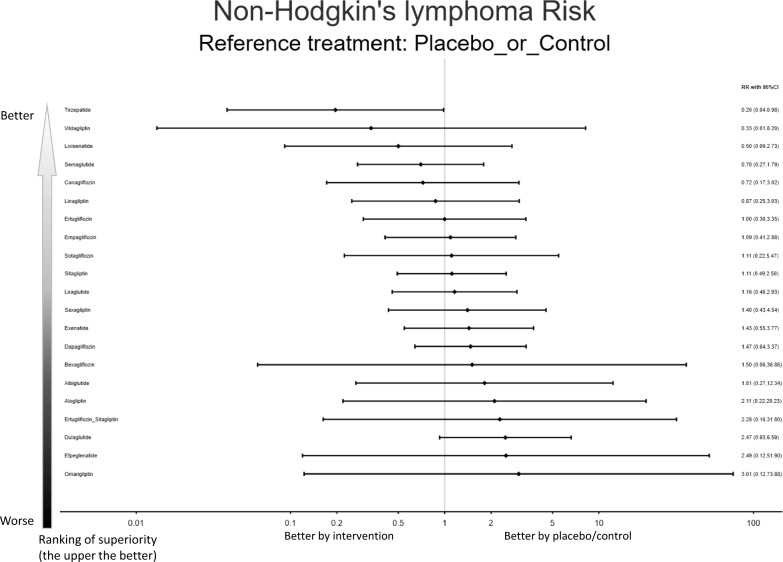
Forest plot of primary outcome: non-Hodgkin’s lymphoma.When the effect size (expressed as risk ratio) is less than 1, the specified treatment is associated with tumor events compared to placebo/controls

The full description of result was listed in Appendix. In brief, tirzepatide was the only agent associated with a statistically significant reduction in overall lymphoma incidence (RR = 0.20; 95% CIs = 0.04–0.99). When further stratified, tirzepatide also showed a marked protective association with non-Hodgkin’s lymphoma (RR = 0.20; 95% CIs = 0.04–0.98) (eFigure 1M–1P, eFigure 2O–2Q, and eTable 6G–6I) (Fig. 1 and Table 1).

**Table 1:** League table of the primary outcome: non-Hodgkin’s lymphoma

*Tirzepatide*	2.56 (0.25,26.64)	1.71 (0.05,61.61)	3.57 (0.62,20.49)	3.69 (0.42,31.99)	4.46 (0.58,34.33)	*5.11 (1.02,25.74)	5.11 (0.68,38.48)	5.68 (0.94,34.38)	5.66 (0.58,54.96)	5.57 (0.84,36.75)	5.92 (0.92,38.18)	7.69 (0.21,277.05)	7.17 (0.97,52.89)	*7.33 (1.12,48.19)	*7.50 (1.22,46.18)	9.27 (0.75,113.83)	11.65 (0.53,255.95)	12.75 (0.41,397.34)	10.77 (0.67,173.65)	15.40 (0.43,555.25)	*12.63 (1.91,83.69)
0.39 (0.04,4.07)	*Lixisenatide*	0.67 (0.02,24.99)	1.40 (0.20,9.71)	1.44 (0.16,13.29)	1.74 (0.21,14.31)	2.00 (0.37,10.91)	2.00 (0.25,16.06)	2.22 (0.34,14.56)	2.21 (0.21,22.77)	2.18 (0.31,15.40)	2.31 (0.33,16.01)	3.01 (0.08,112.39)	2.80 (0.36,22.08)	2.87 (0.41,20.19)	2.93 (0.44,19.40)	3.62 (0.28,46.90)	4.55 (0.20,104.70)	4.99 (0.15,161.44)	4.21 (0.25,71.19)	6.02 (0.16,225.25)	4.94 (0.70,35.06)
0.58 (0.02,21.08)	1.50 (0.04,55.94)	*Vildagliptin*	2.09 (0.07,58.59)	2.16 (0.06,71.84)	2.61 (0.08,80.80)	2.99 (0.12,73.33)	2.99 (0.10,91.45)	3.32 (0.12,90.10)	3.31 (0.09,118.32)	3.26 (0.11,92.31)	3.46 (0.12,96.85)	4.50 (0.05,414.92)	4.19 (0.14,126.68)	4.29 (0.15,121.26)	4.39 (0.16,119.63)	5.42 (0.13,225.96)	6.81 (0.11,430.27)	7.46 (0.09,613.82)	6.30 (0.13,316.94)	9.01 (0.10,831.42)	7.39 (0.26,209.87)
0.28 (0.05,1.61)	0.72 (0.10,4.99)	0.48 (0.02,13.45)	*Semaglutide*	1.03 (0.19,5.74)	1.25 (0.26,5.95)	1.43 (0.56,3.67)	1.43 (0.31,6.61)	1.59 (0.48,5.26)	1.59 (0.25,10.13)	1.56 (0.40,6.04)	1.66 (0.44,6.22)	2.15 (0.08,60.46)	2.01 (0.45,9.05)	2.05 (0.53,7.91)	2.10 (0.60,7.37)	2.60 (0.31,21.98)	3.26 (0.20,53.02)	3.57 (0.15,85.73)	3.02 (0.26,34.96)	4.31 (0.15,121.18)	3.54 (0.91,13.77)
0.27 (0.03,2.35)	0.69 (0.08,6.40)	0.46 (0.01,15.45)	0.97 (0.17,5.38)	*Canagliflozin*	1.21 (0.18,8.09)	1.39 (0.33,5.82)	1.39 (0.21,9.06)	1.54 (0.30,8.00)	1.53 (0.18,13.15)	1.51 (0.27,8.55)	1.60 (0.29,8.87)	2.09 (0.06,69.49)	1.94 (0.30,12.42)	1.99 (0.35,11.21)	2.04 (0.39,10.68)	2.51 (0.23,27.57)	3.16 (0.16,63.54)	3.46 (0.12,99.32)	2.92 (0.20,42.55)	4.18 (0.13,139.27)	3.43 (0.60,19.48)
0.22 (0.03,1.73)	0.57 (0.07,4.72)	0.38 (0.01,11.90)	0.80 (0.17,3.82)	0.83 (0.12,5.54)	*Linagliptin*	1.15 (0.33,4.00)	1.15 (0.20,6.53)	1.27 (0.29,5.65)	1.27 (0.17,9.65)	1.25 (0.26,6.09)	1.33 (0.28,6.30)	1.73 (0.06,53.50)	1.61 (0.29,8.93)	1.65 (0.34,7.97)	1.68 (0.38,7.54)	2.08 (0.21,20.50)	2.61 (0.14,48.32)	2.86 (0.11,76.23)	2.42 (0.18,32.02)	3.46 (0.11,107.23)	2.84 (0.58,13.87)
*0.20 (0.04,0.98)	0.50 (0.09,2.73)	0.33 (0.01,8.20)	0.70 (0.27,1.79)	0.72 (0.17,3.02)	0.87 (0.25,3.03)	*Placebo_or_* *Control*	1.00 (0.30,3.35)	1.11 (0.49,2.50)	1.11 (0.22,5.47)	1.09 (0.41,2.88)	1.16 (0.46,2.93)	1.50 (0.06,36.86)	1.40 (0.43,4.54)	1.43 (0.55,3.77)	1.47 (0.64,3.37)	1.81 (0.27,12.34)	2.28 (0.16,31.80)	2.49 (0.12,51.90)	2.11 (0.22,20.23)	3.01 (0.12,73.88)	2.47 (0.93,6.59)
0.20 (0.03,1.47)	0.50 (0.06,4.03)	0.33 (0.01,10.24)	0.70 (0.15,3.23)	0.72 (0.11,4.72)	0.87 (0.15,4.96)	1.00 (0.30,3.36)	*Ertugliflozin*	1.11 (0.27,4.57)	1.11 (0.15,8.23)	1.09 (0.23,5.16)	1.16 (0.25,5.33)	1.51 (0.05,46.04)	1.40 (0.26,7.59)	1.44 (0.30,6.75)	1.47 (0.34,6.38)	1.81 (0.19,17.53)	2.28 (0.17,31.46)	2.50 (0.10,65.56)	2.11 (0.16,27.43)	3.01 (0.10,92.29)	2.47 (0.52,11.75)
0.18 (0.03,1.07)	0.45 (0.07,2.96)	0.30 (0.01,8.17)	0.63 (0.19,2.08)	0.65 (0.12,3.37)	0.78 (0.18,3.48)	0.90 (0.40,2.03)	0.90 (0.22,3.70)	*Sitagliptin*	1.00 (0.17,5.99)	0.98 (0.28,3.49)	1.04 (0.30,3.58)	1.35 (0.05,36.75)	1.26 (0.30,5.27)	1.29 (0.37,4.56)	1.32 (0.41,4.22)	1.63 (0.20,13.10)	2.05 (0.15,28.31)	2.25 (0.10,52.02)	1.90 (0.17,20.99)	2.71 (0.10,73.66)	2.23 (0.62,7.95)
0.18 (0.02,1.72)	0.45 (0.04,4.65)	0.30 (0.01,10.81)	0.63 (0.10,4.03)	0.65 (0.08,5.58)	0.79 (0.10,5.99)	0.90 (0.18,4.47)	0.90 (0.12,6.71)	1.00 (0.17,6.03)	*Sotagliflozin*	0.98 (0.15,6.40)	1.05 (0.16,6.65)	1.36 (0.04,48.60)	1.27 (0.17,9.22)	1.30 (0.20,8.39)	1.33 (0.22,8.04)	1.64 (0.13,19.90)	2.06 (0.09,44.95)	2.25 (0.07,69.67)	1.90 (0.12,30.39)	2.72 (0.08,97.39)	2.23 (0.34,14.58)
0.18 (0.03,1.19)	0.46 (0.06,3.25)	0.31 (0.01,8.70)	0.64 (0.17,2.48)	0.66 (0.12,3.75)	0.80 (0.16,3.90)	0.92 (0.35,2.43)	0.92 (0.19,4.34)	1.02 (0.29,3.62)	1.02 (0.16,6.61)	*Empagliflozin*	1.06 (0.28,4.08)	1.38 (0.05,39.13)	1.29 (0.28,5.93)	1.32 (0.33,5.19)	1.35 (0.37,4.85)	1.66 (0.19,14.30)	2.09 (0.13,34.77)	2.29 (0.09,55.52)	1.93 (0.16,22.71)	2.77 (0.10,78.44)	2.27 (0.57,9.04)
0.17 (0.03,1.09)	0.43 (0.06,2.99)	0.29 (0.01,8.09)	0.60 (0.16,2.26)	0.62 (0.11,3.44)	0.75 (0.16,3.57)	0.86 (0.34,2.19)	0.86 (0.19,3.98)	0.96 (0.28,3.30)	0.96 (0.15,6.08)	0.94 (0.24,3.62)	*Liraglutide*	1.30 (0.05,36.37)	1.21 (0.27,5.42)	1.24 (0.32,4.73)	1.27 (0.36,4.41)	1.57 (0.19,13.20)	1.97 (0.12,32.23)	2.16 (0.09,51.56)	1.82 (0.16,21.01)	2.60 (0.09,72.89)	2.14 (0.55,8.25)
0.13 (0.00,4.68)	0.33 (0.01,12.43)	0.22 (0.00,20.50)	0.46 (0.02,13.02)	0.48 (0.01,15.97)	0.58 (0.02,17.96)	0.66 (0.03,16.30)	0.66 (0.02,20.32)	0.74 (0.03,20.02)	0.74 (0.02,26.29)	0.72 (0.03,20.51)	0.77 (0.03,21.52)	*Bexagliflozin*	0.93 (0.03,28.15)	0.95 (0.03,26.95)	0.98 (0.04,26.58)	1.20 (0.03,50.21)	1.51 (0.02,95.62)	1.66 (0.02,136.42)	1.40 (0.03,70.43)	2.00 (0.02,184.77)	1.64 (0.06,46.64)
0.14 (0.02,1.03)	0.36 (0.05,2.81)	0.24 (0.01,7.21)	0.50 (0.11,2.24)	0.51 (0.08,3.29)	0.62 (0.11,3.45)	0.71 (0.22,2.31)	0.71 (0.13,3.86)	0.79 (0.19,3.31)	0.79 (0.11,5.75)	0.78 (0.17,3.58)	0.83 (0.18,3.70)	1.07 (0.04,32.43)	*Saxagliptin*	1.02 (0.22,4.69)	1.05 (0.25,4.42)	1.29 (0.14,12.27)	1.63 (0.09,29.16)	1.78 (0.07,46.15)	1.50 (0.12,19.24)	2.15 (0.07,65.00)	1.76 (0.38,8.15)
*0.14 (0.02,0.90)	0.35 (0.05,2.46)	0.23 (0.01,6.59)	0.49 (0.13,1.87)	0.50 (0.09,2.83)	0.61 (0.13,2.94)	0.70 (0.27,1.83)	0.70 (0.15,3.28)	0.77 (0.22,2.73)	0.77 (0.12,4.99)	0.76 (0.19,2.99)	0.81 (0.21,3.08)	1.05 (0.04,29.64)	0.98 (0.21,4.47)	*Exenatide*	1.02 (0.29,3.66)	1.26 (0.15,10.82)	1.59 (0.10,26.32)	1.74 (0.07,42.04)	1.47 (0.13,17.18)	2.10 (0.07,59.41)	1.72 (0.44,6.82)
*0.13 (0.02,0.82)	0.34 (0.05,2.26)	0.23 (0.01,6.21)	0.48 (0.14,1.67)	0.49 (0.09,2.58)	0.59 (0.13,2.66)	0.68 (0.30,1.56)	0.68 (0.16,2.96)	0.76 (0.24,2.42)	0.75 (0.12,4.57)	0.74 (0.21,2.67)	0.79 (0.23,2.74)	1.03 (0.04,27.94)	0.96 (0.23,4.03)	0.98 (0.27,3.49)	*Dapagliflozin*	1.24 (0.15,9.99)	1.55 (0.10,24.64)	1.70 (0.07,39.56)	1.44 (0.13,15.99)	2.05 (0.08,56.00)	1.68 (0.47,6.09)
0.11 (0.01,1.33)	0.28 (0.02,3.57)	0.18 (0.00,7.69)	0.39 (0.05,3.26)	0.40 (0.04,4.36)	0.48 (0.05,4.74)	0.55 (0.08,3.76)	0.55 (0.06,5.33)	0.61 (0.08,4.92)	0.61 (0.05,7.42)	0.60 (0.07,5.17)	0.64 (0.08,5.38)	0.83 (0.02,34.60)	0.77 (0.08,7.34)	0.79 (0.09,6.78)	0.81 (0.10,6.55)	*Albiglutide*	1.26 (0.05,32.76)	1.38 (0.04,49.92)	1.16 (0.06,22.57)	1.66 (0.04,69.34)	1.36 (0.16,11.76)
0.09 (0.00,1.89)	0.22 (0.01,5.05)	0.15 (0.00,9.27)	0.31 (0.02,4.98)	0.32 (0.02,6.37)	0.38 (0.02,7.07)	0.44 (0.03,6.13)	0.44 (0.03,6.06)	0.49 (0.04,6.73)	0.49 (0.02,10.61)	0.48 (0.03,7.95)	0.51 (0.03,8.32)	0.66 (0.01,41.71)	0.62 (0.03,11.04)	0.63 (0.04,10.44)	0.64 (0.04,10.22)	0.80 (0.03,20.74)	*Ertugliflozin_* *Sitagliptin*	1.09 (0.02,61.04)	0.92 (0.03,29.84)	1.32 (0.02,83.58)	1.08 (0.07,18.08)
0.08 (0.00,2.44)	0.20 (0.01,6.50)	0.13 (0.00,11.03)	0.28 (0.01,6.72)	0.29 (0.01,8.30)	0.35 (0.01,9.31)	0.40 (0.02,8.35)	0.40 (0.02,10.53)	0.45 (0.02,10.31)	0.44 (0.01,13.71)	0.44 (0.02,10.59)	0.46 (0.02,11.10)	0.60 (0.01,49.63)	0.56 (0.02,14.57)	0.58 (0.02,13.90)	0.59 (0.03,13.69)	0.73 (0.02,26.35)	0.91 (0.02,50.91)	*Efpeglenatide*	0.84 (0.02,37.22)	1.21 (0.01,99.44)	0.99 (0.04,24.07)
0.09 (0.01,1.50)	0.24 (0.01,4.02)	0.16 (0.00,7.99)	0.33 (0.03,3.84)	0.34 (0.02,4.99)	0.41 (0.03,5.48)	0.47 (0.05,4.56)	0.47 (0.04,6.18)	0.53 (0.05,5.84)	0.53 (0.03,8.39)	0.52 (0.04,6.07)	0.55 (0.05,6.34)	0.71 (0.01,35.94)	0.67 (0.05,8.52)	0.68 (0.06,7.97)	0.70 (0.06,7.76)	0.86 (0.04,16.71)	1.08 (0.03,34.91)	1.18 (0.03,52.22)	*Alogliptin*	1.43 (0.03,72.03)	1.17 (0.10,13.82)
0.06 (0.00,2.34)	0.17 (0.00,6.21)	0.11 (0.00,10.25)	0.23 (0.01,6.51)	0.24 (0.01,7.98)	0.29 (0.01,8.98)	0.33 (0.01,8.15)	0.33 (0.01,10.16)	0.37 (0.01,10.01)	0.37 (0.01,13.14)	0.36 (0.01,10.25)	0.38 (0.01,10.76)	0.50 (0.01,46.09)	0.47 (0.02,14.07)	0.48 (0.02,13.47)	0.49 (0.02,13.29)	0.60 (0.01,25.10)	0.76 (0.01,47.79)	0.83 (0.01,68.18)	0.70 (0.01,35.20)	*Omarigliptin*	0.82 (0.03,23.31)
*0.08 (0.01,0.52)	0.20 (0.03,1.44)	0.14 (0.00,3.84)	0.28 (0.07,1.10)	0.29 (0.05,1.66)	0.35 (0.07,1.72)	0.40 (0.15,1.08)	0.40 (0.09,1.92)	0.45 (0.13,1.61)	0.45 (0.07,2.92)	0.44 (0.11,1.76)	0.47 (0.12,1.81)	0.61 (0.02,17.28)	0.57 (0.12,2.62)	0.58 (0.15,2.30)	0.59 (0.16,2.15)	0.73 (0.09,6.32)	0.92 (0.06,15.36)	1.01 (0.04,24.52)	0.85 (0.07,10.04)	1.22 (0.04,34.64)	*Dulaglutide*

Data presents RR [95%CIs]. Network meta-analysis results are presented as estimate effect sizes for the outcome of non-Hodgkin’s lymphoma risk. Interventions are reported in order of mean ranking of beneficially prophylactic effect on non-Hodgkin’s lymphoma risk, and outcomes are expressed as risk ratio (RR) (95% confidence intervals) (95%CIs). For the upper-right portion, RR of less than 1 indicates that the treatment specified in the row got more beneficial effect than that specified in the column. For the lower-left portion, RR of less than 1 indicates that the treatment specified in the column has more beneficial effect than that specified in the row. Bold results marked with * indicate statistical significance.

In this study, tirzepatide demonstrated a consistent and statistically significant protective association with hematologic malignancy, especially within the lymphoma spectrum. Clinical data on tirzepatide and cancer outcomes are still emerging [5], but preclinical work has suggested that tirzepatide may exert context-dependent effects on carcinogenesis by reshaping metabolic and proliferative pathways. For instance, Kong et al. reported that tirzepatide suppressed ErbB and glycolytic signaling in obese mouse models while enhancing phospholipase D and O-linked glycosylation pathways in lean animals [5]. The ErbB signaling family is involved in the pathobiology and prognosis of multiple tumor types, including non-Hodgkin’s lymphoma [6, 7], and its inhibition is currently being investigated as a therapeutic strategy [8]. Although our findings are consistent with these mechanistic clues, they largely derive from non-human models, and well-designed human studies will be essential to determine whether tirzepatide’s apparent protective effects translate into meaningful reductions in cancer incidence.

Several limitations warrant consideration. Although the total sample size and cumulative follow-up were substantial, the median follow-up of approximately 137.3 weeks may still be insufficient for detecting malignancies with very long latency periods [9]. While our focus on RCTs bolsters internal consistency, it also excludes large observational datasets that might provide additional insights into rare or late-onset cancers. Moreover, the wide variations of diagnostic criteria and reporting records across international trials would result in unavoidable heterogeneity in adverse-event meta-analyses and is often tolerated in early-stage pharmacovigilance research [10, 11]. In addition, the wide confidence interval in dose-stratification or histopathology-stratification analysis might reflect the fact that hematologic malignancy was naturally rare event in ordinary medical research. Although we arranged sensitivity test to re-appraise the main result, the potential effect of sparse event and small sample size still could not be ruled out.

In conclusion, this preliminary histopathology-focused NMA provides a potential direction of hematologic malignancy risk associated with antidiabetic agents. Our results demonstrate that tirzepatide is consistently associated with a reduced incidence of hematologic cancers—most notably lymphoid neoplasms such as non-Hodgkin’s lymphoma—whereas dulaglutide is uniquely linked to an increased overall risk. Linagliptin also exhibited a protective association, while other DPP-4 inhibitors, GLP-1 receptor agonists, and SGLT2 inhibitors did not show significant risk elevations.

### Duplicate submission statement

We previously published a preliminary network meta-analysis on hematologic malignancy risk with novel antidiabetic agents (*Biomolecules 2025, 15(11), 1622*). That study had key limitations: it only included GLP-1 receptor agonists and SGLT2 inhibitors and did not perform histopathology-based analyses of hematologic malignancy subtypes, leading to substantial residual heterogeneity. The present work was explicitly designed as an upgraded extension of that analysis. Here, we broadened the search, added DPP-4 inhibitors and dual agonists, and prespecified a histopathologic framework (leukemia, lymphoma, myeloma) to provide more granular and clinically relevant risk estimates. Although several randomized controlled trials (RCTs) overlap between the two projects, many additional RCTs were newly incorporated, and the pooled results, effect sizes, and interpretations are materially different. Accordingly, this manuscript does not represent duplicate submission.

## Supplementary Information

Additional file 1.

## Data Availability

All the data of the current study were available upon reasonable request to the corresponding authors.
