# Supplementary material for: Agent-specific, histopathology-stratified hematologic malignancy risk among dpp-4 inhibitors, glp-1 receptor agonists, and SGLT2 inhibitors: a network meta-analysis of 270,471 participants
Source: J Hematol Oncol. 2026 Apr 9;19:23. doi: 10.1186/s13045-026-01788-5 (PMC13063595; doi:10.1186/s13045-026-01788-5)
Supplement: Supplementary file 1 — Additional file 1. [file 13045_2026_1788_MOESM1_ESM.docx]

**List of content in supplement materials**

**Agent-Specific, Histopathology-Stratified Hematologic Malignancy Risk**

**Among DPP-4 Inhibitors, GLP-1 Receptor Agonists, and SGLT2 Inhibitors**

**A Network Meta-analysis of 270,471 Participants**

*Pao-Yen Lin, et al.*

| Appendix | Supplement method |
| --- | --- |
|  | Supplement result |
|  | PRISMA Flowchart |
| eFigure 1 | (A) Network structure of NMA of the primary outcome: overall hematologic malignancy risk |
|  | (B) Network structure of NMA of the primary outcome: overall hematologic malignancy risk in aspect of various dosage subgroups |
|  | (C) Network structure of NMA of the primary outcome: overall hematologic malignancy risk subgroup focusing RCTs with at least 1 year treatment duration |
|  | (D) Network structure of NMA of the primary outcome: overall hematologic malignancy risk subgroup excluding RCTs with high risk of bias |
|  | (E) Network structure of NMA of the primary outcome: overall hematologic malignancy risk subgroup focusing subjects with diabetes mellitus |
|  | (F) Network structure of NMA of the primary outcome: overall hematologic malignancy risk subgroup focusing subjects with age of at least 60 years old |
|  | (G) Network structure of NMA of the primary outcome: overall hematologic malignancy risk subgroup focusing subjects with age younger than 60 years old |
|  | (H) Network structure of the primary outcome: leukemia |
|  | (I) Network structure of the primary outcome: acute lymphocytic leukemia |
|  | (J) Network structure of the primary outcome: acute myeloid leukemia |
|  | (K) Network structure of the primary outcome: chronic lymphocytic leukemia |
|  | (L) Network structure of the primary outcome: chronic myeloid leukemia |
|  | (M) Network structure of the primary outcome: lymphoma |
|  | (N) Network structure of the primary outcome: non-Hodgkin's lymphoma |
|  | (O) Network structure of the primary outcome: B cell non-Hodgkin's lymphoma |
|  | (P) Network structure of the primary outcome: T cell non-Hodgkin's lymphoma |
|  | (Q) Network structure of the primary outcome: myeloma |
|  | (R) Network structure of the primary outcome: plasma cell myeloma |
|  | (S) Network structure of NMA of the acceptability: drop-out rate |
| eFigure 2 | (A) Forest plot of NMA of the primary outcome: overall hematologic malignancy risk |
|  | (B) Forest plot of NMA of the primary outcome: overall hematologic malignancy risk – Peto odds ratio |
|  | (C) Forest plot of NMA of the primary outcome: overall hematologic malignancy risk – hazard ratio based on time-to-event data |
|  | (D) Forest plot of NMA of the primary outcome: overall hematologic malignancy risk in aspect of various dosage subgroups |
|  | (E) Forest plot of NMA of the primary outcome: overall hematologic malignancy risk subgroup focusing RCTs with at least 1 year treatment duration |
|  | (F) Forest plot of NMA of the primary outcome: overall hematologic malignancy risk subgroup excluding RCTs with high risk of bias |
|  | (G) Forest plot of NMA of the primary outcome: overall hematologic malignancy risk subgroup focusing subjects with diabetes mellitus |
|  | (H) Forest plot of NMA of the primary outcome: overall hematologic malignancy risk subgroup focusing subjects with age of at least 60 years old |
|  | (I) Forest plot of NMA of the primary outcome: overall hematologic malignancy risk subgroup focusing subjects with age younger than 60 years old |
|  | (J) Forest plot of primary outcome: leukemia |
|  | (K) Forest plot of primary outcome: acute lymphocytic leukemia |
|  | (L) Forest plot of primary outcome: acute myeloid leukemia |
|  | (M) Forest plot of primary outcome: chronic lymphocytic leukemia |
|  | (N) Forest plot of primary outcome: chronic myeloid leukemia |
|  | (O) Forest plot of primary outcome: lymphoma |
|  | (P) Forest plot of primary outcome: B cell non-Hodgkin's lymphoma |
|  | (Q) Forest plot of primary outcome: T cell non-Hodgkin's lymphoma |
|  | (R) Forest plot of primary outcome: myeloma |
|  | (S) Forest plot of primary outcome: plasma cell myeloma |
|  | (T) Forest plot of NMA of the acceptability: drop-out rate |
| eFigure 3 | Individual study result of primary outcome: overall hematologic malignancy risk |
| eFigure 4 | (A) Funnel plot for primary outcome: overall hematologic malignancy risk |
|  | (B) Funnel plot for primary outcome: leukemia risk |
|  | (C) Funnel plot for primary outcome: acute lymphocytic leukemia risk |
|  | (D) Funnel plot for primary outcome: acute myeloid leukemia risk |
|  | (E) Funnel plot for primary outcome: chronic lymphocytic leukemia risk |
|  | (F) Funnel plot for primary outcome: chronic myeloid leukemia risk |
|  | (G) Funnel plot for primary outcome: lymphoma risk |
|  | (H) Funnel plot for primary outcome: non-Hodgkin's lymphoma risk |
|  | (I) Funnel plot for primary outcome: B cell non-Hodgkin's lymphoma risk |
|  | (J) Funnel plot for primary outcome: T cell non-Hodgkin's lymphoma risk |
|  | (K) Funnel plot for primary outcome: myeloma risk |
|  | (L) Funnel plot for primary outcome: plasma cell myeloma risk |
| eFigure 5 | (A) Egger test for primary outcome: overall hematologic malignancy risk |
|  | (B) Egger test for primary outcome: leukemia risk |
|  | (C) Egger test for primary outcome: acute lymphocytic leukemia risk |
|  | (D) Egger test for primary outcome: acute myeloid leukemia risk |
|  | (E) Egger test for primary outcome: chronic lymphocytic leukemia risk |
|  | (F) Egger test for primary outcome: chronic myeloid leukemia risk |
|  | (G) Egger test for primary outcome: lymphoma risk |
|  | (H) Egger test for primary outcome: non-Hodgkin's lymphoma risk |
|  | (I) Egger test for primary outcome: B cell non-Hodgkin's lymphoma risk |
|  | (J) Egger test for primary outcome: T cell non-Hodgkin's lymphoma risk |
|  | (K) Egger test for primary outcome: myeloma risk |
|  | (L) Egger test for primary outcome: plasma cell myeloma risk |
| eFigure 6 | Bayesian-based forest plot of NMA of primary outcome: overall hematologic malignancy risk |
| eFigure 7 | Risk of bias tool 2.0 |
| eTable 1 | PRISMA 2020 checklist of the current network meta-analysis |
| eTable 2 | Keyword used in each database and search results |
| eTable 3 | Dosage stratification (stratified according to the included original RCTs) |
| eTable 4 | Excluded studies and reason |
| eTable 5 | Characteristics of the included studies |
| eTable 6 | (A) League table of the primary outcome: overall hematologic malignancy risk |
|  | (B) League table of the primary outcome: leukemia |
|  | (C) League table of the primary outcome: acute lymphocytic leukemia |
|  | (D) League table of the primary outcome: acute myeloid leukemia |
|  | (E) League table of the primary outcome: chronic lymphocytic leukemia |
|  | (F) League table of the primary outcome: chronic myeloid leukemia |
|  | (G) League table of the primary outcome: lymphoma |
|  | (H) League table of the primary outcome: B cell non-Hodgkin's lymphoma |
|  | (I) League table of the primary outcome: T cell non-Hodgkin's lymphoma |
|  | (J) League table of the primary outcome: myeloma |
|  | (K) League table of the primary outcome: plasma cell myeloma |
|  | (L) League table of NMA of the acceptability: drop-out rate |
| eTable 7 | (A) SUCRA for primary outcome: overall hematologic malignancy risk |
|  | (B) SUCRA for primary outcome: leukemia risk |
|  | (C) SUCRA for primary outcome: acute lymphocytic leukemia risk |
|  | (D) SUCRA for primary outcome: acute myeloid leukemia risk |
|  | (E) SUCRA for primary outcome: chronic lymphocytic leukemia risk |
|  | (F) SUCRA for primary outcome: chronic myeloid leukemia risk |
|  | (G) SUCRA for primary outcome: lymphoma risk |
|  | (H) SUCRA for primary outcome: non-Hodgkin's lymphoma risk |
|  | (I) SUCRA for primary outcome: B cell non-Hodgkin's lymphoma risk |
|  | (J) SUCRA for primary outcome: T cell non-Hodgkin's lymphoma risk |
|  | (K) SUCRA for primary outcome: myeloma risk |
|  | (L) SUCRA for primary outcome: plasma cell myeloma risk |
| eTable 8 | (A) Heterogeneity for primary outcome: overall hematologic malignancy risk |
|  | (B) Heterogeneity for primary outcome: leukemia risk |
|  | (C) Heterogeneity for primary outcome: acute lymphocytic leukemia risk |
|  | (D) Heterogeneity for primary outcome: acute myeloid leukemia risk |
|  | (E) Heterogeneity for primary outcome: chronic lymphocytic leukemia risk |
|  | (F) Heterogeneity for primary outcome: chronic myeloid leukemia risk |
|  | (G) Heterogeneity for primary outcome: lymphoma risk |
|  | (H) Heterogeneity for primary outcome: non-Hodgkin's lymphoma risk |
|  | (I) Heterogeneity for primary outcome: B cell non-Hodgkin's lymphoma risk |
|  | (J) Heterogeneity for primary outcome: T cell non-Hodgkin's lymphoma risk |
|  | (K) Heterogeneity for primary outcome: myeloma risk |
|  | (L) Heterogeneity for primary outcome: plasma cell myeloma risk |
| eTable 9 | (A) Side-splitting model inconsistency for primary outcome: overall hematologic malignancy risk |
|  | (B) Side-splitting model inconsistency for primary outcome: leukemia risk |
|  | (C) Side-splitting model inconsistency for primary outcome: acute lymphocytic leukemia risk |
|  | (D) Side-splitting model inconsistency for primary outcome: acute myeloid leukemia risk |
|  | (E) Side-splitting model inconsistency for primary outcome: chronic lymphocytic leukemia risk |
|  | (F) Side-splitting model inconsistency for primary outcome: chronic myeloid leukemia risk |
|  | (G) Side-splitting model inconsistency for primary outcome: lymphoma risk |
|  | (H) Side-splitting model inconsistency for primary outcome: non-Hodgkin's lymphoma risk |
|  | (I) Side-splitting model inconsistency for primary outcome: B cell non-Hodgkin's lymphoma risk |
|  | (J) Side-splitting model inconsistency for primary outcome: T cell non-Hodgkin's lymphoma risk |
|  | (K) Side-splitting model inconsistency for primary outcome: myeloma risk |
|  | (L) Side-splitting model inconsistency for primary outcome: plasma cell myeloma risk |
|  | (M) Design-by-treatment model and loop inconsistency for all primary outcomes |
| eTable 10 | GRADE for primary outcome: overall hematologic malignancy risk |

**Appendix:**

**Supplement Methods**

**Study Design and Analytical Framework**

We designed this NMA with a prespecified focus on hematologic malignancy risk associated with modern antihyperglycemic agents, including DPP-4 inhibitors, GLP-1 receptor agonists, and SGLT2 inhibitors. Methodology followed Cochrane Collaboration standards for evaluating drug-related adverse events [1], prioritizing malignancy-related outcomes rather than general tolerability. Reporting adhered to the PRISMA extension for network meta-analyses (PRISMA-NMA) (see eTable 1) [2]. The protocol was prospectively registered in PROSPERO (CRD420251151419), and institutional review board approval was obtained from Tri-Service General Hospital (TSGHIRB E202516007).

**Literature Search Strategy**

A systematic search was implemented across eight databases—PubMed, Embase, Cochrane CENTRAL, ClinicalTrials.gov, ProQuest, ScienceDirect, Web of Science, and ClinicalKey—from inception through 19 September 2025. Two investigators (PT Tseng and BY Zeng) independently screened titles and abstracts, followed by full-text review of potentially eligible reports. Discrepancies were resolved through discussion. Reference lists of prior meta-analyses and relevant reviews were additionally screened to identify any missed trials. No language restrictions were applied.

**Eligibility Criteria**

Study selection followed a PICOS framework:

- **Population:** Adult participants without a previous diagnosis of hematologic malignancy
- **Intervention:** Any eligible antidiabetic agent listed below, at any approved dose
- **Comparator:** Placebo, usual care, or active comparator
- **Outcome:** Newly diagnosed hematologic malignancy
- **Study Design:** Randomized controlled trials (RCTs)

Trials were eligible if they enrolled adults free of hematologic cancer at baseline, evaluated at least one of the prespecified antidiabetic agents in human participants, and either explicitly reported hematologic malignancies or provided structured adverse-event data from which such outcomes could be extracted. Eligible therapies included incretin-based drugs (DPP-4 inhibitors and GLP-1 receptor agonists), SGLT2 inhibitors, dual agonists such as tirzepatide, and other novel agents (e.g., retatrutide, imeglimin). Exclusion criteria were: (1) inclusion of participants with pre-existing hematologic malignancy, (2) absence of a comparison arm, (3) no hematologic outcome reporting, (4) non-randomized design, (5) animal or preclinical studies, (6) pediatric populations, or (7) inadequate randomization or substantial baseline imbalance. To minimize reporting bias, we included only RCTs that adopted structured, systematic approaches to safety monitoring [3].

**Risk of Bias Assessment**

Two reviewers independently appraised each eligible trial using the Cochrane Risk of Bias 2.0 tool [4]. Any disagreements were resolved by consensus with a third investigator.

**Outcome Definition and Histopathology Classification**

Hematologic malignancy outcomes were defined with reference to the Global Burden of Disease framework [5] and the World Health Organization (WHO) 5th edition classification of hematolymphoid neoplasms [6]. Malignancies were categorized into:

- **Leukemia:** Including acute lymphocytic leukemia, acute myeloid leukemia, chronic lymphocytic leukemia, and chronic myeloid leukemia
- **Lymphoma:** Including non-Hodgkin’s lymphoma (e.g., B-cell and T-cell subtypes, adenolymphoma, lymphocytic lymphoma) and Hodgkin’s lymphoma
- **Myeloma:** Including multiple myeloma and plasma cell myeloma

We analyzed hematologic malignancy both as an overall composite endpoint and stratified by these histopathologic groups to highlight subtype-specific patterns. To approximate treatment acceptability, we also extracted trial discontinuation rates, following established NMA approaches that use drop-out rates as a proxy for overall tolerability [7,8].

**Dose Stratification and Subgrouping**

To explore dose–response relationships, we conducted subgroup analyses based on dosing regimens reported in the original RCTs (eTable 3) [9-82]. Interventions were categorized into low-, medium-, and high-dose strata when trial designs allowed such differentiation.

In order to explore the potentially confounding effects related to treatment duration, risk of bias of included RCT, baseline diseases (i.e. diabetes or obesity), and age, we arrange subgroup analyses based on treatment duration (at least 1 year), risk of bias of included RCT (excluding high risk of bias RCT), baseline diseases (i.e. diabetes or obesity), and age (at least 60 years old or younger than 60 years old). We choose baseline diseases with diabetes mellitus and obesity because these two diseases were the major indications of such medication. The “1 year treatment duration” was chosen to represent the cut-off point in our study for treatment duration stratification based on the frequently recommended period for long term treatment or follow-up in several guidelines or large-scale trials [83]. We choose 60 years old to be our cut-off points of age stratification based on the evidence regarding prediction of hematologic malignancy in the previous study [84].

**Data Extraction Process**

Two authors (PT Tseng and BY Zeng) independently collected information on study design, participant characteristics, treatment regimens, hematologic malignancy outcomes, and discontinuation events using standardized forms. When key data were unclear or missing, we attempted to contact corresponding authors for clarification. Data extraction procedures followed recommendations from the Cochrane Handbook for Systematic Reviews of Interventions [85].

**Statistical Analysis**

We performed a frequentist random-effects NMA using the network suite in STATA version 16.0 (StataCorp, College Station, TX) [86]. Contrast-based models were applied to integrate both direct and indirect comparisons across the treatment network [87]. Effect estimates were summarized as risk ratios (RRs) with 95% confidence intervals (CIs). To rank interventions according to their hematologic safety profiles, we calculated Surface Under the Cumulative Ranking (SUCRA) values [88]. Between-study heterogeneity was assessed via τ² estimates, and network inconsistency was evaluated using loop-specific, node-splitting, and design-by-treatment interaction approaches [89]. The certainty of evidence was graded using the GRADE framework [90].

Potential small-study effects and publication bias were examined by visual inspection of comparison-adjusted funnel plots and by Egger’s regression tests. To test robustness, we performed Bayesian sensitivity analyses using the netmeta module in MetaInsight v4.0.2 [91,92], which accommodates zero-event trials without the need for continuity corrections [93,94]. This application of Bayesian NMA can be advantageous in sparse-event settings because it reduces reliance on arbitrary continuity corrections and offers an additional robustness check under alternative modeling assumptions, thereby strengthening the credibility of inference when events are infrequent [95,96].

**Sensitivity test**

Although there is no universally accepted threshold for defining sparse-event meta-analysis, we considered hematology-related outcomes to be uncommon and potentially subject to sparse-data problems in routine medical research. Therefore, we prespecified a two-step strategy to assess the robustness of our findings to potential sparse-event bias.

First, we performed sensitivity analysis using Bayesian NMA mentioned above (by *multinma* package in R) [97], which may be particularly informative in sparse-event settings, as it can lessen dependence on arbitrary continuity corrections and permit sensitivity assessment under different modeling assumptions, thereby enhancing confidence in the resulting estimates when events were rare [95,96]. We also performed sensitivity analysis using NMA based on hazard ratio estimates derived from time-to-event data, when available, because this approach can retain event-time and censoring information [98].

Second, in parallel, we conducted traditional pair-wise meta-analyses [by Comprehensive Meta-Analysis (version 3; Biostat, Englewood, NJ, USA)] using Peto odds ratios [99] to re-evaluate whether sparse-event conditions substantially influenced the primary findings of our study.

**Ethics**

All procedures were conducted in accordance with the Declaration of Helsinki and relevant institutional regulations.

**Supplement Results**

**Summary of Main Findings:**

Overall, tirzepatide was associated with a statistically significant reduction in the incidence of hematologic malignancies, with the most pronounced effect observed for lymphoma, particularly non-Hodgkin’s lymphoma. In contrast, dulaglutide was uniquely linked to a higher overall risk of hematologic cancers. No other antidiabetic agent demonstrated a statistically significant increase in hematologic malignancy risk, helping to clarify concerns raised by prior, less granular analyses.

**Study Selection and Characteristics of Included Trials:**

The PRISMA flow diagram summarizing study selection is shown in Appendix: PRISMA Flowchart. After excluding 294 records that did not meet eligibility criteria (details in eTable 3), 75 RCTs from 74 publications were included in the final quantitative synthesis (eTable 4) [9-82]. Neal, B. (2017) [53] consisted of data from CANVAS and CANVAS-R. Collectively, these trials enrolled 270,471 adults, with a mean age of 62.9 years (range: 41.2–72.6 years) and an average female proportion of 37.5% (range: 20.8%–79.0%). Median follow-up was 137.3 weeks (range: 12–338 weeks), providing substantial cumulative exposure time.

The treatment network comprised the following interventions:

- **DPP-4 inhibitors:** alogliptin, linagliptin, omarigliptin, saxagliptin, sitagliptin, vildagliptin
- **GLP-1 receptor agonists:** albiglutide, dulaglutide, efpeglenatide, exenatide, liraglutide, lixisenatide, semaglutide
- **SGLT2 inhibitors:** canagliflozin, bexagliflozin, dapagliflozin, empagliflozin, ertugliflozin, sotagliflozin
- **Dual agonist:** tirzepatide

Agents such as teneligliptin, retatrutide, imeglimin, dorzagliatin, petrelintide, and teplizumab were considered during screening but ultimately excluded from the NMA because hematologic cancer outcomes were not reported.

**Primary Outcome: Overall Hematologic Malignancy Risk**

When all hematologic malignancies were analyzed as a single composite, dulaglutide was the only agent associated with a significantly elevated risk compared with control (RR = 2.17; 95% CIs = 1.14–4.17). Conversely, tirzepatide (RR = 0.22; 95% CIs = 0.06–0.78) and linagliptin (RR = 0.51; 95% CIs = 0.27–0.95) showed significantly lower risks. Among all therapies assessed, tirzepatide yielded the most favorable ranking for overall hematologic malignancy prevention (eFigure 1A, eFigure 2A, eFigure 3, and eTable 6A).

Dose-stratified analyses indicated that the protective effect of linagliptin was driven by the standard 5 mg/day regimen, which retained a significant reduction in overall hematologic malignancy risk (RR = 0.51; 95% CIs = 0.27–0.95). In contrast, medium-dose dulaglutide (1.5 mg/week) was associated with an increased risk (RR = 2.25; 95% CIs = 1.17–4.33), and low-dose empagliflozin (1–10 mg/day) also showed a higher overall risk (RR = 1.96; 95% CIs = 1.05–3.67). For tirzepatide, dose-specific analyses suggested a protective trend that did not reach statistical significance, likely owing to limited sample size in individual dose strata (eFigure 1B and eFigure 2B).

*Sensitivity test of Primary outcomes:*

In the Peto odds ratio method, the main results remained similar findings. Specifically, dulaglutide was the only agent associated with a significantly elevated risk compared with control (Peto odds ratio = 2.24; 95% CIs = 1.21–4.14). Conversely, tirzepatide (Peto odds ratio = 0.12; 95% CIs = 0.03–0.58) and linagliptin (Peto odds ratio = 0.51; 95% CIs = 0.28–0.92) showed significantly lower risks (eFigure 2B). Regarding hazard ratio, dulaglutide was still the only agent associated with a significantly elevated risk compared with control (hazard ratio = 2.17; 95% CIs = 1.13–4.17). Conversely, tirzepatide (hazard ratio = 0.22; 95% CIs = 0.06–0.78) and linagliptin (hazard ratio = 0.51; 95% CIs = 0.27–0.95) showed significantly lower risks (eFigure 2C).

*Subgroup Analysis of Primary outcomes:*

Treatment duration-stratified analyses indicated that dulaglutide was still the only agent associated with a significantly elevated risk compared with control (RR = 2.17; 95% CIs = 1.11–4.25) in RCTs with treatment duration at least 1 year. Conversely, linagliptin (RR = 0.51; 95% CIs = 0.26–0.97) showed significantly lower risks in RCTs with treatment duration at at least 1 year (eFigure 1C and eFigure 2E). However, we could not perform subgroup of RCTs with treatment duration less than 1 year due to insufficient numbers of RCTs to form a network.

When we excluded RCTs with high Risk of Bias, we noticed that dulaglutide was still the only agent associated with a significantly elevated risk compared with control (RR = 2.17; 95% CIs = 1.14–4.17). Conversely, tirzepatide (RR = 0.22; 95% CIs = 0.06–0.78) and linagliptin (RR = 0.51; 95% CIs = 0.27–0.95) showed significantly lower risks (eFigure 1D and eFigure 2F).

When we focused on subjects with diabetes mellitus, the main results revealed that dulaglutide was still the only agent associated with a significantly elevated risk compared with control (RR = 2.17; 95% CIs = 1.14–4.17). Conversely, tirzepatide (RR = 0.14; 95% CIs = 0.02–0.98) and linagliptin (RR = 0.51; 95% CIs = 0.27–0.95) showed significantly lower risks (eFigure 1E and eFigure 2G). However, we could not perform subgroup of subjects with obesity due to insufficient numbers of RCTs to form a network.

In age-stratification subgroup analysis, the main results revealed that dulaglutide was still the only agent associated with a significantly elevated risk compared with control (RR = 2.25; 95% CIs = 1.09–4.65) in subjects at least 60 years old. Conversely, linagliptin (RR = 0.50; 95% CIs = 0.26–0.98) showed significantly lower risks in subjects at least 60 years old (eFigure 1F and eFigure 2H). On the other hand, in subgroup of subjects younger than 60 years old, only tirzepatide (RR = 0.18; 95% CIs = 0.04–0.86) showed significantly lower risks (eFigure 1G and eFigure 2I).

*Subgroup Analysis: Leukemia Subtypes*

In analyses restricted to leukemia as a group, as well as to individual leukemia subtypes (acute lymphocytic leukemia, acute myeloid leukemia, chronic lymphocytic leukemia, and chronic myeloid leukemia), no antidiabetic agent demonstrated a statistically significant association with increased or decreased risk. All effect estimates crossed unity, and no consistent signal emerged for any specific drug (eFigure 1H–1L, eFigure 2J–2N, and eTable 6B–6F).

*Subgroup Analysis: Lymphoma Spectrum Tumors*

Within the lymphoma spectrum, tirzepatide was the only agent associated with a statistically significant reduction in overall lymphoma incidence (RR = 0.20; 95% CIs = 0.04–0.99). When further stratified, tirzepatide also showed a marked protective association with non-Hodgkin’s lymphoma (RR = 0.20; 95% CIs = 0.04–0.98). For other lymphoma subtypes—such as adenolymphoma, lymphocytic lymphoma, and Hodgkin’s lymphoma—data were too sparse to support NMA estimation, and no robust conclusions could be drawn (Figure 1, Table 1, eFigure 1M–1P, eFigure 2O–2Q, and eTable 6G–6I).

*Subgroup Analysis: Myeloma and Related Malignancies*

For myeloma-spectrum diseases, including multiple myeloma and plasma cell myeloma, none of the evaluated agents showed a statistically significant association with risk relative to control (eFigure 1Q–1R, eFigure 2R–2S, and eTable 6J–6K). Attempts to further isolate outcomes for multiple myeloma were hindered by the limited number of events and incomplete subtype reporting.

**Acceptability Based on Drop-Out Rates**

We used trial discontinuation rates as an indirect measure of treatment acceptability. Several drugs were associated with significantly lower drop-out rates compared with control, suggesting better overall tolerability: tirzepatide (RR = 0.63; 95% CIs = 0.52–0.77), liraglutide (RR = 0.70; 95% CIs = 0.58–0.85), canagliflozin (RR = 0.77; 95% CIs = 0.60–0.98), and semaglutide (RR = 0.77; 95% CIs = 0.63–0.96) (eFigure 1S, eFigure 2T, and eTable 6L).

**Assessment of Bias, Treatment Ranking, and Heterogeneity**

Visual inspection of comparison-adjusted funnel plots did not reveal obvious asymmetry suggestive of small-study effects (eFigure 4A–4L), and Egger’s tests were non-significant (eFigure 5A–5L). SUCRA rankings consistently placed tirzepatide at the top for minimizing hematologic malignancy risk across a range of analyses (eTable 7A–7L). Overall heterogeneity was low according to τ² estimates (eTable 8A–8L), and network inconsistency was minimal based on loop-specific, node-splitting, and design-by-treatment interaction tests (eTable 9A–9M).

Bayesian sensitivity analyses corroborated the principal findings, particularly the protective association for tirzepatide, and were robust to alternative modeling strategies that handled zero-event trials without continuity corrections (eFigure 6).

**Risk of Bias and Certainty of Evidence**

According to the Cochrane Risk of Bias 2.0 assessment, 56 of the 75 trials (74.7%) were classified as having low risk of bias, 14 (18.7%) as having some concerns, and 5 (6.6%) as high risk (eFigure 7). Using the GRADE framework, the certainty of evidence for most key comparisons ranged from moderate to high (eTable 10), with downgrades primarily related to imprecision and wide CIs.

**Appendix: PRISMA Flowchart**

**
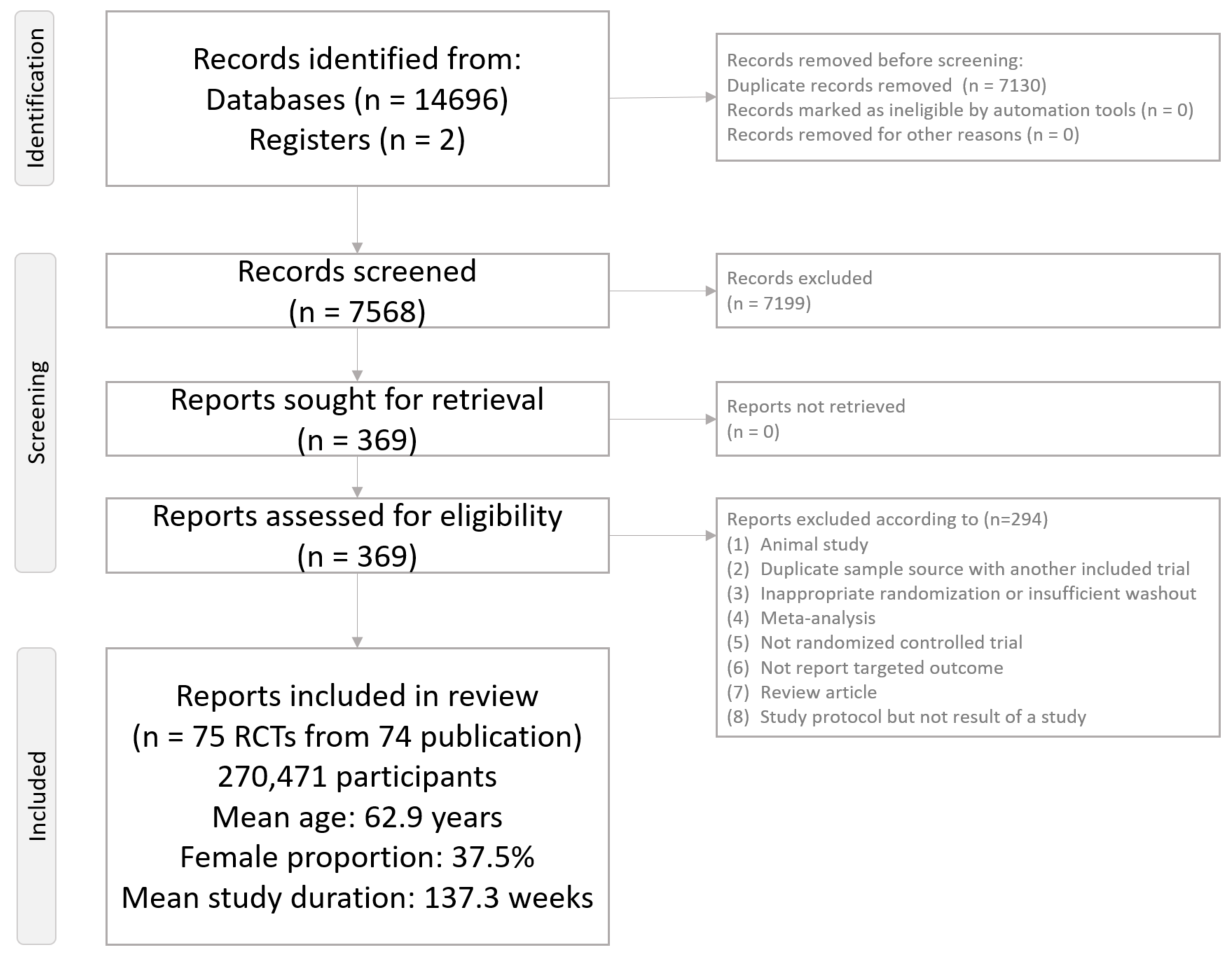
**

**eFigure 1A Network structure of the primary outcome: overall hematologic malignancy**

**
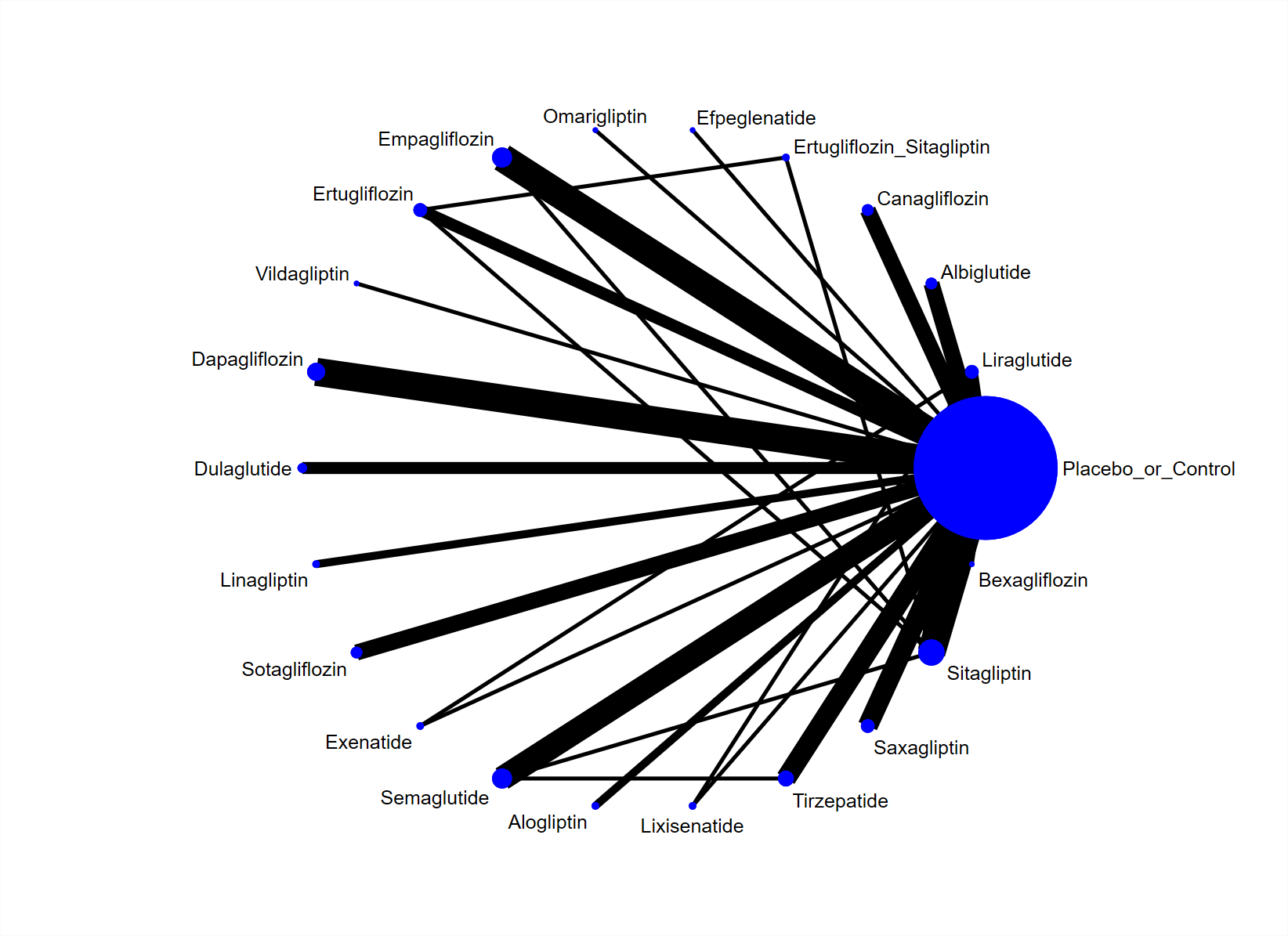
**

**eFigure 1B Network structure of NMA of the primary outcome: overall hematologic malignancy risk in aspect of various dosage subgroups**

**
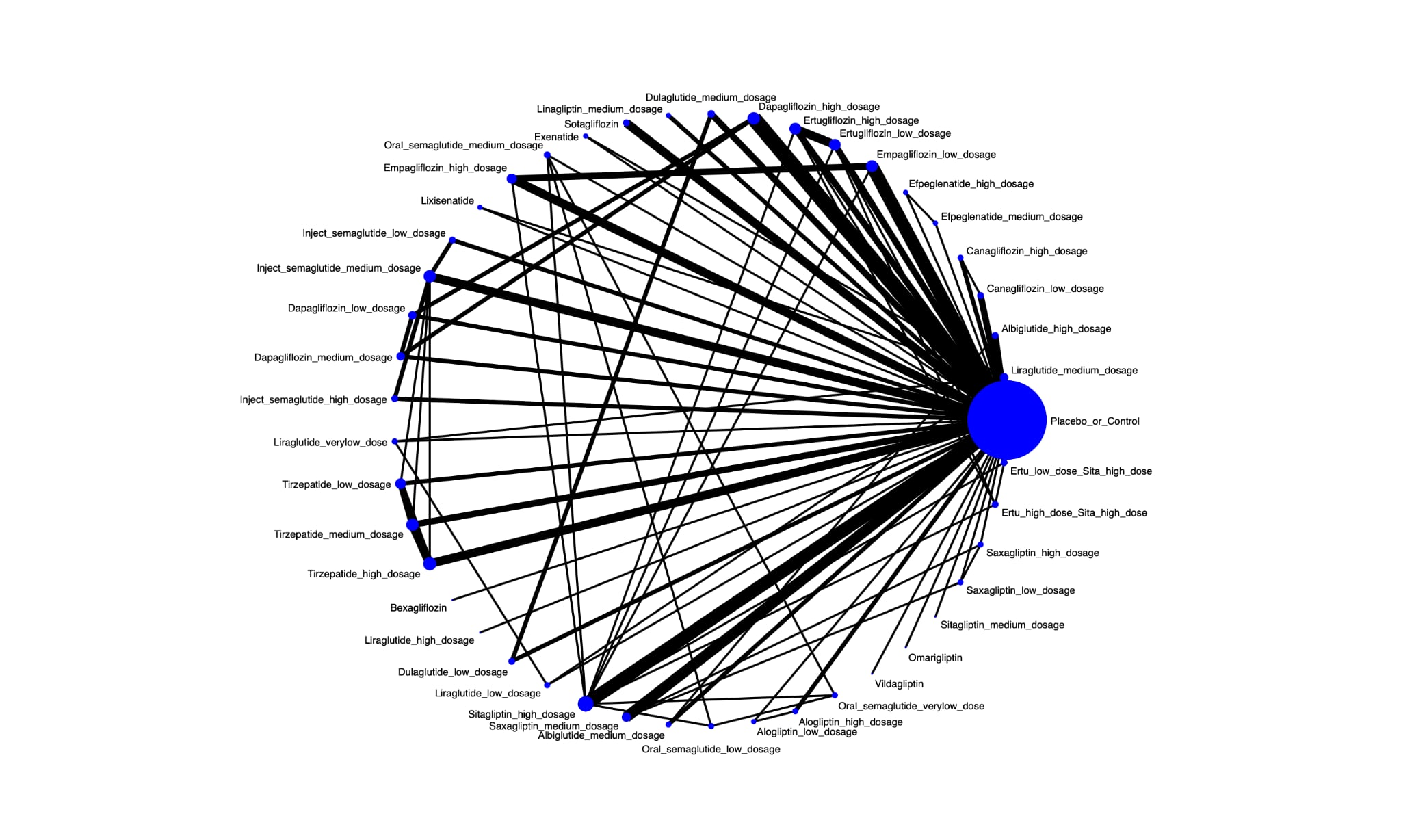
**

**eFigure 1C Network structure of NMA of the primary outcome: overall hematologic malignancy risk subgroup focusing RCTs with at least 1 year treatment duration**

**
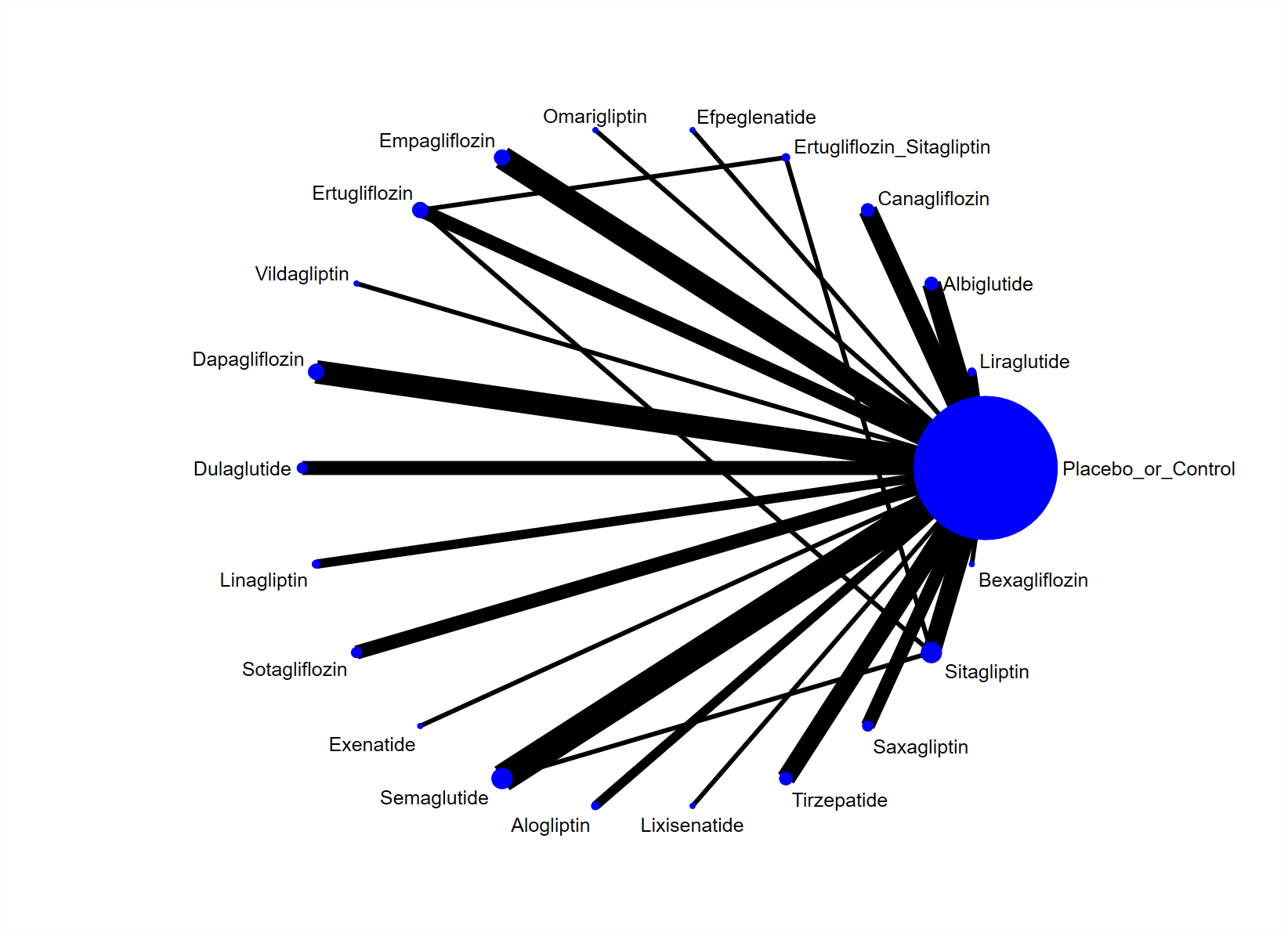
**

**eFigure 1D Network structure of NMA of the primary outcome: overall hematologic malignancy risk subgroup excluding RCTs with high risk of bias**

**
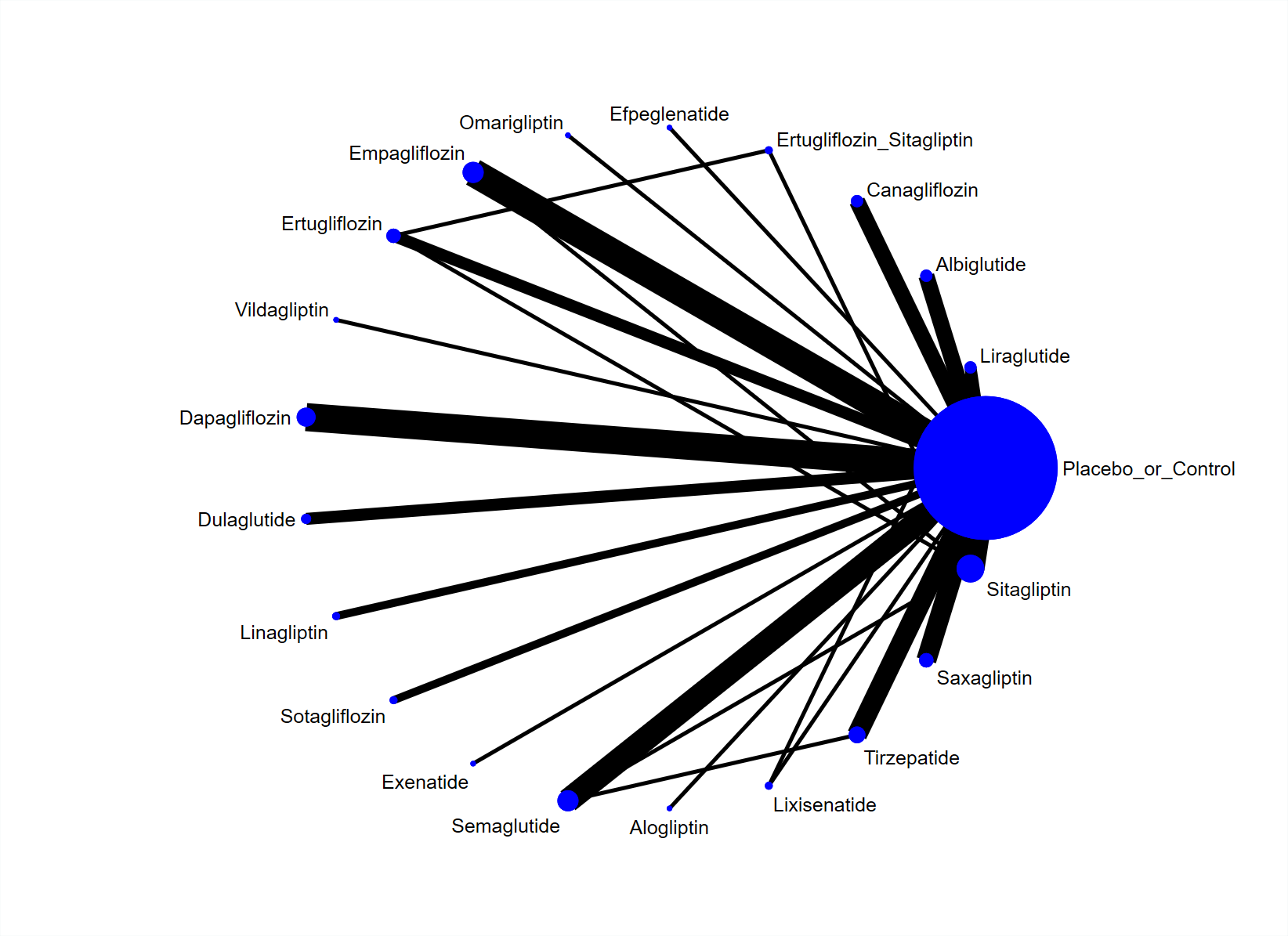
**

**eFigure 1E Network structure of NMA of the primary outcome: overall hematologic malignancy risk subgroup focusing subjects with diabetes mellitus**

**
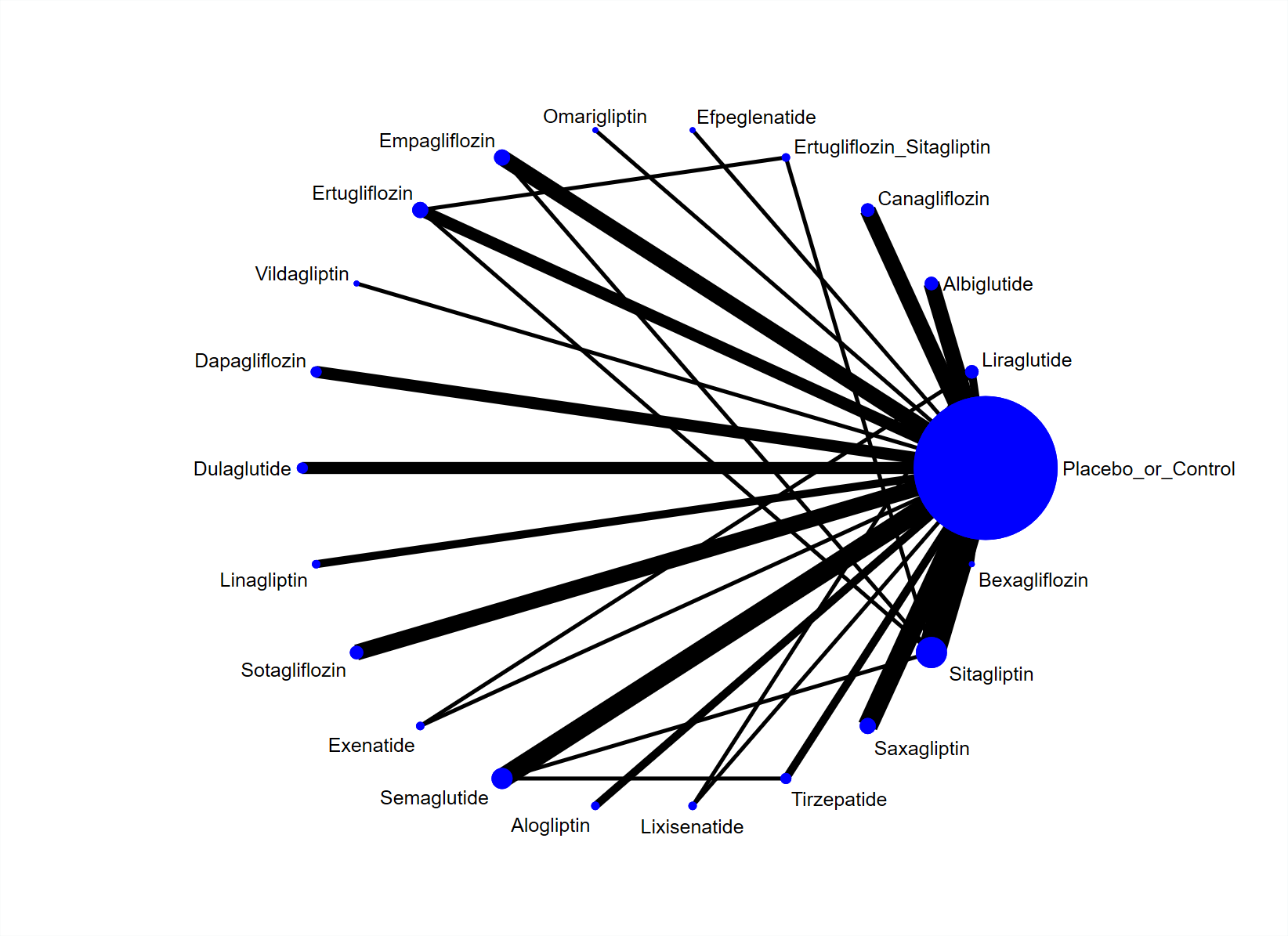
**

**eFigure 1F Network structure of NMA of the primary outcome: overall hematologic malignancy risk subgroup focusing subjects with age of at least 60 years old**

**
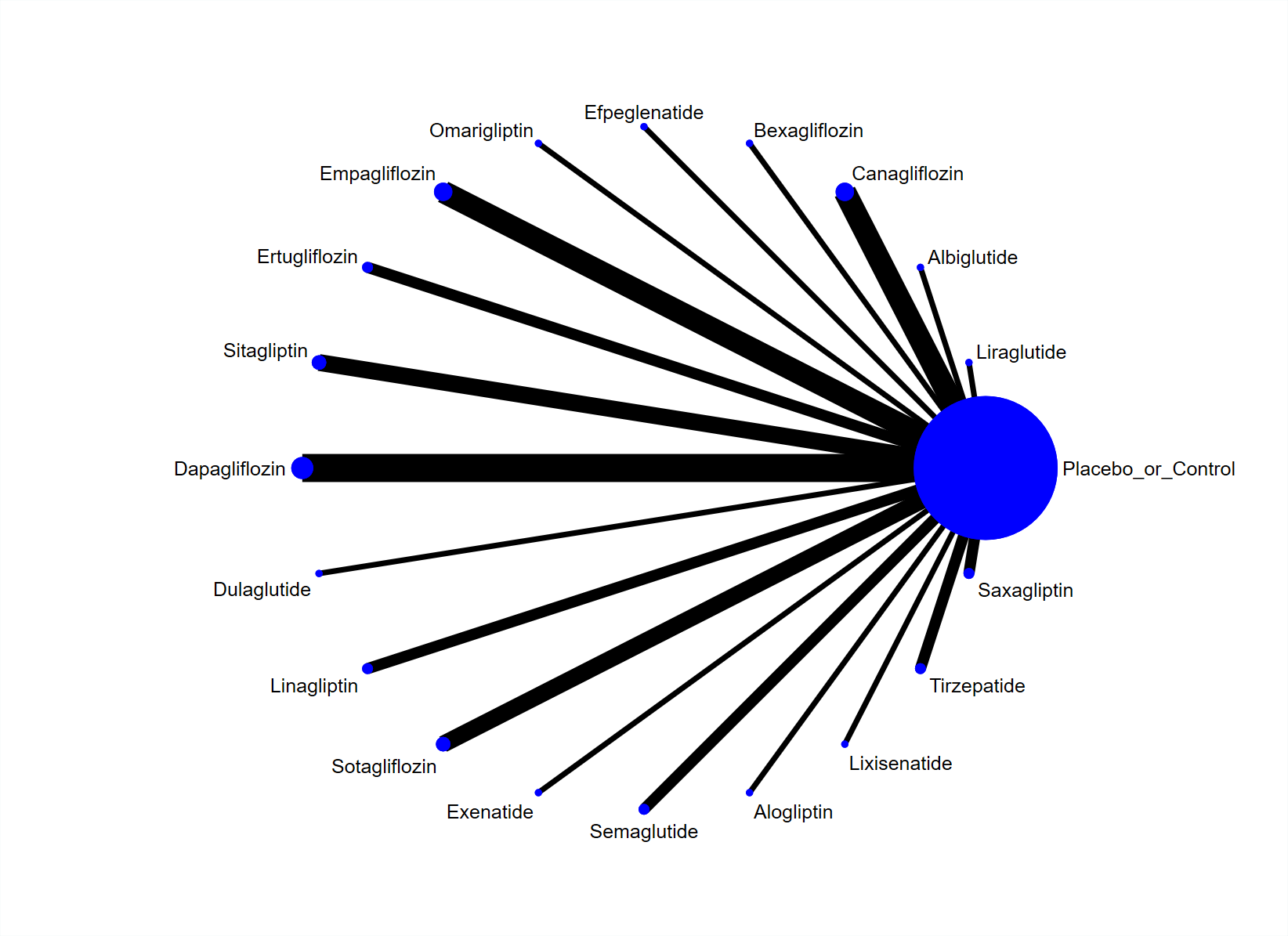
**

**eFigure 1G Network structure of NMA of the primary outcome: overall hematologic malignancy risk subgroup focusing subjects with age younger than 60 years old**

**
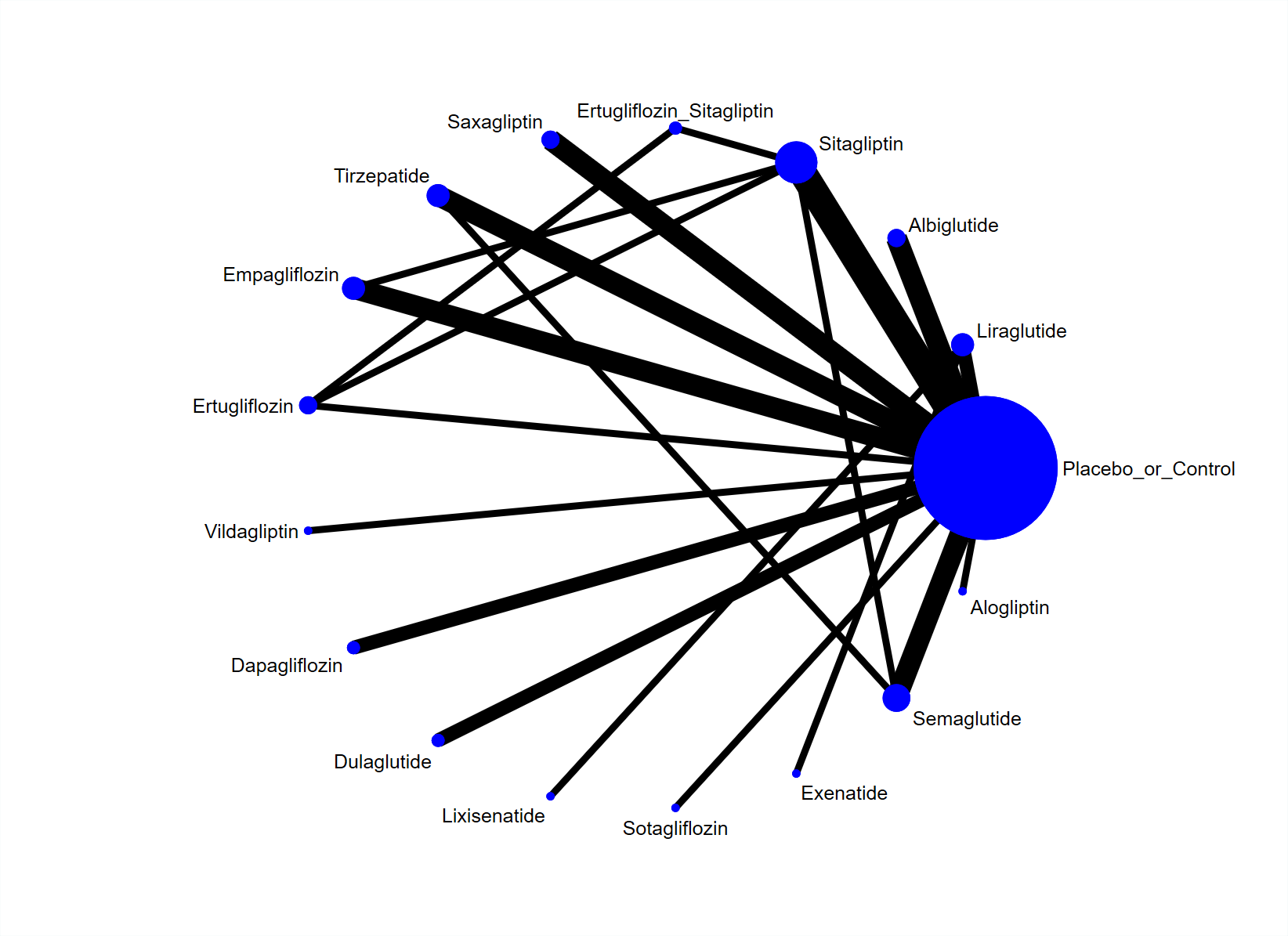
**

**eFigure 1H Network structure of the primary outcome: leukemia**

**
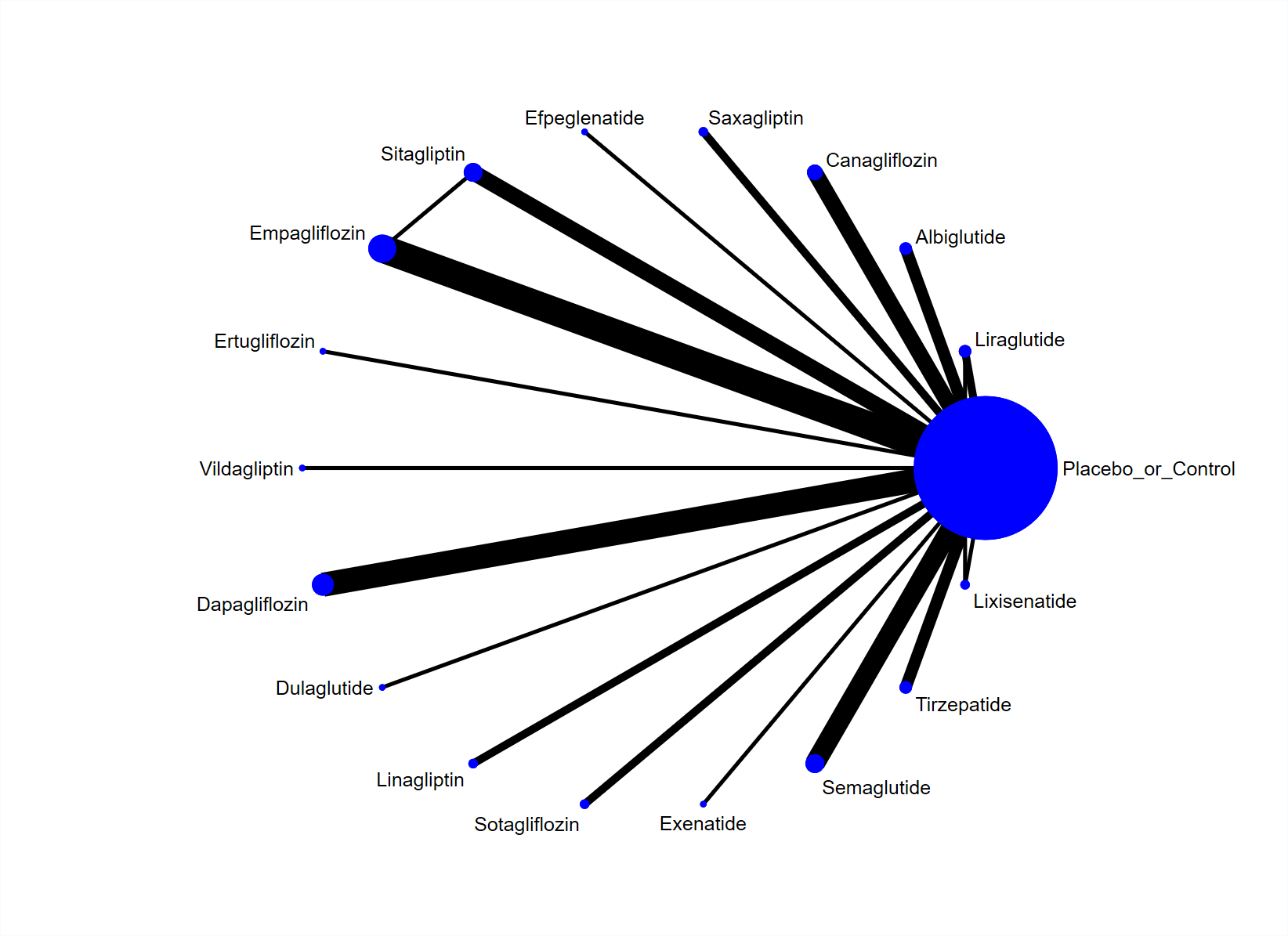
**

**eFigure 1I Network structure of the primary outcome: acute lymphocytic leukemia**

**
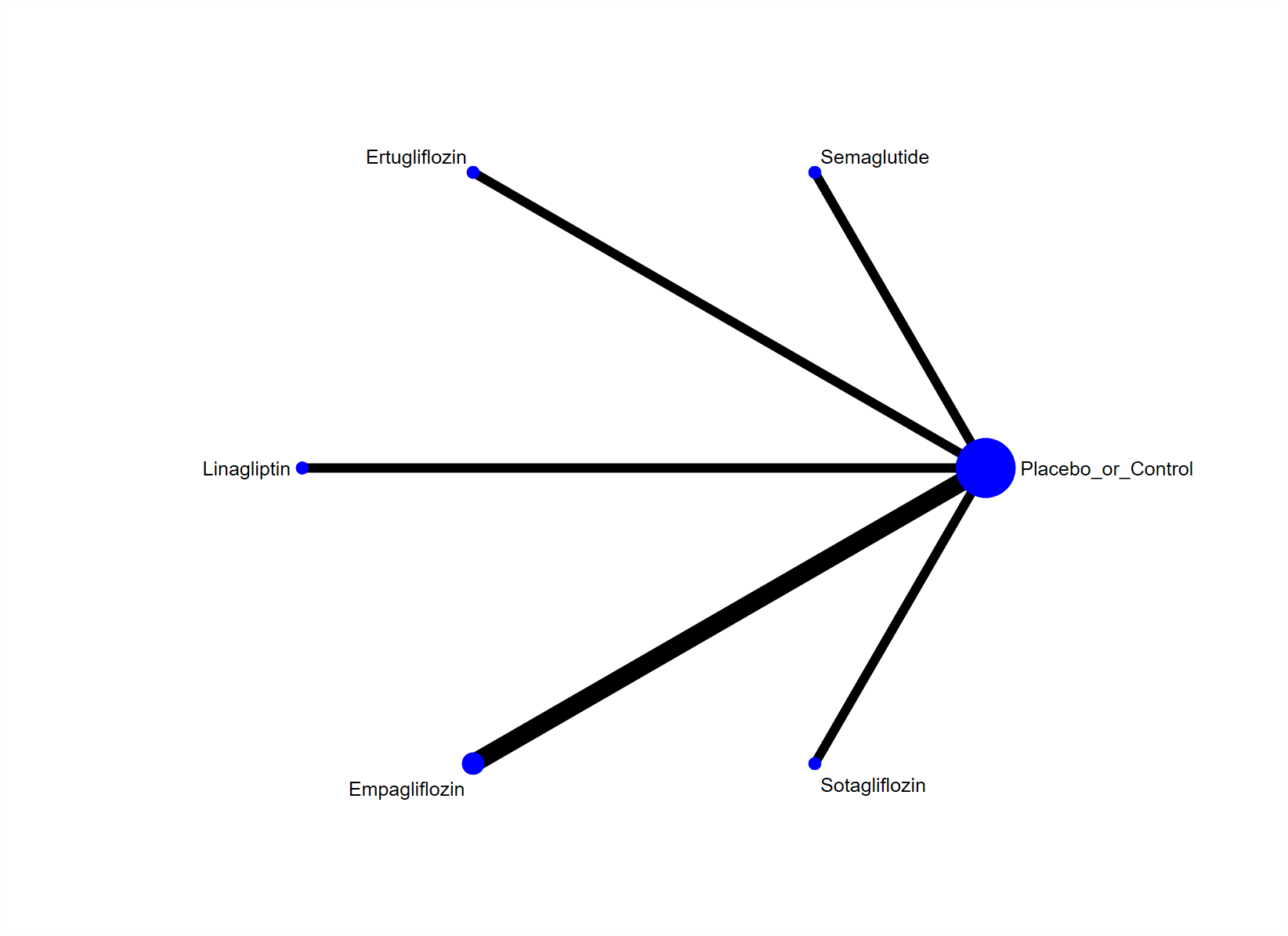
**

**eFigure 1J Network structure of the primary outcome: acute myeloid leukemia**

**
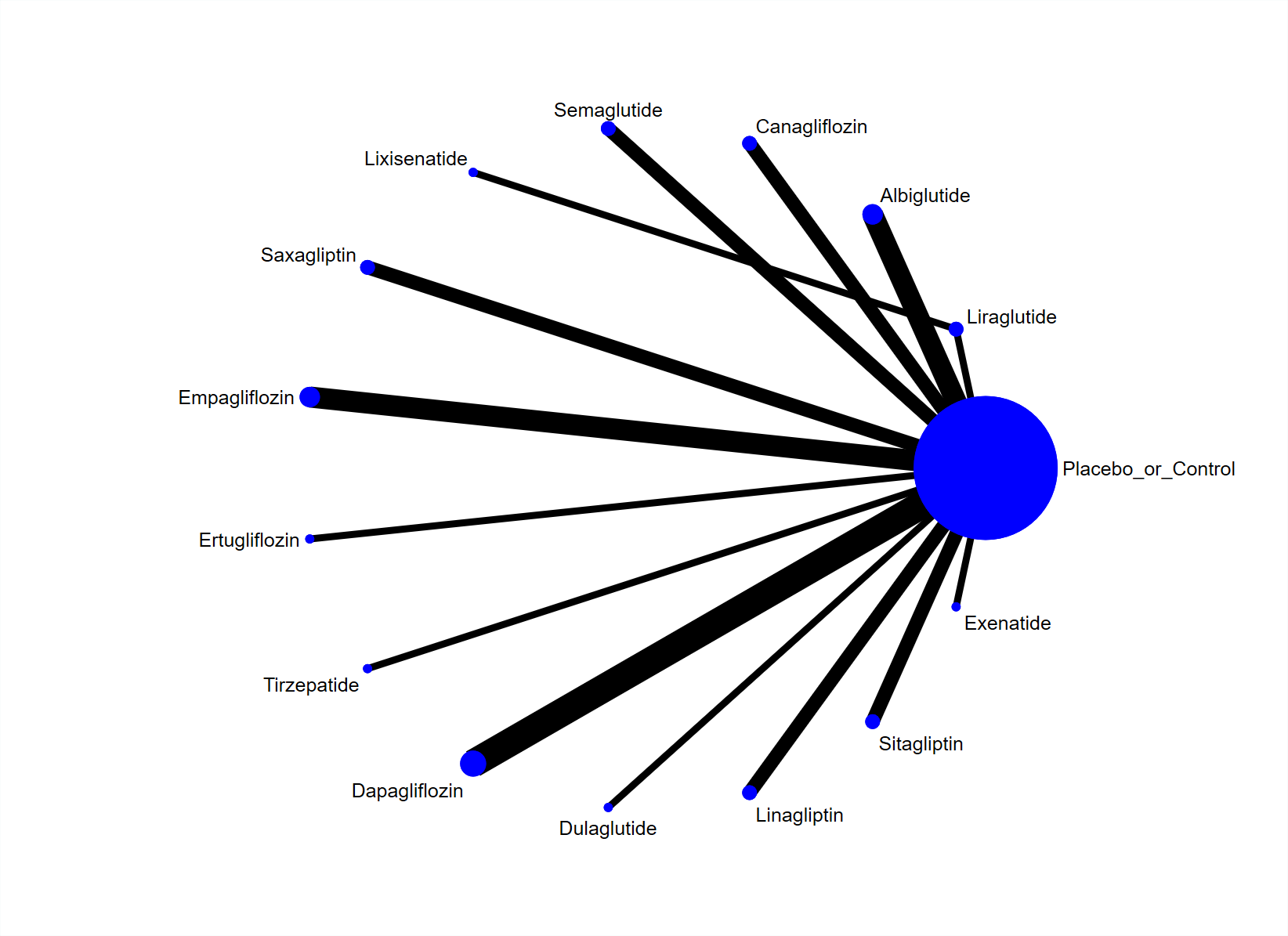
**

**eFigure 1K Network structure of the primary outcome: chronic lymphocytic leukemia**

**
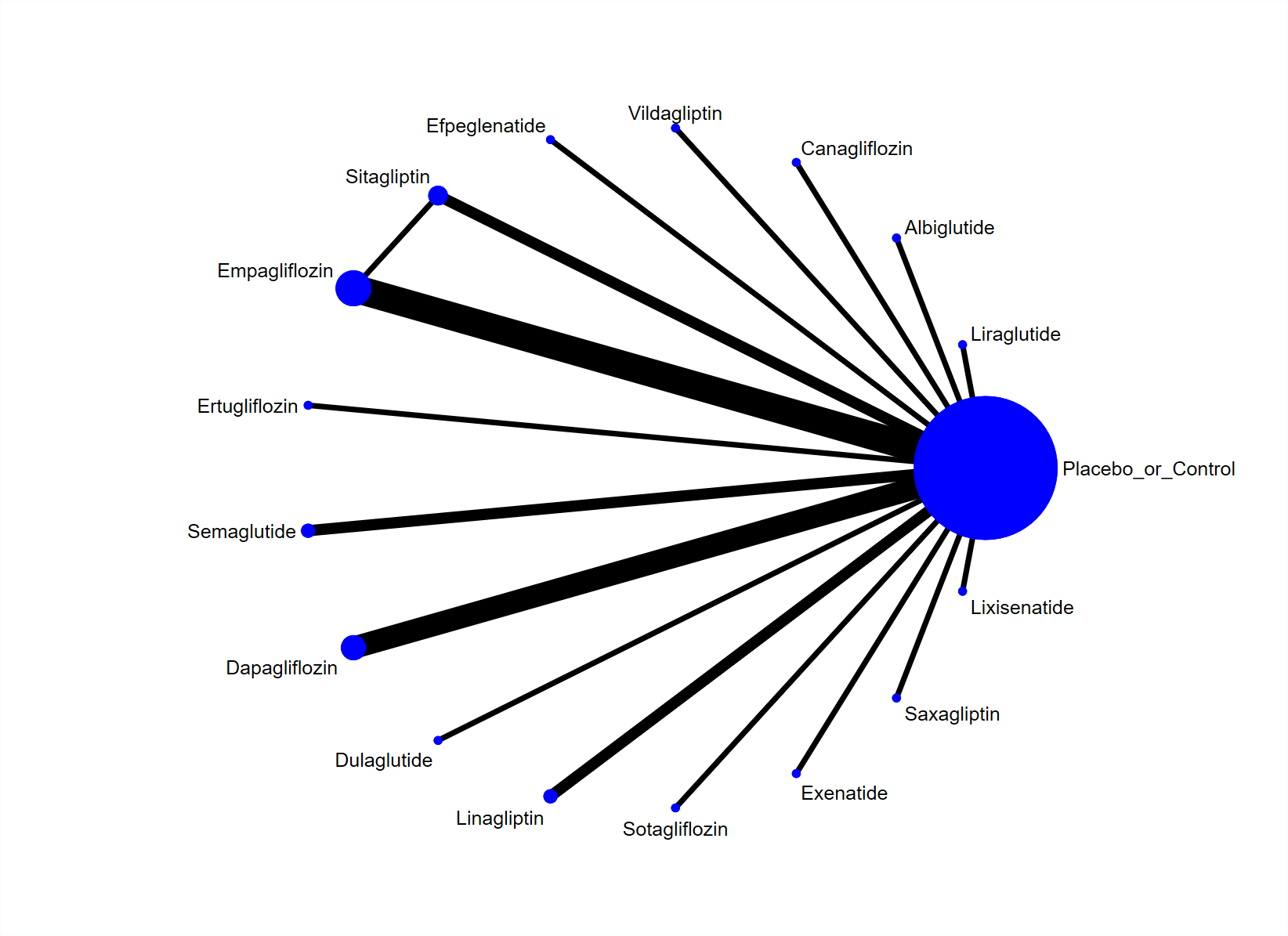
**

**eFigure 1L Network structure of the primary outcome: chronic myeloid leukemia**

**
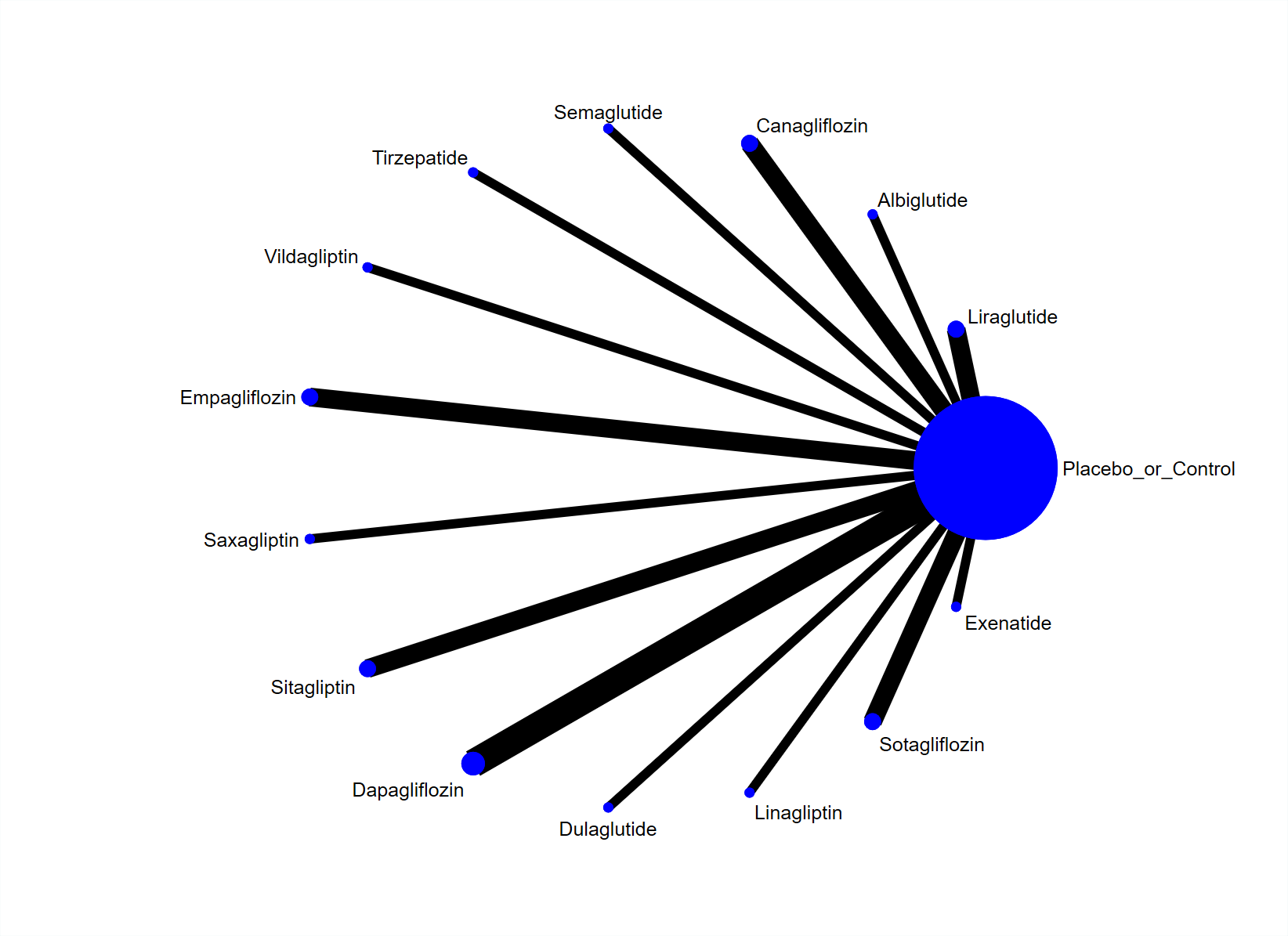
**

**eFigure 1M Network structure of the primary outcome: lymphoma**

**
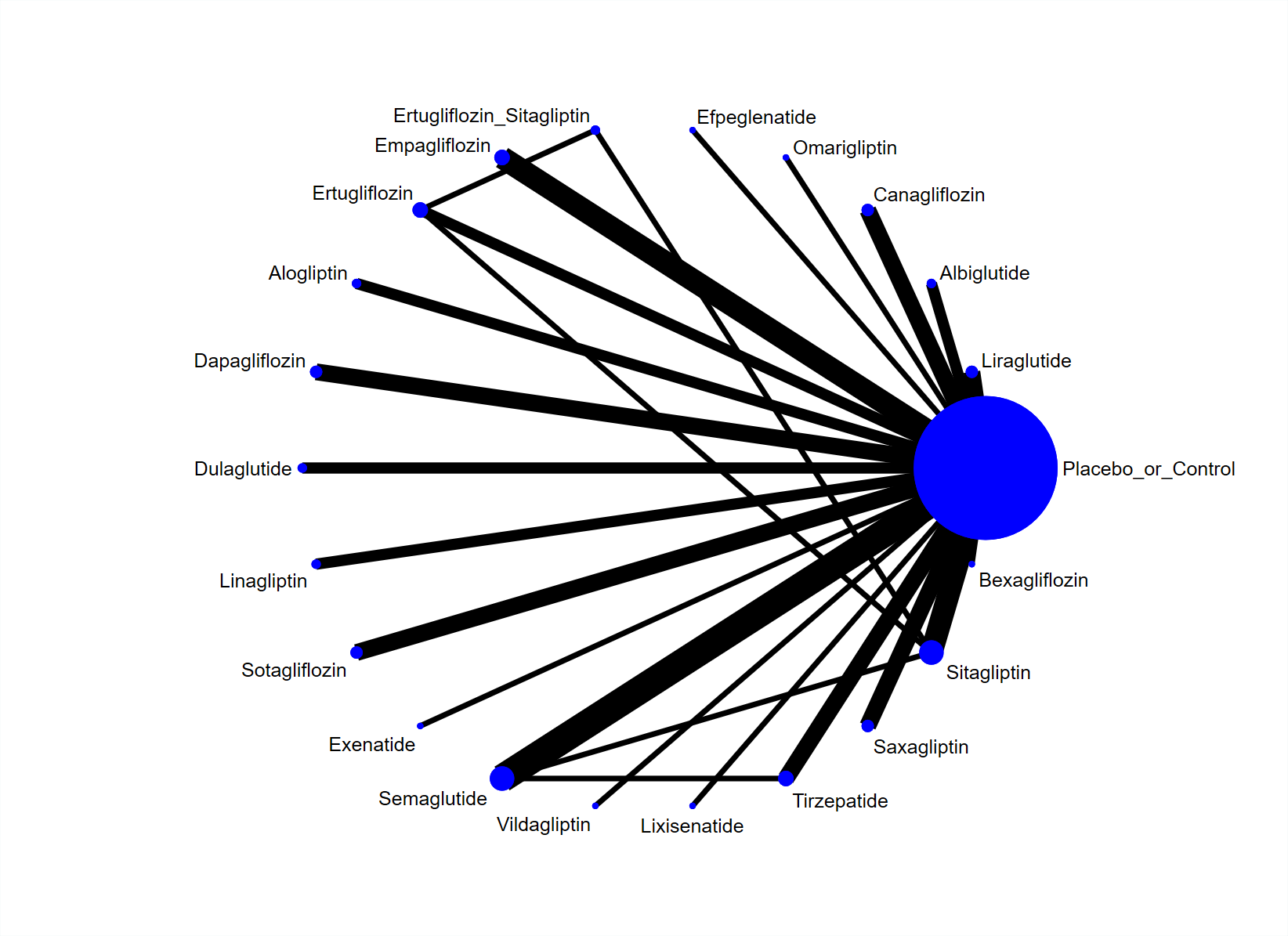
**

**eFigure 1N Network structure of the primary outcome: non-Hodgkin's lymphoma**

**
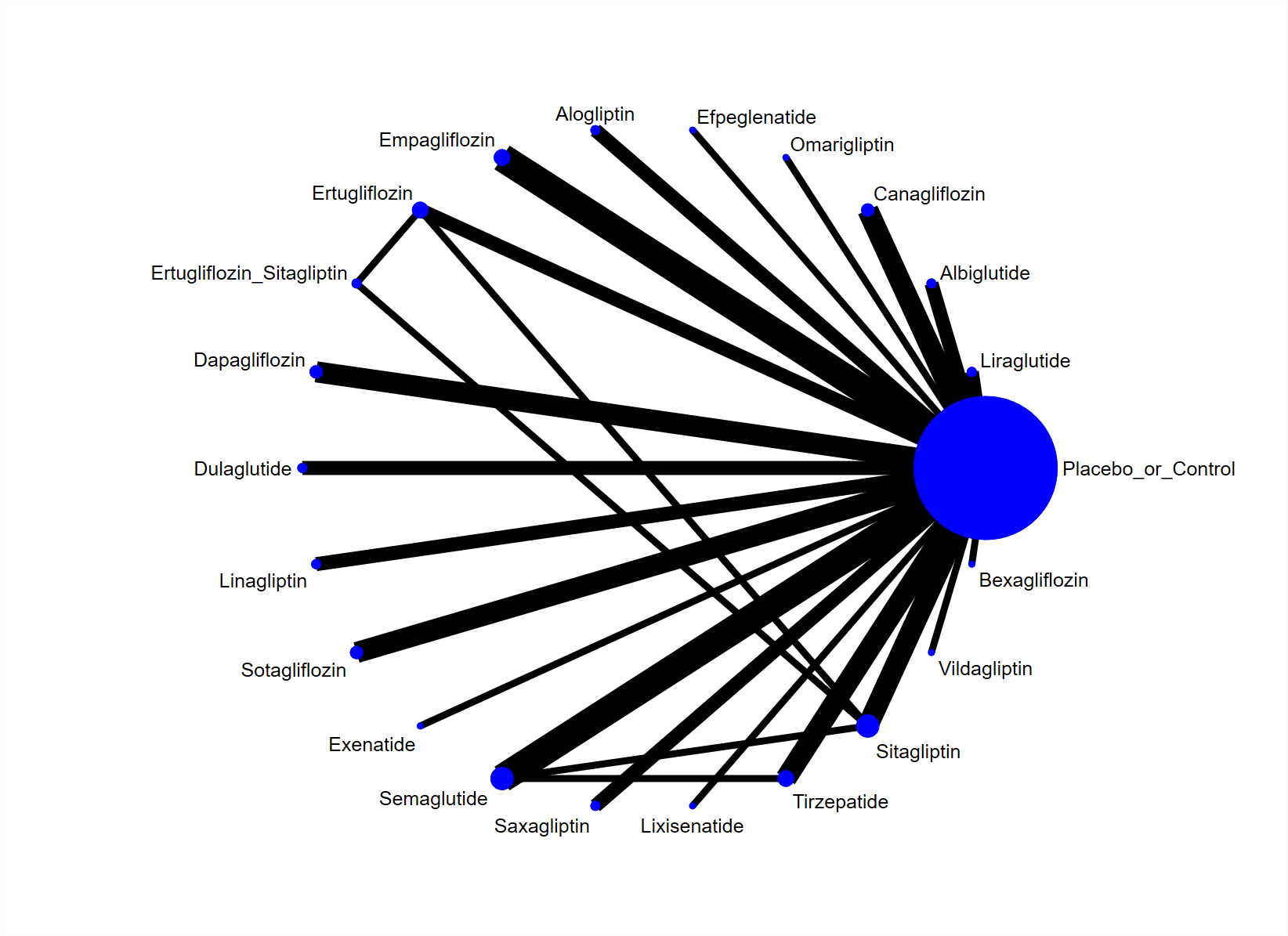
**

**eFigure 1O Network structure of the primary outcome: B cell non-Hodgkin's lymphoma**

**
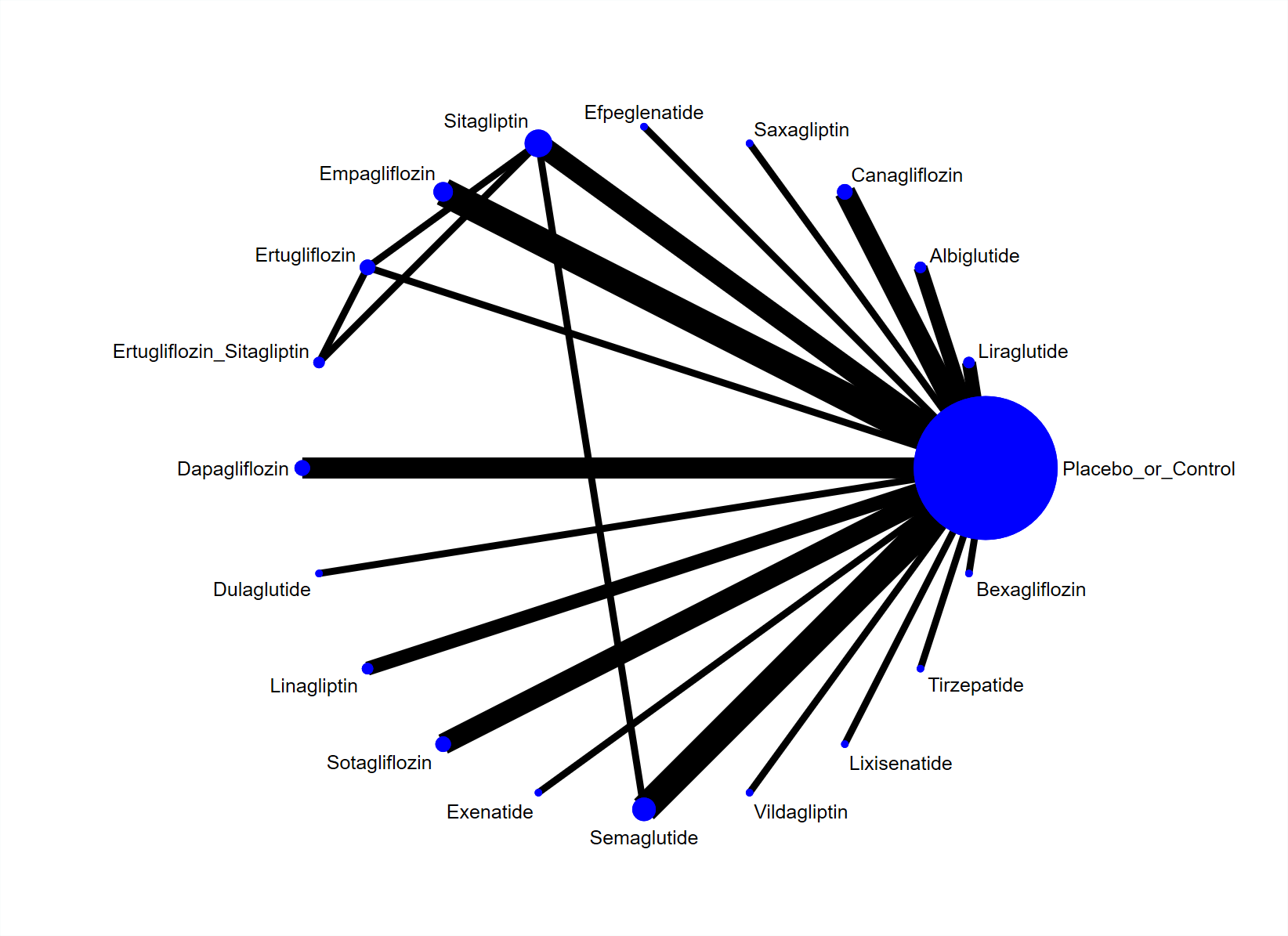
**

**eFigure 1P Network structure of the primary outcome: T cell non-Hodgkin's lymphoma**

**
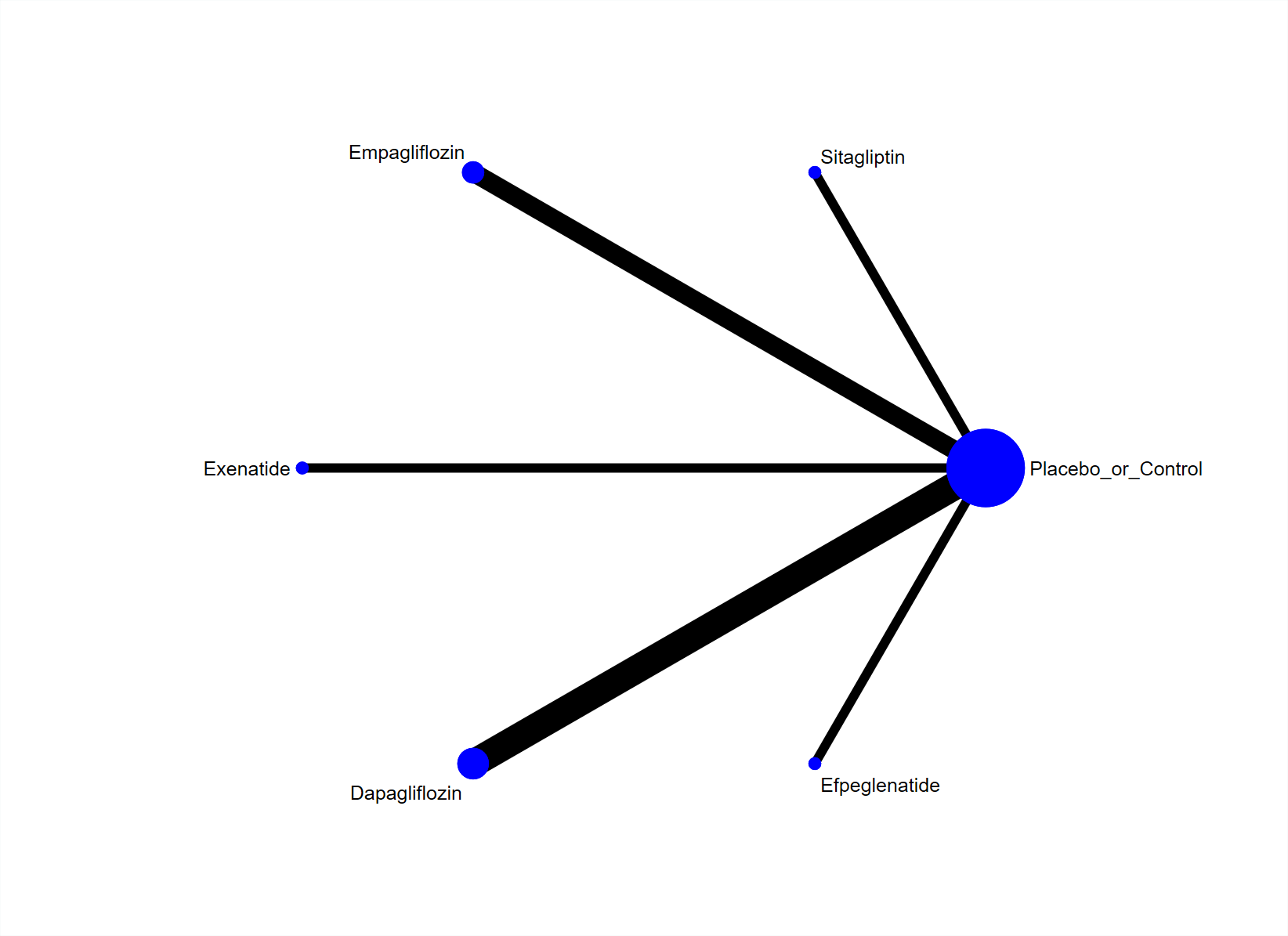
**

**eFigure 1Q Network structure of the primary outcome: myeloma**

**
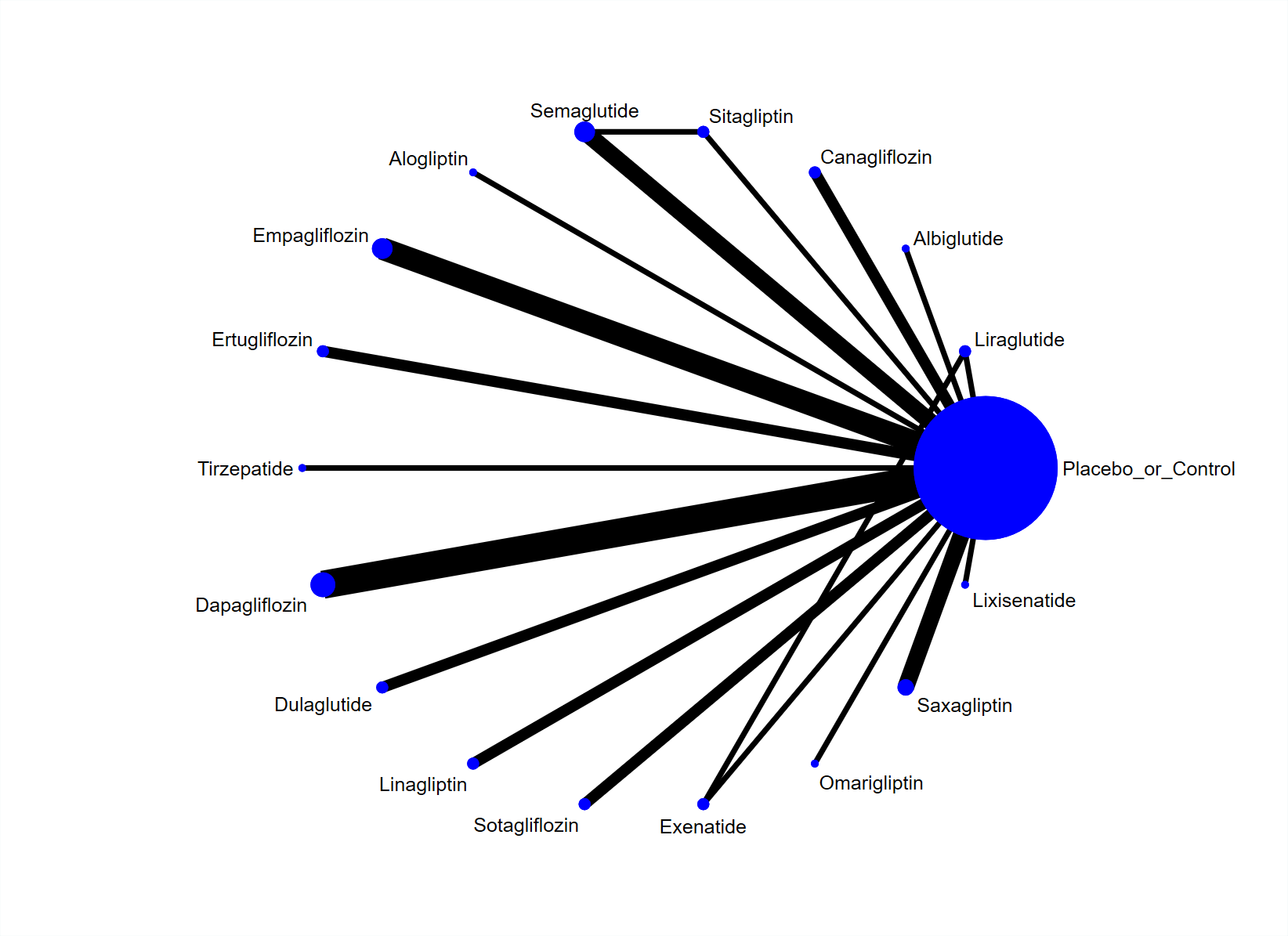
**

**eFigure 1R Network structure of the primary outcome: plasma cell myeloma**

**
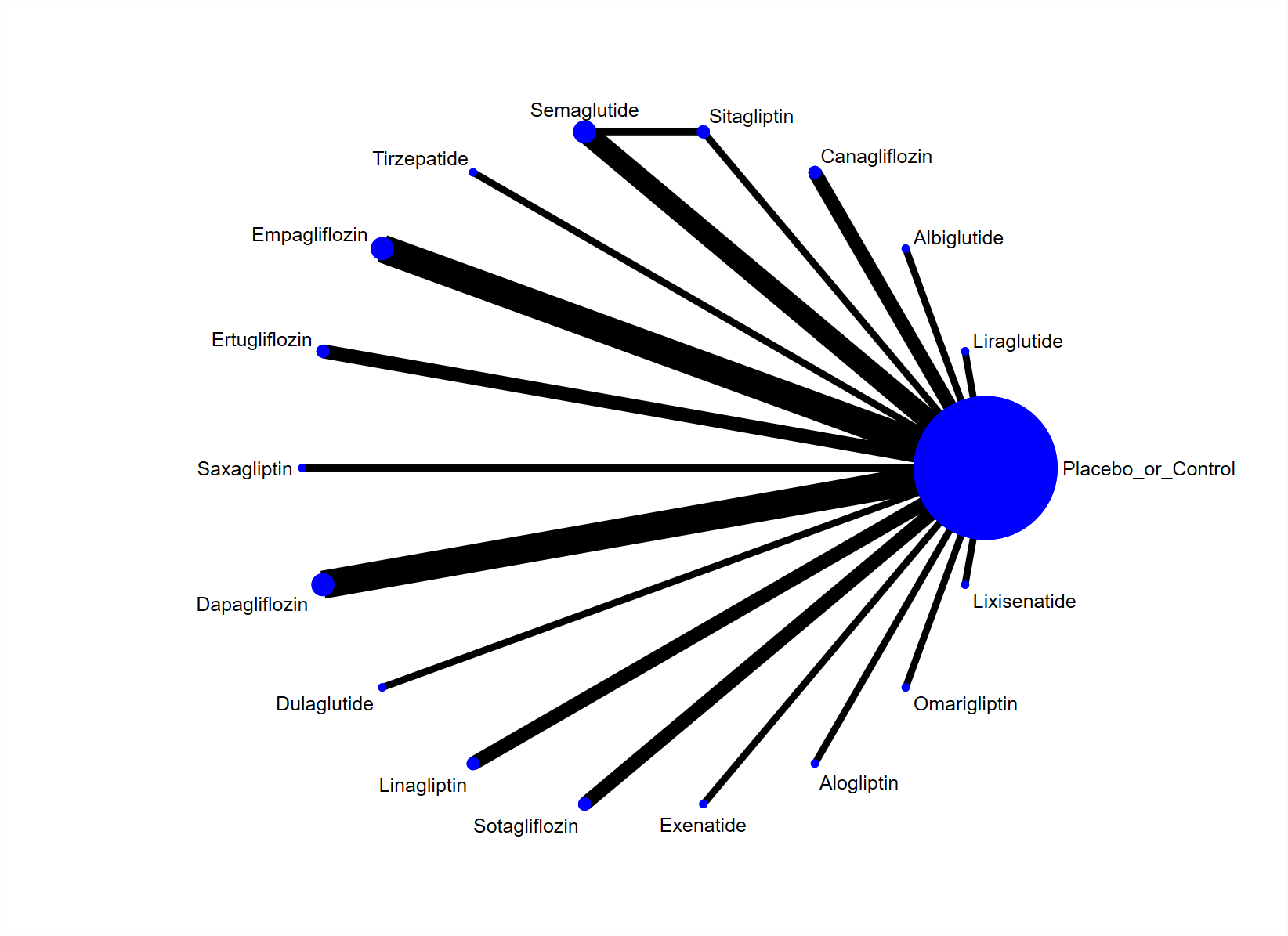
**

**eFigure 1S Network structure of NMA of the acceptability: drop-out rate**

**
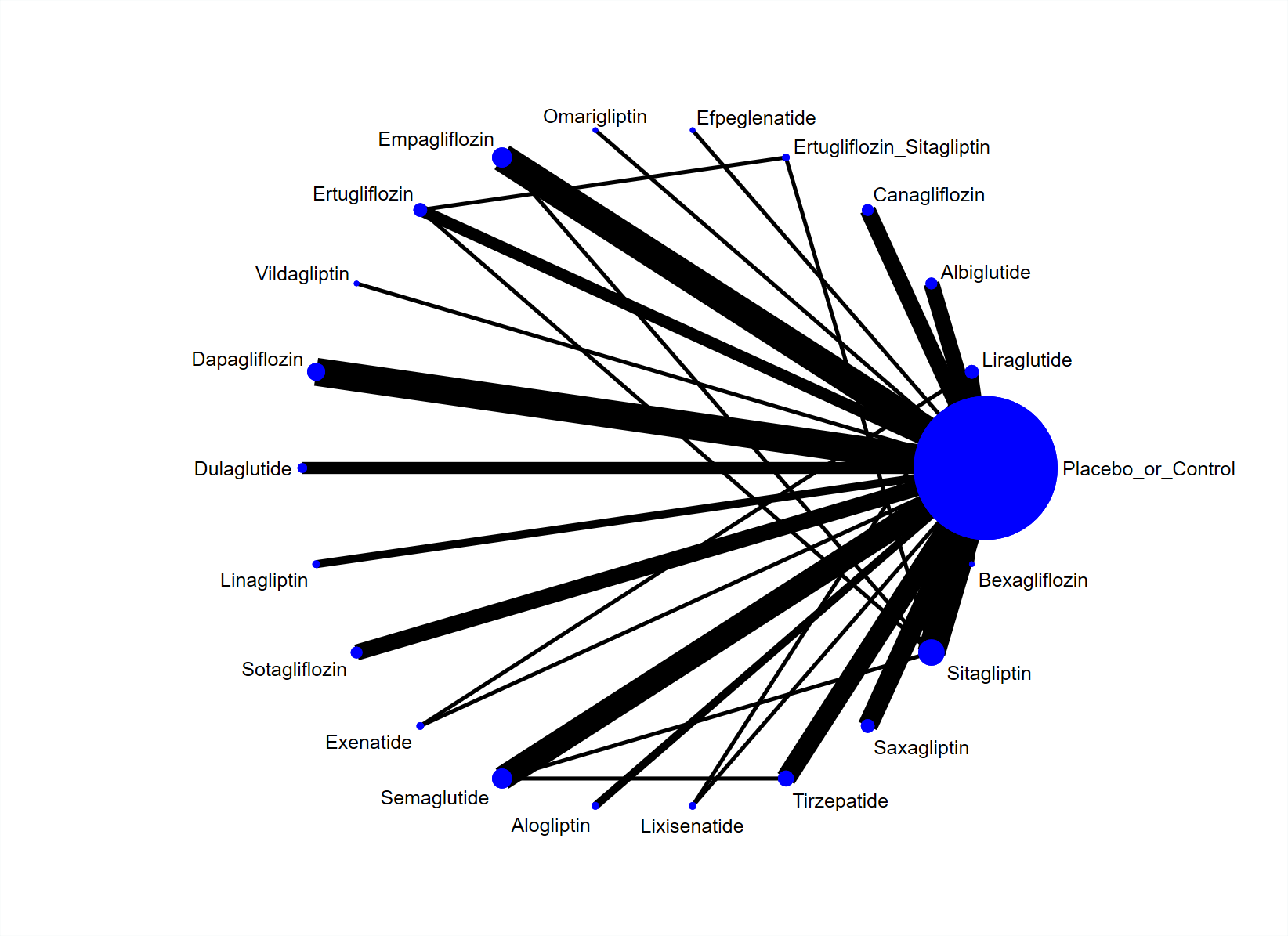
**

**Figure legend of eFigure 1A-1S**

Overall structure of the network meta-analysis. The lines between nodes represent direct comparisons in various trials, and the size of each circle is proportional to the number of participants in each specific treatment. The thickness of the lines is proportional to the number of trials connected to the network.

***Abbreviation for eFigure 1A-1S:***

*95%CIs: 95% confidence intervals; DPP4 inhibitor: dipeptidyl peptidase 4 inhibitor; GLP-1 agonist: glucagon-like peptide-1 agonist; NMA: network meta-analysis; RCT: randomized controlled trial; RR: risk ratio; SGLT2 inhibitor: sodium–glucose cotransporter 2 inhibitor*

**eFigure 2A Forest plot of primary outcome: overall hematologic malignancy**

**
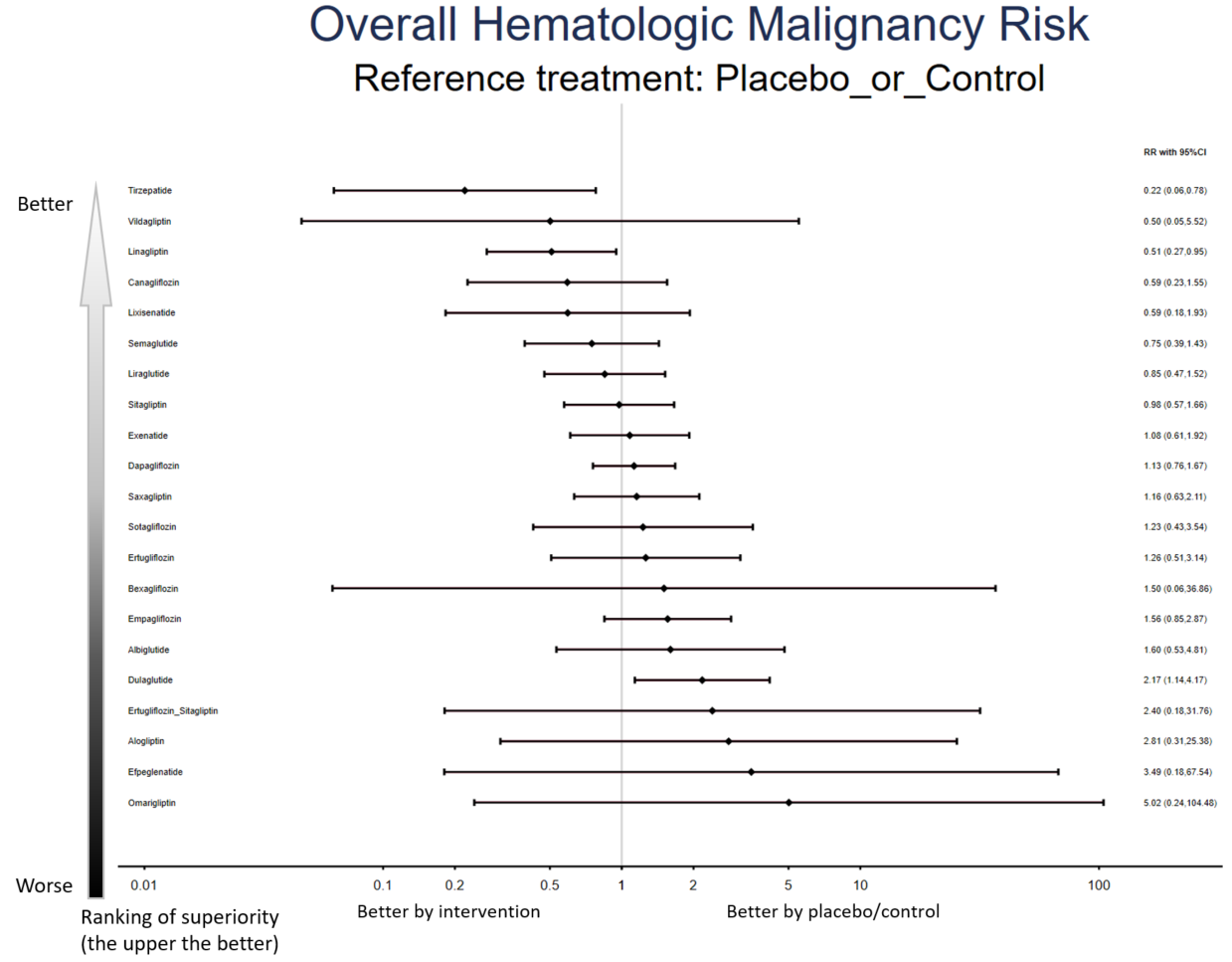
**

**eFigure 2B Forest plot of primary outcome: overall hematologic malignancy risk – Peto odds ratio**

**
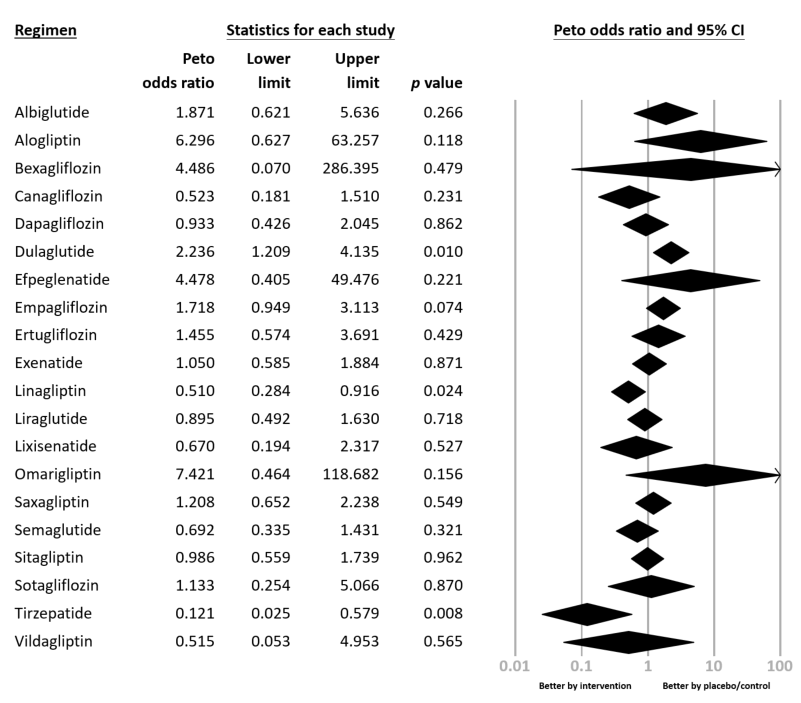
**

**eFigure 2C Forest plot of primary outcome: overall hematologic malignancy risk – hazard ratio based on time-to-event data**

**
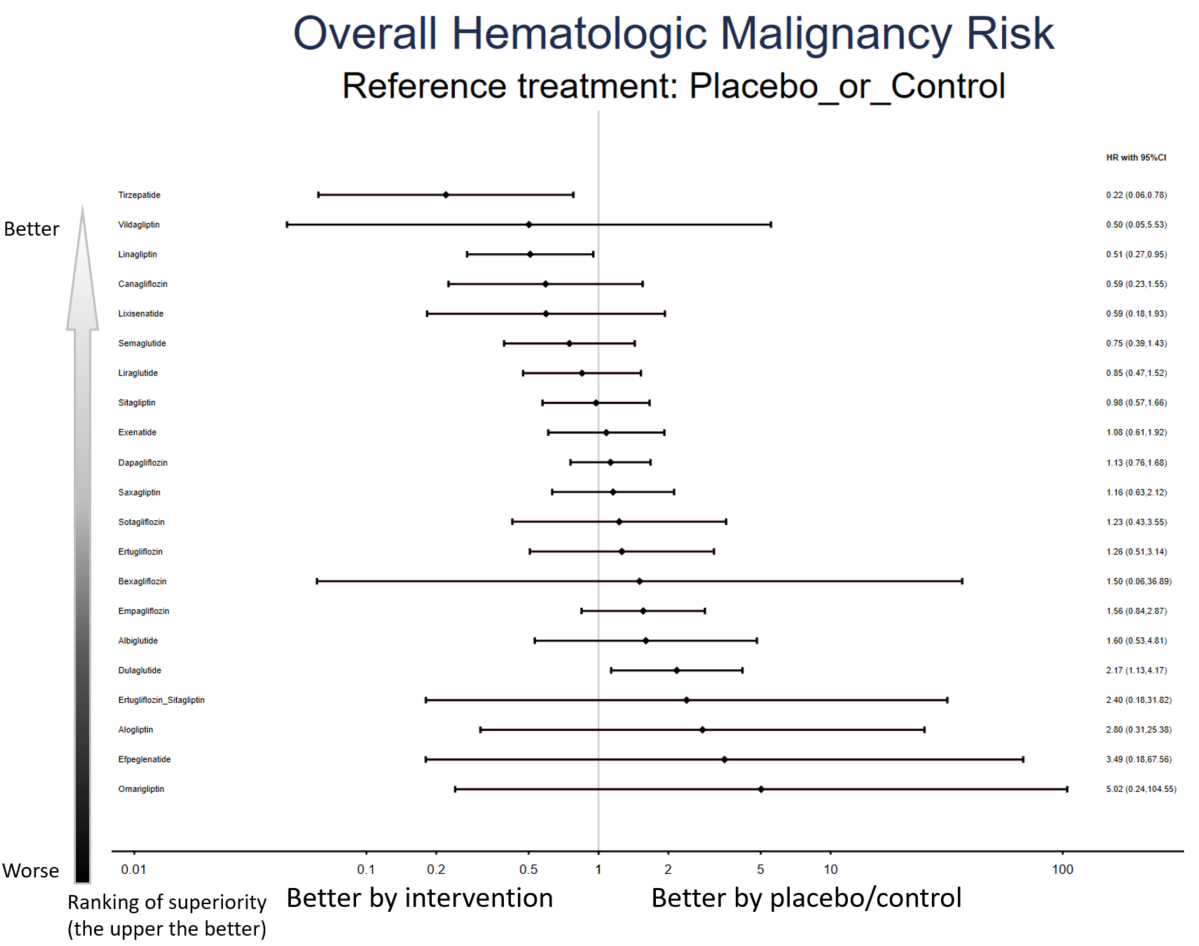
**

**eFigure 2D Forest plot of NMA of the primary outcome: overall hematologic malignancy risk in aspect of various dosage subgroups**

**
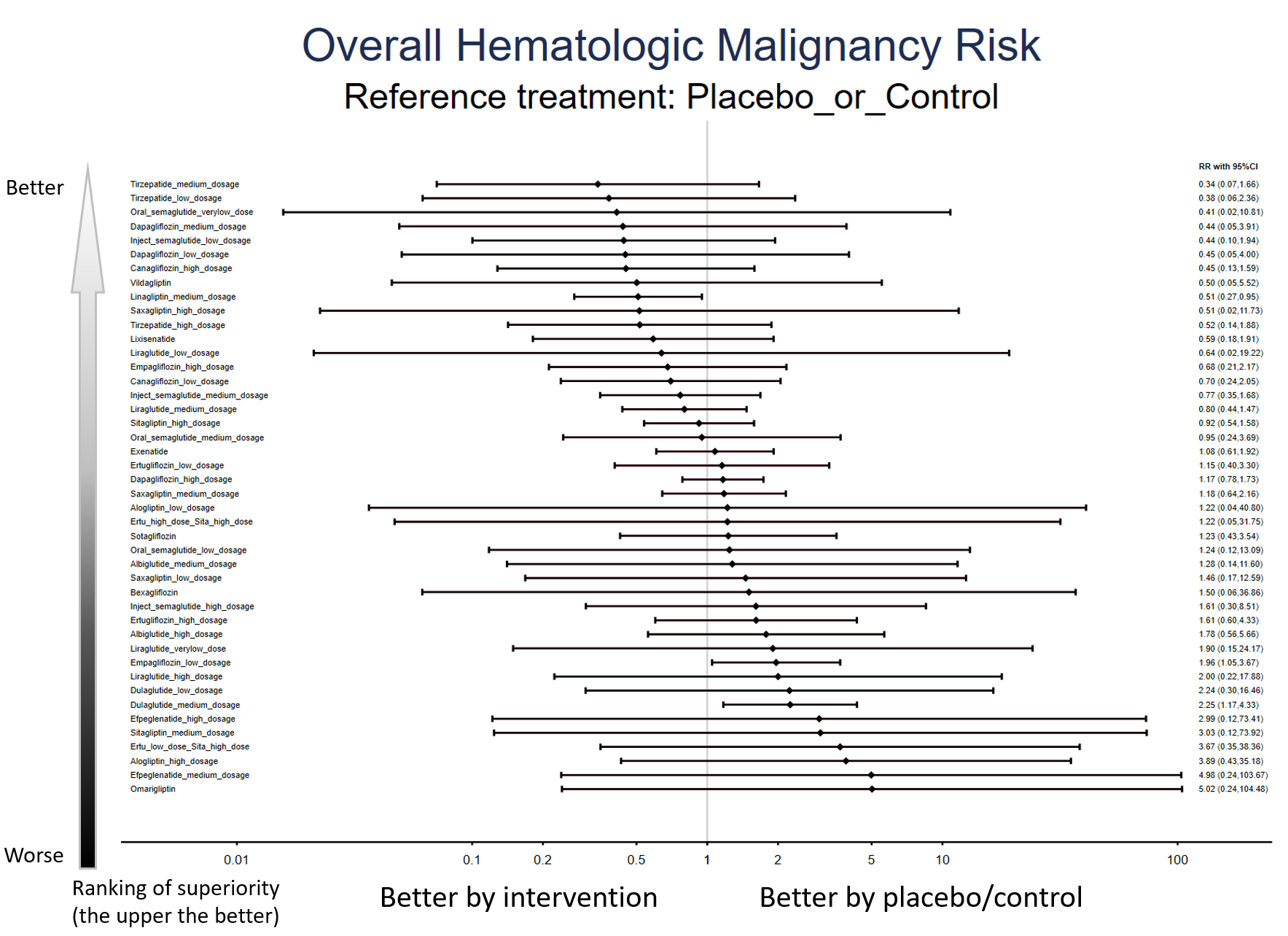
**

**eFigure 2E Forest plot of NMA of the primary outcome: overall hematologic malignancy risk subgroup focusing RCTs with at least 1 year treatment duration**

**
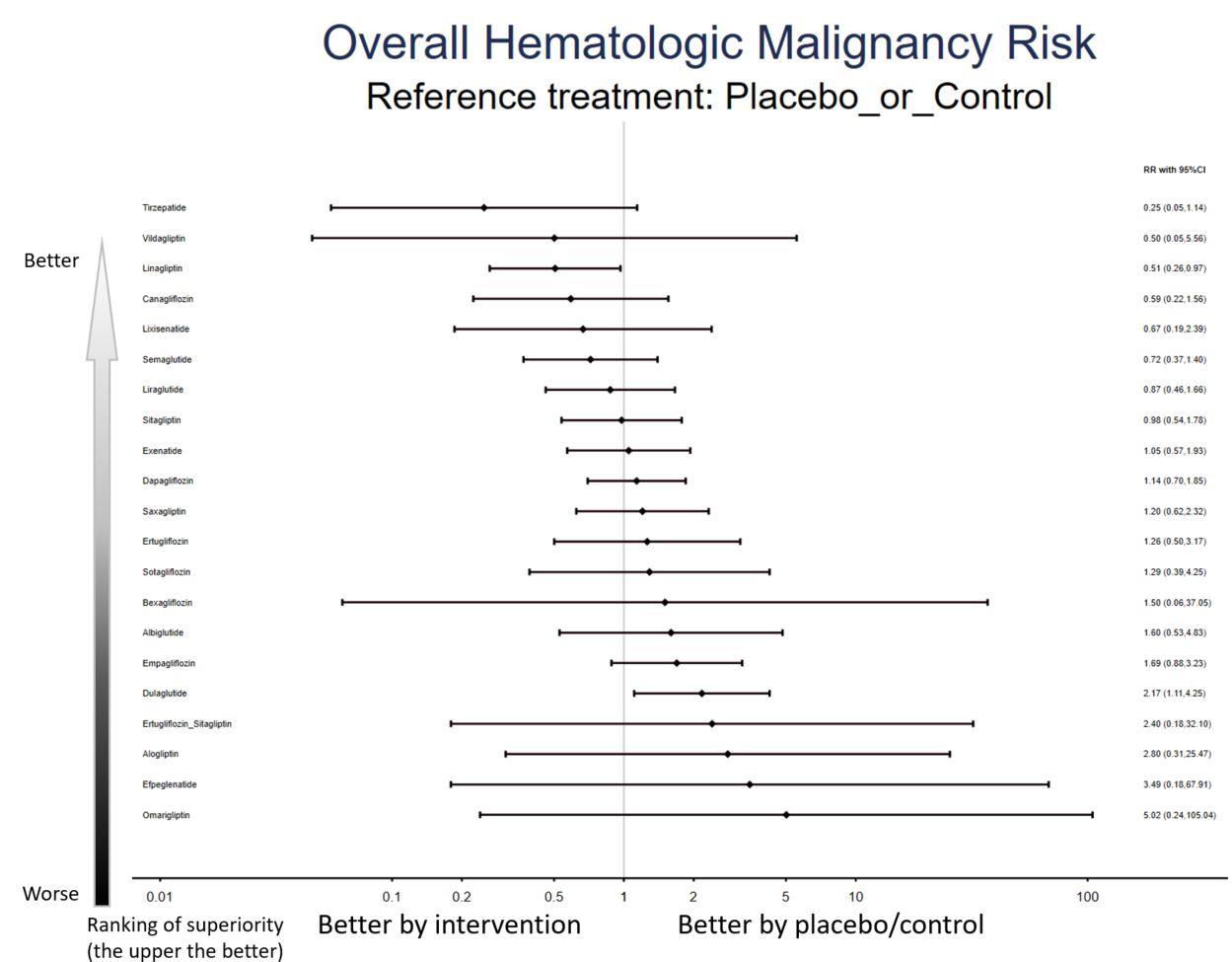
**

**eFigure 2F Forest plot of NMA of the primary outcome: overall hematologic malignancy risk subgroup excluding RCTs with high risk of bias**

**
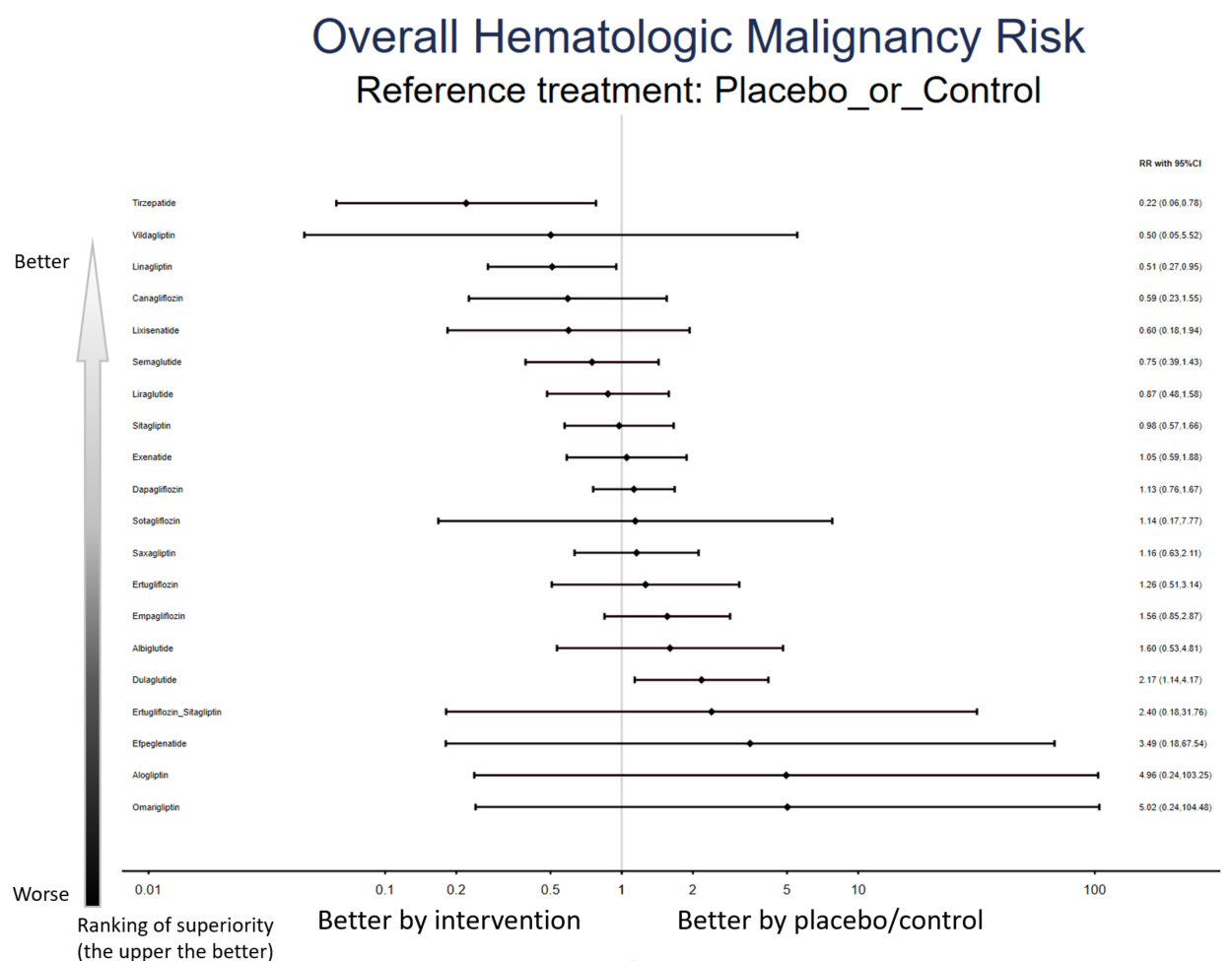
**

**eFigure 2G Forest plot of NMA of the primary outcome: overall hematologic malignancy risk subgroup focusing subjects with diabetes mellitus**

**
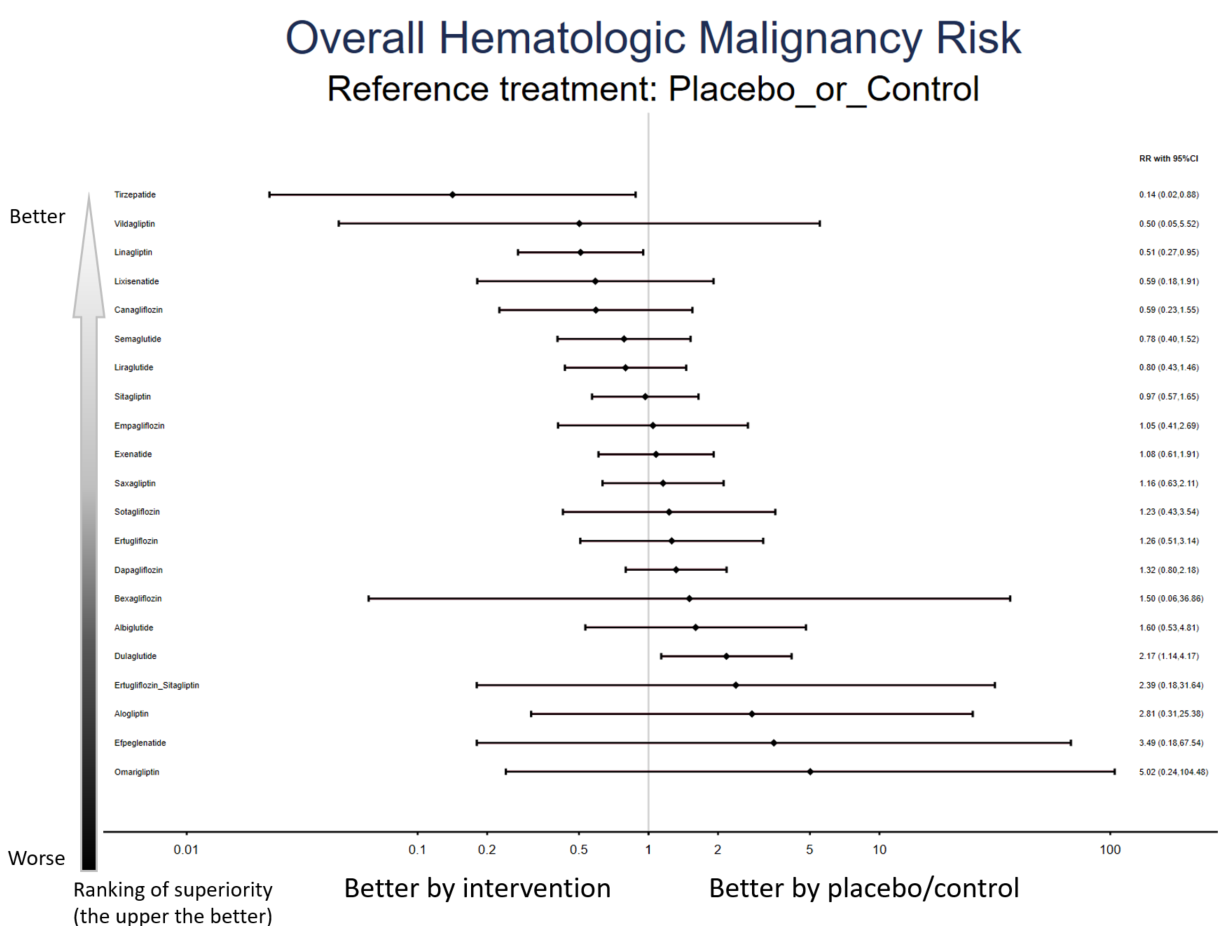
**

**eFigure 2H Forest plot of NMA of the primary outcome: overall hematologic malignancy risk subgroup focusing subjects with age of at least 60 years old**

**
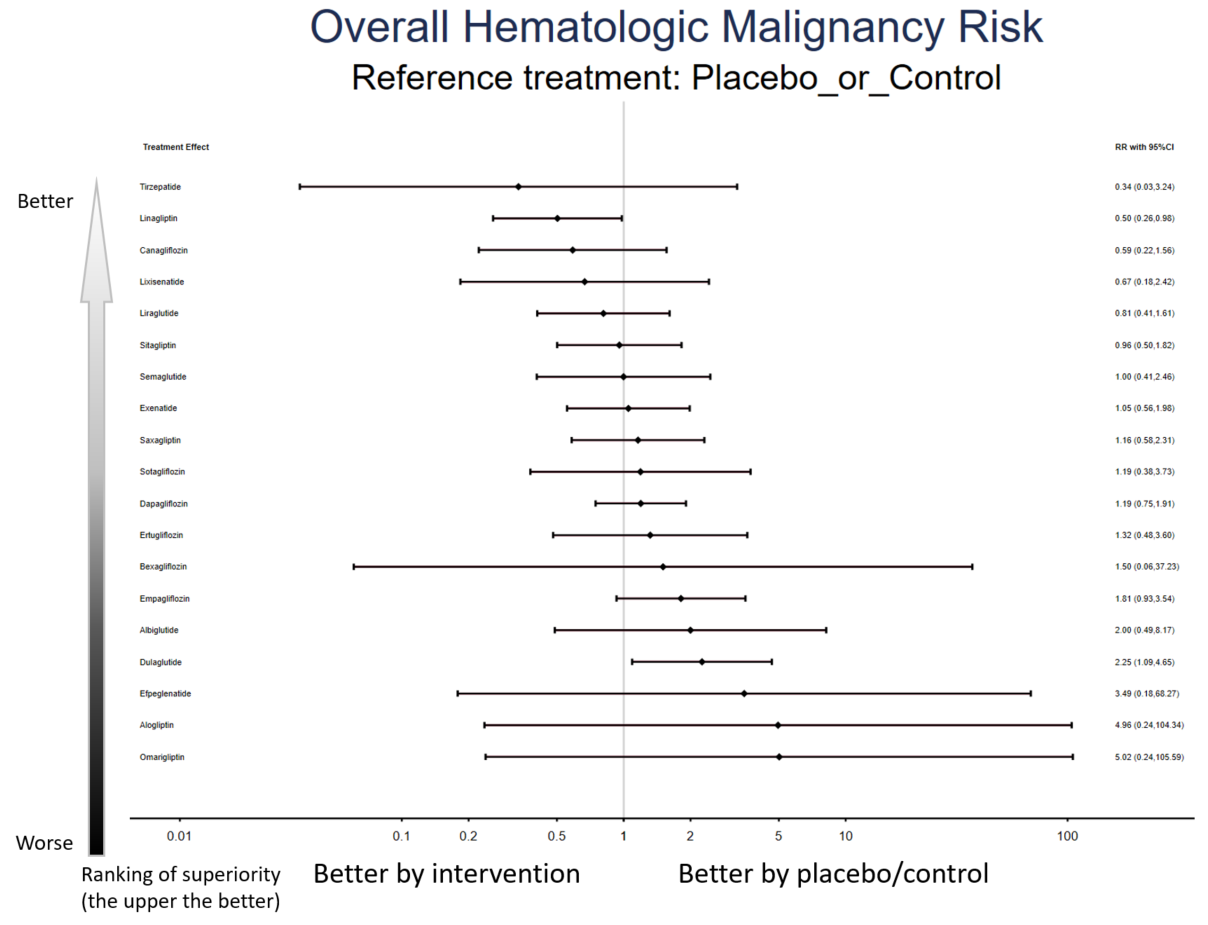
**

**eFigure 2I Forest plot of NMA of the primary outcome: overall hematologic malignancy risk subgroup focusing subjects with age younger than 60 years old**

**
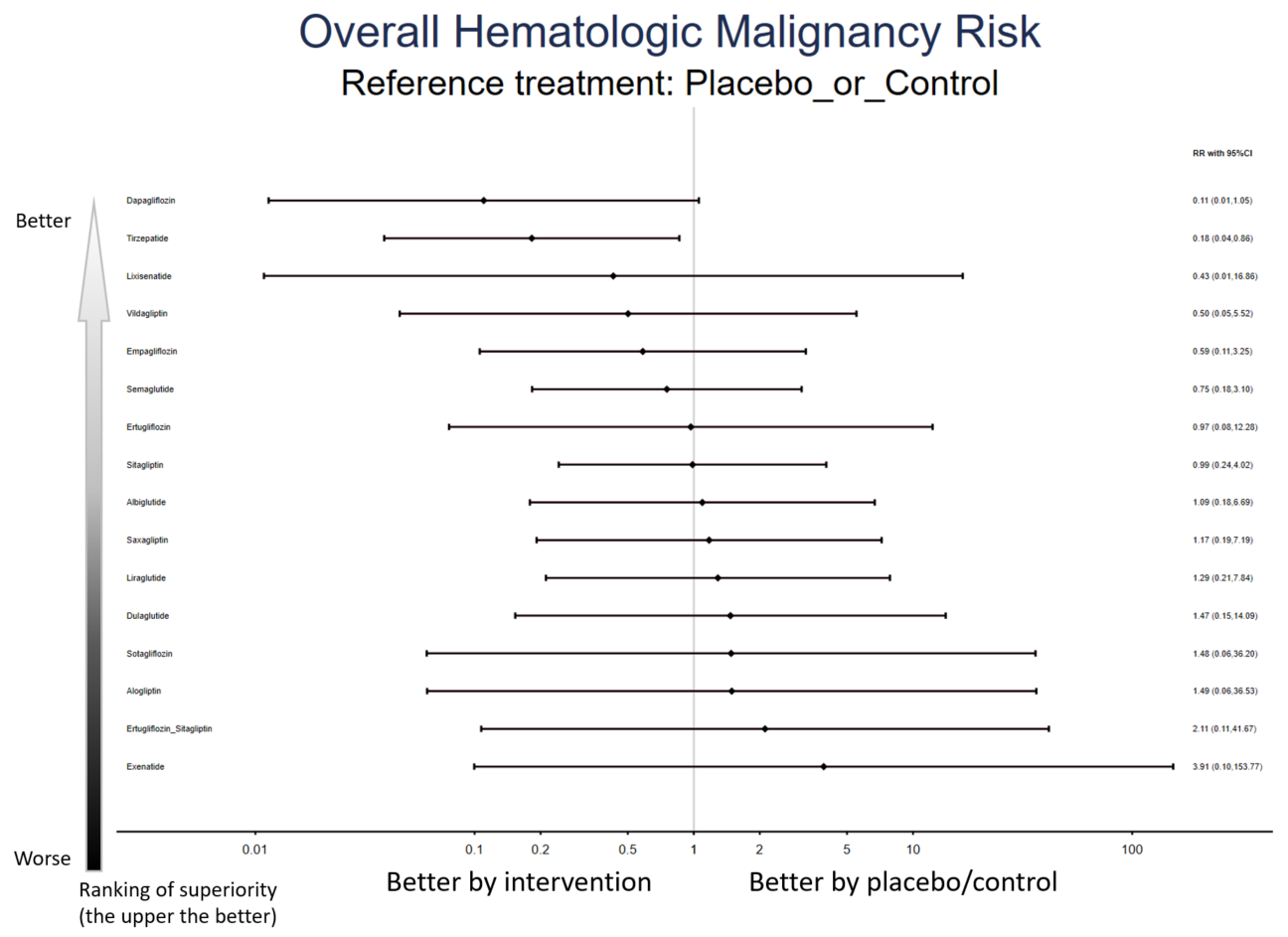
**

**eFigure 2J Forest plot of primary outcome: leukemia**

**
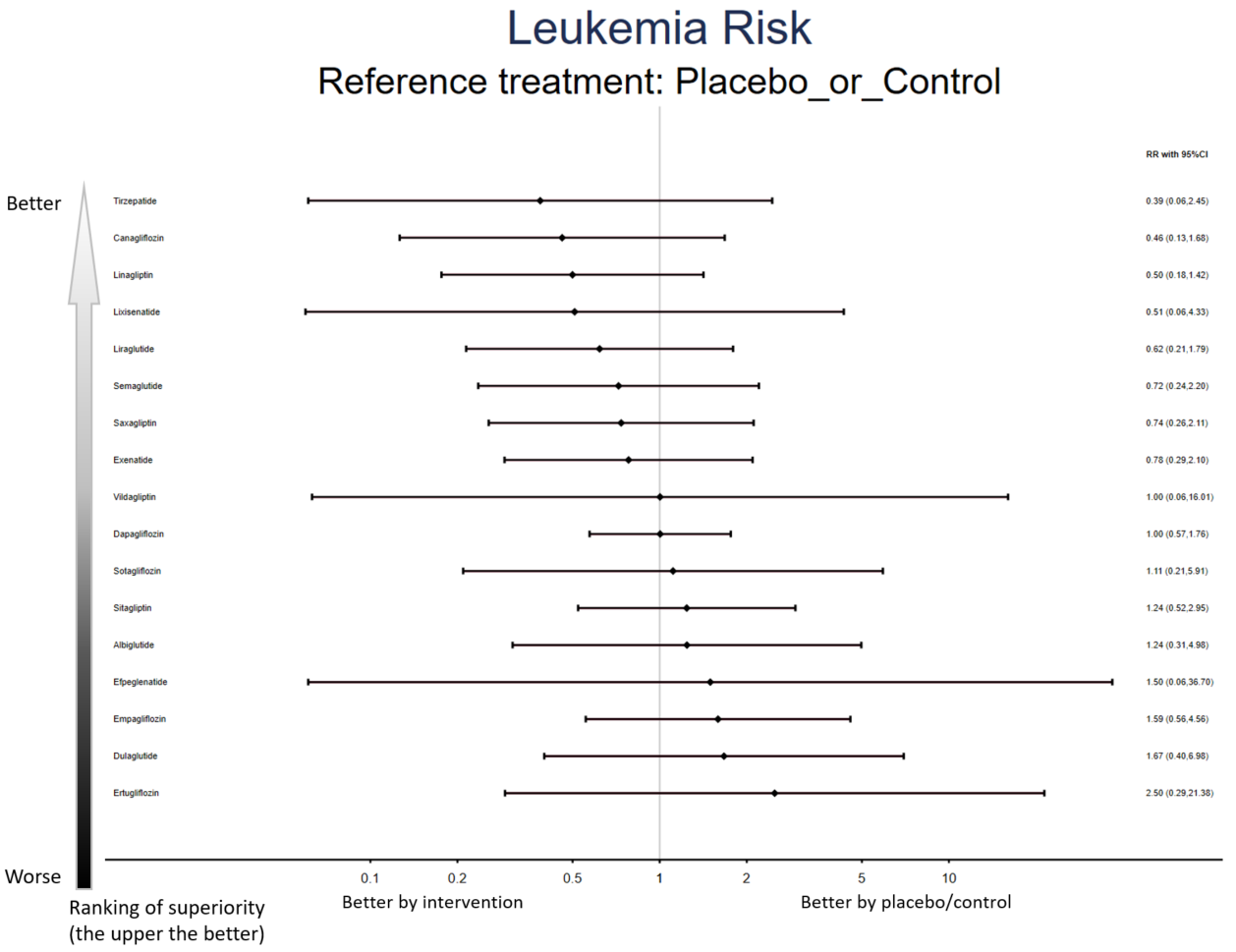
**

**eFigure 2K Forest plot of primary outcome: acute lymphocytic leukemia**

**
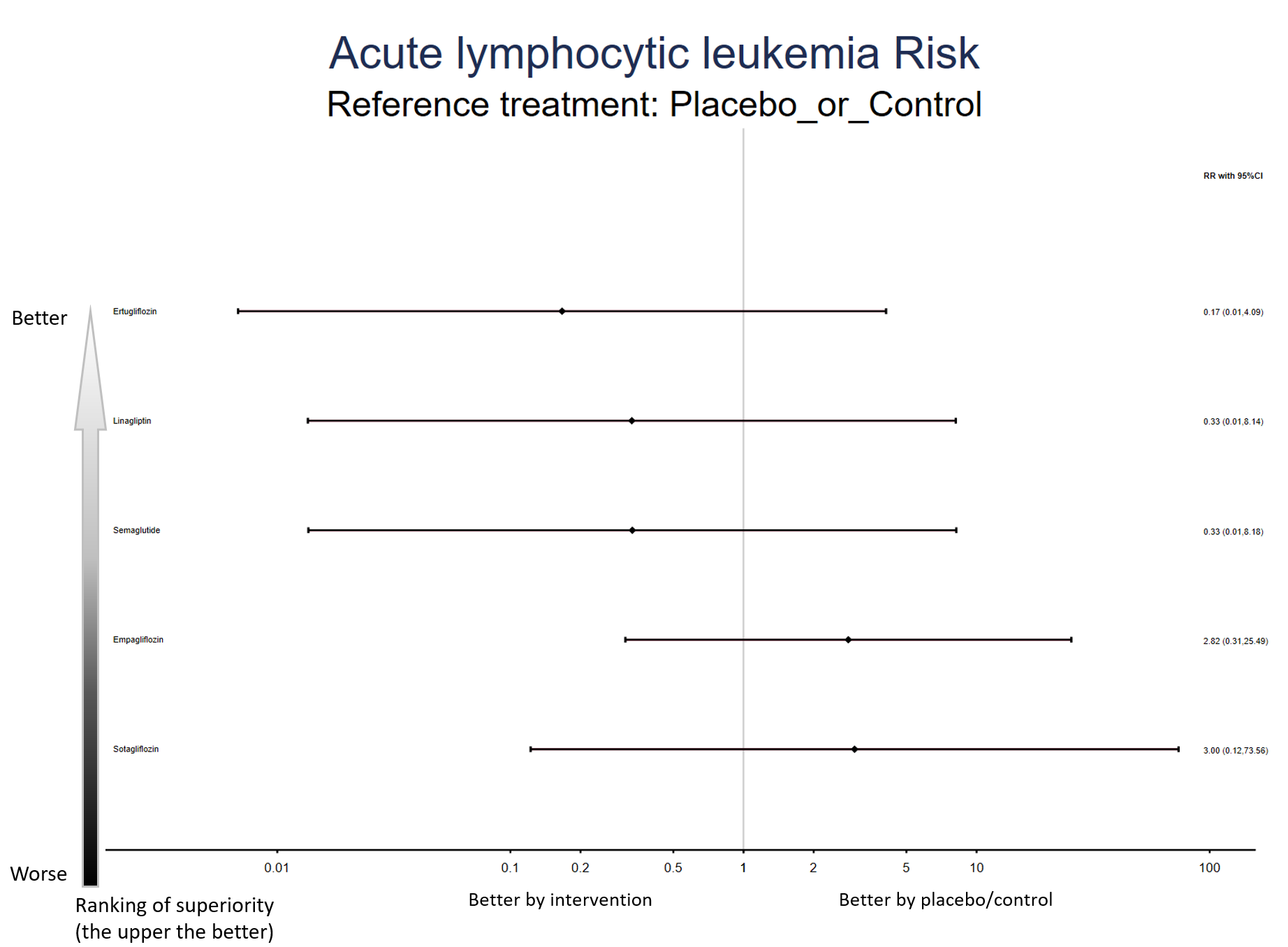
**

**eFigure 2L Forest plot of primary outcome: acute myeloid leukemia**

**
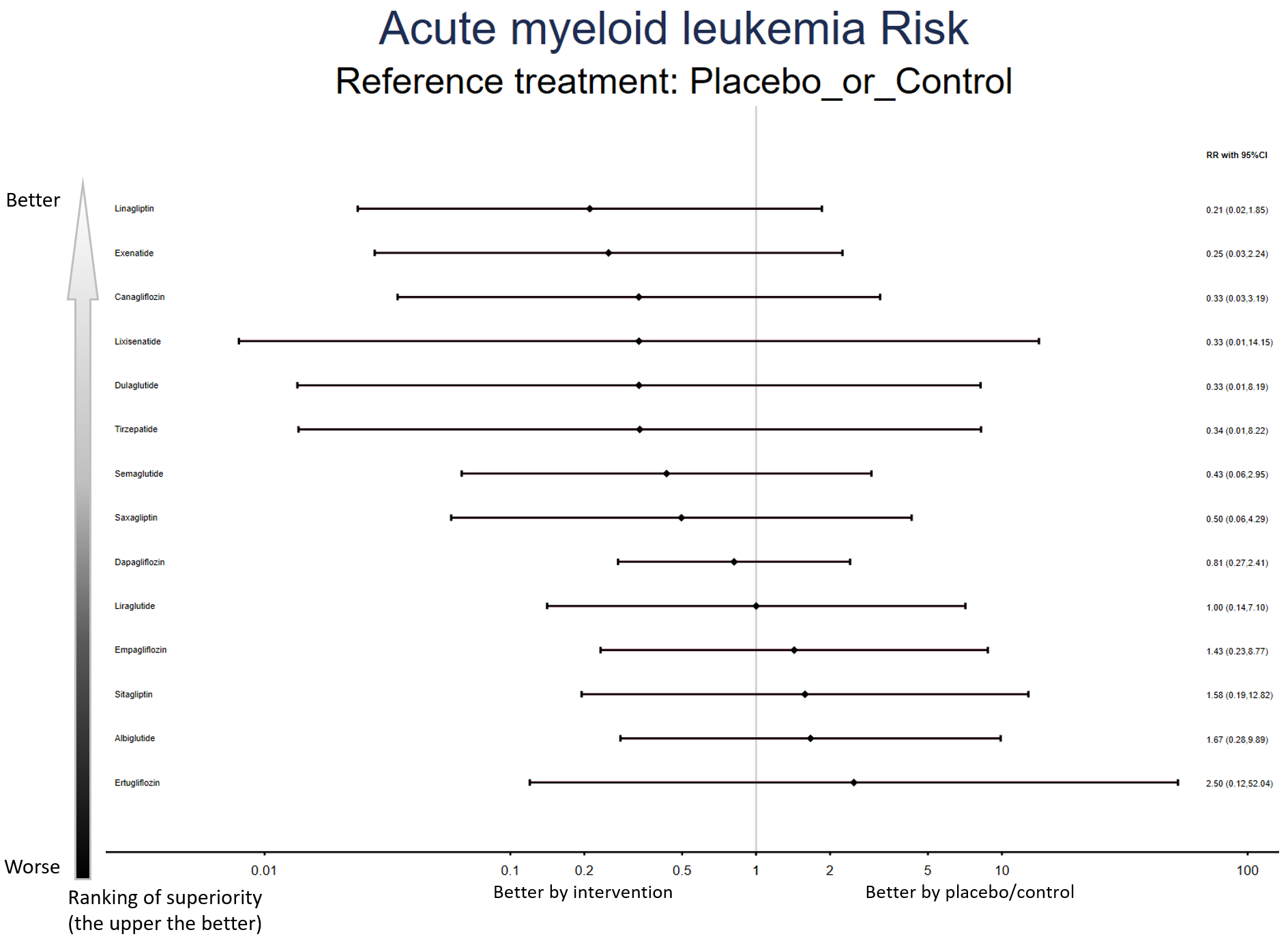
**

**eFigure 2M Forest plot of primary outcome: chronic lymphocytic leukemia**

**
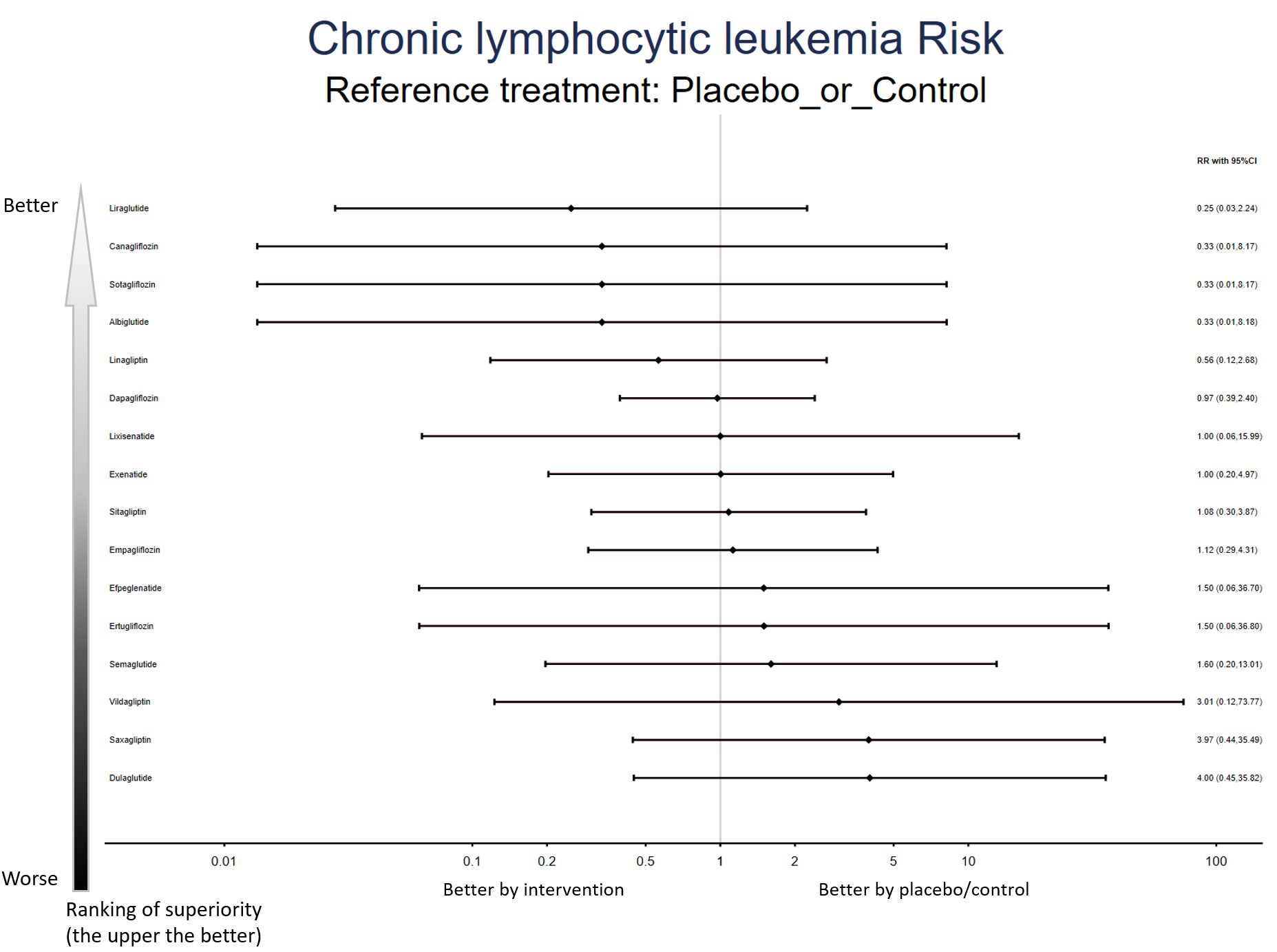
**

**eFigure 2N Forest plot of primary outcome: chronic myeloid leukemia**

**
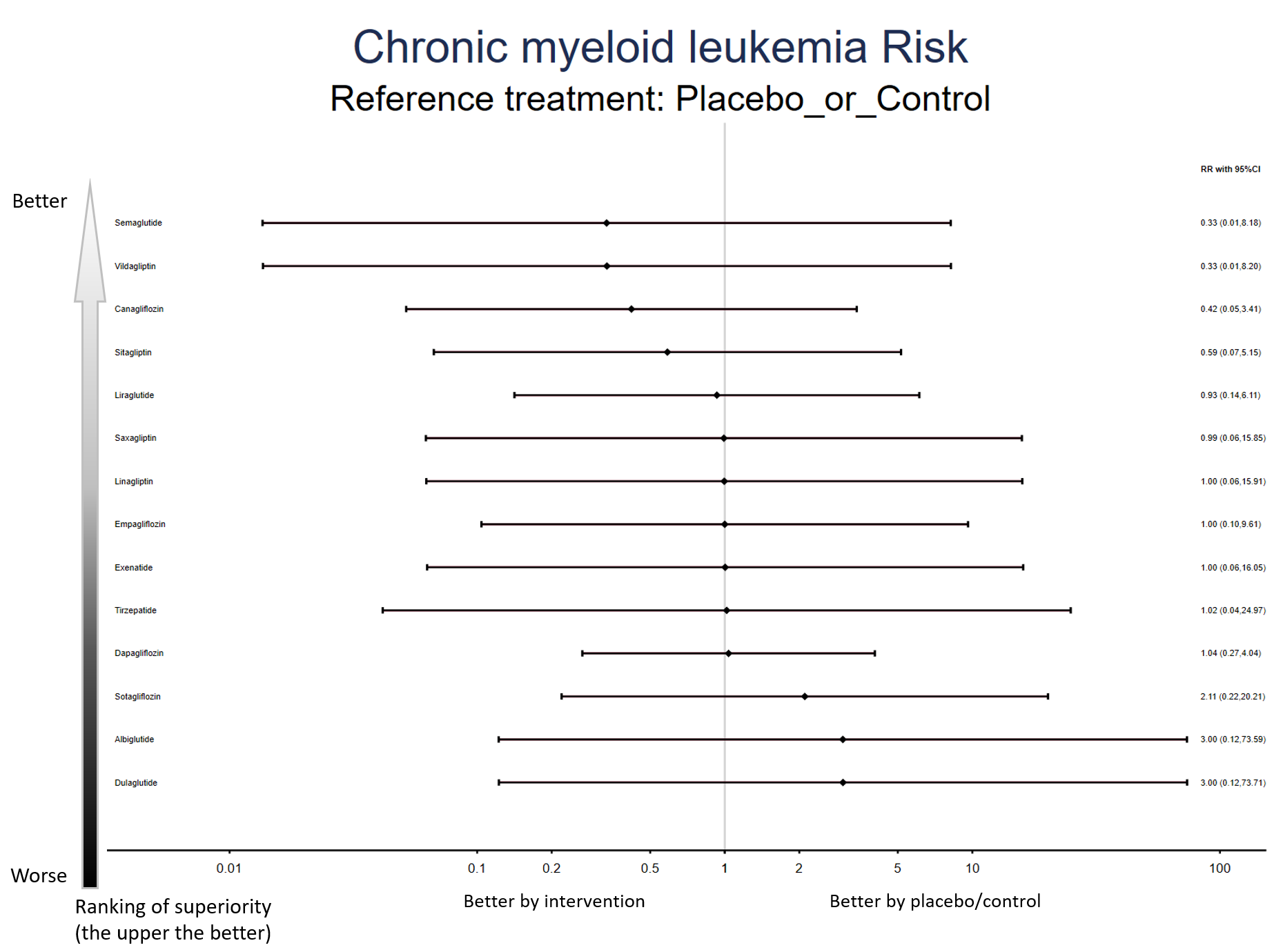
**

**eFigure 2O Forest plot of primary outcome: lymphoma**

**
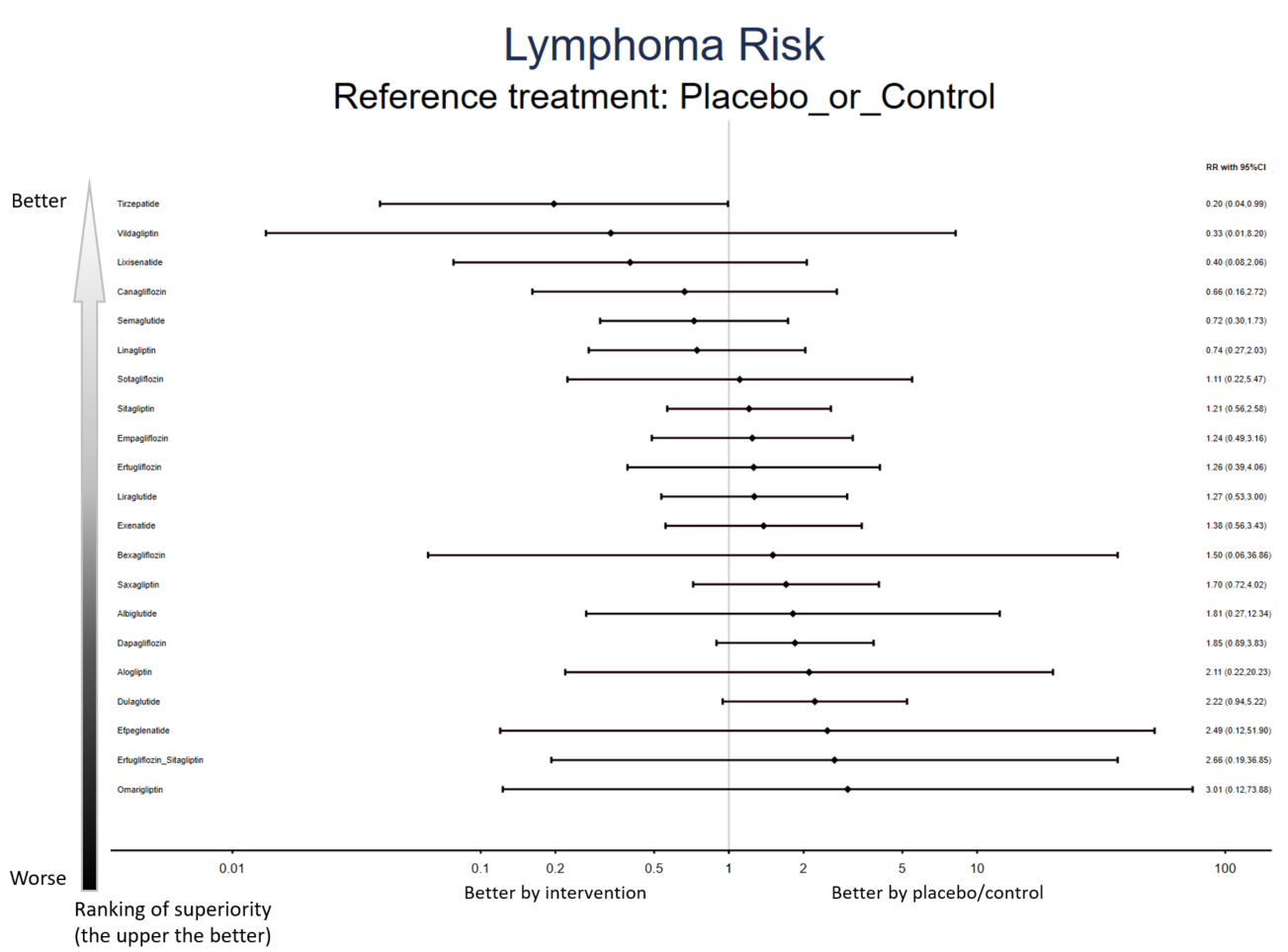
**

**eFigure 2P Forest plot of primary outcome: B cell non-Hodgkin's lymphoma**

**
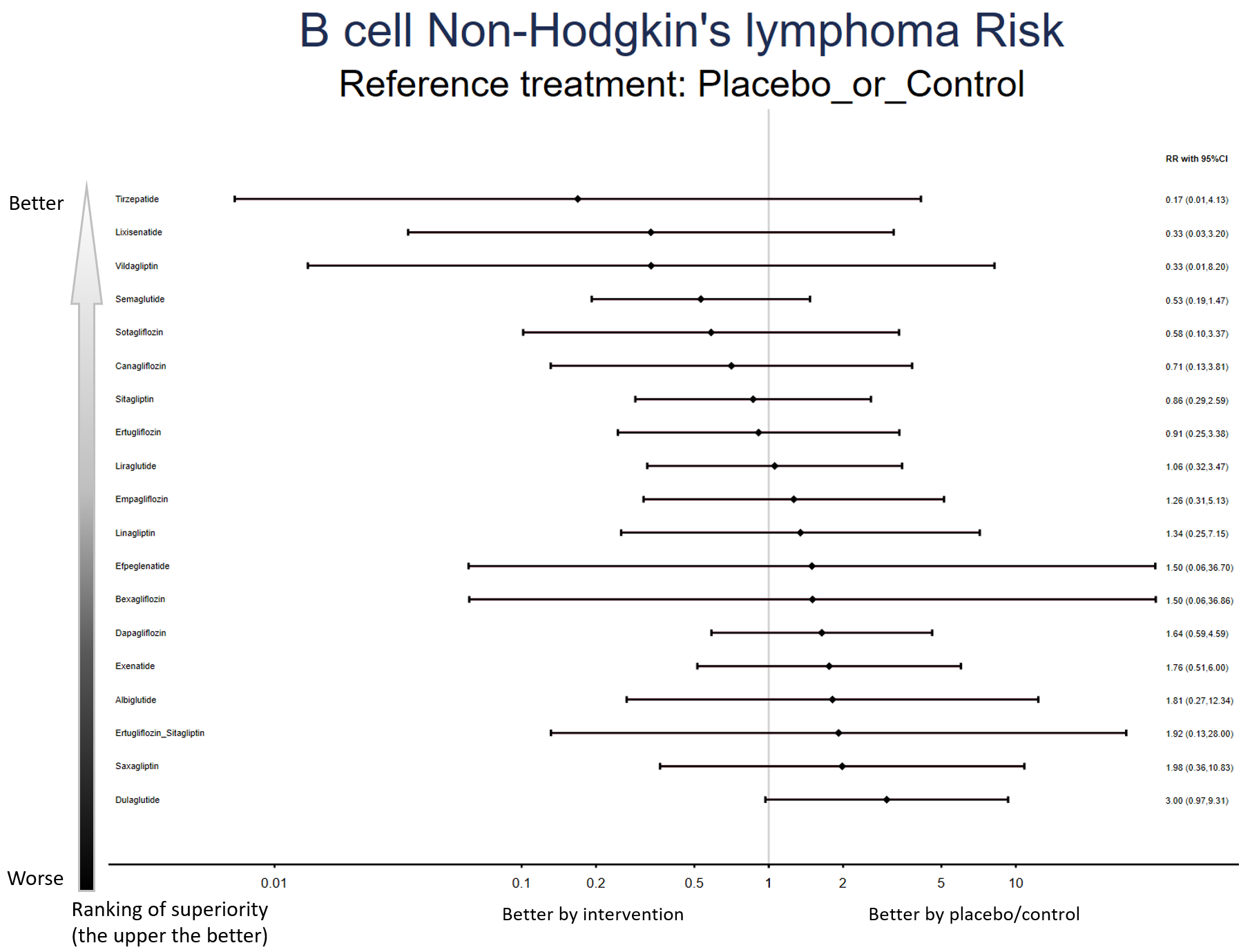
**

**eFigure 2Q Forest plot of primary outcome: T cell non-Hodgkin's lymphoma**

**
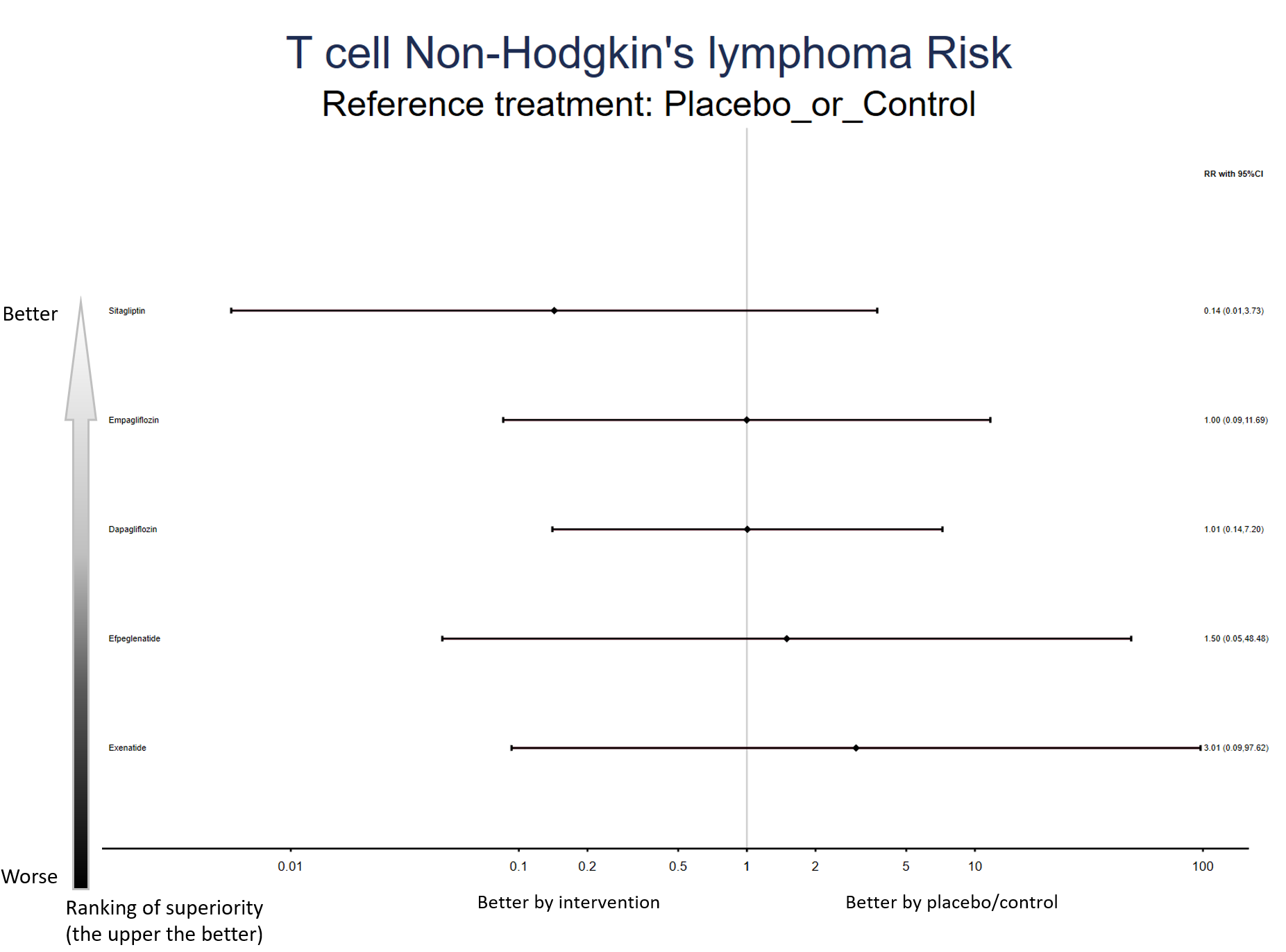
**

**eFigure 2R Forest plot of primary outcome: myeloma**

**
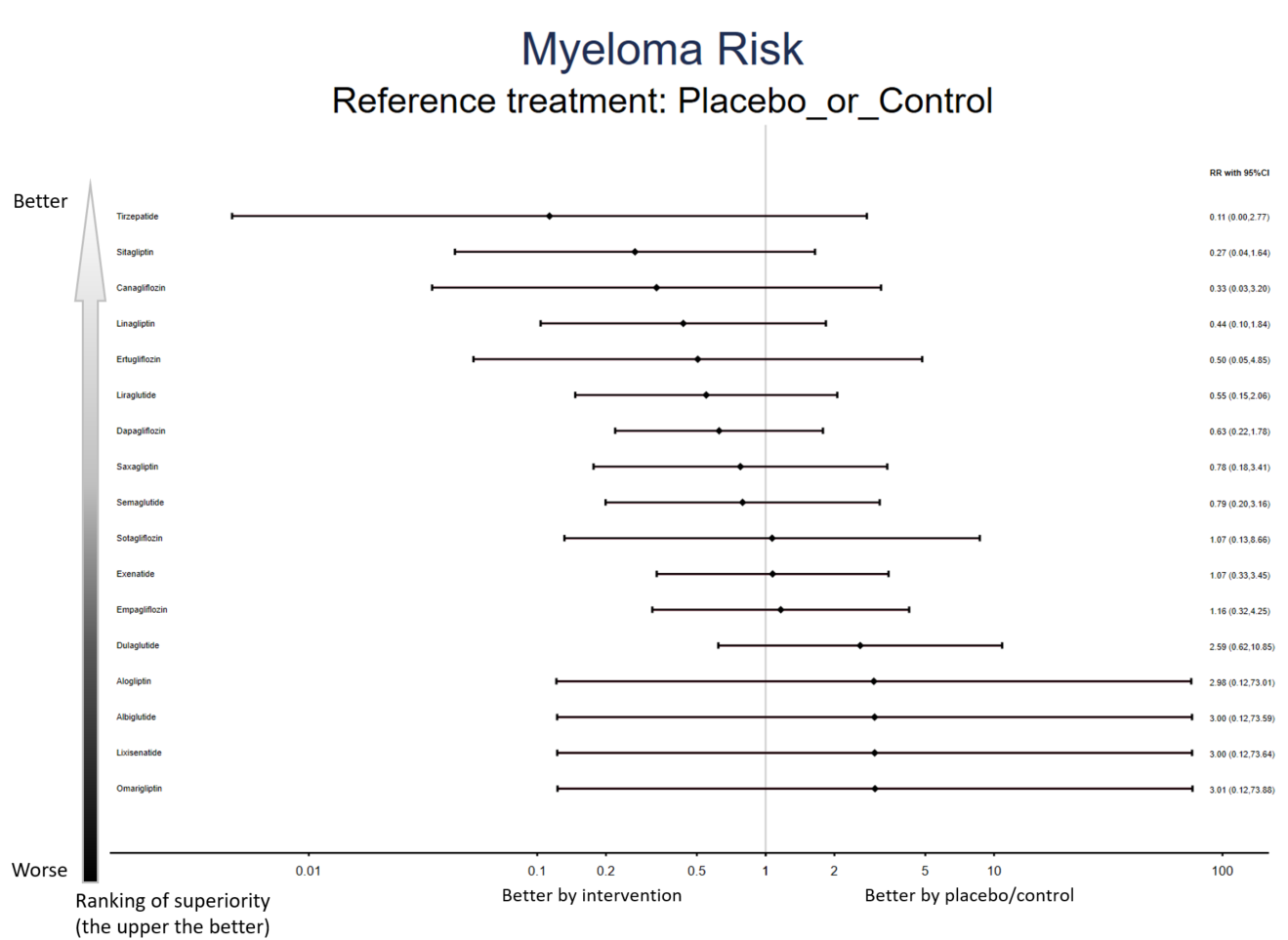
**

**eFigure 2S Forest plot of primary outcome: plasma cell myeloma**

**
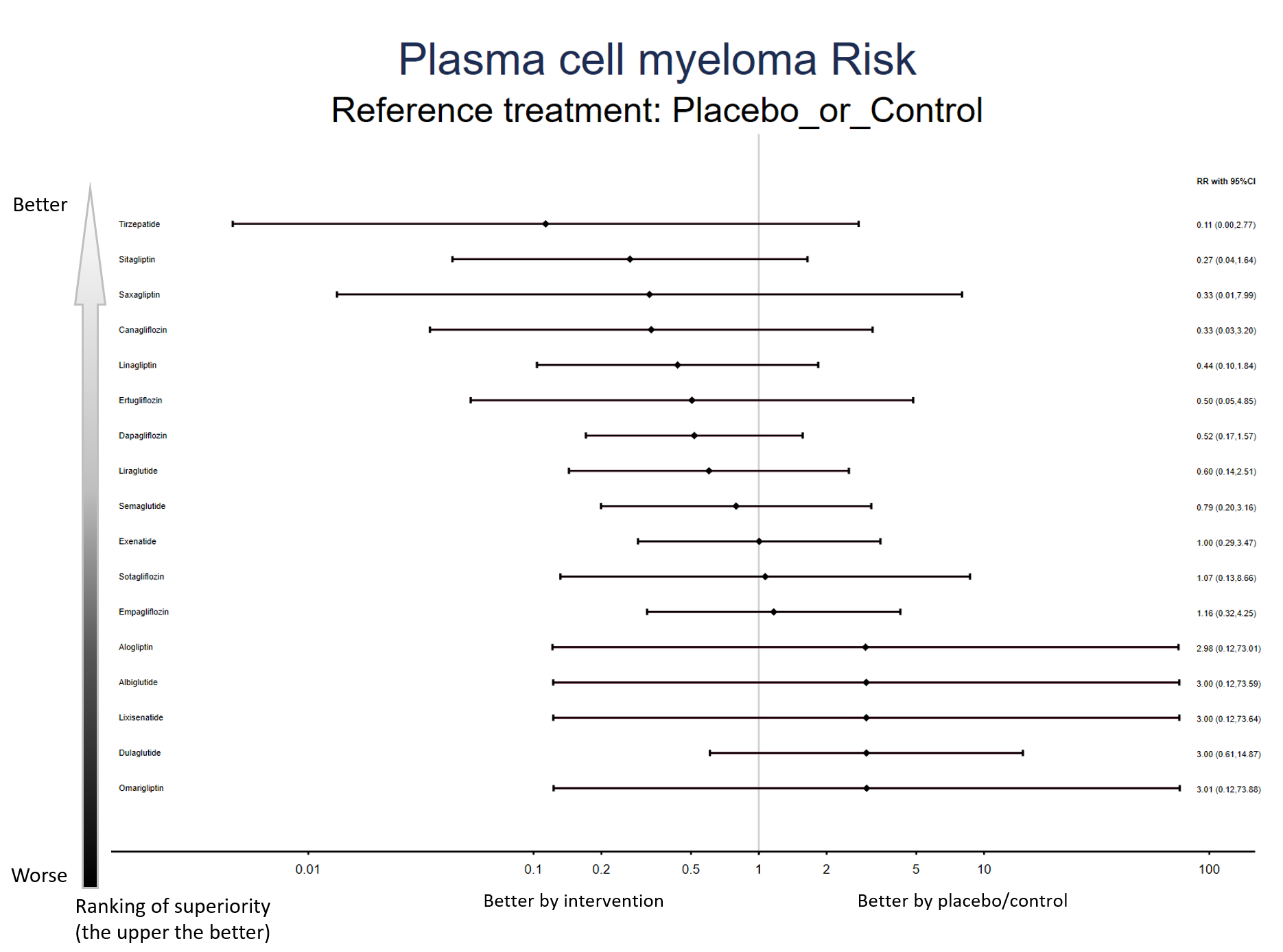
**

**eFigure 2T Forest plot of NMA of the acceptability: drop-out rate**

***
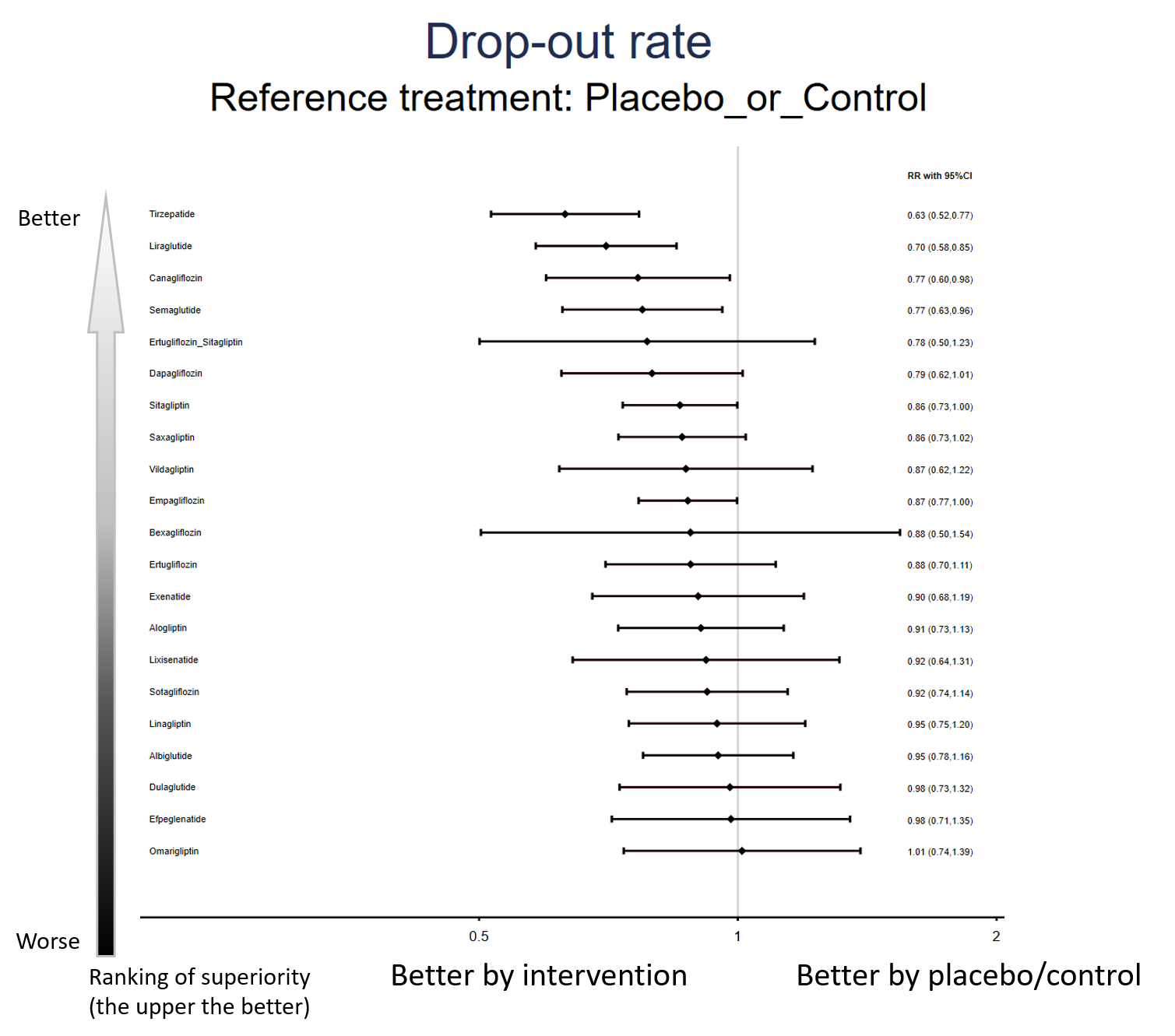
***

***Abbreviation for eFigure 2A-2T:***

*95%CIs: 95% confidence intervals; DPP4 inhibitor: dipeptidyl peptidase 4 inhibitor; GLP-1 agonist: glucagon-like peptide-1 agonist; NMA: network meta-analysis; RCT: randomized controlled trial; RR: risk ratio; SGLT2 inhibitor: sodium–glucose cotransporter 2 inhibitor*

**eFigure 3 Individual study result of primary outcome: overall hematologic malignancy risk**

***
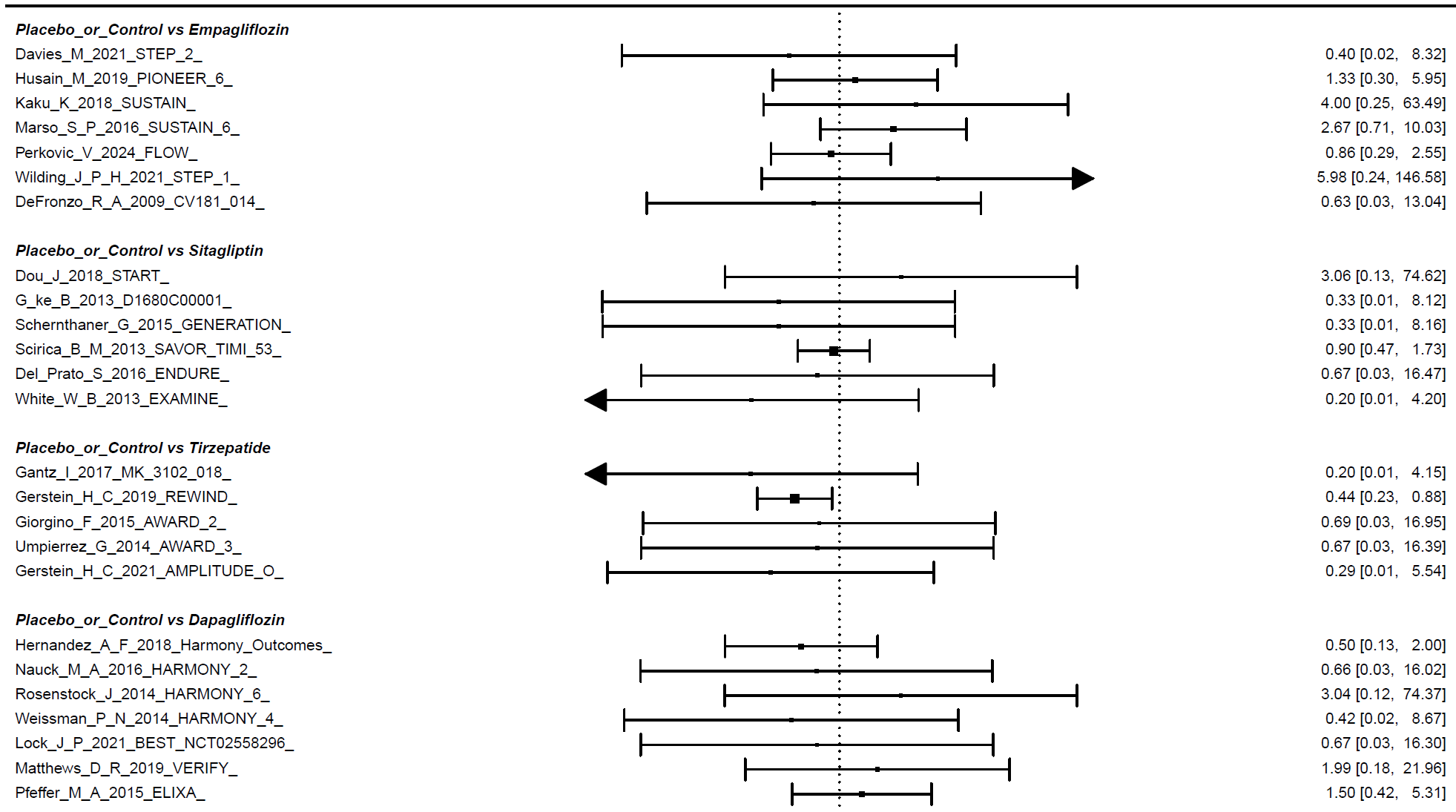
***

***
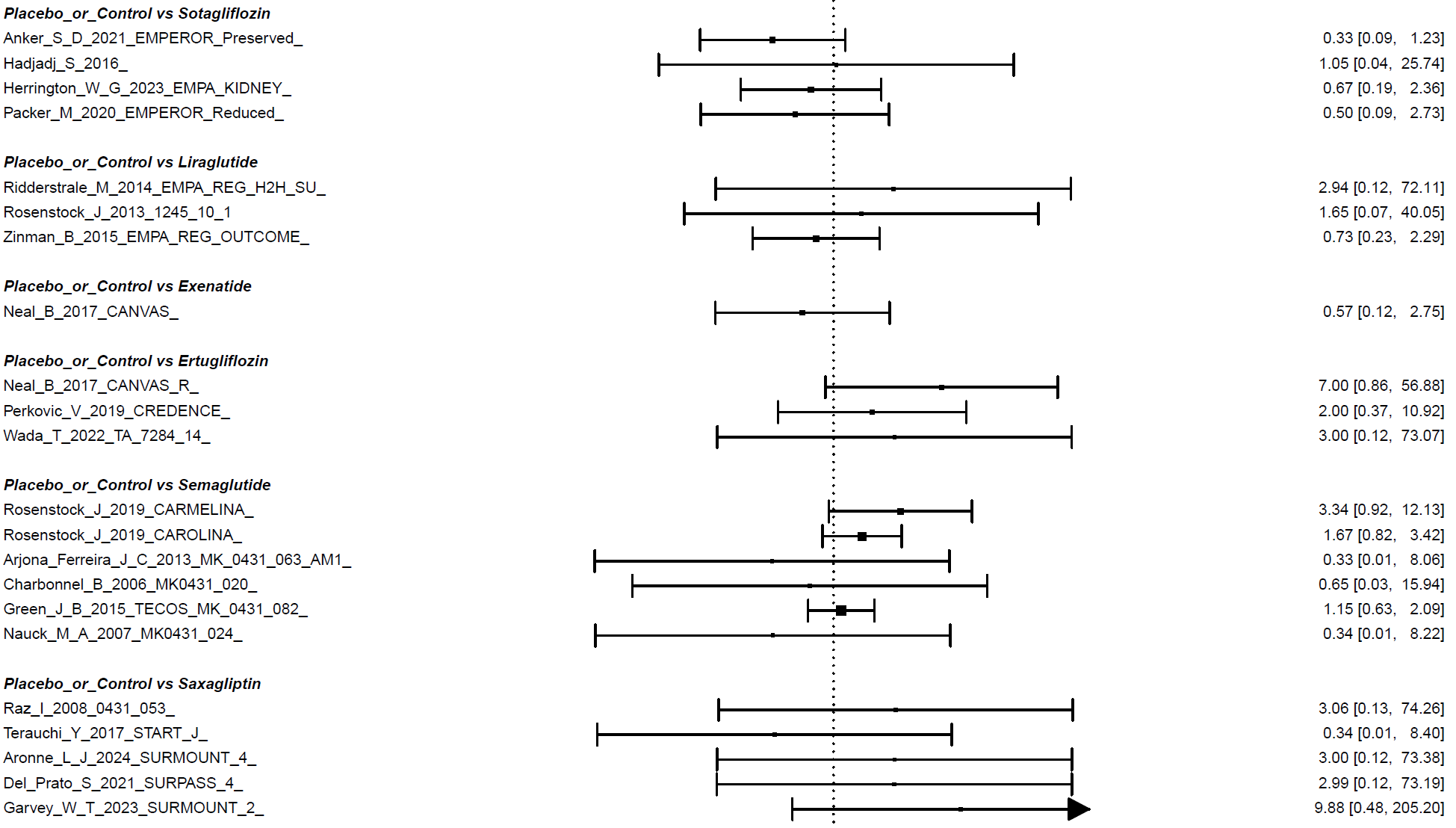
***

***
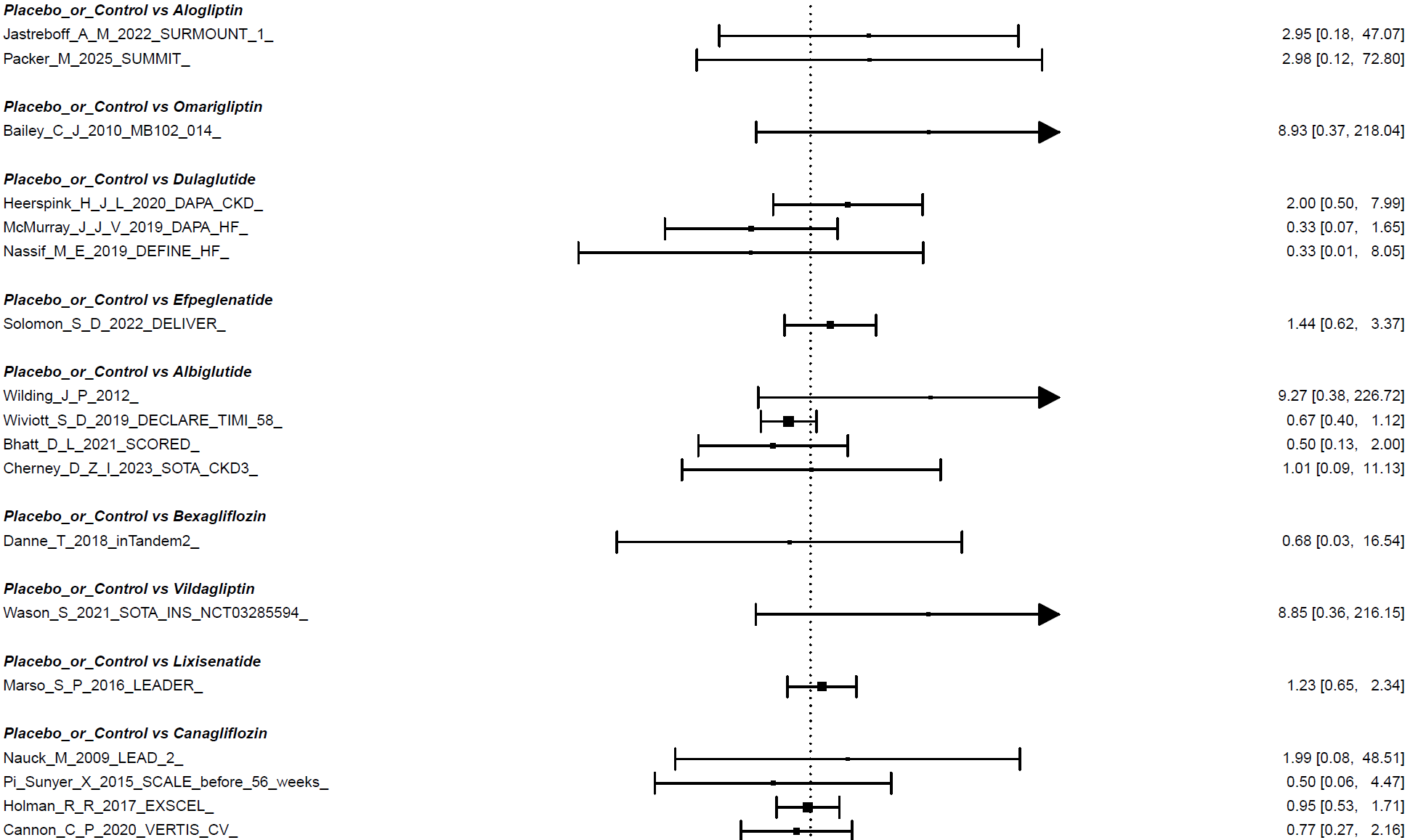
***

***
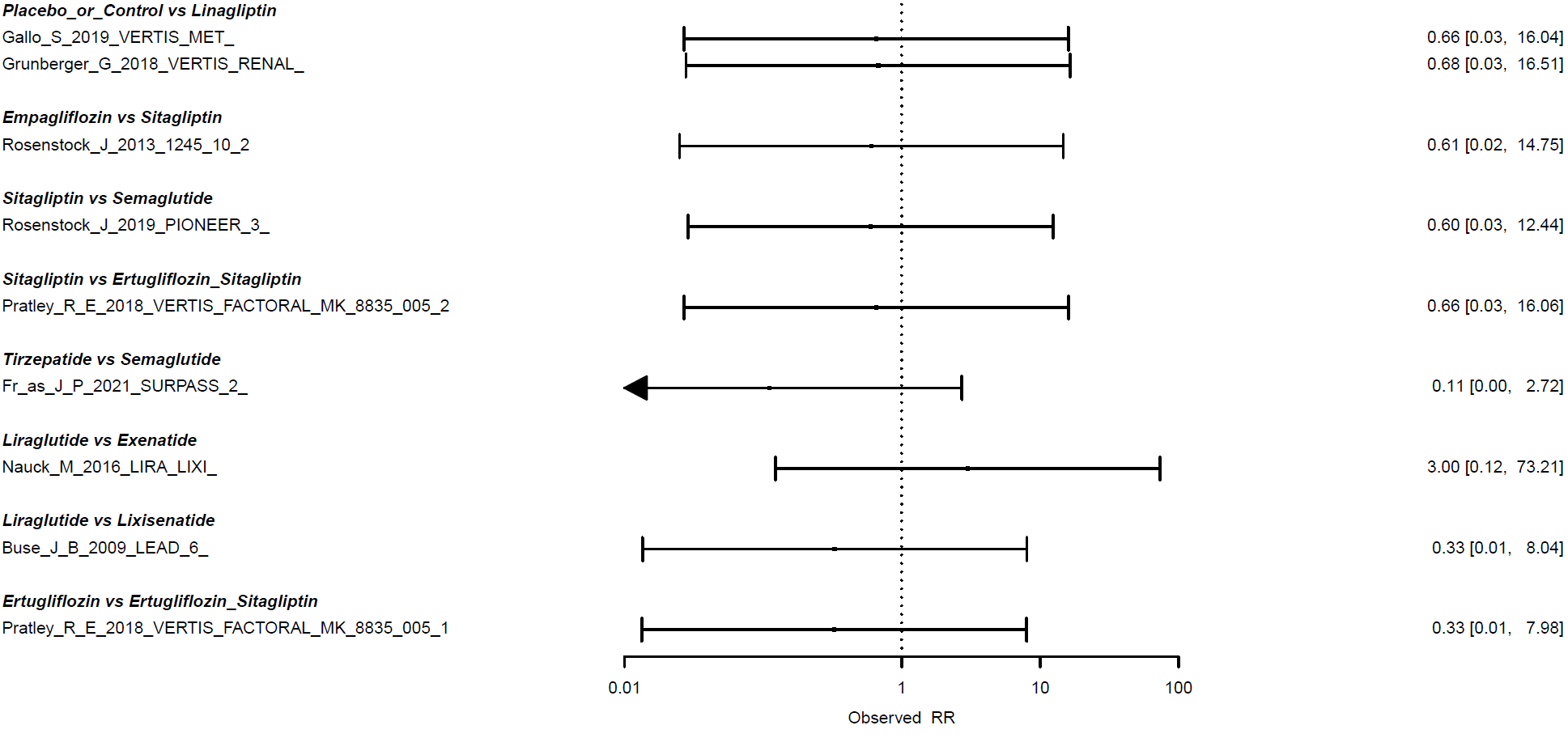
***

***Abbreviation for eFigure 3:***

*95%CIs: 95% confidence intervals; DPP4 inhibitor: dipeptidyl peptidase 4 inhibitor; GLP-1 agonist: glucagon-like peptide-1 agonist; NMA: network meta-analysis; RCT: randomized controlled trial; RR: risk ratio; SGLT2 inhibitor: sodium–glucose cotransporter 2 inhibitor*

**eFigure 4A Funnel plot for primary outcome: overall hematologic malignancy risk**

**
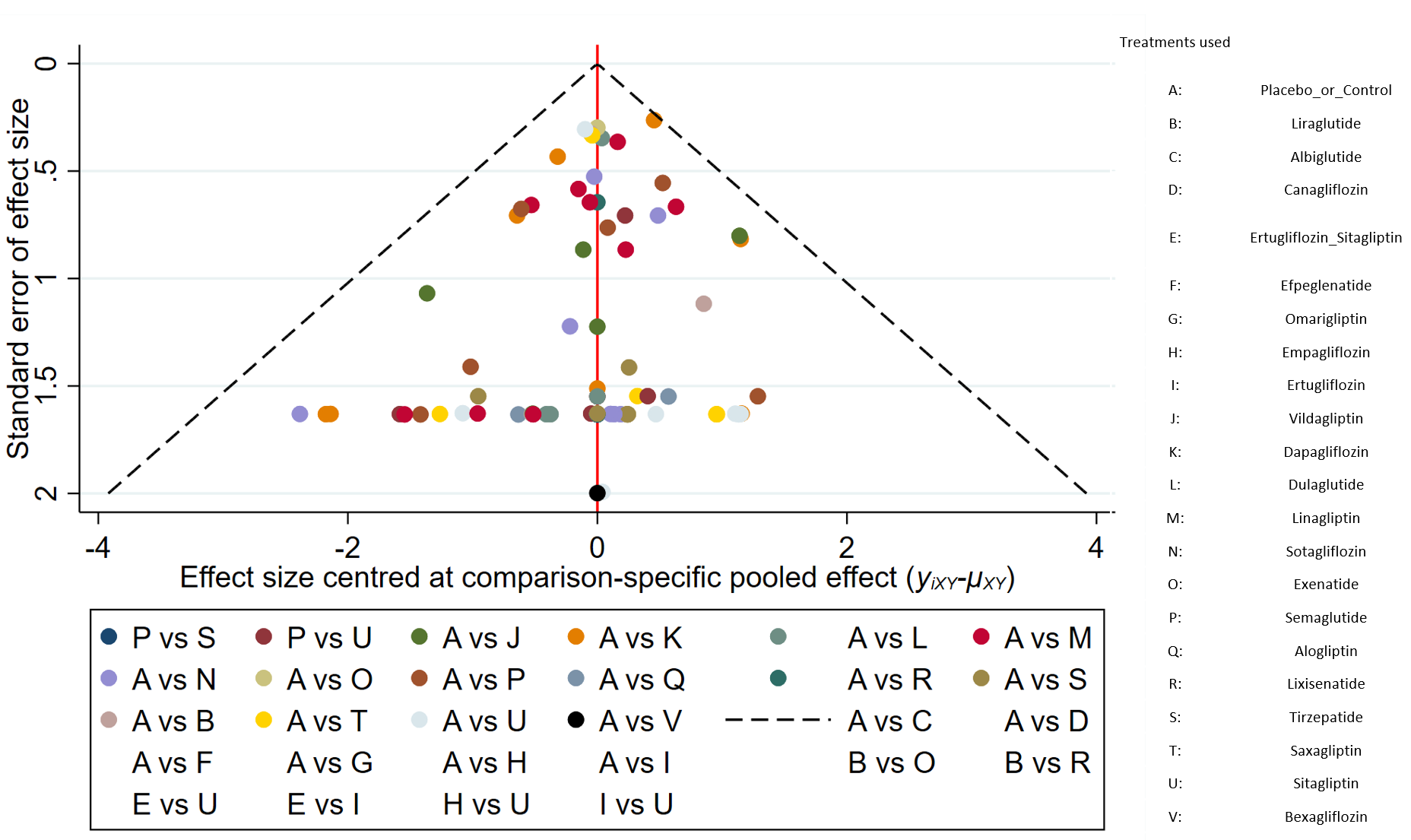
**

**eFigure 4B Funnel plot for primary outcome: leukemia risk**

**
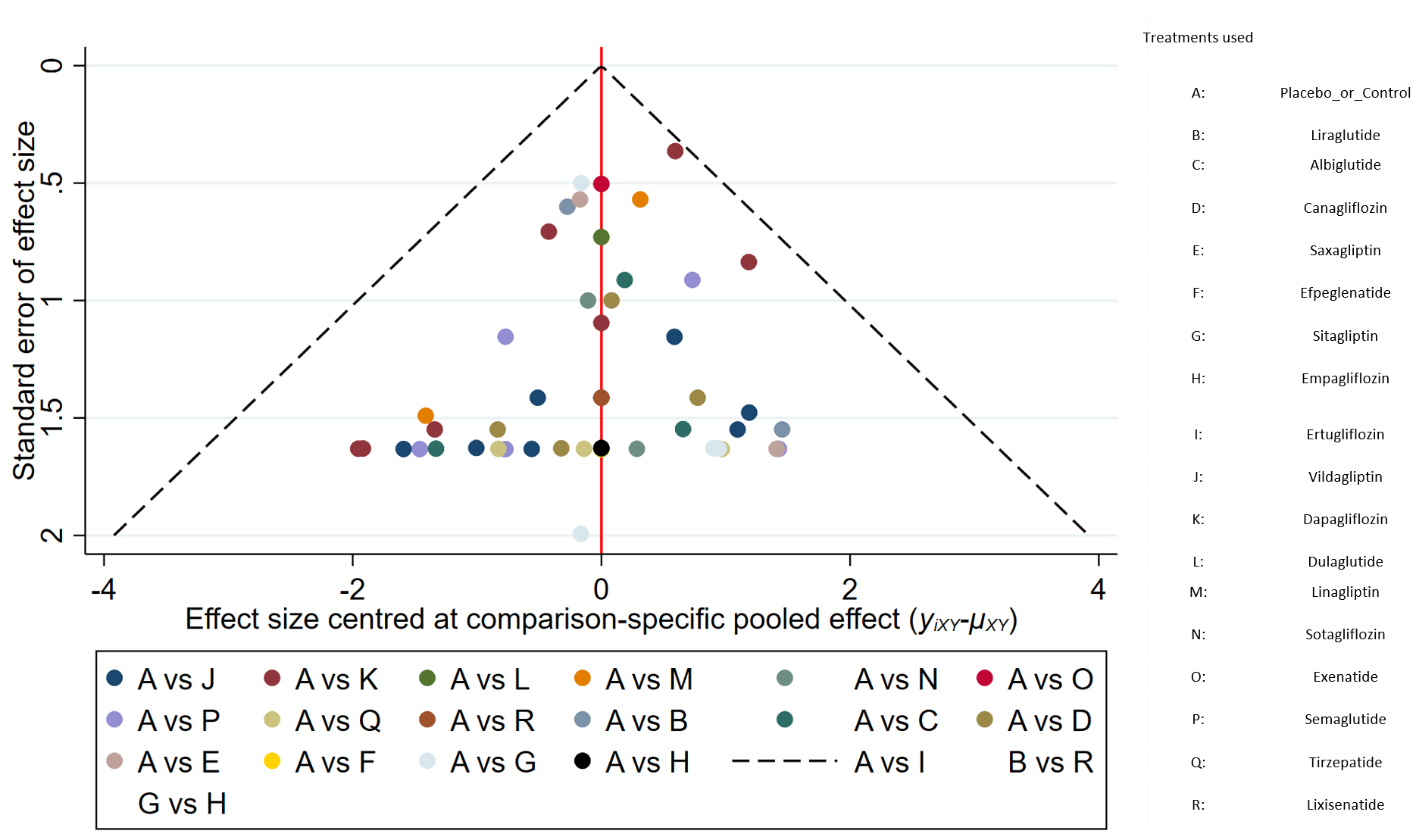
**

**eFigure 4C Funnel plot for primary outcome: acute lymphocytic leukemia risk**

**
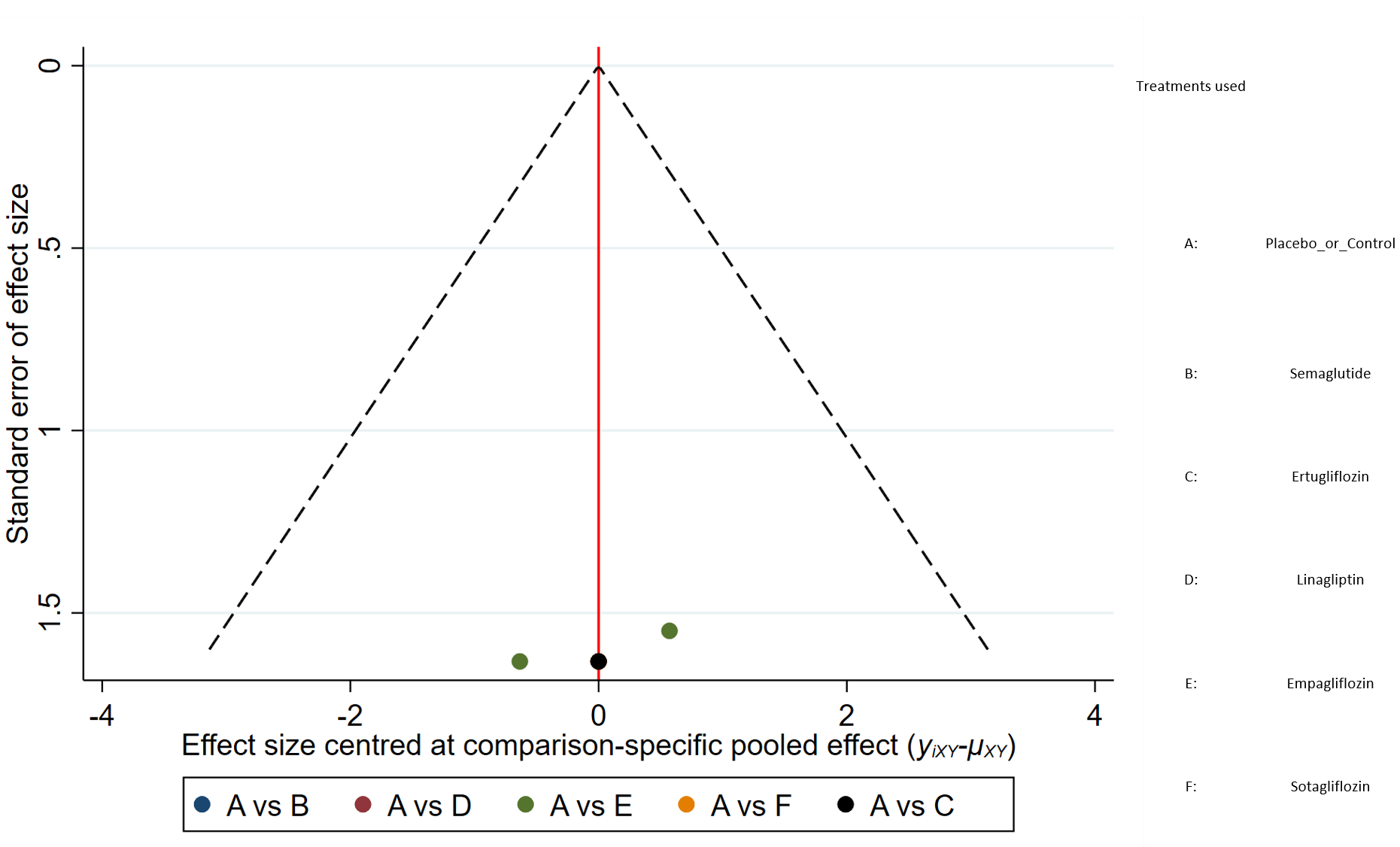
**

**eFigure 4D Funnel plot for primary outcome: acute myeloid leukemia risk**

**
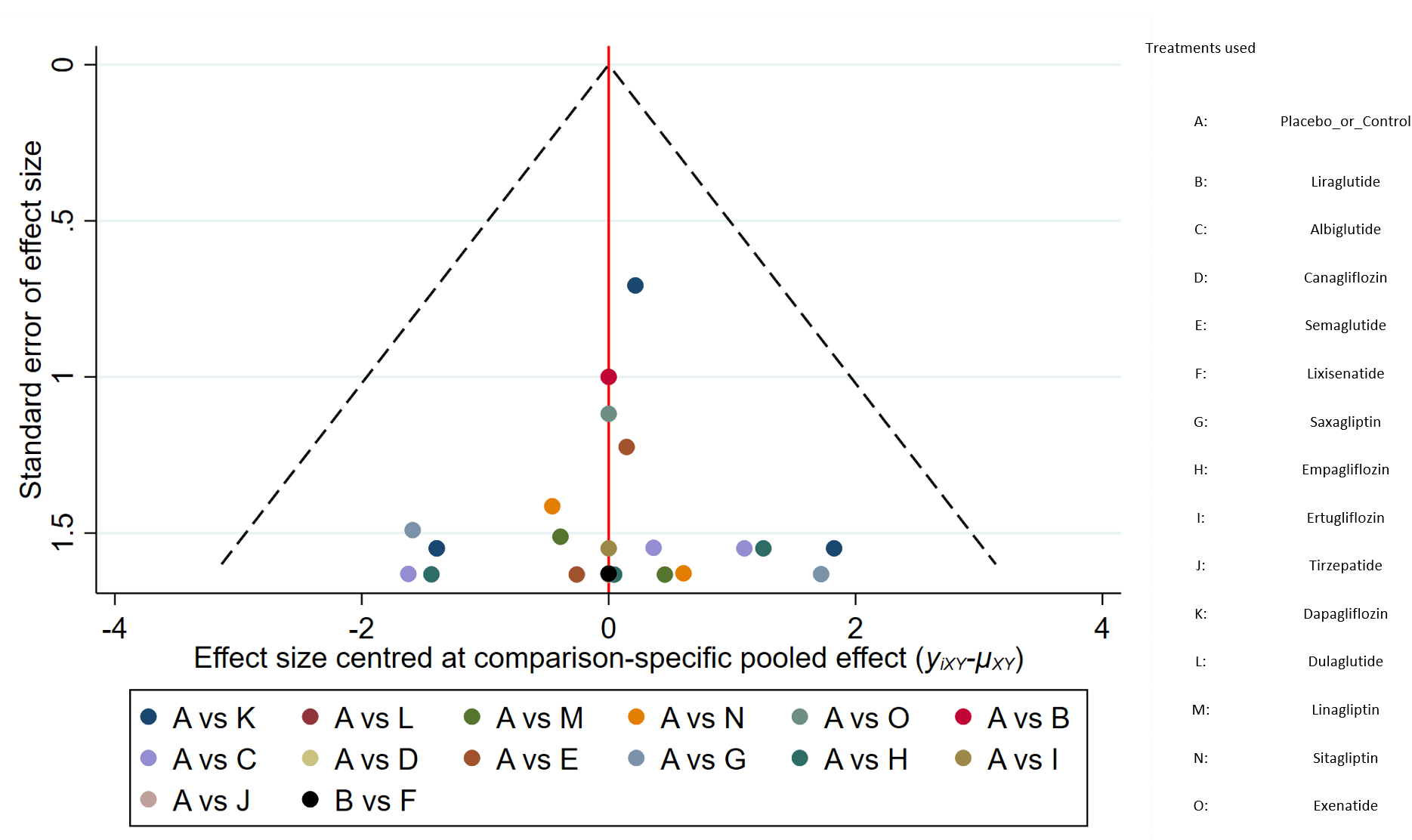
**

**eFigure 4E Funnel plot for primary outcome: chronic lymphocytic leukemia risk**

**
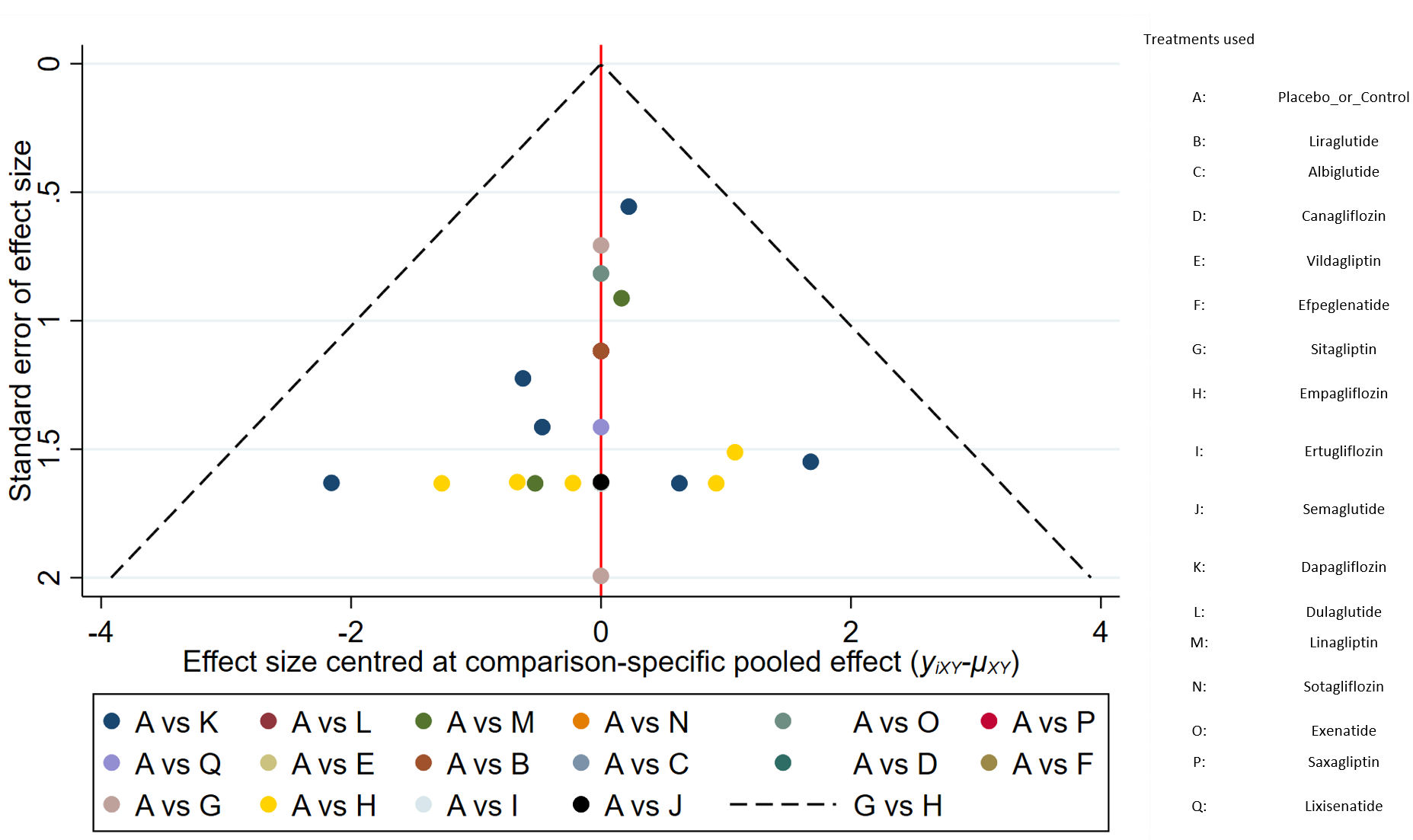
**

**eFigure 4F Funnel plot for primary outcome: chronic myeloid leukemia risk**

**
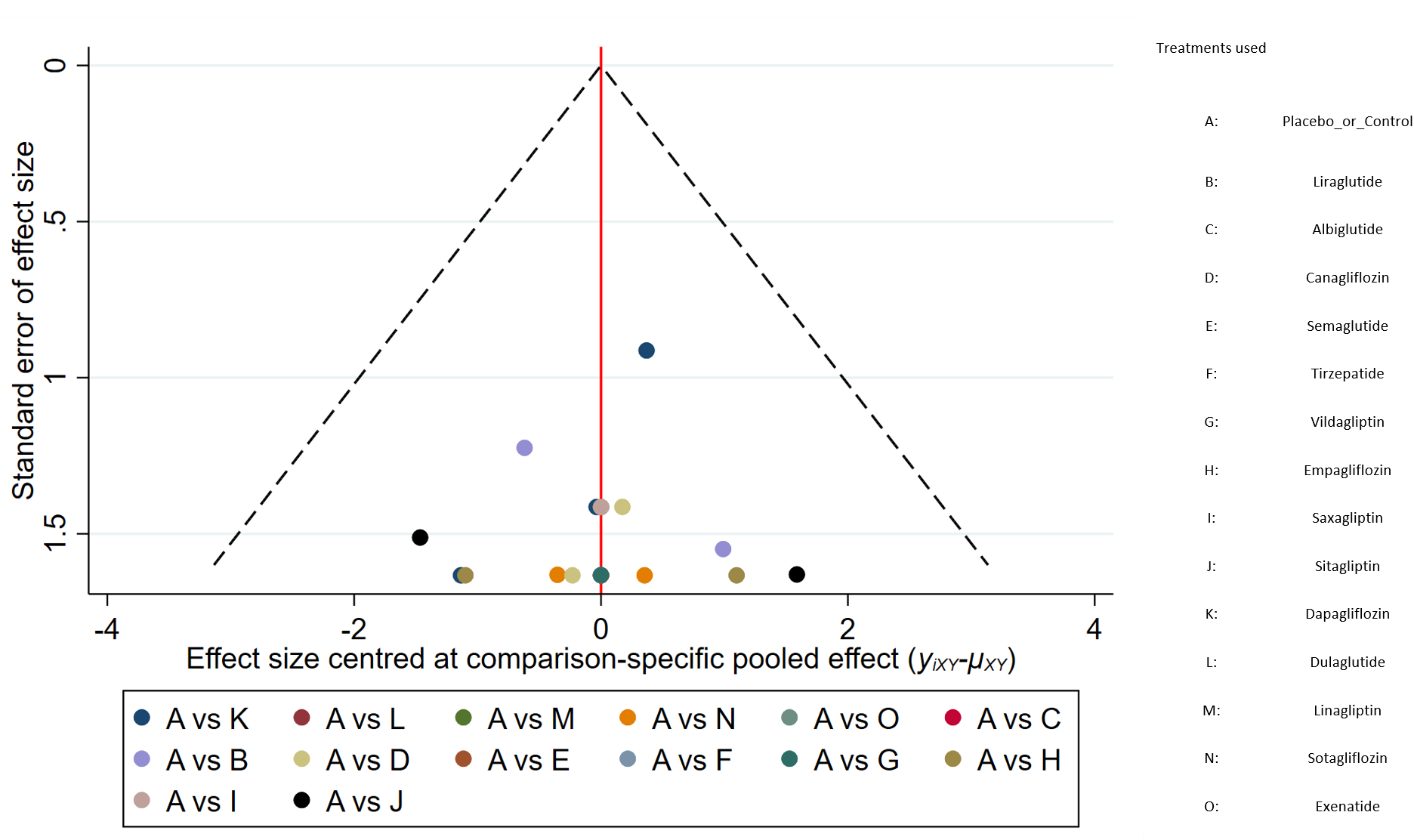
**

**eFigure 4G Funnel plot for primary outcome: lymphoma risk**

**
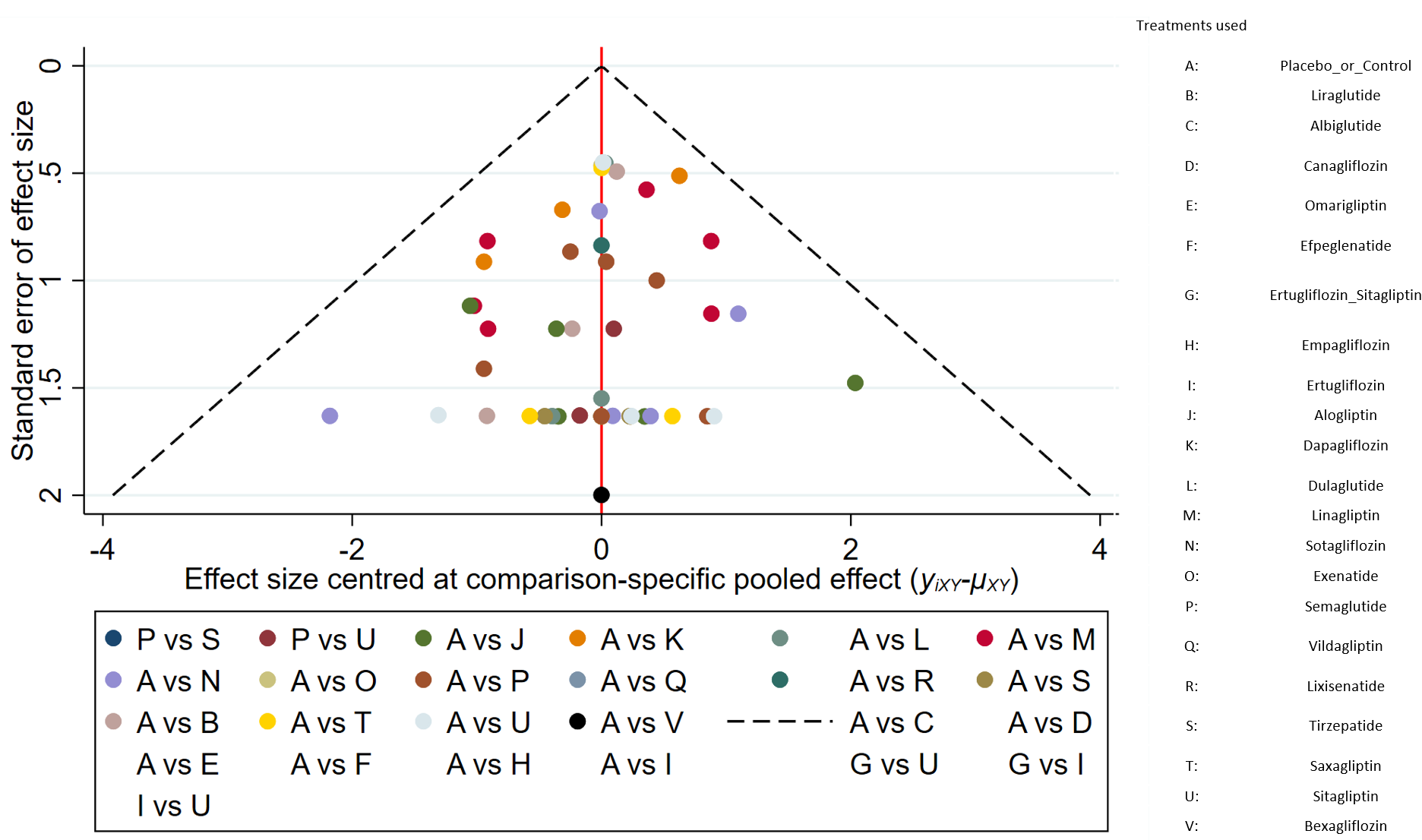
**

**eFigure 4H Funnel plot for primary outcome: non-Hodgkin's lymphoma risk**

**
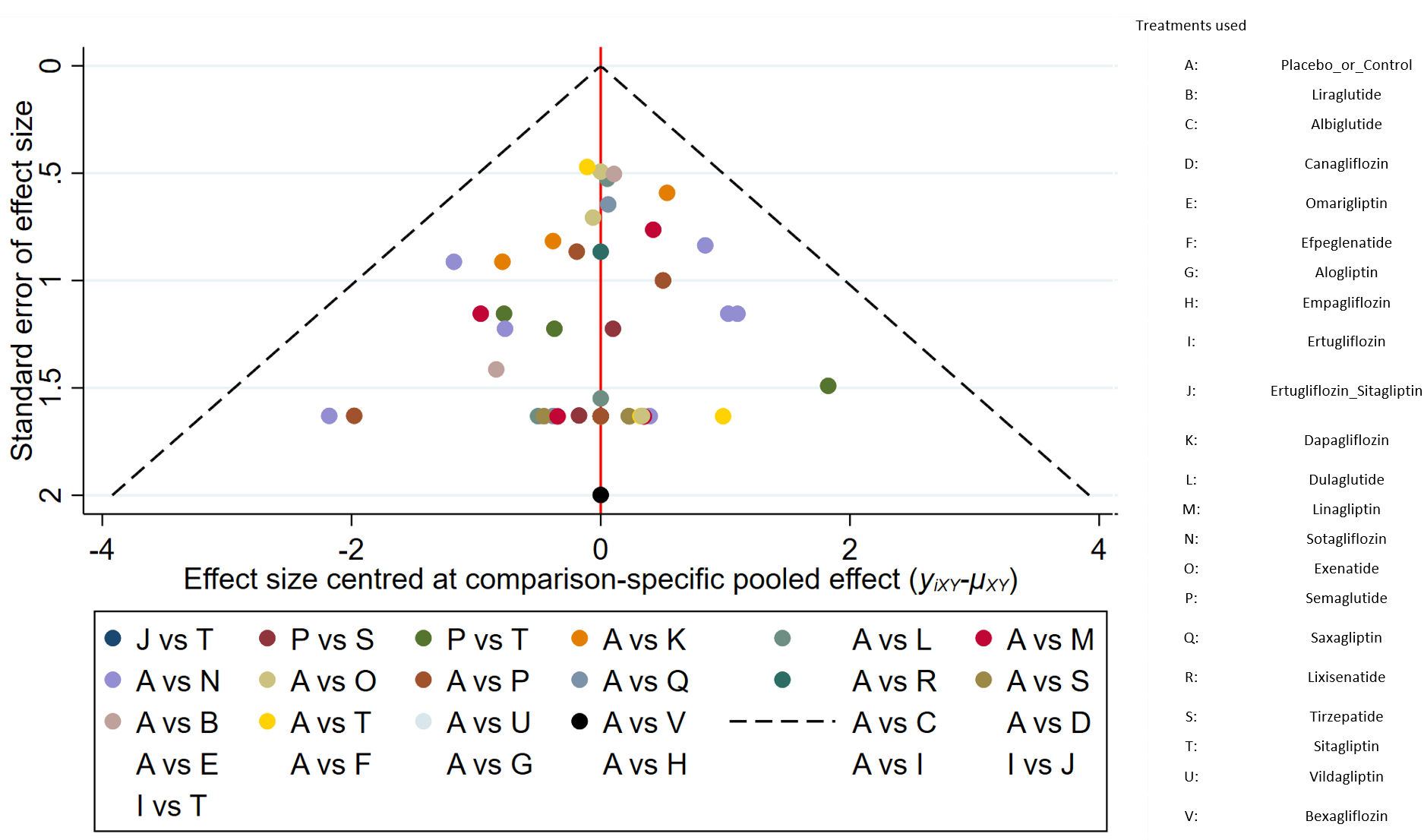
**

**eFigure 4I Funnel plot for primary outcome: B cell non-Hodgkin's lymphoma risk**

**
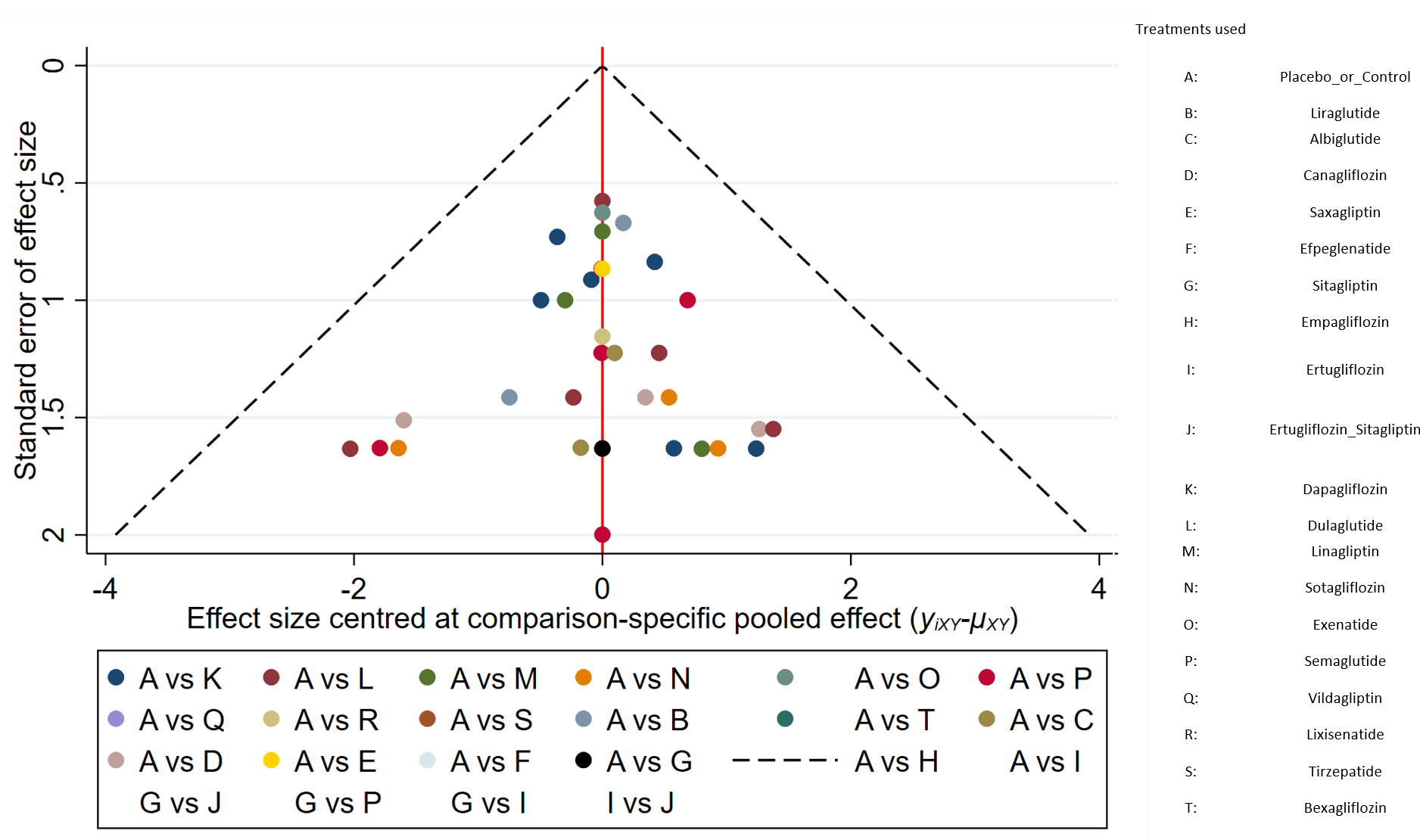
**

**eFigure 4J Funnel plot for primary outcome: T cell non-Hodgkin's lymphoma risk**

**
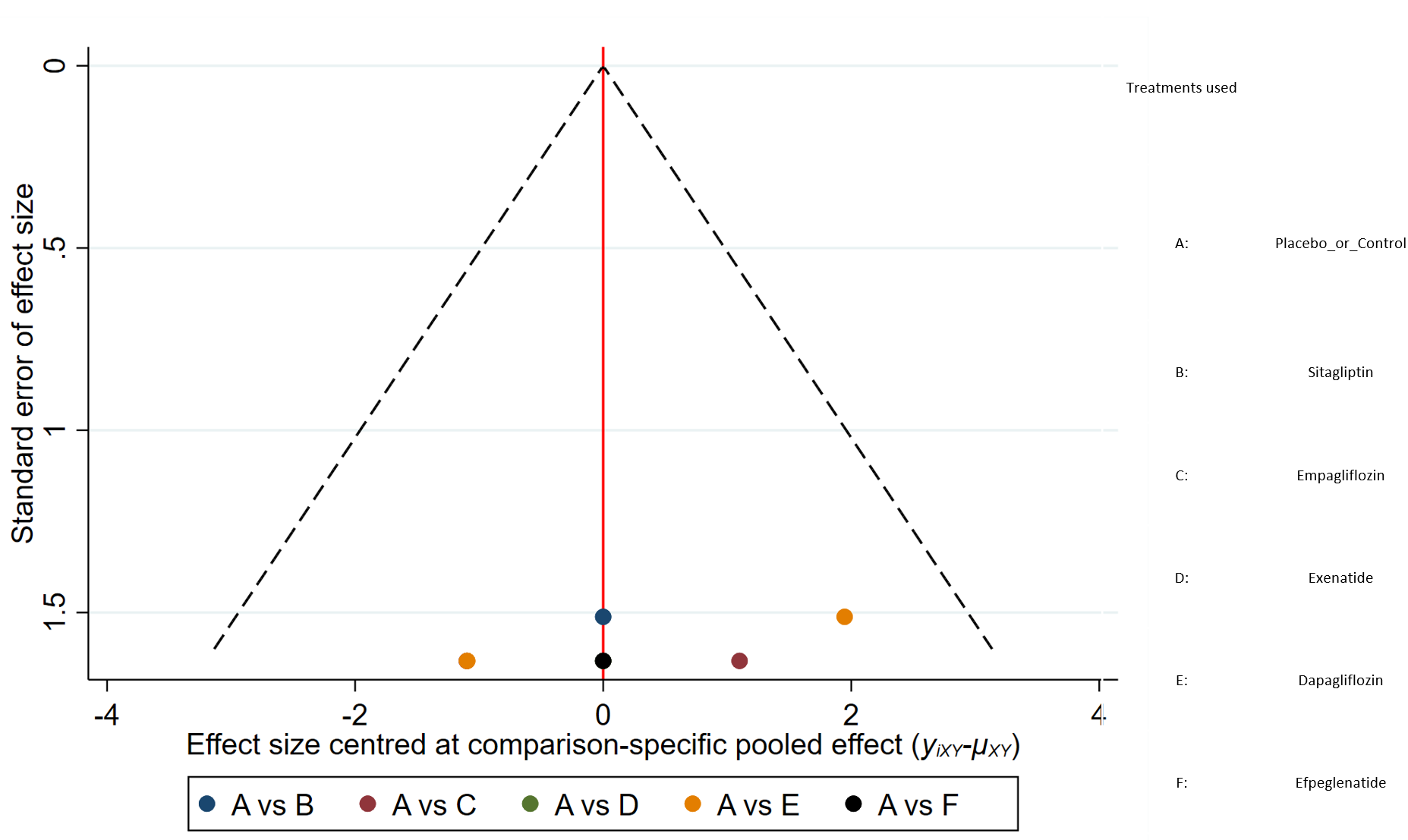
**

**eFigure 4K Funnel plot for primary outcome: myeloma risk**

**
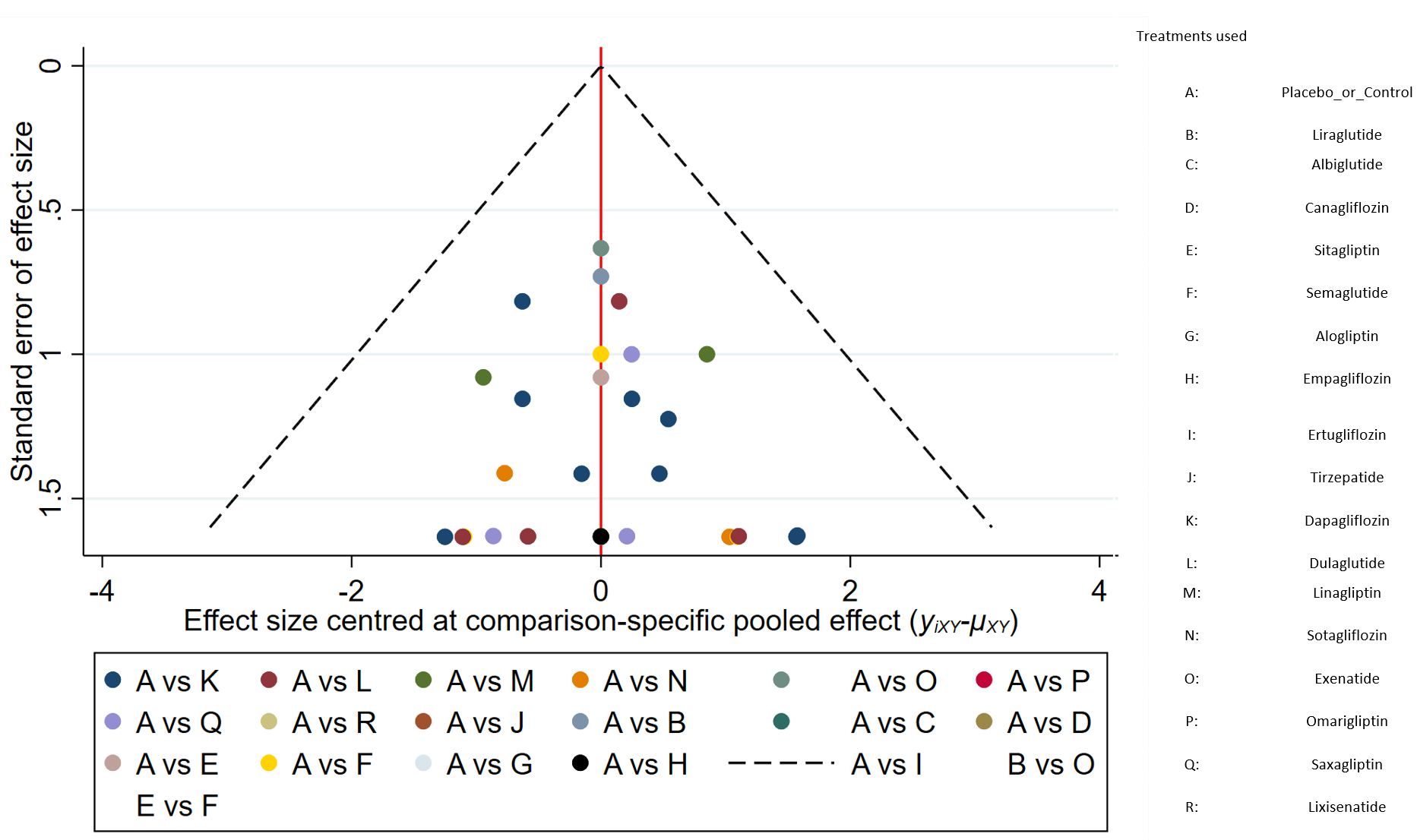
**

**eFigure 4L Funnel plot for primary outcome: plasma cell myeloma risk**

***
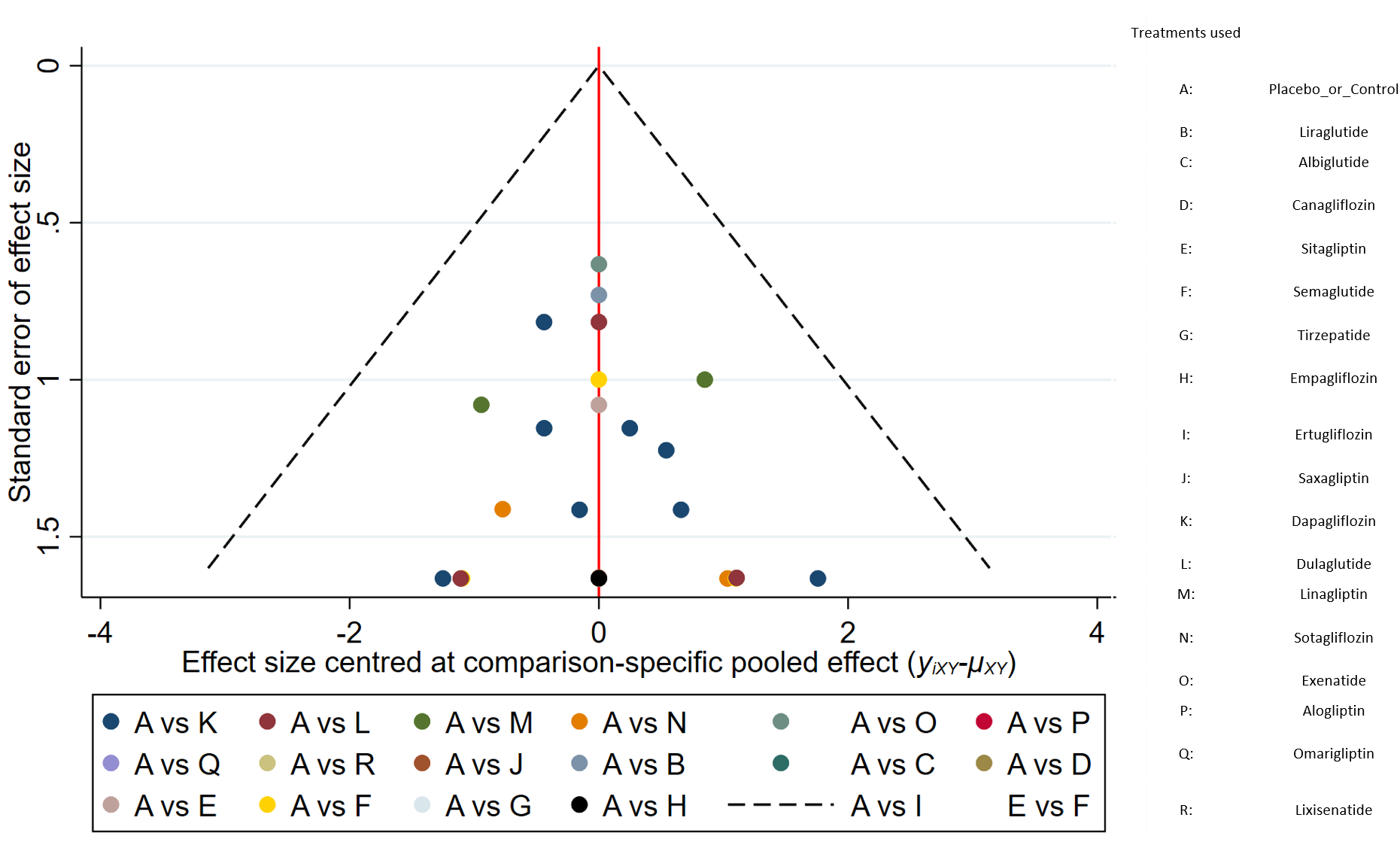
***

***Abbreviation for eFigure 4A-4L:***

*95%CIs: 95% confidence intervals; DPP4 inhibitor: dipeptidyl peptidase 4 inhibitor; GLP-1 agonist: glucagon-like peptide-1 agonist; NMA: network meta-analysis; RCT: randomized controlled trial; RR: risk ratio; SGLT2 inhibitor: sodium–glucose cotransporter 2 inhibitor*

**eFigure 5A Egger test for primary outcome: overall hematologic malignancy risk**


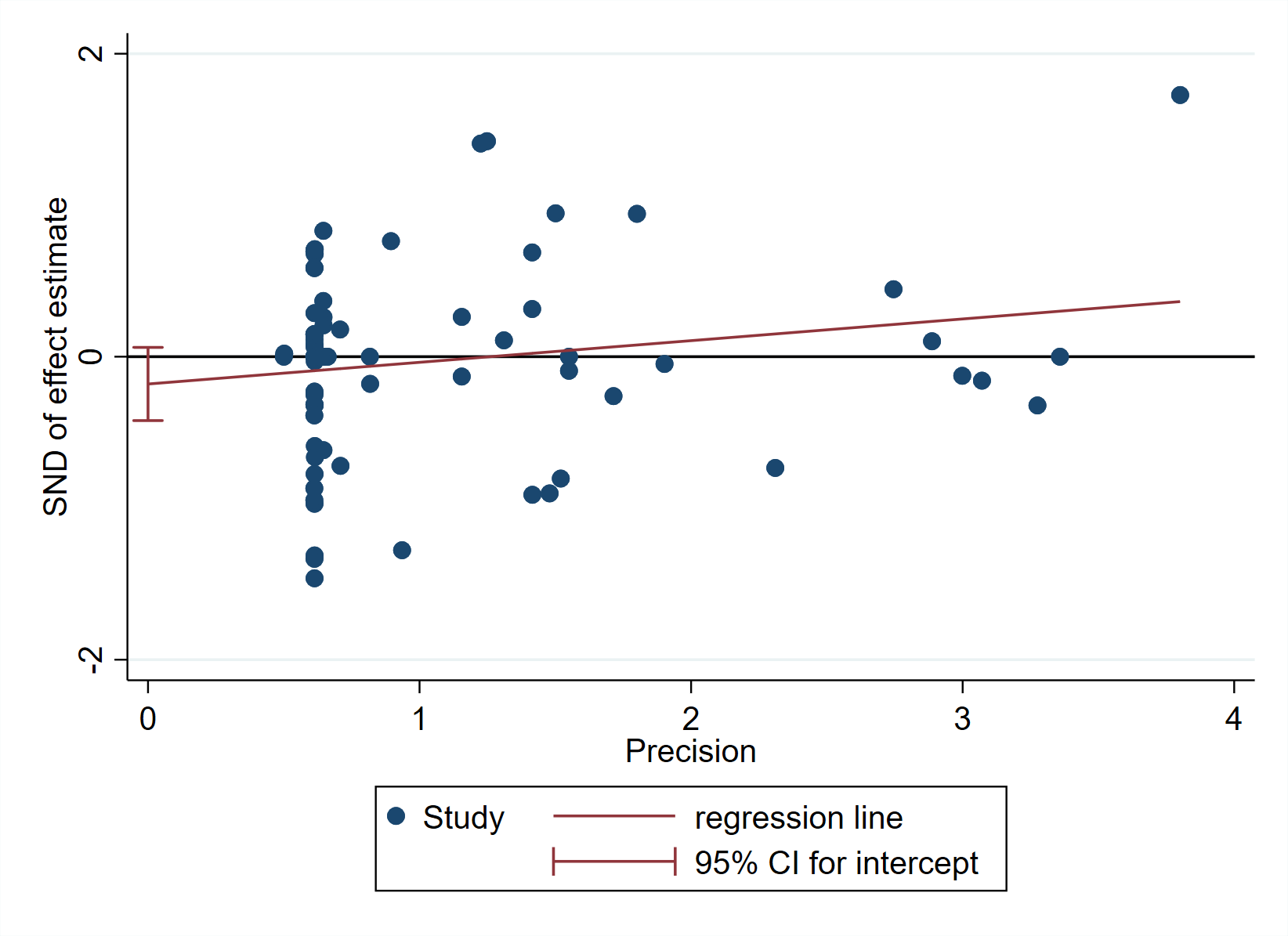


**eFigure 5B Egger test for primary outcome: leukemia risk**


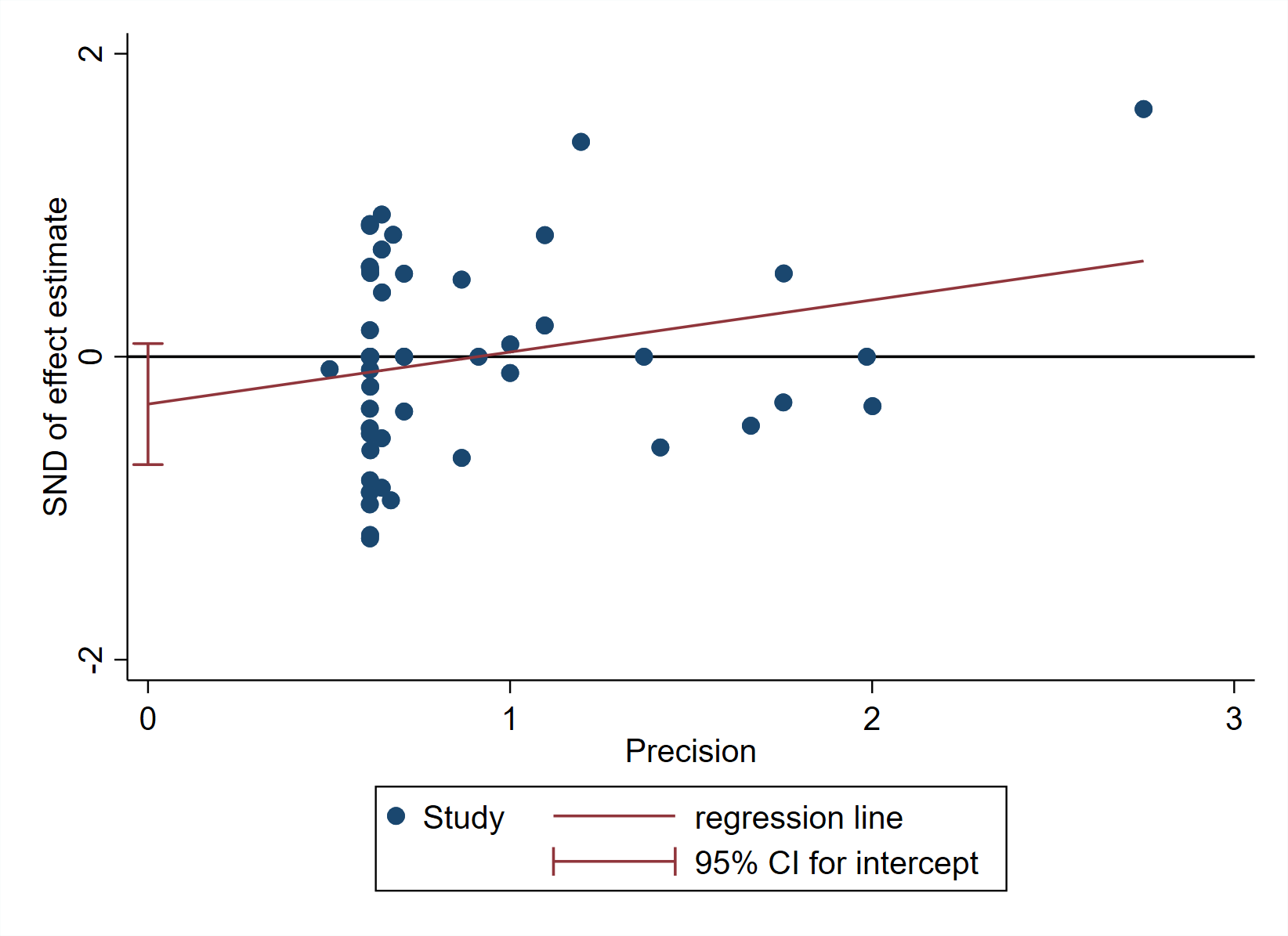


**eFigure 5C Egger test for primary outcome: acute lymphocytic leukemia risk**


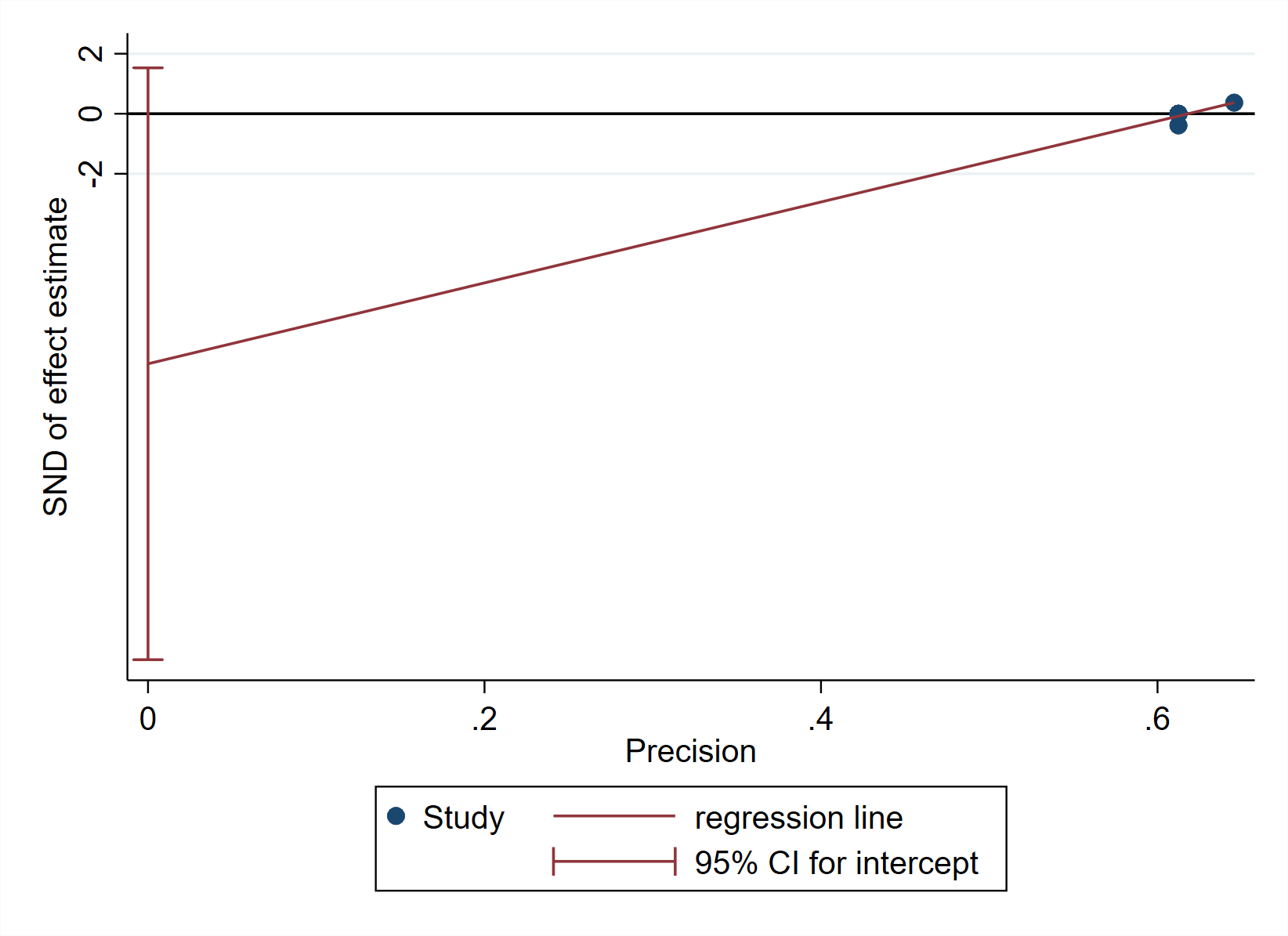


**eFigure 5D Egger test for primary outcome: acute myeloid leukemia risk**


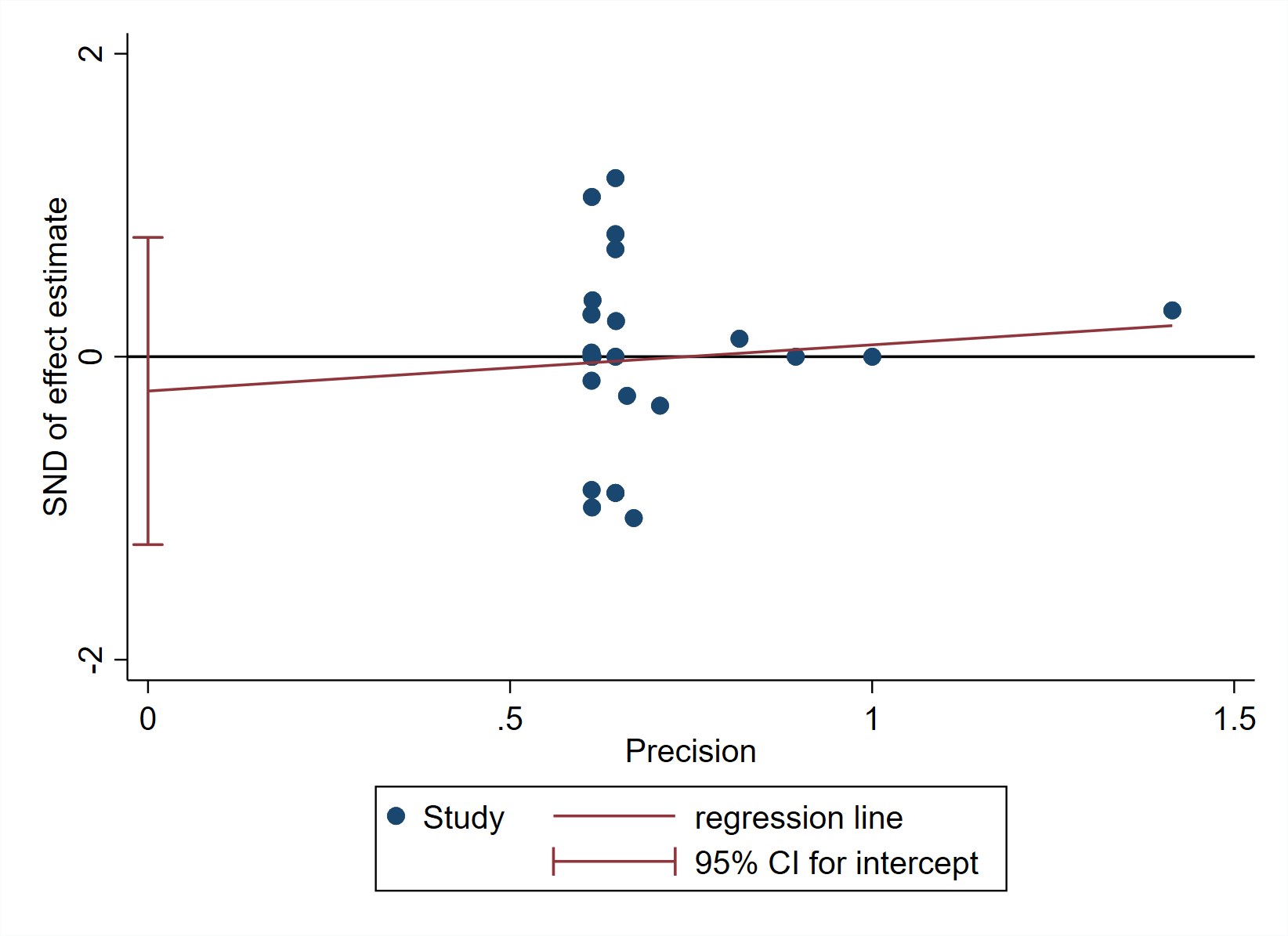


**eFigure 5E Egger test for primary outcome: chronic lymphocytic leukemia risk**


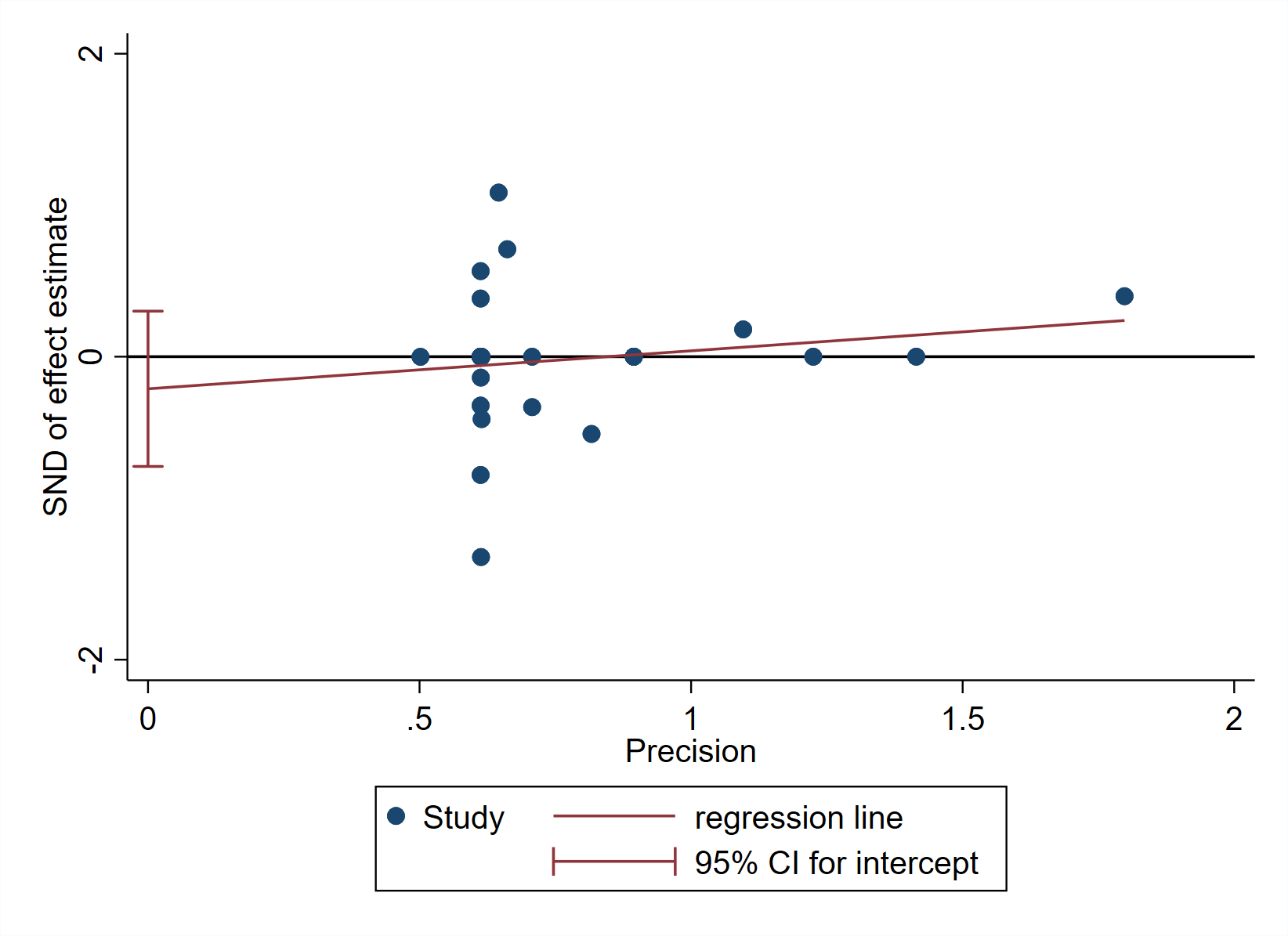


**eFigure 5F Egger test for primary outcome: chronic myeloid leukemia risk**


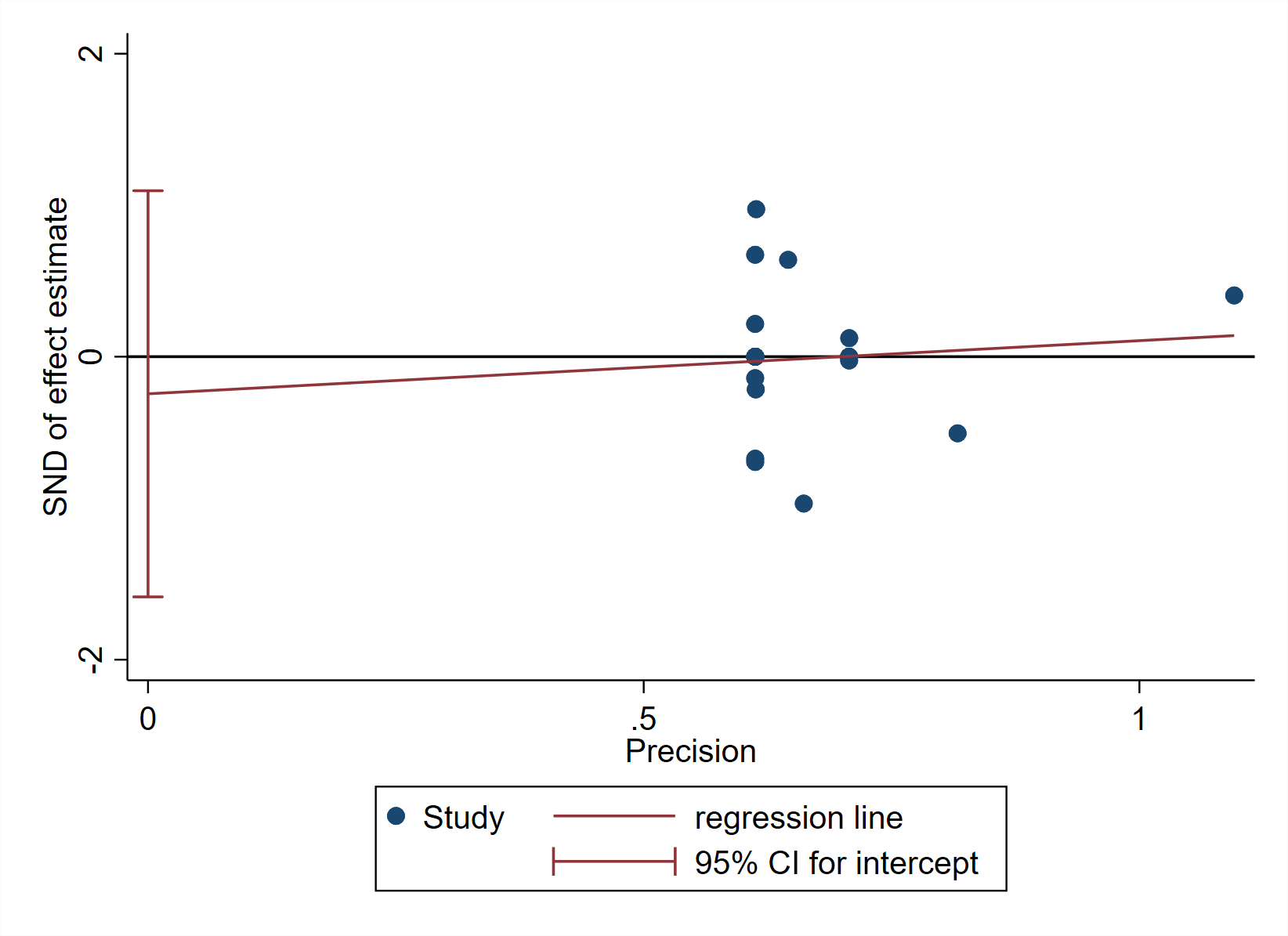


**eFigure 5G Egger test for primary outcome: lymphoma risk**


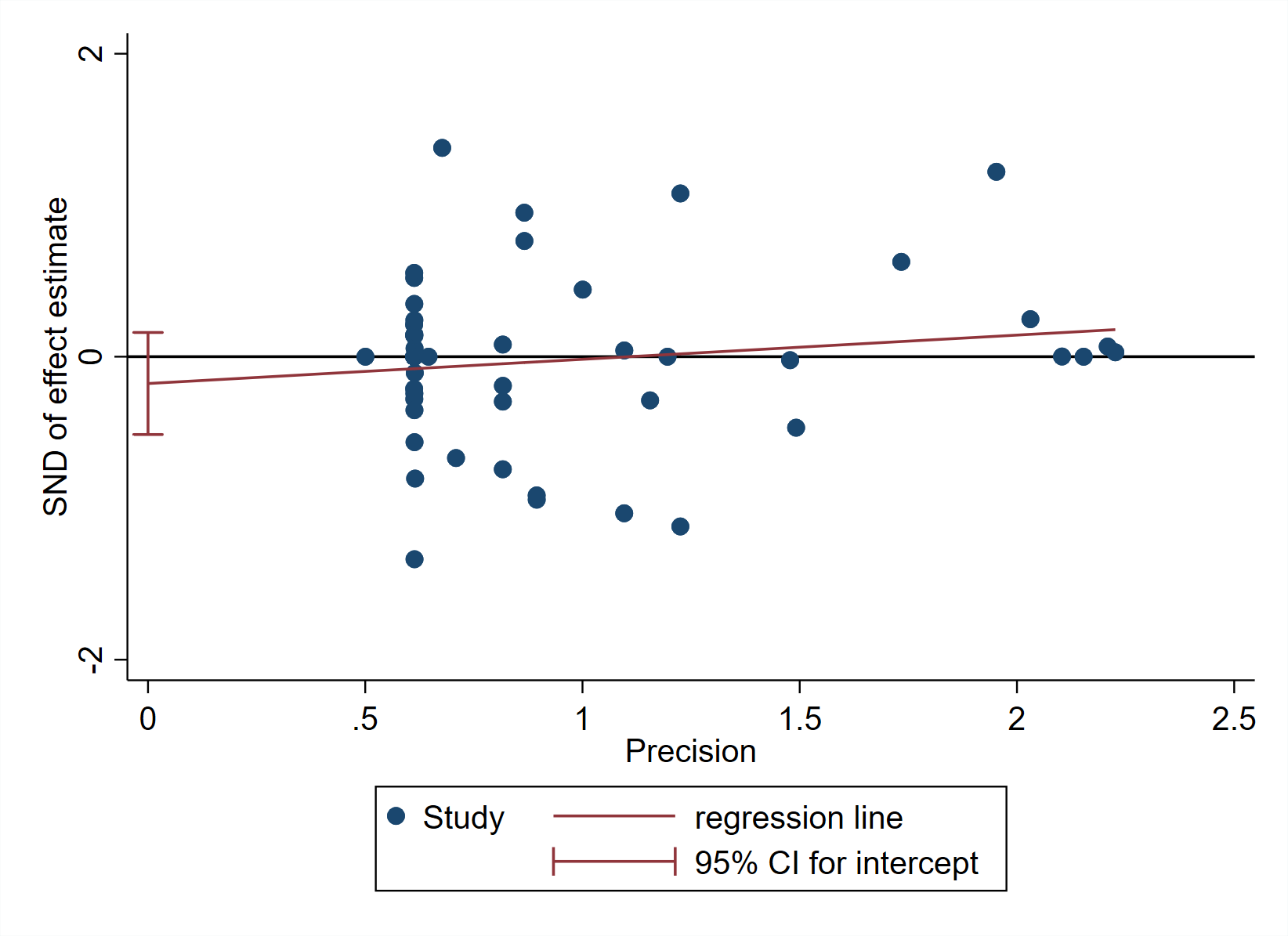


**eFigure 5H Egger test for primary outcome: non-Hodgkin's lymphoma risk**


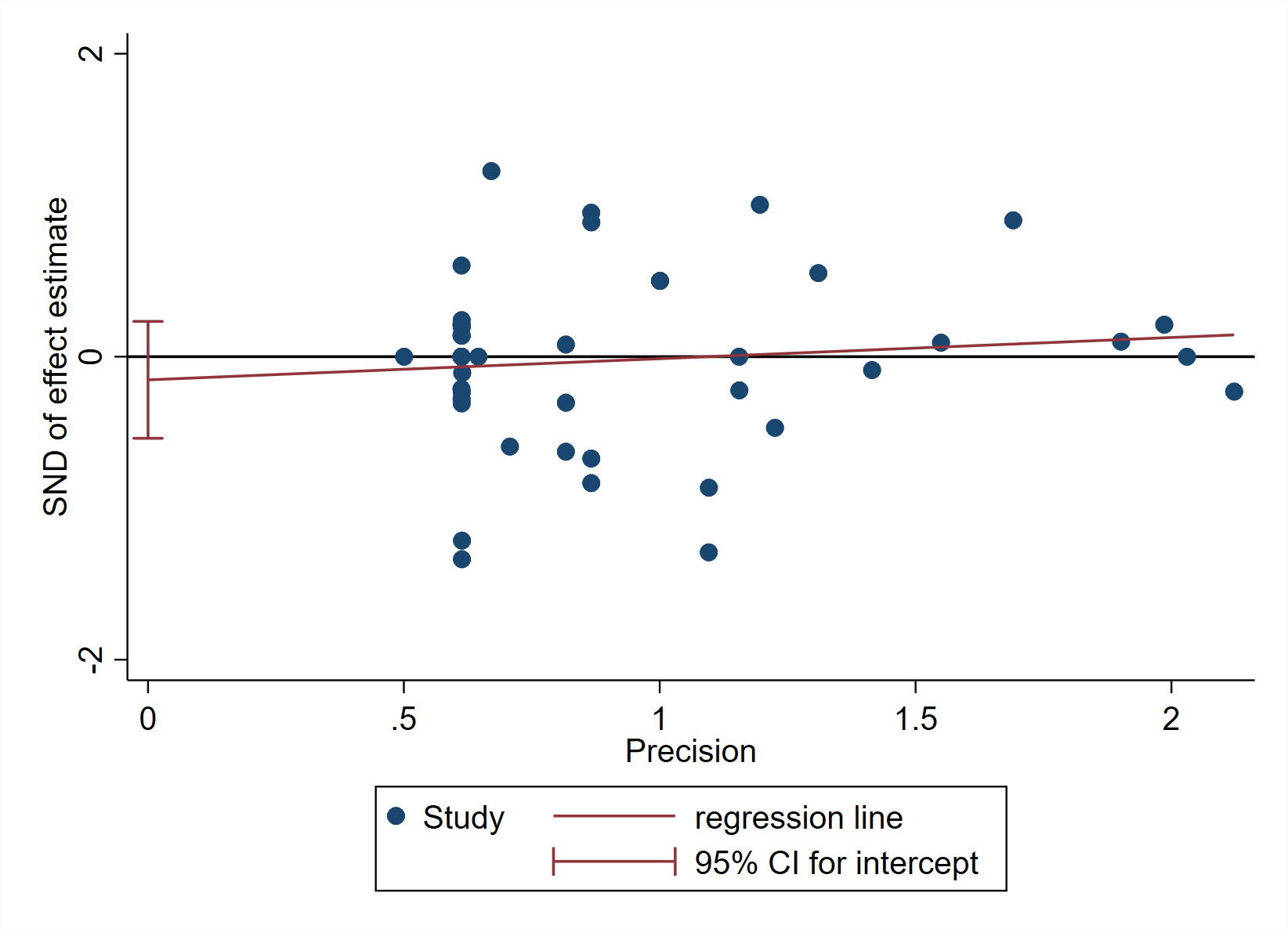


**eFigure 5I Egger test for primary outcome: B cell non-Hodgkin's lymphoma risk**


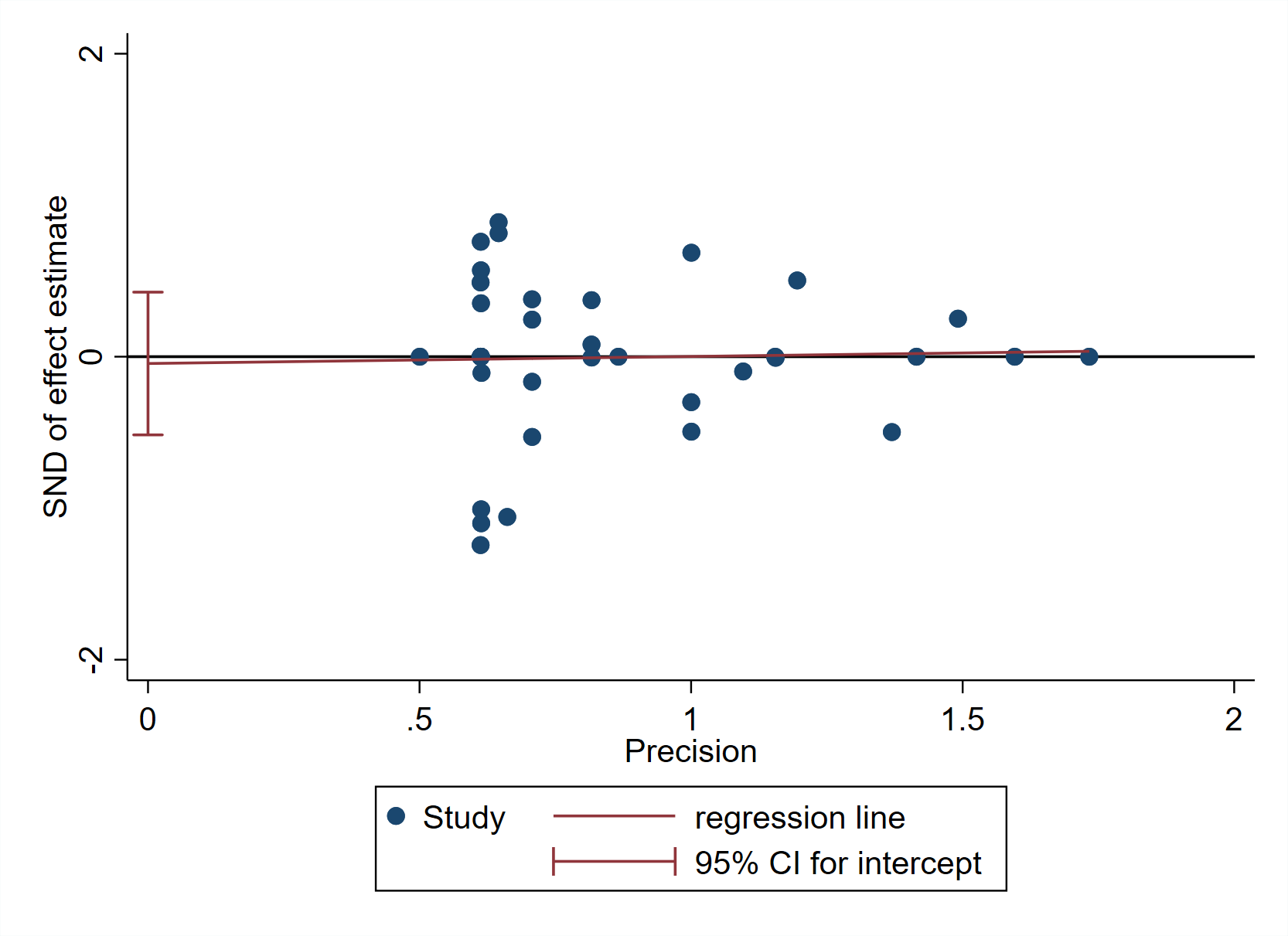


**eFigure 5J Egger test for primary outcome: T cell non-Hodgkin's lymphoma risk**


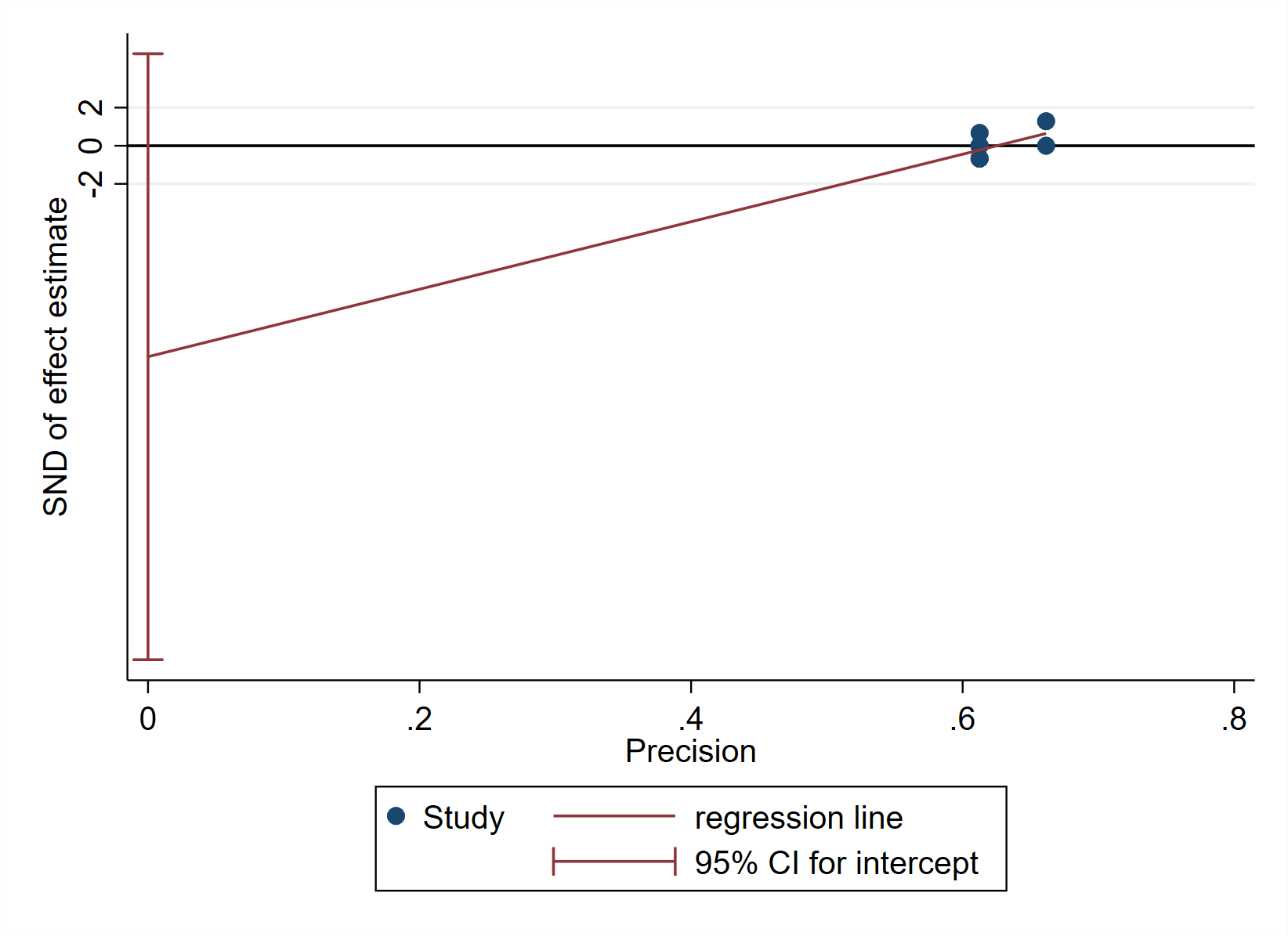


**eFigure 5K Egger test for primary outcome: myeloma risk**


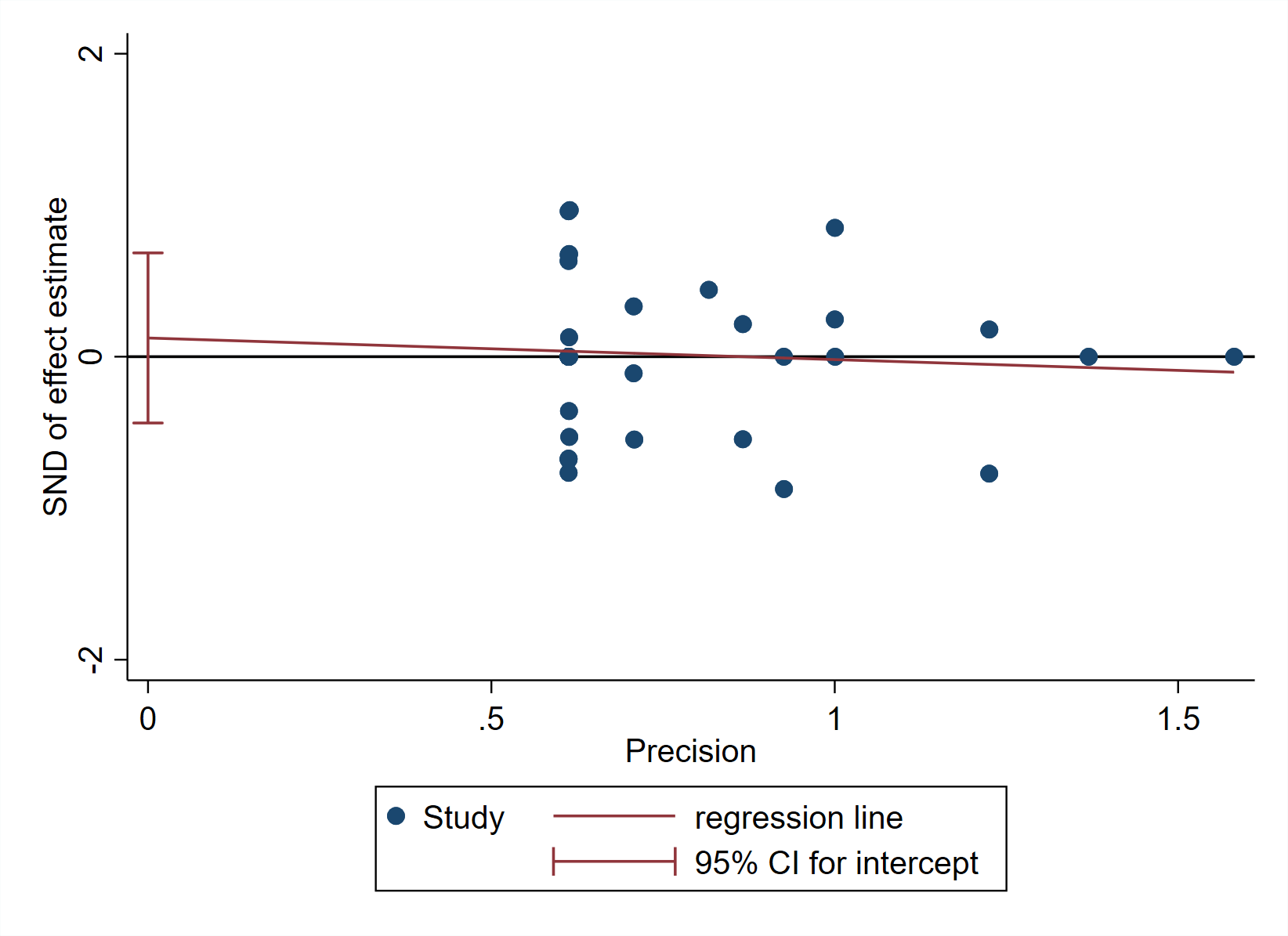


**eFigure 5L Egger test for primary outcome: plasma cell myeloma risk**


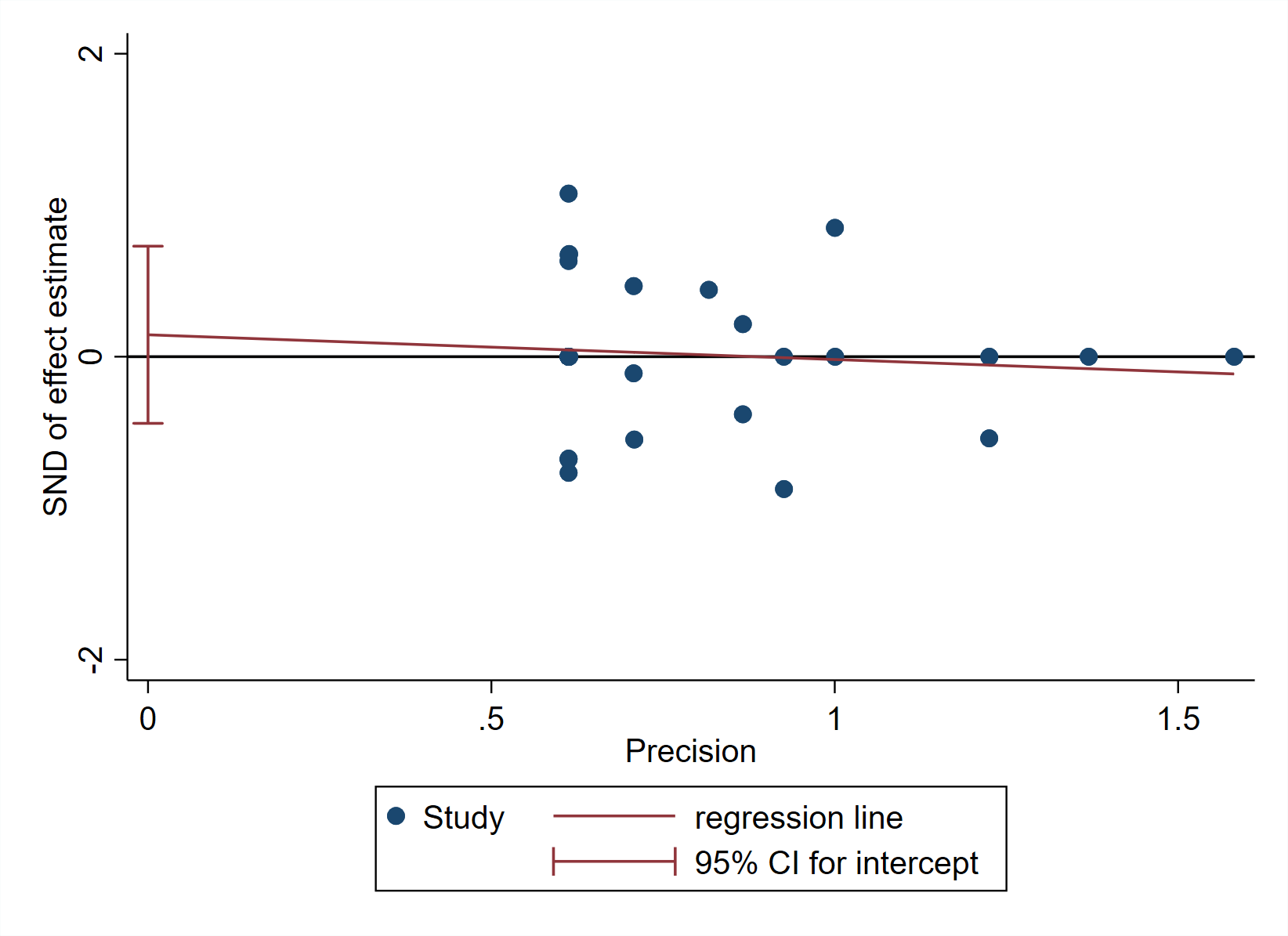


***Abbreviation for eFigure 5A-5L:***

*95%CIs: 95% confidence intervals; DPP4 inhibitor: dipeptidyl peptidase 4 inhibitor; GLP-1 agonist: glucagon-like peptide-1 agonist; NMA: network meta-analysis; RCT: randomized controlled trial; RR: risk ratio; SGLT2 inhibitor: sodium–glucose cotransporter 2 inhibitor*

**eFigure 6 Bayesian-based forest plot of NMA of primary outcome: overall hematologic malignancy risk**

***
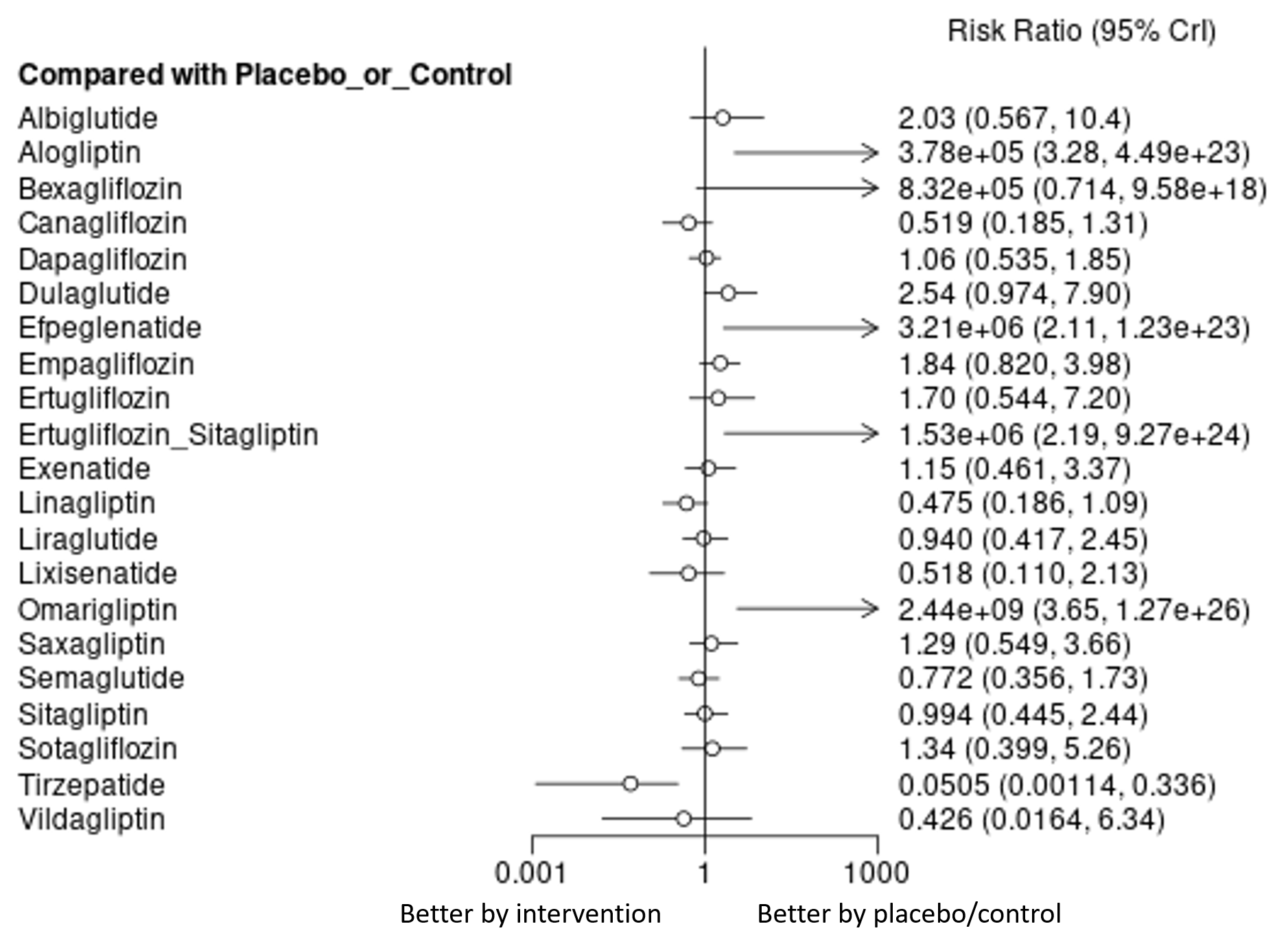
***

***Abbreviation for eFigure 6:***

*95%CIs: 95% confidence intervals; DPP4 inhibitor: dipeptidyl peptidase 4 inhibitor; GLP-1 agonist: glucagon-like peptide-1 agonist; NMA: network meta-analysis; RCT: randomized controlled trial; RR: risk ratio; SGLT2 inhibitor: sodium–glucose cotransporter 2 inhibitor*

**eFigure 7 Risk of bias tool 2.0**

**
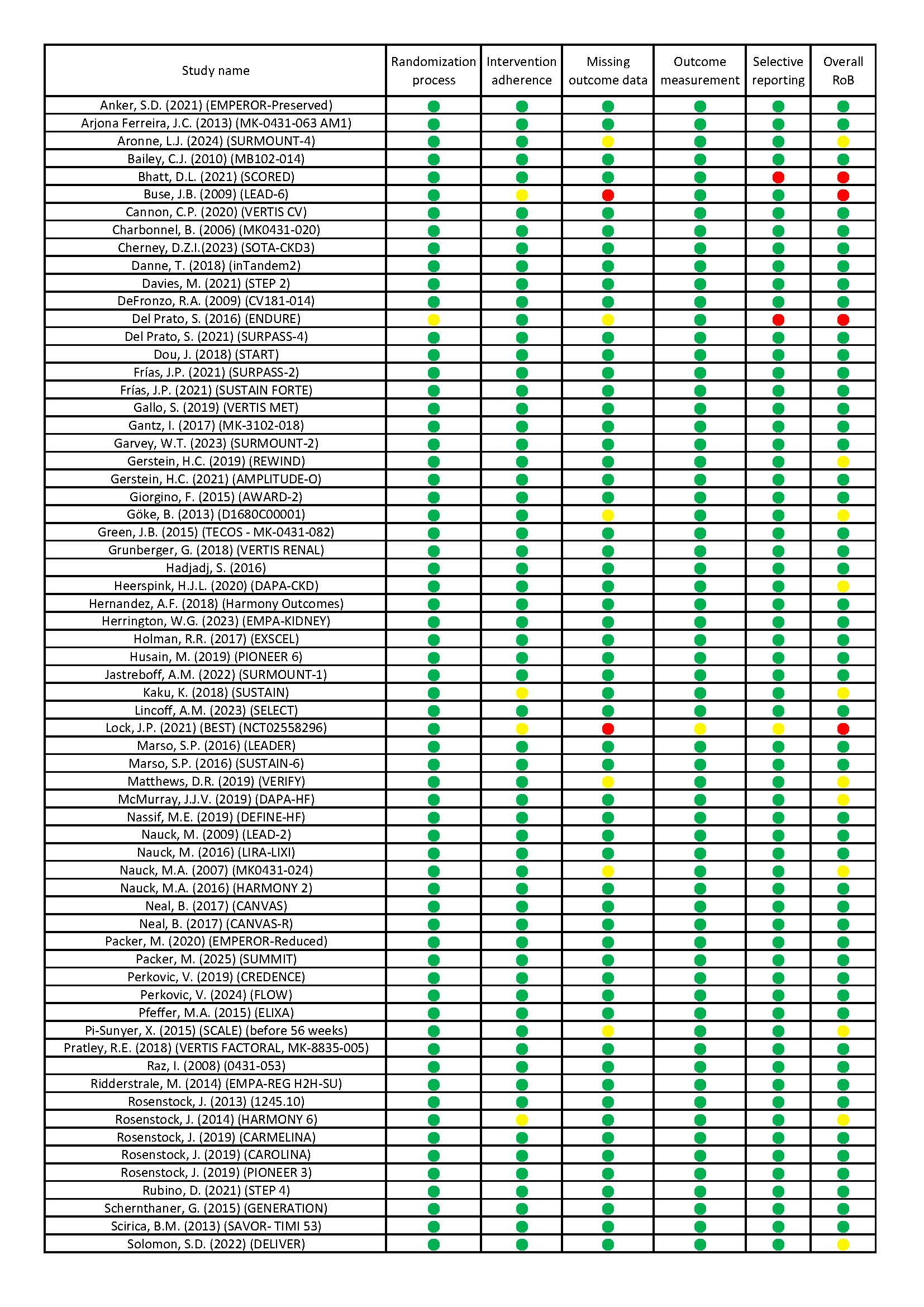
**

**
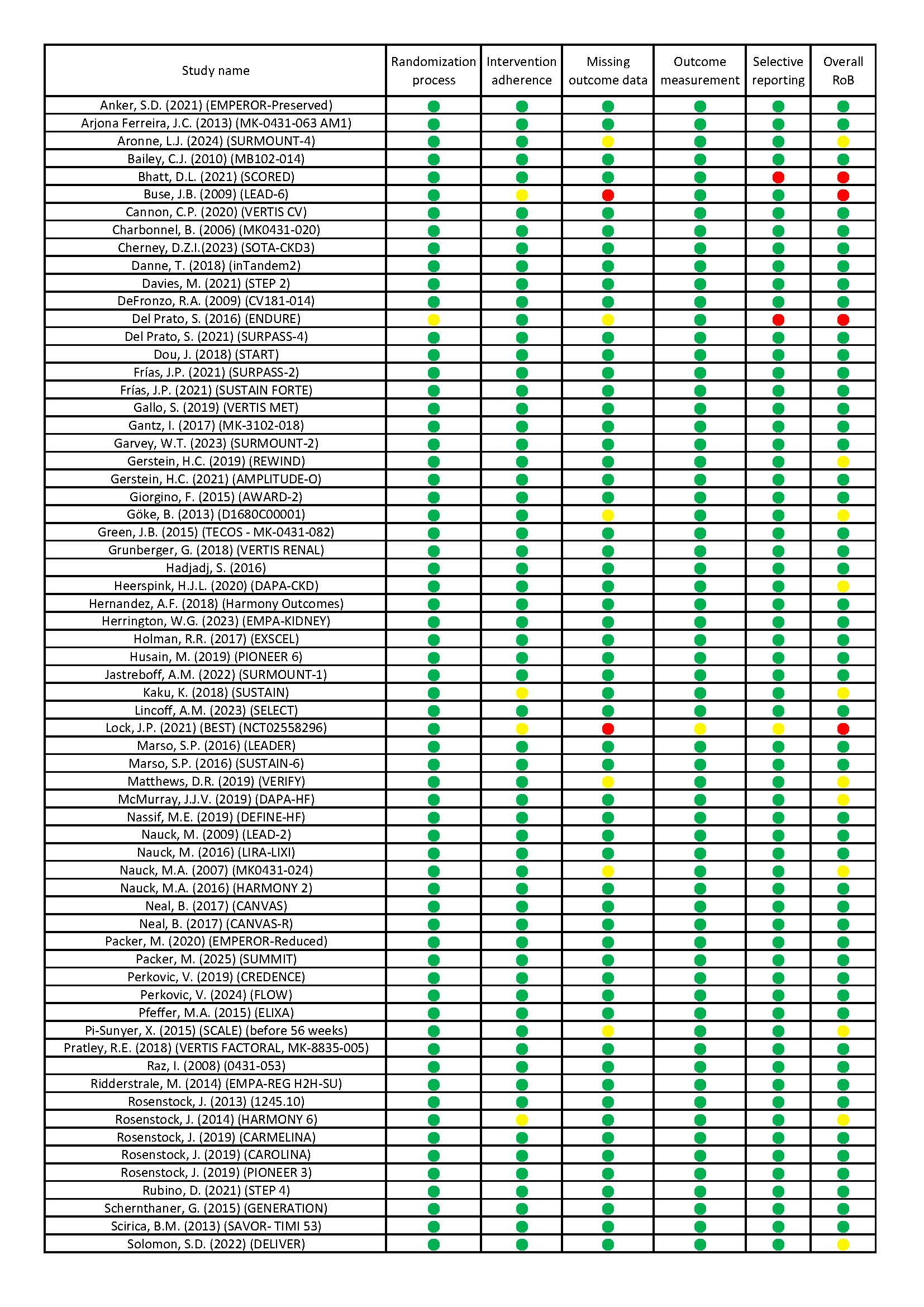
**

**
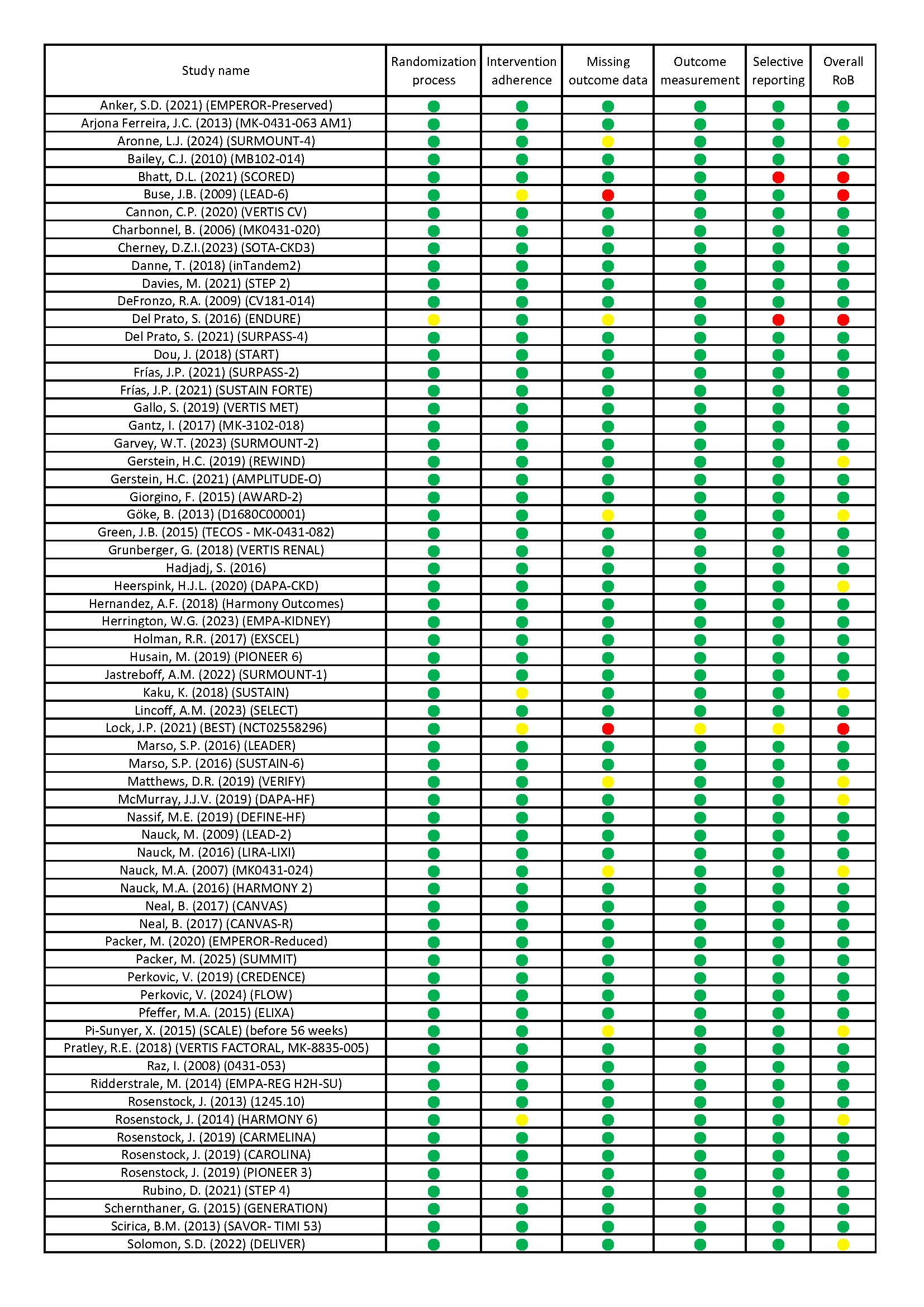
**

**
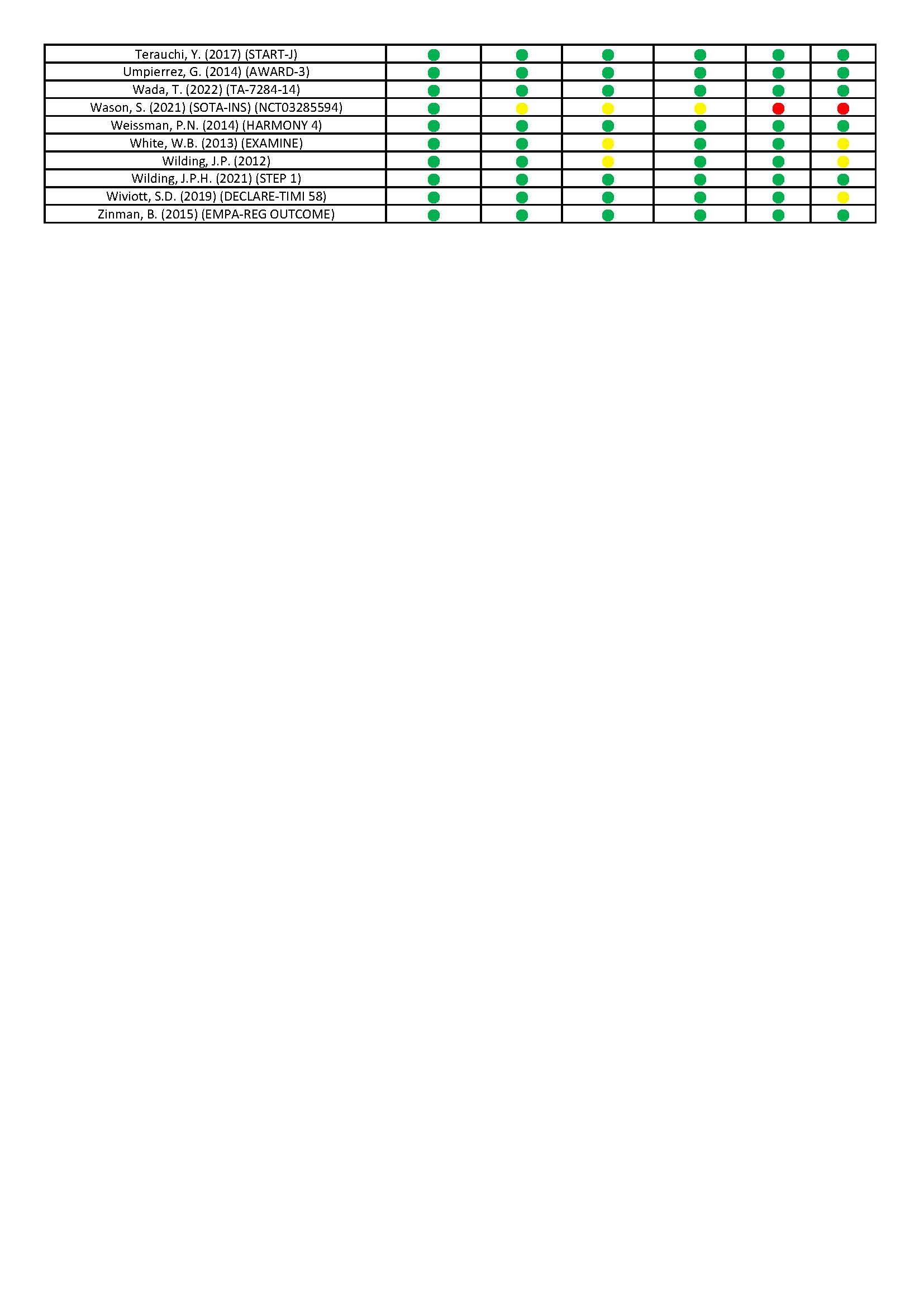
**

**eTable 1: PRISMA 2020 checklist of the current network meta-analysis**

| **Section and Topic** | **Item #** | **Checklist item** | **Page where item is reported** |
| --- | --- | --- | --- |
| **TITLE** | | |  |
| Title | 1 | Identify the report as a systematic review. | 1 |
| **ABSTRACT** | | |  |
| Abstract | 2 | See the PRISMA 2020 for Abstracts checklist. | 7 |
| **INTRODUCTION** | | |  |
| Rationale | 3 | Describe the rationale for the review in the context of existing knowledge. | 9 |
| Objectives | 4 | Provide an explicit statement of the objective(s) or question(s) the review addresses. | 9 |
| **METHODS** | | |  |
| Eligibility criteria | 5 | Specify the inclusion and exclusion criteria for the review and how studies were grouped for the syntheses. | Supplement method |
| Information sources | 6 | Specify all databases, registers, websites, organisations, reference lists and other sources searched or consulted to identify studies. Specify the date when each source was last searched or consulted. | Supplement method |
| Search strategy | 7 | Present the full search strategies for all databases, registers and websites, including any filters and limits used. | Supplement method |
| Selection process | 8 | Specify the methods used to decide whether a study met the inclusion criteria of the review, including how many reviewers screened each record and each report retrieved, whether they worked independently, and if applicable, details of automation tools used in the process. | Supplement method |
| Data collection process | 9 | Specify the methods used to collect data from reports, including how many reviewers collected data from each report, whether they worked independently, any processes for obtaining or confirming data from study investigators, and if applicable, details of automation tools used in the process. | Supplement method |
| Data items | 10a | List and define all outcomes for which data were sought. Specify whether all results that were compatible with each outcome domain in each study were sought (e.g. for all measures, time points, analyses), and if not, the methods used to decide which results to collect. | Supplement method |
|  | 10b | List and define all other variables for which data were sought (e.g. participant and intervention characteristics, funding sources). Describe any assumptions made about any missing or unclear information. | Supplement method |
| Study risk of bias assessment | 11 | Specify the methods used to assess risk of bias in the included studies, including details of the tool(s) used, how many reviewers assessed each study and whether they worked independently, and if applicable, details of automation tools used in the process. | Supplement method |
| Effect measures | 12 | Specify for each outcome the effect measure(s) (e.g. risk ratio, mean difference) used in the synthesis or presentation of results. | Supplement method |
| Synthesis methods | 13a | Describe the processes used to decide which studies were eligible for each synthesis (e.g. tabulating the study intervention characteristics and comparing against the planned groups for each synthesis (item #5)). | Supplement method |
|  | 13b | Describe any methods required to prepare the data for presentation or synthesis, such as handling of missing summary statistics, or data conversions. | Supplement method |
|  | 13c | Describe any methods used to tabulate or visually display results of individual studies and syntheses. | Supplement method |
|  | 13d | Describe any methods used to synthesize results and provide a rationale for the choice(s). If meta-analysis was performed, describe the model(s), method(s) to identify the presence and extent of statistical heterogeneity, and software package(s) used. | Supplement method |
|  | 13e | Describe any methods used to explore possible causes of heterogeneity among study results (e.g. subgroup analysis, meta-regression). | Supplement method |
|  | 13f | Describe any sensitivity analyses conducted to assess robustness of the synthesized results. | Supplement method |
| Reporting bias assessment | 14 | Describe any methods used to assess risk of bias due to missing results in a synthesis (arising from reporting biases). | Supplement method |
| Certainty assessment | 15 | Describe any methods used to assess certainty (or confidence) in the body of evidence for an outcome. | Supplement method |
| **RESULTS** | | |  |
| Study selection | 16a | Describe the results of the search and selection process, from the number of records identified in the search to the number of studies included in the review, ideally using a flow diagram. | Supplement result |
|  | 16b | Cite studies that might appear to meet the inclusion criteria, but which were excluded, and explain why they were excluded. | Supplement result |
| Study characteristics | 17 | Cite each included study and present its characteristics. | Supplement result |
| Risk of bias in studies | 18 | Present assessments of risk of bias for each included study. | Supplement result |
| Results of individual studies | 19 | For all outcomes, present, for each study: (a) summary statistics for each group (where appropriate) and (b) an effect estimate and its precision (e.g. confidence/credible interval), ideally using structured tables or plots. | Supplement result |
| Results of syntheses | 20a | For each synthesis, briefly summarise the characteristics and risk of bias among contributing studies. | Supplement result |
|  | 20b | Present results of all statistical syntheses conducted. If meta-analysis was done, present for each the summary estimate and its precision (e.g. confidence/credible interval) and measures of statistical heterogeneity. If comparing groups, describe the direction of the effect. | Supplement result |
|  | 20c | Present results of all investigations of possible causes of heterogeneity among study results. | Supplement result |
|  | 20d | Present results of all sensitivity analyses conducted to assess the robustness of the synthesized results. | Supplement result |
| Reporting biases | 21 | Present assessments of risk of bias due to missing results (arising from reporting biases) for each synthesis assessed. | Supplement result |
| Certainty of evidence | 22 | Present assessments of certainty (or confidence) in the body of evidence for each outcome assessed. | Supplement result |
| **DISCUSSION** | | |  |
| Discussion | 23a | Provide a general interpretation of the results in the context of other evidence. | 10-11 |
|  | 23b | Discuss any limitations of the evidence included in the review. | 10-11 |
|  | 23c | Discuss any limitations of the review processes used. | 11 |
|  | 23d | Discuss implications of the results for practice, policy, and future research. | 11 |
| **OTHER INFORMATION** | | |  |
| Registration and protocol | 24a | Provide registration information for the review, including register name and registration number, or state that the review was not registered. | Supplement method |
|  | 24b | Indicate where the review protocol can be accessed, or state that a protocol was not prepared. | Supplement method |
|  | 24c | Describe and explain any amendments to information provided at registration or in the protocol. | Supplement method |
| Support | 25 | Describe sources of financial or non-financial support for the review, and the role of the funders or sponsors in the review. | Supplement method |
| Competing interests | 26 | Declare any competing interests of review authors. | Supplement method |
| Availability of data, code and other materials | 27 | Report which of the following are publicly available and where they can be found: template data collection forms; data extracted from included studies; data used for all analyses; analytic code; any other materials used in the review. | Supplement method |

The current checklist followed the latest PRISMA 2020 guideline [100].

**eTable 2: Keyword used in each database and search results**

| Database | Keyword | Filter | Date | Result |
| --- | --- | --- | --- | --- |
| PubMed | (leukemia OR leukaemia OR lymphoma OR myeloma) AND (glucagon-like peptide-1 receptor agonist OR Sodium Glucose Cotransporter 2 Inhibitor OR lixisenatide OR orforglipron OR exenatide OR semaglutide OR liraglutide OR albiglutide OR dulaglutide OR tirzepatide OR bexagliflozin OR canagliflozin OR dapagliflozin OR empagliflozin OR ertugliflozin OR ipragliflozin OR luseogliflozin OR remogliflozin OR sergliflozin OR sotagliflozin OR tofogliflozin OR henagliflozin OR janagliflozin OR mizagliflozin OR velagliflozin OR enavogliflozin OR licogliflozin OR rongliflozin OR sitagliptin OR vildagliptin OR saxagliptin OR linagliptin OR gemigliptin OR anagliptin OR teneligliptin OR alogliptin OR trelagliptin OR omarigliptin OR evogliptin OR gosogliptin OR dutogliptin OR neogliptin OR retagliptin OR denagliptin OR cofrogliptin OR fotagliptin OR prusogliptin OR cetagliptin OR berberine OR retatrutide OR imeglimin OR dorzagliatin OR petrelintide OR teplizumab) AND (random OR randomized OR randomised) | N/A | 2025/09/19 | 11 |
| ClinicalKey | (leukemia OR leukaemia OR lymphoma OR myeloma) AND (glucagon-like peptide-1 receptor agonist OR Sodium Glucose Cotransporter 2 Inhibitor OR lixisenatide OR orforglipron OR exenatide OR semaglutide OR liraglutide OR albiglutide OR dulaglutide OR tirzepatide OR bexagliflozin OR canagliflozin OR dapagliflozin OR empagliflozin OR ertugliflozin OR ipragliflozin OR luseogliflozin OR remogliflozin OR sergliflozin OR sotagliflozin OR tofogliflozin OR henagliflozin OR janagliflozin OR mizagliflozin OR velagliflozin OR enavogliflozin OR licogliflozin OR rongliflozin OR sitagliptin OR vildagliptin OR saxagliptin OR linagliptin OR gemigliptin OR anagliptin OR teneligliptin OR alogliptin OR trelagliptin OR omarigliptin OR evogliptin OR gosogliptin OR dutogliptin OR neogliptin OR retagliptin OR denagliptin OR cofrogliptin OR fotagliptin OR prusogliptin OR cetagliptin OR berberine OR retatrutide OR imeglimin OR dorzagliatin OR petrelintide OR teplizumab) AND (random OR randomized OR randomised) | N/A | 2025/09/19 | 777 |
| Cochrane CENTRAL | (leukemia OR leukaemia OR lymphoma OR myeloma) AND (glucagon-like peptide-1 receptor agonist OR Sodium Glucose Cotransporter 2 Inhibitor OR lixisenatide OR orforglipron OR exenatide OR semaglutide OR liraglutide OR albiglutide OR dulaglutide OR tirzepatide OR bexagliflozin OR canagliflozin OR dapagliflozin OR empagliflozin OR ertugliflozin OR ipragliflozin OR luseogliflozin OR remogliflozin OR sergliflozin OR sotagliflozin OR tofogliflozin OR henagliflozin OR janagliflozin OR mizagliflozin OR velagliflozin OR enavogliflozin OR licogliflozin OR rongliflozin OR sitagliptin OR vildagliptin OR saxagliptin OR linagliptin OR gemigliptin OR anagliptin OR teneligliptin OR alogliptin OR trelagliptin OR omarigliptin OR evogliptin OR gosogliptin OR dutogliptin OR neogliptin OR retagliptin OR denagliptin OR cofrogliptin OR fotagliptin OR prusogliptin OR cetagliptin OR berberine OR retatrutide OR imeglimin OR dorzagliatin OR petrelintide OR teplizumab) AND (random OR randomized OR randomised) | N/A | 2025/09/19 | 10 |
| Embase | (leukemia OR leukaemia OR lymphoma OR myeloma) AND (glucagon-like peptide-1 OR Sodium Glucose Cotransporter 2 OR mitochondrial bioenergetics modulator OR glucokinase activator OR amylin analogue OR anti-CD3 monoclonal antibody) AND (random OR randomized OR randomised) | N/A | 2025/09/19 | 56 |
| ProQuest | (leukemia OR leukaemia OR lymphoma OR myeloma) AND (glucagon-like peptide-1 OR Sodium Glucose Cotransporter 2 OR mitochondrial bioenergetics modulator OR glucokinase activator OR amylin analogue OR anti-CD3 monoclonal antibody) AND (random OR randomized OR randomised) | N/A | 2025/09/19 | 7437 |
| ScienceDirect | (leukemia OR leukaemia OR lymphoma OR myeloma) AND (glucagon-like peptide-1 receptor agonist OR Sodium Glucose Cotransporter 2 Inhibitor OR DPP-4 inhibitor) | N/A | 2025/09/19 | 6395 |
| Web of Science | (leukemia OR leukaemia OR lymphoma OR myeloma) AND (glucagon-like peptide-1 OR Sodium Glucose Cotransporter 2 OR mitochondrial bioenergetics modulator OR glucokinase activator OR amylin analogue OR anti-CD3 monoclonal antibody) AND (random OR randomized OR randomised) | N/A | 2025/09/19 | 10 |
| ClinicalTrials.gov | (leukemia OR leukaemia OR lymphoma OR myeloma) AND (glucagon-like peptide-1 receptor agonist OR Sodium Glucose Cotransporter 2 Inhibitor OR lixisenatide OR orforglipron OR exenatide OR semaglutide OR liraglutide OR albiglutide OR dulaglutide OR tirzepatide OR bexagliflozin OR canagliflozin OR dapagliflozin OR empagliflozin OR ertugliflozin OR ipragliflozin OR luseogliflozin OR remogliflozin OR sergliflozin OR sotagliflozin OR tofogliflozin OR henagliflozin OR janagliflozin OR mizagliflozin OR velagliflozin OR enavogliflozin OR licogliflozin OR rongliflozin OR sitagliptin OR vildagliptin OR saxagliptin OR linagliptin OR gemigliptin OR anagliptin OR teneligliptin OR alogliptin OR trelagliptin OR omarigliptin OR evogliptin OR gosogliptin OR dutogliptin OR neogliptin OR retagliptin OR denagliptin OR cofrogliptin OR fotagliptin OR prusogliptin OR cetagliptin OR berberine OR retatrutide OR imeglimin OR dorzagliatin OR petrelintide OR teplizumab) AND (random OR randomized OR randomised) | N/A | 2025/09/19 | 2 |

Abbreviation: N/A: not applied

**eTable 3: Dosage stratification (stratified according to the included original RCTs)**

| Medication | Low-dose | Medium-dose | High-dose |
| --- | --- | --- | --- |
| Albiglutide | 15 mg/week | 30 mg/week | 50 mg/week |
| Alogliptin | 12.5 mg/day | NA | 25 mg/day |
| Canagliflozin | 50-100 mg/day | 200 mg/day | 300-600 mg/day |
| Dapagliflozin | 2.5 mg/day | 5 mg/day | 10 mg/day |
| Dulaglutide | <1.5 mg/week | 1.5 mg/week | >1.5 mg/week |
| Efpeglenatide | 2mg/week | 4 mg/week | 6 mg/week |
| Empagliflozin | 1-10 mg/day | NA | 25-50 mg/day |
| Ertugliflozin | 5 mg/day | NA | 15 mg/day |
| Injectable semaglutide | 0.05-0.5 mg/week | 1.0-1.7 mg/week | 2.0-2.4 mg/week |
| Linagliptin | 2.5 mg/day | 5 mg/day | 10 mg/day |
| Liraglutide | 1.2 mg/day | 1.8 mg/day | 3.0 mg/day |
| Oral semaglutide | 7-10 mg/day | 14 mg/day | 20-25 mg/day |
| Saxagliptin | 2.5 mg/day | 5 mg/day | 10 mg/day |
| Sitagliptin | 25 mg/day | 50 mg/day | 100 mg/day |
| Teneligliptin | 5 mg/day | 10 mg/day | 20 mg/day |
| Tirzepatide | 1-5 mg/week | 10 mg/week | 15 mg/week |

*Abbreviation: NA: not applied; RCT: randomized controlled trial*

**eTable 4: Excluded studies and reason**

| Reason | Numbers | References |
| --- | --- | --- |
| All patients received target medication first and then shift to comparators without adequate washout period | 1 | [68] |
| Animal study | 2 | [101,102] |
| Duplicate sample source with another included trial | 1 | [103] |
| Inappropriate randomization (i.e. significantly different baseline demographic data between groups, which might affect primary outcomes, such as age, gender, comorbid diseases, or concurrent medications) | 1 | [42] |
| Meta-analysis | 1 | [104] |
| Not randomized controlled trial | 3 | [105-107] |
| Not report targeted outcome | 283 | [108-226] [227-344] [345-390] |
| Review article | 1 | [391] |
| Study protocol but not result of a study | 1 | [392] |

**eTable 5: Characteristics of the included studies**

| Study name | Baseline illness | Comparison | Subjects | Mean age (year) | Female (%) | Treatment duration | Route | Category | ClinicalTrials.gov | Country |
| --- | --- | --- | --- | --- | --- | --- | --- | --- | --- | --- |
| Packer, M. (2025) (SUMMIT)[55] | patients with obesity and heart failure | Tirzepatide 15mg/week Placebo | 364 367 | 65.5±10.5 65.0±10.9 | 54.9 52.6 | 160 weeks | injection | GLP-1 agonist | NCT04847557 | Multiple countries |
| Aronne, L.J. (2024) (SURMOUNT-4)[11] | patients with obesity | Tirzepatide 10-15mg/week Placebo | 335 335 | 49.0±13.0 48.0±12.0 | 70.4 70.7 | 36 weeks | injection | GLP-1 agonist | NCT04660643 | Multiple countries |
| Perkovic, V. (2024) (FLOW)[57] | patients with type 2 diabetes mellitus and chronic renal insufficiency | Inject semaglutide 1.0 mg/week Placebo | 1767 1766 | 66.6±9.0 66.7±9.0 | 29.4 31.1 | 208 weeks | injection | GLP-1 agonist | NCT03819153 | Multiple countries |
| Cherney, D.Z.I.(2023) (SOTA-CKD3)[17] | patients with type 2 diabetes mellitus and chronic kidney disease | Sotagliflozin Placebo | 527 260 | 69.5±7.9 69.3±8.1 | 44.0 42.7 | 26 weeks | oral | SGLT2 inhibitor | NCT03242252 | Multiple countries |
| Garvey, W.T. (2023) (SURMOUNT-2)[28] | patients with type 2 diabetes mellitus and obesity | Tirzepatide 10mg/week Tirzepatide 15mg/week Placebo | 312 311 315 | 54.3±10.7 53.6±10.6 54.7±10.5 | 50.6 51.1 50.5 | 72 weeks | injection | GLP-1 agonist | NCT04657003 | Multiple countries |
| Herrington, W.G. (2023) (EMPA-KIDNEY)[73] | patients with renal failure | Empagliflozin 10mg/day Placebo | 3304 3305 | 63.9±13.9 63.8±13.9 | 33.2 33.1 | 104 weeks | oral | SGLT2 inhibitor | NCT03594110 | Multiple countries |
| Lincoff, A.M. (2023) (SELECT)[42] | patients with obesity | Inject Semaglutide 2.4 mg/week Placebo | 8803 8801 | 61.6±8.9 61.6±8.8 | 27.8 27.5 | 104 weeks | injection | GLP-1 agonist | NCT03574597 | Multiple countries |
| Jastreboff, A.M. (2022) (SURMOUNT-1)[40] | patients with obesity | Tirzepatide 5mg/week Tirzepatide 10mg/week Tirzepatide 15mg/week Placebo | 630 636 630 643 | 45.6±12.7 44.7±12.4 44.9±12.3 44.4±12.5 | 67.6 67.1 67.5 67.8 | 72 weeks | injection | GLP-1 agonist | NCT04184622 | Multiple countries |
| Solomon, S.D. (2022) (DELIVER)[71] | patients with stabilized heart failure | Dapagliflozin 10mg/day Placebo | 3131 3132 | 71.8±9.6 71.5±9.5 | 43.6 44.2 | 120 weeks | oral | SGLT2 inhibitor | NCT03619213 | Multiple countries |
| Wada, T. (2022) (TA-7284-14)[75] | patients with type 2 diabetes mellitus and chronic kidney disease | Canagliflozin 100 mg/day Placebo | 154 154 | 62.5±10.5 62.4±11.1 | 25.3 16.2 | 104 weeks | oral | SGLT2 inhibitor | NCT03436693 | Japan |
| Anker, S.D. (2021) (EMPEROR-Preserved)[9] | patients with heart failure with preserved ejection fraction | Empagliflozin 10mg/day Placebo | 2997 2991 | 71.8±9.3 71.9±9.6 | 44.6 44.7 | 156 weeks | oral | SGLT2 inhibitor | NCT03057951 | Multiple countries |
| Bhatt, D.L. (2021) (SCORED)[13] | patients with type 2 diabetes mellitus and chronic kidney disease | Sotagliflozin 200-400mg/day Placebo | 5292 5292 | 68.4±8.4 68.2±8.4 | 44.3 45.5 | 116 weeks | oral | SGLT2 inhibitor | NCT03315143 | Multiple countries |
| Davies, M. (2021) (STEP 2)[19] | patients with type 2 diabetes mellitus and obesity | Inject semaglutide 1.0 mg/week Inject Semaglutide 2.4 mg/week Placebo | 403 404 403 | 56.0±10.0 55.0±11.0 55.0±11.0 | 50.4 55.2 47.1 | 68 weeks | injection | GLP-1 agonist | NCT03552757 | Multiple countries |
| Del Prato, S. (2021) (SURPASS-4)[22] | patients with type 2 diabetes mellitus | Tirzepatide 5mg/week Tirzepatide 10mg/week Tirzepatide 15mg/week Control | 329 328 338 1000 | 62.9±8.6 63.7±8.7 63.7±8.6 63.8±8.5 | 39.8 36.3 39.9 36.4 | 108 weeks | injection | GLP-1 agonist | NCT03730662 | Multiple countries |
| Frías, J.P. (2021) (SURPASS-2)[25] | patients with type 2 diabetes mellitus | Tirzepatide 5mg/week Tirzepatide 10mg/week Tirzepatide 15mg/week Inject semaglutide 1.0 mg/week | 470 469 470 469 | 56.3±10.0 57.2±10.5 55.9±10.4 56.9±10.8 | 56.4 49.3 54.5 52.0 | 40 weeks | injection | GLP-1 agonist | NCT03987919 | Multiple countries |
| Frías, J.P. (2021) (SUSTAIN FORTE)[24] | patients with type 2 diabetes mellitus | Inject semaglutide 1.0 mg/week Inject semaglutide 2.0 mg/week | 481 480 | 58.2±9.9 57.9±10.0 | 41.0 41.9 | 40 weeks | injection | GLP-1 agonist | NCT03989232 | Multiple countries |
| Gerstein, H.C. (2021) (AMPLITUDE-O)[30] | patients with type 2 diabetes mellitus | Efpeglenatide 4 mg/week Efpeglenatide 6 mg/week Placebo | 1359 1358 1359 | 64.6±8.2 64.7±8.2 64.4±8.3 | 32.5 35.6 30.8 | 104 weeks | injection | GLP-1 agonist | NCT03496298 | Multiple countries |
| Lock, J.P. (2021) (BEST) (NCT02558296)[43] | patients with type 2 diabetes mellitus | Bexagliflozin 20mg/day Placebo | 1132 567 | 64.4±7.9 64.6±8.0 | 30.1 31.2 | 52 weeks | oral | SGLT2 inhibitor | NCT02558296 | Multiple countries |
| Rubino, D. (2021) (STEP 4)[68] | patients with overweight or obesity | Inject semaglutide 2.4 mg/week Placebo | 535 268 | 47.0±12.0 46.0±12.0 | 80.2 76.5 | 68 weeks | injection | GLP-1 agonist | NCT03548987 | Multiple countries |
| Wason, S. (2021) (SOTA-INS) (NCT03285594)[76] | patients with type 2 diabetes mellitus | Sotagliflozin 200-400mg/day Placebo | 427 144 | 62.5±9.5 62.2±8.9 | 46.2 40.3 | 52 weeks | oral | SGLT2 inhibitor | NCT03285594 | Multiple countries |
| Wilding, J.P.H. (2021) (STEP 1)[80] | patients with obesity | Inject semaglutide 2.4 mg/week Placebo | 1306 655 | 46.0±13.0 47.0±12.0 | 73.1 76.0 | 68 weeks | injection | GLP-1 agonist | NCT03548935 | Multiple countries |
| Cannon, C.P. (2020) (VERTIS CV)[15] | patients with type 2 diabetes mellitus | Ertugliflozin 5 mg/day Ertugliflozin 15 mg/day Placebo | 2752 2747 2747 | 64.3±8.2 64.4±8.0 64.4±8.0 | 29.1 30.3 30.7 | 182 weeks | oral | SGLT2 inhibitor | NCT01986881 | Multiple countries |
| Heerspink, H.J.L. (2020) (DAPA-CKD)[36] | patients with renal failure | Dapagliflozin 10mg/day Placebo | 2152 2152 | 61.8±12.1 61.9±12.1 | 32.9 33.3 | 125 weeks | oral | SGLT2 inhibitor | NCT03036150 | Multiple countries |
| Packer, M. (2020) (EMPEROR-Reduced)[54] | patients with chronic heart failure | Empagliflozin 10mg/day Placebo | 1863 1867 | 67.2±10.8 66.5±11.2 | 23.5 24.4 | 64 weeks | oral | SGLT2 inhibitor | NCT03057977 | Multiple countries |
| Gallo, S. (2019) (VERTIS MET)[26] | patients with type 2 diabetes mellitus | Ertugliflozin 5 mg/day Ertugliflozin 15 mg/day Placebo | 207 205 209 | 56.6±8.2 56.9±9.4 56.5±8.7 | 53.1 54.6 53.1 | 104 weeks | oral | SGLT2 inhibitor | NCT02033889 | Multiple countries |
| Gerstein, H.C. (2019) (REWIND)[29] | patients with type 2 diabetes mellitus | Dulaglutide 1.5 mg/week Placebo | 4949 4952 | 66.2±6.5 66.2±6.5 | 46.6 46.1 | 281 weeks | injection | GLP-1 agonist | NCT01394952 | Multiple countries |
| Husain, M. (2019) (PIONEER 6)[39] | patients withtype 2 diabetes mellitus and cardiovascular disease or chronic kidney disease | Oral semaglutide 14mg/day Placebo | 1591 1592 | 66.0±7.0 66.0±7.0 | 31.9 31.4 | 64 weeks | oral | GLP-1 agonist | NCT02692716 | Multiple countries |
| Matthews, D.R. (2019) (VERIFY)[46] | patients with type 2 diabetes mellitus | Vildagliptin 100mg/day Placebo | 998 1003 | 54.1±9.5 54.6±9.2 | 54.6 51.3 | 260 weeks | oral | DPP4 inhibitor | NCT01528254 | Multiple countries |
| McMurray, J.J.V. (2019) (DAPA-HF)[47] | patients with stabilized heart failure | Dapagliflozin 10mg/day Placebo | 2373 2371 | 66.2±11.0 66.5±10.8 | 23.8 23.0 | 73 weeks | oral | SGLT2 inhibitor | NCT03036124 | Multiple countries |
| Nassif, M.E. (2019) (DEFINE-HF)[48] | patients with heart failure | Dapagliflozin 10mg/day Placebo | 131 132 | 62.2±11.0 60.4±12.0 | 27.5 25.8 | 12 weeks | oral | SGLT2 inhibitor | NCT02653482 | USA |
| Perkovic, V. (2019) (CREDENCE)[56] | patients with type 2 diabetes mellitus and nephropathy | Canagliflozin 100 mg/day Placebo | 2202 2199 | 62.9±9.2 63.2±9.2 | 34.6 33.3 | 130 weeks | oral | SGLT2 inhibitor | NCT02065791 | Multiple countries |
| Rosenstock, J. (2019) (CARMELINA)[66] | patients with type 2 diabetes mellitus | Linagliptin 5 mg/day Placebo | 3494 3485 | 66.1±9.1 65.6±9.1 | 38.5 35.7 | 182 weeks | oral | DPP4 inhibitor | NCT01897532 | Multiple countries |
| Rosenstock, J. (2019) (CAROLINA)[65] | patients with type 2 diabetes mellitus | Linagliptin 5 mg/day Placebo | 3023 3010 | 63.9±9.5 64.2±9.5 | 39.2 40.8 | 338 weeks | oral | DPP4 inhibitor | NCT01243424 | Multiple countries |
| Rosenstock, J. (2019) (PIONEER 3)[63] | patients with type 2 diabetes mellitus | Oral semaglutide 3 mg/day Oral semaglutide 7 mg/day Oral semaglutide 14 mg/day Sitagliptin 100 mg/day | 466 465 465 467 | 58.0±10.0 58.0±10.0 57.0±10.0 58.0±10.0 | 45.5 47.3 46.9 49.0 | 78 weeks | oral | GLP-1 agonist | NCT02607865 | Multiple countries |
| Wiviott, S.D. (2019) (DECLARE-TIMI 58)[81] | patients with type 2 diabetes mellitus and atherosclerotic vascular disease | Dapagliflozin 10mg/day Placebo | 8582 8578 | 63.9±6.8 64.0±6.8 | 36.9 37.9 | 206 weeks | oral | SGLT2 inhibitor | NCT01730534 | Multiple countries |
| Danne, T. (2018) (inTandem2)[18] | patients with type 1 diabetes mellitus | Sotagliflozin 200-400mg/day Placebo | 524 258 | 42.0±13.4 39.7±13.4 | 48.1 48.1 | 52 weeks | oral | SGLT2 inhibitor | NCT02421510 | Multiple countries |
| Dou, J. (2018) (START)[23] | patients with type 2 diabetes mellitus | Saxagliptin 5mg/day Placebo | 210 207 | 50.8±10.4 50.1±11.0 | 35.2 36.2 | 24 weeks | oral | DPP4 inhibitor | NCT02273050 | China |
| Grunberger, G. (2018) (VERTIS RENAL)[34] | patients with type 2 diabetes mellitus with chronic kidney disease | Ertugliflozin 5 mg/day Ertugliflozin 15 mg/day Placebo | 158 155 154 | 66.7±8.3 67.5±8.5 67.5±8.9 | 46.8 51.6 53.2 | 54 weeks | oral | SGLT2 inhibitor | NCT01986855 | Multiple countries |
| Hernandez, A.F. (2018) (Harmony Outcomes)[37] | patients with type 2 diabetes mellitus | Albiglutide 50 mg/week Placebo | 4731 4732 | 64.1±8.7 64.2±8.7 | 30.2 31.0 | 86 weeks | injection | GLP-1 agonist | NCT02465515 | Multiple countries |
| Kaku, K. (2018) (SUSTAIN)[41] | patients with type 2 diabetes mellitus | Inject semaglutide 0.5 mg/week Inject semaglutide 1.0 mg/week Control | 239 241 121 | 58.0±10.6 58.7±10.2 59.2±10.1 | 30.5 27.8 25.8 | 61 weeks | injection | GLP-1 agonist | NCT02207374 | Multiple countries |
| Pratley, R.E. (2018) (VERTIS FACTORAL, MK-8835-005)[60] | patients with type 2 diabetes mellitus | Ertugliflozin 5 mg/day Ertugliflozin 15 mg/day Sitagliptin 100 mg/day Ertugliflozin 5 mg/day + Sitagliptin 100 mg/day Ertugliflozin 15 mg/day + Sitagliptin 100 mg/day | 250 248 247 243 244 | 55.1±10.1 55.3±9.5 54.8±10.7 55.2±10.4 55.1±9.8 | 49.2 46.0 37.7 49.4 48.4 | 52 weeks | oral | DPP4 inhibitor/SGLT2 inhibitor | NCT02099110 | Multiple countries |
| Gantz, I. (2017) (MK-3102-018)[27] | patients with type 2 diabetes mellitus and cardiovascular disease | Omarigliptin 25 mg/week Placebo | 2100 2102 | 63.7±8.5 63.6±8.5 | 30.4 29.3 | 156 weeks | oral | DPP4 inhibitor | NCT01703208 | Multiple countries |
| Holman, R.R. (2017) (EXSCEL)[38] | patients with type 2 diabetes mellitus | Exenatide 2mg/day Placebo | 7356 7396 | 61.8±9.4 61.9±9.4 | 38.0 38.0 | 166 weeks | injection | GLP-1 agonist | NCT01144338 | Multiple countries |
| Neal, B. (2017) (CANVAS)[53] | patients with type 2 diabetes mellitus | Canagliflozin 100 mg/day Canagliflozin 300 mg/day Placebo | 1445 1443 1442 | 62.2±8.0 62.8±8.1 62.3±7.9 | 33.5 34.6 33.7 | 126 weeks | oral | SGLT2 inhibitor | NCT01032629 | Multiple countries |
| Neal, B. (2017) (CANVAS-R)[53] | patients with type 2 diabetes mellitus | Canagliflozin 300 mg/day Placebo | 2907 2905 | 63.9±8.4 64.0±8.3 | 36.2 38.2 | 126 weeks | oral | SGLT2 inhibitor | NCT01989754 | Multiple countries |
| Terauchi, Y. (2017) (START-J)[72] | patients with type 2 diabetes mellitus | Sitagliptin 100 mg/day Control | 143 129 | 70.2±5.4 70.8±5.5 | 48.3 38.8 | 52 weeks | oral | DPP4 inhibitor | NCT01183104 | Japan |
| Del Prato, S. (2016) (ENDURE)[21] | patients with type 2 diabetes mellitus | Alogliptin 12.5 mg/day Alogliptin 25 mg/day Placebo | 880 885 874 | 55.2±9.6 55.5±9.8 55.4±9.6 | 52.4 48.9 49.5 | 104 weeks | oral | DPP4 inhibitor | NCT00856284 | Multiple countries |
| Hadjadj, S. (2016)[35] | patients with type 2 diabetes mellitus | Empagliflozin 10mg/day Empagliflozin 25mg/day Placebo | 497 551 332 | 52.5±11.2 52.4±10.7 52.5±10.9 | 41.1 42.5 46.4 | 24 weeks | oral | SGLT2 inhibitor | NCT01719003 | Multiple countries |
| Marso, S.P. (2016) (LEADER)[45] | patients with type 2 diabetes mellitus | Liraglutide 1.8mg/day Placebo | 4668 4672 | 64.2±7.2 64.4±7.2 | 35.5 36.0 | 198 weeks | injection | GLP-1 agonist | NCT01179048 | Multiple countries |
| Marso, S.P. (2016) (SUSTAIN-6)[44] | patients with type 2 diabetes mellitus | Inject semaglutide 0.5 mg/week Inject semaglutide 1.0 mg/week Placebo | 826 822 1649 | NA | 40.1 37.0 40.0 | 109 weeks | injection | GLP-1 agonist | NCT01720446 | Multiple countries |
| Nauck, M. (2016) (LIRA-LIXI)[50] | patients with type 2 diabetes mellitus | Liraglutide 1.8mg/day Lixisenatide 20ug/day | 202 202 | 56.3±10.6 56.1±10.0 | 35.0 45.0 | 26 weeks | injection | GLP-1 agonist | NCT01973231 | Multiple countries |
| Nauck, M.A. (2016) (HARMONY 2)[52] | patients with type 2 diabetes mellitus | Albiglutide 30 mg/week Albiglutide 50 mg/week Placebo | 101 99 101 | 53.6±10.9 52.0±11.8 53.1±11.7 | 42.6 49.5 42.6 | 52 weeks | injection | GLP-1 agonist | NCT00849017 | Multiple countries |
| Giorgino, F. (2015) (AWARD-2)[31] | patients with type 2 diabetes mellitus | Dulaglutide 0.75 mg/week Dulaglutide 1.5 mg/week Control | 272 273 262 | 56.6±9.3 56.2±9.8 57.0±9.0 | 50.0 47.3 48.9 | 78 weeks | injection | GLP-1 agonist | NCT01075282 | Multiple countries |
| Green, J.B. (2015) (TECOS - MK-0431-082)[33] | patients with type 2 diabetes mellitus | Sitagliptin 100 mg/day Placebo | 7332 7339 | 65.4±7.9 65.5±8.0 | 29.1 29.5 | 192 weeks | oral | DPP4 inhibitor | NCT00790205 | Multiple countries |
| Pfeffer, M.A. (2015) (ELIXA)[58] | patients with type 2 diabetes mellitus and recent acute coronary syndrome | Lixisenatide 20ug/day Placebo | 3034 3034 | 59.9±9.7 60.6±9.6 | 30.4 30.9 | 100 weeks | injection | GLP-1 agonist | NCT01147250 | Multiple countries |
| Pi-Sunyer, X. (2015) (SCALE) (before 56 weeks)[59] | patients with obesity | Liraglutide 3.0mg/day Placebo | 2487 1244 | 45.2±12.1 45.0±12.0 | 78.7 78.1 | 56 weeks | injection | GLP-1 agonist | NCT01272219 | Multiple countries |
| Schernthaner, G. (2015) (GENERATION)[69] | patients with type 2 diabetes mellitus | Saxagliptin 5mg/day Placebo | 360 360 | 72.5±5.7 72.7±5.4 | 39.7 36.7 | 52 weeks | oral | DPP4 inhibitor | NCT01006603 | Multiple countries |
| Zinman, B. (2015) (EMPA-REG OUTCOME)[82] | patients with type 2 diabetes mellitus | Empagliflozin 10mg/day Empagliflozin 25mg/day Placebo | 2345 2342 2333 | 63.0±8.6 63.2±8.6 63.2±8.8 | 29.5 28.1 28.0 | 135 weeks | oral | SGLT2 inhibitor | NCT01131676 | Multiple countries |
| Ridderstrale, M. (2014) (EMPA-REG H2H-SU)[62] | patients with type 2 diabetes mellitus and moderate-to-severe chronic kidney disease | Empagliflozin 25mg/day Control | 765 780 | 56.2±10.3 55.7±10.4 | 43.5 46.0 | 104 weeks | oral | SGLT2 inhibitor | NCT01167881 | Multiple countries |
| Rosenstock, J. (2014) (HARMONY 6)[64] | patients with type 2 diabetes mellitus | Albiglutide 50 mg/week Control | 285 281 | 54.8±9.1 56.3±8.9 | 53.7 51.6 | 52 weeks | injection | GLP-1 agonist | NCT00976391 | Multiple countries |
| Umpierrez, G. (2014) (AWARD-3)[74] | patients with type 2 diabetes mellitus | Dulaglutide 0.75 mg/week Dulaglutide 1.5 mg/week Control | 270 269 268 | 55.9±10.7 55.5±10.4 55.0±10.0 | 56.3 57.6 54.9 | 52 weeks | injection | GLP-1 agonist | NCT01126580 | Multiple countries |
| Weissman, P.N. (2014) (HARMONY 4)[77] | patients with type 2 diabetes mellitus | Albiglutide 30 mg/week Control | 504 241 | 55.8±9.3 54.7±9.8 | 43.3 45.2 | 52 weeks | injection | GLP-1 agonist | NCT00838916 | Multiple countries |
| Arjona Ferreira, J.C. (2013) (MK-0431-063 AM1)[10] | patients with type 2 diabetes mellitus and chronic renal insufficiency | Sitagliptin 50 mg/day Placebo | 135 142 | 64.8±10.6 64.3±9.2 | 40.7 45.1 | 54 weeks | oral | DPP4 inhibitor | NCT00509262 | Multiple countries |
| Göke, B. (2013) (D1680C00001)[32] | patients with type 2 diabetes mellitus | Saxagliptin 5mg/day Placebo | 428 430 | 57.5 57.6 | 50.5 46.0 | 104 weeks | oral | DPP4 inhibitor | NCT00575588 | Multiple countries |
| Rosenstock, J. (2013) (1245.10)[67] | patients with type 2 diabetes mellitus | Empagliflozin 1-10mg/day Empagliflozin 25-50mg/day Placebo Sitagliptin 100 mg/day | 213 140 71 71 | 58.7±8.5 57.3±8.9 59.7±8.5 | 51.7 45.7 53.5 | 12 weeks | oral | SGLT2 inhibitor | NCT00749190 | Multiple countries |
| Scirica, B.M. (2013) (SAVOR- TIMI 53)[70] | patients with type 2 diabetes mellitus | Saxagliptin 5mg/day Placebo | 8280 8212 | 65.1±8.5 65.0±8.6 | 33.4 32.7 | 128 weeks | oral | DPP4 inhibitor | NCT01107886 | Multiple countries |
| White, W.B. (2013) (EXAMINE)[78] | patients with type 2 diabetes mellitus | Alogliptin 25 mg/day Placebo | 2701 2679 | 61.0 61.0 | 32.3 32.0 | 52 weeks | oral | DPP4 inhibitor | NCT00968708 | Multiple countries |
| Wilding, J.P. (2012)[79] | patients with type 2 diabetes mellitus and moderate-to-severe chronic kidney disease | Dapagliflozin 2.5 mg/day Dapagliflozin 5 mg/day Dapagliflozin 10 mg/day Placebo | 202 211 194 193 | 59.8±7.6 59.3±7.9 59.3±8.8 58.8±8.6 | 50.5 52.6 55.2 50.8 | 104 weeks | oral | SGLT2 inhibitor | NCT00673231 | Multiple countries |
| Bailey, C.J. (2010) (MB102-014)[12] | patients with type 2 diabetes mellitus | Dapagliflozin 2.5 mg/day Dapagliflozin 5 mg/day Dapagliflozin 10 mg/day Placebo | 137 137 135 137 | 55.0±9.3 54.3±9.4 52.7±9.9 53.7±10.3 | 48.9 49.6 43.0 44.5 | 24 weeks | oral | SGLT2 inhibitor | NCT00528879 | Multiple countries |
| Buse, J.B. (2009) (LEAD-6)[14] | patients with type 2 diabetes mellitus and moderate-to-severe chronic kidney disease | Liraglutide 1.8 mg/day Exenatide | 233 231 | 56.3±9.8 57.1±10.8 | 51.1 45.0 | 26 weeks | injection | GLP-1 agonist | NCT00518882 | Multiple countries |
| DeFronzo, R.A. (2009) (CV181-014)[20] | patients with type 2 diabetes mellitus | Saxagliptin 2.5mg/day Saxagliptin 5mg/day Saxagliptin 10mg/day Placebo | 192 191 181 179 | 54.7±10.1 54.7±9.6 54.2±10.1 54.8±10.2 | 56.8 46.1 47.5 46.4 | 24 weeks | oral | DPP4 inhibitor | NCT00121667 | Multiple countries |
| Nauck, M. (2009) (LEAD-2)[49] | patients with type 2 diabetes mellitus | Liraglutide 0.6 mg/day Liraglutide 1.2 mg/day Liraglutide 1.8 mg/day Placebo | 242 240 242 121 | 56.0±10.5 57.2±9.2 56.8±9.4 56.0±9.0 | 37.6 46.3 41.3 40.0 | 26 weeks | injection | GLP-1 agonist | NCT00318461 | Multiple countries |
| Raz, I. (2008) (0431-053)[61] | patients with type 2 diabetes mellitus | Sitagliptin 100 mg/day Placebo | 96 94 | 53.6±9.5 56.1±9.5 | 49.0 58.5 | 30 weeks | oral | DPP4 inhibitor | NCT00337610 | Multiple countries |
| Nauck, M.A. (2007) (MK0431-024)[51] | patients with type 2 diabetes mellitus | Sitagliptin 100 mg/day Placebo | 588 584 | 56.8±9.3 56.6±9.8 | 42.9 38.7 | 52 weeks | oral | DPP4 inhibitor | NCT00094770 | Multiple countries |
| Charbonnel, B. (2006) (MK0431-020)[16] | patients with type 2 diabetes mellitus | Sitagliptin 100 mg/day Placebo | 464 237 | 54.4±10.4 54.7±9.7 | 44.2 40.5 | 24 weeks | oral | DPP4 inhibitor | NCT00086515 | Multiple countries |

*Abbreviations: DPP4 inhibitor: dipeptidyl peptidase 4 inhibitor; GLP-1 agonist: glucagon-like peptide-1 agonist; NA: not available; SGLT2 inhibitor: sodium–glucose cotransporter 2 inhibitor*

**eTable 6A: League table of the primary outcome: overall hematologic malignancy**

| Tirzepatide | 2.31 (0.56,9.45) | 2.69 (0.55,13.17) | 2.70 (0.48,15.18) | 2.28 (0.15,34.29) | 3.40 (0.86,13.44) | 3.86 (0.96,15.52) | ***4.43 (1.13,17.43)** | ***4.54 (1.28,16.08)** | ***4.91 (1.22,19.67)** | ***5.25 (1.29,21.31)** | ***5.12 (1.36,19.25)** | ***5.58 (1.07,29.03)** | 6.83 (0.22,213.02) | ***5.73 (1.21,27.23)** | ***7.27 (1.36,38.85)** | 10.90 (0.61,193.27) | ***7.08 (1.74,28.83)** | 15.86 (0.63,397.27) | ***12.74 (1.01,161.46)** | ***9.88 (2.38,40.92)** | 22.80 (0.85,611.07) |
| --- | --- | --- | --- | --- | --- | --- | --- | --- | --- | --- | --- | --- | --- | --- | --- | --- | --- | --- | --- | --- | --- |
| 0.43 (0.11,1.77) | Linagliptin | 1.16 (0.37,3.67) | 1.17 (0.31,4.44) | 0.99 (0.08,11.78) | 1.48 (0.60,3.63) | 1.67 (0.71,3.93) | 1.92 (0.85,4.36) | ***1.97 (1.05,3.68)** | 2.13 (0.91,4.97) | 2.28 (0.95,5.42) | ***2.22 (1.06,4.65)** | 2.42 (0.71,8.27) | 2.96 (0.11,77.08) | 2.48 (0.82,7.50) | 3.15 (0.89,11.17) | 4.72 (0.33,67.36) | ***3.07 (1.28,7.35)** | 6.87 (0.33,141.90) | 5.52 (0.56,54.49) | ***4.28 (1.74,10.55)** | 9.88 (0.45,219.19) |
| 0.37 (0.08,1.82) | 0.86 (0.27,2.70) | Canagliflozin | 1.00 (0.22,4.60) | 0.85 (0.06,11.24) | 1.27 (0.40,4.04) | 1.44 (0.47,4.42) | 1.65 (0.55,4.95) | 1.69 (0.65,4.43) | 1.83 (0.60,5.60) | 1.95 (0.63,6.09) | 1.90 (0.67,5.39) | 2.08 (0.50,8.69) | 2.54 (0.09,71.78) | 2.13 (0.57,8.03) | 2.70 (0.63,11.67) | 4.05 (0.26,63.85) | 2.63 (0.84,8.24) | 5.90 (0.26,132.94) | 4.74 (0.43,52.44) | ***3.68 (1.15,11.74)** | 8.48 (0.35,204.95) |
| 0.37 (0.07,2.09) | 0.86 (0.23,3.25) | 1.00 (0.22,4.56) | Lixisenatide | 0.84 (0.06,12.23) | 1.26 (0.33,4.84) | 1.43 (0.40,5.14) | 1.64 (0.45,5.98) | 1.68 (0.52,5.47) | 1.82 (0.49,6.74) | 1.95 (0.52,7.32) | 1.90 (0.55,6.58) | 2.07 (0.42,10.09) | 2.53 (0.08,76.58) | 2.12 (0.48,9.42) | 2.69 (0.54,13.51) | 4.04 (0.24,69.09) | 2.62 (0.70,9.90) | 5.88 (0.24,142.52) | 4.72 (0.39,57.41) | 3.66 (0.95,14.06) | 8.45 (0.33,219.37) |
| 0.44 (0.03,6.61) | 1.01 (0.08,12.08) | 1.18 (0.09,15.65) | 1.18 (0.08,17.14) | Vildagliptin | 1.49 (0.12,17.93) | 1.69 (0.14,19.99) | 1.95 (0.17,22.69) | 1.99 (0.18,21.96) | 2.15 (0.18,25.38) | 2.31 (0.19,27.35) | 2.25 (0.20,25.55) | 2.45 (0.18,33.72) | 3.00 (0.06,163.50) | 2.51 (0.19,32.74) | 3.19 (0.23,44.68) | 4.78 (0.14,162.45) | 3.11 (0.26,36.95) | 6.96 (0.15,314.90) | 5.59 (0.22,145.20) | 4.34 (0.36,52.06) | 10.01 (0.21,479.41) |
| 0.29 (0.07,1.16) | 0.68 (0.28,1.67) | 0.79 (0.25,2.52) | 0.79 (0.21,3.04) | 0.67 (0.06,8.03) | Semaglutide | 1.13 (0.47,2.71) | 1.30 (0.57,2.96) | 1.33 (0.70,2.55) | 1.44 (0.61,3.43) | 1.54 (0.64,3.74) | 1.50 (0.70,3.21) | 1.64 (0.47,5.67) | 2.01 (0.08,52.49) | 1.68 (0.55,5.15) | 2.14 (0.60,7.66) | 3.20 (0.22,45.79) | 2.08 (0.85,5.07) | 4.66 (0.22,96.65) | 3.74 (0.38,37.17) | ***2.90 (1.16,7.26)** | 6.70 (0.30,149.28) |
| 0.26 (0.06,1.04) | 0.60 (0.25,1.41) | 0.70 (0.23,2.15) | 0.70 (0.19,2.51) | 0.59 (0.05,6.97) | 0.88 (0.37,2.11) | Liraglutide | 1.15 (0.52,2.53) | 1.18 (0.66,2.11) | 1.27 (0.57,2.85) | 1.36 (0.59,3.15) | 1.33 (0.66,2.69) | 1.45 (0.43,4.85) | 1.77 (0.07,45.76) | 1.49 (0.50,4.39) | 1.88 (0.54,6.55) | 2.82 (0.20,39.92) | 1.84 (0.79,4.27) | 4.11 (0.20,84.19) | 3.30 (0.34,32.24) | ***2.56 (1.07,6.13)** | 5.91 (0.27,130.07) |
| ***0.23 (0.06,0.89)** | 0.52 (0.23,1.18) | 0.61 (0.20,1.82) | 0.61 (0.17,2.22) | 0.51 (0.04,6.00) | 0.77 (0.34,1.75) | 0.87 (0.40,1.91) | Sitagliptin | 1.03 (0.60,1.74) | 1.11 (0.51,2.42) | 1.19 (0.53,2.65) | 1.15 (0.60,2.24) | 1.26 (0.39,4.12) | 1.54 (0.06,39.48) | 1.29 (0.46,3.66) | 1.64 (0.48,5.57) | 2.46 (0.19,32.46) | 1.60 (0.72,3.55) | 3.58 (0.18,72.58) | 2.88 (0.30,27.71) | 2.23 (0.96,5.16) | 5.15 (0.24,112.16) |
| ***0.22 (0.06,0.78)** | ***0.51 (0.27,0.95)** | 0.59 (0.23,1.55) | 0.59 (0.18,1.93) | 0.50 (0.05,5.52) | 0.75 (0.39,1.43) | 0.85 (0.47,1.52) | 0.98 (0.57,1.66) | Placebo_or_  Control | 1.08 (0.61,1.92) | 1.16 (0.63,2.11) | 1.13 (0.76,1.67) | 1.23 (0.43,3.54) | 1.50 (0.06,36.86) | 1.26 (0.51,3.14) | 1.60 (0.53,4.81) | 2.40 (0.18,31.76) | 1.56 (0.85,2.87) | 3.49 (0.18,67.54) | 2.81 (0.31,25.38) | ***2.17 (1.14,4.17)** | 5.02 (0.24,104.48) |
| ***0.20 (0.05,0.82)** | 0.47 (0.20,1.10) | 0.55 (0.18,1.68) | 0.55 (0.15,2.04) | 0.46 (0.04,5.47) | 0.69 (0.29,1.65) | 0.79 (0.35,1.76) | 0.90 (0.41,1.97) | 0.93 (0.52,1.64) | Exenatide | 1.07 (0.47,2.46) | 1.04 (0.52,2.10) | 1.14 (0.34,3.79) | 1.39 (0.05,35.91) | 1.17 (0.40,3.43) | 1.48 (0.43,5.13) | 2.22 (0.16,31.31) | 1.44 (0.62,3.34) | 3.23 (0.16,66.06) | 2.60 (0.27,25.28) | 2.01 (0.85,4.79) | 4.65 (0.21,102.06) |
| ***0.19 (0.05,0.77)** | 0.44 (0.18,1.05) | 0.51 (0.16,1.59) | 0.51 (0.14,1.93) | 0.43 (0.04,5.15) | 0.65 (0.27,1.57) | 0.73 (0.32,1.70) | 0.84 (0.38,1.89) | 0.87 (0.47,1.58) | 0.93 (0.41,2.15) | Saxagliptin | 0.97 (0.47,2.01) | 1.06 (0.31,3.60) | 1.30 (0.05,33.74) | 1.09 (0.37,3.26) | 1.38 (0.39,4.86) | 2.07 (0.15,29.46) | 1.35 (0.57,3.18) | 3.02 (0.15,62.09) | 2.43 (0.25,23.81) | 1.88 (0.77,4.57) | 4.34 (0.20,95.92) |
| ***0.20 (0.05,0.73)** | ***0.45 (0.22,0.94)** | 0.53 (0.19,1.49) | 0.53 (0.15,1.83) | 0.45 (0.04,5.06) | 0.67 (0.31,1.42) | 0.75 (0.37,1.53) | 0.87 (0.45,1.68) | 0.89 (0.60,1.32) | 0.96 (0.48,1.93) | 1.03 (0.50,2.11) | Dapagliflozin | 1.09 (0.35,3.38) | 1.34 (0.05,33.53) | 1.12 (0.41,3.03) | 1.42 (0.44,4.58) | 2.13 (0.16,29.06) | 1.38 (0.67,2.87) | 3.10 (0.16,61.56) | 2.49 (0.27,23.34) | 1.93 (0.90,4.13) | 4.46 (0.21,95.18) |
| ***0.18 (0.03,0.93)** | 0.41 (0.12,1.41) | 0.48 (0.12,2.02) | 0.48 (0.10,2.36) | 0.41 (0.03,5.62) | 0.61 (0.18,2.11) | 0.69 (0.21,2.32) | 0.79 (0.24,2.60) | 0.81 (0.28,2.35) | 0.88 (0.26,2.94) | 0.94 (0.28,3.19) | 0.92 (0.30,2.84) | Sotagliflozin | 1.22 (0.04,35.60) | 1.03 (0.25,4.16) | 1.30 (0.28,6.00) | 1.95 (0.12,31.86) | 1.27 (0.37,4.31) | 2.84 (0.12,66.07) | 2.28 (0.20,26.30) | 1.77 (0.51,6.14) | 4.09 (0.16,101.79) |
| 0.15 (0.00,4.56) | 0.34 (0.01,8.79) | 0.39 (0.01,11.11) | 0.39 (0.01,11.94) | 0.33 (0.01,18.18) | 0.50 (0.02,13.03) | 0.56 (0.02,14.59) | 0.65 (0.03,16.61) | 0.66 (0.03,16.30) | 0.72 (0.03,18.53) | 0.77 (0.03,19.93) | 0.75 (0.03,18.81) | 0.82 (0.03,23.75) | Bexagliflozin | 0.84 (0.03,23.35) | 1.06 (0.04,31.35) | 1.59 (0.03,97.38) | 1.04 (0.04,26.91) | 2.32 (0.03,181.66) | 1.87 (0.04,90.66) | 1.45 (0.06,37.83) | 3.34 (0.04,274.60) |
| ***0.17 (0.04,0.83)** | 0.40 (0.13,1.22) | 0.47 (0.12,1.77) | 0.47 (0.11,2.09) | 0.40 (0.03,5.18) | 0.59 (0.19,1.82) | 0.67 (0.23,1.99) | 0.77 (0.27,2.19) | 0.79 (0.32,1.97) | 0.86 (0.29,2.52) | 0.92 (0.31,2.74) | 0.89 (0.33,2.41) | 0.97 (0.24,3.94) | 1.19 (0.04,33.20) | Ertugliflozin | 1.27 (0.30,5.30) | 1.90 (0.14,25.11) | 1.24 (0.41,3.70) | 2.77 (0.12,61.42) | 2.22 (0.21,24.12) | 1.72 (0.56,5.29) | 3.98 (0.17,94.73) |
| ***0.14 (0.03,0.73)** | 0.32 (0.09,1.12) | 0.37 (0.09,1.60) | 0.37 (0.07,1.86) | 0.31 (0.02,4.39) | 0.47 (0.13,1.68) | 0.53 (0.15,1.84) | 0.61 (0.18,2.07) | 0.62 (0.21,1.88) | 0.68 (0.20,2.34) | 0.72 (0.21,2.53) | 0.70 (0.22,2.27) | 0.77 (0.17,3.54) | 0.94 (0.03,27.68) | 0.79 (0.19,3.29) | Albiglutide | 1.50 (0.09,24.84) | 0.97 (0.28,3.43) | 2.18 (0.09,51.43) | 1.75 (0.15,20.56) | 1.36 (0.38,4.88) | 3.14 (0.12,79.21) |
| 0.09 (0.01,1.63) | 0.21 (0.01,3.02) | 0.25 (0.02,3.89) | 0.25 (0.01,4.24) | 0.21 (0.01,7.10) | 0.31 (0.02,4.47) | 0.35 (0.03,5.00) | 0.41 (0.03,5.37) | 0.42 (0.03,5.52) | 0.45 (0.03,6.35) | 0.48 (0.03,6.84) | 0.47 (0.03,6.41) | 0.51 (0.03,8.36) | 0.63 (0.01,38.30) | 0.53 (0.04,6.95) | 0.67 (0.04,11.06) | Ertugliflozin_  Sitagliptin | 0.65 (0.05,9.23) | 1.46 (0.03,74.16) | 1.17 (0.04,34.87) | 0.91 (0.06,13.02) | 2.09 (0.04,112.71) |
| ***0.14 (0.03,0.57)** | ***0.33 (0.14,0.78)** | 0.38 (0.12,1.19) | 0.38 (0.10,1.44) | 0.32 (0.03,3.83) | 0.48 (0.20,1.17) | 0.54 (0.23,1.27) | 0.63 (0.28,1.39) | 0.64 (0.35,1.18) | 0.69 (0.30,1.60) | 0.74 (0.31,1.75) | 0.72 (0.35,1.50) | 0.79 (0.23,2.68) | 0.96 (0.04,25.06) | 0.81 (0.27,2.43) | 1.03 (0.29,3.62) | 1.54 (0.11,21.85) | Empagliflozin | 2.24 (0.11,46.12) | 1.80 (0.18,17.69) | 1.40 (0.57,3.41) | 3.22 (0.15,71.25) |
| 0.06 (0.00,1.58) | 0.15 (0.01,3.00) | 0.17 (0.01,3.82) | 0.17 (0.01,4.12) | 0.14 (0.00,6.50) | 0.21 (0.01,4.45) | 0.24 (0.01,4.98) | 0.28 (0.01,5.67) | 0.29 (0.01,5.54) | 0.31 (0.02,6.33) | 0.33 (0.02,6.81) | 0.32 (0.02,6.41) | 0.35 (0.02,8.18) | 0.43 (0.01,33.72) | 0.36 (0.02,8.02) | 0.46 (0.02,10.81) | 0.69 (0.01,35.00) | 0.45 (0.02,9.19) | Efpeglenatide | 0.80 (0.02,32.22) | 0.62 (0.03,12.93) | 1.44 (0.02,99.96) |
| ***0.08 (0.01,0.99)** | 0.18 (0.02,1.79) | 0.21 (0.02,2.33) | 0.21 (0.02,2.57) | 0.18 (0.01,4.64) | 0.27 (0.03,2.65) | 0.30 (0.03,2.95) | 0.35 (0.04,3.35) | 0.36 (0.04,3.22) | 0.39 (0.04,3.75) | 0.41 (0.04,4.04) | 0.40 (0.04,3.76) | 0.44 (0.04,5.04) | 0.54 (0.01,26.06) | 0.45 (0.04,4.88) | 0.57 (0.05,6.69) | 0.85 (0.03,25.48) | 0.56 (0.06,5.46) | 1.24 (0.03,49.90) | Alogliptin | 0.78 (0.08,7.70) | 1.79 (0.04,76.11) |
| ***0.10 (0.02,0.42)** | ***0.23 (0.09,0.58)** | ***0.27 (0.09,0.87)** | 0.27 (0.07,1.05) | 0.23 (0.02,2.77) | ***0.34 (0.14,0.86)** | ***0.39 (0.16,0.94)** | 0.45 (0.19,1.04) | ***0.46 (0.24,0.88)** | 0.50 (0.21,1.18) | 0.53 (0.22,1.29) | 0.52 (0.24,1.11) | 0.56 (0.16,1.96) | 0.69 (0.03,18.10) | 0.58 (0.19,1.78) | 0.74 (0.21,2.64) | 1.10 (0.08,15.83) | 0.72 (0.29,1.75) | 1.61 (0.08,33.33) | 1.29 (0.13,12.82) | Dulaglutide | 2.31 (0.10,51.47) |
| 0.04 (0.00,1.18) | 0.10 (0.00,2.25) | 0.12 (0.00,2.85) | 0.12 (0.00,3.07) | 0.10 (0.00,4.79) | 0.15 (0.01,3.33) | 0.17 (0.01,3.72) | 0.19 (0.01,4.24) | 0.20 (0.01,4.15) | 0.22 (0.01,4.73) | 0.23 (0.01,5.09) | 0.22 (0.01,4.79) | 0.24 (0.01,6.10) | 0.30 (0.00,24.66) | 0.25 (0.01,5.98) | 0.32 (0.01,8.05) | 0.48 (0.01,25.73) | 0.31 (0.01,6.87) | 0.70 (0.01,48.36) | 0.56 (0.01,23.78) | 0.43 (0.02,9.66) | Omarigliptin |

Data presents RR [95%CIs]. Network meta-analysis results are presented as estimate effect sizes for the outcome of overall hematologic malignancy risk. Interventions are reported in order of mean ranking of beneficially prophylactic effect on overall hematologic malignancy risk, and outcomes are expressed as risk ratio (RR) (95% confidence intervals) (95%CIs). For the upper-right portion, RR of less than 1 indicates that the treatment specified in the row got more beneficial effect than that specified in the column. For the lower-left portion, RR of less than 1 indicates that the treatment specified in the column has more beneficial effect than that specified in the row. Bold results marked with * indicate statistical significance.

**eTable 6B: League table of the primary outcome: leukemia**

| Tirzepatide | 1.19 (0.12,11.35) | 1.29 (0.16,10.77) | 1.32 (0.08,22.25) | 1.60 (0.19,13.50) | 1.87 (0.22,16.14) | 1.90 (0.23,15.97) | 2.02 (0.25,16.40) | 2.60 (0.09,72.46) | 2.60 (0.38,17.90) | 2.59 (0.41,16.40) | 2.88 (0.24,34.72) | 3.87 (0.10,155.72) | 3.21 (0.32,32.39) | 3.21 (0.42,24.66) | 4.32 (0.42,44.64) | 4.12 (0.49,34.51) | 6.47 (0.38,109.72) |
| --- | --- | --- | --- | --- | --- | --- | --- | --- | --- | --- | --- | --- | --- | --- | --- | --- | --- |
| 0.84 (0.09,8.00) | Canagliflozin | 1.09 (0.21,5.72) | 1.10 (0.09,13.49) | 1.35 (0.25,7.18) | 1.57 (0.28,8.65) | 1.60 (0.30,8.49) | 1.70 (0.33,8.64) | 2.18 (0.10,46.37) | 2.18 (0.53,8.94) | 2.17 (0.60,7.92) | 2.42 (0.29,20.00) | 3.25 (0.10,102.57) | 2.70 (0.40,18.00) | 2.70 (0.57,12.78) | 3.63 (0.53,24.96) | 3.46 (0.65,18.34) | 5.43 (0.44,66.56) |
| 0.77 (0.09,6.45) | 0.92 (0.17,4.85) | Linagliptin | 1.02 (0.09,11.02) | 1.24 (0.28,5.50) | 1.44 (0.31,6.65) | 1.47 (0.33,6.49) | 1.56 (0.37,6.57) | 2.01 (0.10,38.76) | 2.01 (0.61,6.57) | 2.00 (0.71,5.68) | 2.23 (0.31,15.96) | 3.00 (0.10,86.71) | 2.49 (0.44,14.11) | 2.48 (0.64,9.63) | 3.34 (0.57,19.62) | 3.19 (0.72,14.03) | 5.00 (0.46,54.39) |
| 0.76 (0.04,12.87) | 0.91 (0.07,11.06) | 0.98 (0.09,10.65) | Lixisenatide | 1.22 (0.14,10.78) | 1.42 (0.13,15.89) | 1.45 (0.13,15.78) | 1.54 (0.15,16.25) | 1.97 (0.06,65.50) | 1.98 (0.22,18.10) | 1.97 (0.23,16.77) | 2.19 (0.14,33.12) | 2.94 (0.06,138.48) | 2.44 (0.19,31.40) | 2.44 (0.24,24.60) | 3.28 (0.25,43.18) | 3.13 (0.29,34.09) | 4.92 (0.24,102.05) |
| 0.62 (0.07,5.25) | 0.74 (0.14,3.96) | 0.81 (0.18,3.57) | 0.82 (0.09,7.25) | Liraglutide | 1.16 (0.25,5.43) | 1.19 (0.27,5.30) | 1.26 (0.30,5.37) | 1.62 (0.08,31.44) | 1.62 (0.49,5.39) | 1.61 (0.56,4.67) | 1.79 (0.25,12.99) | 2.41 (0.08,70.29) | 2.00 (0.35,11.51) | 2.00 (0.51,7.87) | 2.69 (0.45,15.99) | 2.57 (0.58,11.46) | 4.03 (0.37,44.21) |
| 0.54 (0.06,4.64) | 0.64 (0.12,3.53) | 0.69 (0.15,3.19) | 0.70 (0.06,7.90) | 0.86 (0.18,4.02) | Semaglutide | 1.02 (0.22,4.74) | 1.08 (0.24,4.81) | 1.39 (0.07,27.59) | 1.39 (0.40,4.86) | 1.39 (0.45,4.24) | 1.54 (0.21,11.51) | 2.08 (0.07,61.52) | 1.72 (0.29,10.24) | 1.72 (0.42,7.07) | 2.31 (0.38,14.22) | 2.21 (0.48,10.25) | 3.47 (0.31,38.97) |
| 0.52 (0.06,4.40) | 0.63 (0.12,3.32) | 0.68 (0.15,2.99) | 0.69 (0.06,7.52) | 0.84 (0.19,3.76) | 0.98 (0.21,4.55) | Saxagliptin | 1.06 (0.25,4.50) | 1.36 (0.07,26.41) | 1.36 (0.41,4.50) | 1.36 (0.47,3.90) | 1.51 (0.21,10.90) | 2.03 (0.07,59.05) | 1.69 (0.30,9.64) | 1.69 (0.43,6.59) | 2.27 (0.38,13.41) | 2.16 (0.49,9.60) | 3.39 (0.31,37.10) |
| 0.49 (0.06,4.02) | 0.59 (0.12,3.00) | 0.64 (0.15,2.69) | 0.65 (0.06,6.89) | 0.79 (0.19,3.39) | 0.92 (0.21,4.10) | 0.94 (0.22,4.00) | Exenatide | 1.28 (0.07,24.33) | 1.29 (0.41,4.00) | 1.28 (0.48,3.44) | 1.42 (0.20,9.92) | 1.92 (0.07,54.55) | 1.59 (0.29,8.74) | 1.59 (0.43,5.90) | 2.14 (0.38,12.16) | 2.04 (0.48,8.64) | 3.20 (0.30,33.99) |
| 0.39 (0.01,10.76) | 0.46 (0.02,9.76) | 0.50 (0.03,9.61) | 0.51 (0.02,16.82) | 0.62 (0.03,12.01) | 0.72 (0.04,14.25) | 0.73 (0.04,14.23) | 0.78 (0.04,14.74) | Vildagliptin | 1.00 (0.06,16.91) | 1.00 (0.06,15.92) | 1.11 (0.04,28.18) | 1.49 (0.02,102.77) | 1.24 (0.06,27.46) | 1.24 (0.07,22.53) | 1.66 (0.07,37.60) | 1.59 (0.08,30.74) | 2.49 (0.07,82.88) |
| 0.38 (0.06,2.65) | 0.46 (0.11,1.88) | 0.50 (0.15,1.63) | 0.51 (0.06,4.64) | 0.62 (0.19,2.05) | 0.72 (0.21,2.51) | 0.73 (0.22,2.42) | 0.78 (0.25,2.42) | 1.00 (0.06,16.88) | Dapagliflozin | 1.00 (0.57,1.75) | 1.11 (0.19,6.46) | 1.49 (0.06,38.39) | 1.24 (0.28,5.53) | 1.24 (0.44,3.47) | 1.66 (0.36,7.73) | 1.59 (0.48,5.23) | 2.49 (0.27,22.89) |
| 0.39 (0.06,2.45) | 0.46 (0.13,1.68) | 0.50 (0.18,1.42) | 0.51 (0.06,4.33) | 0.62 (0.21,1.79) | 0.72 (0.24,2.20) | 0.74 (0.26,2.11) | 0.78 (0.29,2.10) | 1.00 (0.06,16.01) | 1.00 (0.57,1.76) | Placebo_or_Control | 1.11 (0.21,5.91) | 1.50 (0.06,36.70) | 1.24 (0.31,4.98) | 1.24 (0.52,2.95) | 1.67 (0.40,6.98) | 1.59 (0.56,4.56) | 2.50 (0.29,21.38) |
| 0.35 (0.03,4.19) | 0.41 (0.05,3.42) | 0.45 (0.06,3.22) | 0.46 (0.03,6.92) | 0.56 (0.08,4.04) | 0.65 (0.09,4.84) | 0.66 (0.09,4.77) | 0.70 (0.10,4.89) | 0.90 (0.04,22.92) | 0.90 (0.15,5.26) | 0.90 (0.17,4.78) | Sotagliflozin | 1.35 (0.04,49.72) | 1.12 (0.13,9.80) | 1.12 (0.17,7.32) | 1.50 (0.17,13.54) | 1.43 (0.20,10.31) | 2.25 (0.15,34.11) |
| 0.26 (0.01,10.39) | 0.31 (0.01,9.71) | 0.33 (0.01,9.67) | 0.34 (0.01,15.98) | 0.41 (0.01,12.07) | 0.48 (0.02,14.29) | 0.49 (0.02,14.30) | 0.52 (0.02,14.86) | 0.67 (0.01,46.19) | 0.67 (0.03,17.29) | 0.67 (0.03,16.40) | 0.74 (0.02,27.48) | Efpeglenatide | 0.83 (0.03,27.17) | 0.83 (0.03,22.81) | 1.12 (0.03,37.13) | 1.06 (0.04,30.90) | 1.67 (0.04,78.73) |
| 0.31 (0.03,3.13) | 0.37 (0.06,2.47) | 0.40 (0.07,2.28) | 0.41 (0.03,5.25) | 0.50 (0.09,2.86) | 0.58 (0.10,3.45) | 0.59 (0.10,3.39) | 0.63 (0.11,3.45) | 0.81 (0.04,17.90) | 0.81 (0.18,3.61) | 0.80 (0.20,3.22) | 0.90 (0.10,7.86) | 1.20 (0.04,39.40) | Albiglutide | 1.00 (0.19,5.12) | 1.34 (0.18,9.86) | 1.28 (0.22,7.32) | 2.01 (0.16,25.91) |
| 0.31 (0.04,2.39) | 0.37 (0.08,1.76) | 0.40 (0.10,1.56) | 0.41 (0.04,4.13) | 0.50 (0.13,1.97) | 0.58 (0.14,2.39) | 0.59 (0.15,2.32) | 0.63 (0.17,2.34) | 0.81 (0.04,14.73) | 0.81 (0.29,2.27) | 0.81 (0.34,1.91) | 0.90 (0.14,5.88) | 1.21 (0.04,33.19) | 1.00 (0.20,5.14) | Sitagliptin | 1.35 (0.25,7.16) | 1.28 (0.34,4.78) | 2.01 (0.20,20.38) |
| 0.23 (0.02,2.39) | 0.28 (0.04,1.90) | 0.30 (0.05,1.76) | 0.30 (0.02,4.00) | 0.37 (0.06,2.21) | 0.43 (0.07,2.65) | 0.44 (0.07,2.61) | 0.47 (0.08,2.66) | 0.60 (0.03,13.59) | 0.60 (0.13,2.80) | 0.60 (0.14,2.51) | 0.67 (0.07,6.01) | 0.90 (0.03,29.85) | 0.74 (0.10,5.47) | 0.74 (0.14,3.96) | Dulaglutide | 0.95 (0.16,5.64) | 1.50 (0.11,19.76) |
| 0.24 (0.03,2.03) | 0.29 (0.05,1.53) | 0.31 (0.07,1.38) | 0.32 (0.03,3.48) | 0.39 (0.09,1.74) | 0.45 (0.10,2.10) | 0.46 (0.10,2.05) | 0.49 (0.12,2.08) | 0.63 (0.03,12.21) | 0.63 (0.19,2.08) | 0.63 (0.22,1.80) | 0.70 (0.10,5.04) | 0.94 (0.03,27.30) | 0.78 (0.14,4.46) | 0.78 (0.21,2.90) | 1.05 (0.18,6.20) | Empagliflozin | 1.57 (0.14,17.15) |
| 0.15 (0.01,2.62) | 0.18 (0.02,2.26) | 0.20 (0.02,2.17) | 0.20 (0.01,4.22) | 0.25 (0.02,2.72) | 0.29 (0.03,3.24) | 0.29 (0.03,3.22) | 0.31 (0.03,3.32) | 0.40 (0.01,13.36) | 0.40 (0.04,3.70) | 0.40 (0.05,3.42) | 0.45 (0.03,6.76) | 0.60 (0.01,28.23) | 0.50 (0.04,6.41) | 0.50 (0.05,5.02) | 0.67 (0.05,8.81) | 0.64 (0.06,6.96) | Ertugliflozin |

Data presents RR [95%CIs]. Network meta-analysis results are presented as estimate effect sizes for the outcome of leukemia risk. Interventions are reported in order of mean ranking of beneficially prophylactic effect on leukemia risk, and outcomes are expressed as risk ratio (RR) (95% confidence intervals) (95%CIs). For the upper-right portion, RR of less than 1 indicates that the treatment specified in the row got more beneficial effect than that specified in the column. For the lower-left portion, RR of less than 1 indicates that the treatment specified in the column has more beneficial effect than that specified in the row. Bold results marked with * indicate statistical significance.

**eTable 6C: League table of the primary outcome: acute lymphocytic leukemia**

| Ertugliflozin | 1.99 (0.02,184.02) | 2.00 (0.02,184.88) | 6.00 (0.24,147.29) | 17.99 (0.19,1661.94) | 16.91 (0.35,823.01) |
| --- | --- | --- | --- | --- | --- |
| 0.50 (0.01,46.37) | Linagliptin | 1.00 (0.01,92.80) | 3.01 (0.12,73.93) | 9.03 (0.10,834.21) | 8.49 (0.17,413.11) |
| 0.50 (0.01,46.13) | 1.00 (0.01,91.89) | Semaglutide | 3.00 (0.12,73.54) | 8.99 (0.10,829.93) | 8.45 (0.17,410.97) |
| 0.17 (0.01,4.09) | 0.33 (0.01,8.14) | 0.33 (0.01,8.18) | Placebo_or_Control | 3.00 (0.12,73.56) | 2.82 (0.31,25.49) |
| 0.06 (0.00,5.14) | 0.11 (0.00,10.23) | 0.11 (0.00,10.28) | 0.33 (0.01,8.19) | Sotagliflozin | 0.94 (0.02,45.75) |
| 0.06 (0.00,2.88) | 0.12 (0.00,5.73) | 0.12 (0.00,5.76) | 0.35 (0.04,3.21) | 1.06 (0.02,51.77) | Empagliflozin |

Data presents RR [95%CIs]. Network meta-analysis results are presented as estimate effect sizes for the outcome of acute lymphocytic leukemia risk. Interventions are reported in order of mean ranking of beneficially prophylactic effect on acute lymphocytic leukemia risk, and outcomes are expressed as risk ratio (RR) (95% confidence intervals) (95%CIs). For the upper-right portion, RR of less than 1 indicates that the treatment specified in the row got more beneficial effect than that specified in the column. For the lower-left portion, RR of less than 1 indicates that the treatment specified in the column has more beneficial effect than that specified in the row. Bold results marked with * indicate statistical significance.

**eTable 6D: League table of the primary outcome: acute myeloid leukemia**

| Linagliptin | 1.19 (0.05,26.11) | 1.58 (0.07,36.44) | 1.59 (0.03,75.93) | 1.60 (0.03,76.27) | 1.58 (0.02,120.69) | 2.05 (0.11,37.31) | 2.36 (0.11,50.43) | 3.86 (0.34,43.92) | 4.75 (0.25,88.76) | 4.75 (0.54,41.78) | 6.79 (0.40,115.17) | 7.51 (0.37,153.57) | 7.91 (0.48,131.48) | 11.87 (0.28,496.88) |
| --- | --- | --- | --- | --- | --- | --- | --- | --- | --- | --- | --- | --- | --- | --- |
| 0.84 (0.04,18.37) | Exenatide | 1.33 (0.06,30.93) | 1.33 (0.03,64.30) | 1.34 (0.03,64.59) | 1.33 (0.02,102.11) | 1.72 (0.09,31.70) | 1.98 (0.09,42.82) | 3.24 (0.28,37.40) | 3.99 (0.21,75.40) | 3.98 (0.45,35.64) | 5.69 (0.33,97.88) | 6.30 (0.30,130.40) | 6.64 (0.39,111.75) | 9.96 (0.24,420.94) |
| 0.63 (0.03,14.54) | 0.75 (0.03,17.54) | Canagliflozin | 1.00 (0.02,50.37) | 1.01 (0.02,50.60) | 1.00 (0.01,79.65) | 1.30 (0.07,25.16) | 1.49 (0.07,33.88) | 2.44 (0.20,29.97) | 3.00 (0.15,59.80) | 3.00 (0.31,28.76) | 4.29 (0.24,77.76) | 4.74 (0.22,103.27) | 5.00 (0.28,88.82) | 7.50 (0.17,330.21) |
| 0.63 (0.01,30.21) | 0.75 (0.02,36.36) | 1.00 (0.02,50.23) | Dulaglutide | 1.01 (0.01,92.84) | 1.00 (0.01,138.10) | 1.29 (0.03,54.06) | 1.49 (0.03,70.57) | 2.44 (0.08,71.57) | 3.00 (0.07,127.85) | 3.00 (0.12,73.54) | 4.28 (0.11,169.49) | 4.74 (0.10,216.91) | 4.99 (0.13,194.43) | 7.49 (0.09,616.86) |
| 0.63 (0.01,29.92) | 0.75 (0.02,36.02) | 0.99 (0.02,49.76) | 0.99 (0.01,91.55) | Tirzepatide | 0.99 (0.01,136.87) | 1.29 (0.03,53.55) | 1.48 (0.03,69.90) | 2.42 (0.08,70.87) | 2.98 (0.07,126.64) | 2.98 (0.12,72.80) | 4.25 (0.11,167.87) | 4.70 (0.10,214.86) | 4.95 (0.13,192.56) | 7.44 (0.09,611.23) |
| 0.63 (0.01,48.05) | 0.75 (0.01,57.77) | 1.00 (0.01,79.49) | 1.00 (0.01,138.19) | 1.01 (0.01,138.89) | Lixisenatide | 1.30 (0.02,87.30) | 1.49 (0.02,112.36) | 2.44 (0.05,120.70) | 3.00 (0.12,73.21) | 3.00 (0.07,127.17) | 4.28 (0.07,275.38) | 4.74 (0.06,346.70) | 4.99 (0.08,316.48) | 7.49 (0.06,931.58) |
| 0.49 (0.03,8.86) | 0.58 (0.03,10.70) | 0.77 (0.04,14.97) | 0.77 (0.02,32.26) | 0.78 (0.02,32.40) | 0.77 (0.01,52.06) | Semaglutide | 1.15 (0.06,20.63) | 1.88 (0.21,17.09) | 2.32 (0.15,35.99) | 2.31 (0.34,15.78) | 3.31 (0.24,46.39) | 3.66 (0.21,62.64) | 3.85 (0.28,52.88) | 5.78 (0.16,210.02) |
| 0.42 (0.02,9.07) | 0.51 (0.02,10.94) | 0.67 (0.03,15.27) | 0.67 (0.01,31.88) | 0.68 (0.01,32.03) | 0.67 (0.01,50.73) | 0.87 (0.05,15.62) | Saxagliptin | 1.64 (0.15,18.34) | 2.02 (0.11,37.16) | 2.01 (0.23,17.41) | 2.88 (0.17,48.19) | 3.18 (0.16,64.31) | 3.35 (0.20,55.01) | 5.03 (0.12,208.57) |
| 0.26 (0.02,2.94) | 0.31 (0.03,3.56) | 0.41 (0.03,5.03) | 0.41 (0.01,12.05) | 0.41 (0.01,12.10) | 0.41 (0.01,20.31) | 0.53 (0.06,4.82) | 0.61 (0.05,6.83) | Dapagliflozin | 1.23 (0.13,11.57) | 1.23 (0.41,3.65) | 1.76 (0.21,14.56) | 1.94 (0.18,20.55) | 2.05 (0.25,16.50) | 3.07 (0.12,77.27) |
| 0.21 (0.01,3.93) | 0.25 (0.01,4.74) | 0.33 (0.02,6.63) | 0.33 (0.01,14.22) | 0.34 (0.01,14.28) | 0.33 (0.01,8.13) | 0.43 (0.03,6.71) | 0.50 (0.03,9.14) | 0.81 (0.09,7.64) | Liraglutide | 1.00 (0.14,7.09) | 1.43 (0.10,20.62) | 1.58 (0.09,27.78) | 1.66 (0.12,23.51) | 2.50 (0.07,92.62) |
| 0.21 (0.02,1.85) | 0.25 (0.03,2.24) | 0.33 (0.03,3.19) | 0.33 (0.01,8.19) | 0.34 (0.01,8.22) | 0.33 (0.01,14.15) | 0.43 (0.06,2.95) | 0.50 (0.06,4.29) | 0.81 (0.27,2.41) | 1.00 (0.14,7.10) | Placebo_or_Control | 1.43 (0.23,8.77) | 1.58 (0.19,12.82) | 1.67 (0.28,9.89) | 2.50 (0.12,52.04) |
| 0.15 (0.01,2.50) | 0.18 (0.01,3.02) | 0.23 (0.01,4.23) | 0.23 (0.01,9.25) | 0.24 (0.01,9.29) | 0.23 (0.00,15.02) | 0.30 (0.02,4.24) | 0.35 (0.02,5.82) | 0.57 (0.07,4.72) | 0.70 (0.05,10.12) | 0.70 (0.11,4.30) | Empagliflozin | 1.11 (0.07,17.66) | 1.17 (0.09,14.82) | 1.75 (0.05,60.10) |
| 0.13 (0.01,2.72) | 0.16 (0.01,3.29) | 0.21 (0.01,4.59) | 0.21 (0.00,9.66) | 0.21 (0.00,9.71) | 0.21 (0.00,15.44) | 0.27 (0.02,4.68) | 0.31 (0.02,6.34) | 0.51 (0.05,5.44) | 0.63 (0.04,11.13) | 0.63 (0.08,5.13) | 0.90 (0.06,14.41) | Sitagliptin | 1.05 (0.07,16.45) | 1.58 (0.04,63.14) |
| 0.13 (0.01,2.10) | 0.15 (0.01,2.54) | 0.20 (0.01,3.56) | 0.20 (0.01,7.81) | 0.20 (0.01,7.84) | 0.20 (0.00,12.70) | 0.26 (0.02,3.56) | 0.30 (0.02,4.89) | 0.49 (0.06,3.94) | 0.60 (0.04,8.49) | 0.60 (0.10,3.57) | 0.86 (0.07,10.91) | 0.95 (0.06,14.83) | Albiglutide | 1.50 (0.04,50.71) |
| 0.08 (0.00,3.53) | 0.10 (0.00,4.24) | 0.13 (0.00,5.87) | 0.13 (0.00,11.00) | 0.13 (0.00,11.05) | 0.13 (0.00,16.60) | 0.17 (0.00,6.28) | 0.20 (0.00,8.23) | 0.33 (0.01,8.18) | 0.40 (0.01,14.86) | 0.40 (0.02,8.33) | 0.57 (0.02,19.64) | 0.63 (0.02,25.27) | 0.67 (0.02,22.51) | Ertugliflozin |

Data presents RR [95%CIs]. Network meta-analysis results are presented as estimate effect sizes for the outcome of acute myeloid leukemia risk. Interventions are reported in order of mean ranking of beneficially prophylactic effect on acute myeloid leukemia risk, and outcomes are expressed as risk ratio (RR) (95% confidence intervals) (95%CIs). For the upper-right portion, RR of less than 1 indicates that the treatment specified in the row got more beneficial effect than that specified in the column. For the lower-left portion, RR of less than 1 indicates that the treatment specified in the column has more beneficial effect than that specified in the row. Bold results marked with * indicate statistical significance.

**eTable 6E: League table of the primary outcome: chronic lymphocytic leukemia**

| Liraglutide | 1.33 (0.03,64.35) | 1.33 (0.03,64.38) | 1.33 (0.03,64.31) | 2.25 (0.15,33.16) | 3.89 (0.36,41.59) | 4.01 (0.27,60.47) | 4.00 (0.45,35.74) | 4.32 (0.34,54.50) | 4.00 (0.12,136.80) | 4.49 (0.34,58.69) | 5.99 (0.12,289.73) | 5.98 (0.12,289.01) | 6.40 (0.31,132.62) | 12.03 (0.25,580.99) | 16.01 (0.72,354.75) | 15.86 (0.72,351.44) |
| --- | --- | --- | --- | --- | --- | --- | --- | --- | --- | --- | --- | --- | --- | --- | --- | --- |
| 0.75 (0.02,36.33) | Sotagliflozin | 1.00 (0.01,92.44) | 1.00 (0.01,92.33) | 1.69 (0.05,59.51) | 2.92 (0.10,81.24) | 3.01 (0.08,107.92) | 3.00 (0.12,73.70) | 3.25 (0.10,101.73) | 3.00 (0.04,207.15) | 3.38 (0.10,108.57) | 4.50 (0.05,415.97) | 4.49 (0.05,414.95) | 4.81 (0.10,220.39) | 9.04 (0.10,834.22) | 12.03 (0.25,581.47) | 11.91 (0.25,576.04) |
| 0.75 (0.02,36.31) | 1.00 (0.01,92.34) | Albiglutide | 1.00 (0.01,92.28) | 1.69 (0.05,59.48) | 2.92 (0.10,81.19) | 3.01 (0.08,107.86) | 3.00 (0.12,73.65) | 3.24 (0.10,101.67) | 3.00 (0.04,207.03) | 3.37 (0.10,108.51) | 4.50 (0.05,415.74) | 4.49 (0.05,414.73) | 4.81 (0.10,220.27) | 9.03 (0.10,833.77) | 12.02 (0.25,581.15) | 11.91 (0.25,575.73) |
| 0.75 (0.02,36.33) | 1.00 (0.01,92.41) | 1.00 (0.01,92.45) | Canagliflozin | 1.69 (0.05,59.52) | 2.92 (0.11,81.25) | 3.02 (0.08,107.94) | 3.00 (0.12,73.70) | 3.25 (0.10,101.74) | 3.01 (0.04,207.18) | 3.38 (0.11,108.58) | 4.50 (0.05,416.05) | 4.49 (0.05,415.03) | 4.81 (0.10,220.43) | 9.04 (0.10,834.39) | 12.03 (0.25,581.56) | 11.92 (0.25,576.13) |
| 0.44 (0.03,6.55) | 0.59 (0.02,20.81) | 0.59 (0.02,20.82) | 0.59 (0.02,20.80) | Linagliptin | 1.73 (0.28,10.49) | 1.78 (0.19,16.67) | 1.78 (0.37,8.46) | 1.92 (0.26,14.41) | 1.78 (0.07,42.76) | 2.00 (0.25,15.66) | 2.66 (0.08,93.71) | 2.66 (0.08,93.48) | 2.84 (0.21,38.77) | 5.34 (0.15,187.91) | 7.11 (0.48,104.81) | 7.05 (0.48,103.83) |
| 0.26 (0.02,2.75) | 0.34 (0.01,9.53) | 0.34 (0.01,9.53) | 0.34 (0.01,9.52) | 0.58 (0.10,3.52) | Dapagliflozin | 1.03 (0.16,6.49) | 1.03 (0.42,2.54) | 1.11 (0.23,5.31) | 1.03 (0.06,18.98) | 1.16 (0.23,5.84) | 1.54 (0.06,42.90) | 1.54 (0.06,42.79) | 1.65 (0.17,16.13) | 3.09 (0.11,86.01) | 4.12 (0.38,44.08) | 4.08 (0.38,43.67) |
| 0.25 (0.02,3.76) | 0.33 (0.01,11.88) | 0.33 (0.01,11.88) | 0.33 (0.01,11.87) | 0.56 (0.06,5.25) | 0.97 (0.15,6.09) | Exenatide | 1.00 (0.20,4.93) | 1.08 (0.14,8.33) | 1.00 (0.04,24.45) | 1.12 (0.14,9.05) | 1.49 (0.04,53.48) | 1.49 (0.04,53.34) | 1.60 (0.11,22.26) | 3.00 (0.08,107.23) | 3.99 (0.26,60.14) | 3.95 (0.26,59.58) |
| 0.25 (0.03,2.24) | 0.33 (0.01,8.17) | 0.33 (0.01,8.18) | 0.33 (0.01,8.17) | 0.56 (0.12,2.68) | 0.97 (0.39,2.40) | 1.00 (0.20,4.97) | Placebo_or_Control | 1.08 (0.30,3.87) | 1.00 (0.06,15.99) | 1.12 (0.29,4.31) | 1.50 (0.06,36.80) | 1.50 (0.06,36.70) | 1.60 (0.20,13.01) | 3.01 (0.12,73.77) | 4.00 (0.45,35.82) | 3.97 (0.44,35.49) |
| 0.23 (0.02,2.92) | 0.31 (0.01,9.66) | 0.31 (0.01,9.66) | 0.31 (0.01,9.65) | 0.52 (0.07,3.91) | 0.90 (0.19,4.30) | 0.93 (0.12,7.19) | 0.93 (0.26,3.31) | Sitagliptin | 0.93 (0.04,19.56) | 1.04 (0.19,5.84) | 1.39 (0.04,43.49) | 1.38 (0.04,43.38) | 1.48 (0.13,17.21) | 2.78 (0.09,87.20) | 3.71 (0.29,46.76) | 3.67 (0.29,46.32) |
| 0.25 (0.01,8.56) | 0.33 (0.00,22.96) | 0.33 (0.00,22.97) | 0.33 (0.00,22.94) | 0.56 (0.02,13.55) | 0.97 (0.05,17.94) | 1.00 (0.04,24.62) | 1.00 (0.06,15.97) | 1.08 (0.05,22.83) | Lixisenatide | 1.12 (0.05,24.44) | 1.50 (0.02,103.36) | 1.50 (0.02,103.11) | 1.60 (0.05,51.65) | 3.01 (0.04,207.28) | 4.00 (0.12,137.00) | 3.97 (0.12,135.72) |
| 0.22 (0.02,2.91) | 0.30 (0.01,9.53) | 0.30 (0.01,9.53) | 0.30 (0.01,9.52) | 0.50 (0.06,3.93) | 0.87 (0.17,4.37) | 0.89 (0.11,7.21) | 0.89 (0.23,3.41) | 0.96 (0.17,5.40) | 0.89 (0.04,19.36) | Empagliflozin | 1.33 (0.04,42.90) | 1.33 (0.04,42.79) | 1.42 (0.12,17.16) | 2.68 (0.08,86.02) | 3.56 (0.27,46.55) | 3.53 (0.27,46.11) |
| 0.17 (0.00,8.07) | 0.22 (0.00,20.52) | 0.22 (0.00,20.53) | 0.22 (0.00,20.50) | 0.38 (0.01,13.22) | 0.65 (0.02,18.04) | 0.67 (0.02,23.97) | 0.67 (0.03,16.37) | 0.72 (0.02,22.59) | 0.67 (0.01,46.00) | 0.75 (0.02,24.11) | Ertugliflozin | 1.00 (0.01,92.15) | 1.07 (0.02,48.94) | 2.01 (0.02,185.26) | 2.67 (0.06,129.13) | 2.65 (0.05,127.92) |
| 0.17 (0.00,8.08) | 0.22 (0.00,20.56) | 0.22 (0.00,20.57) | 0.22 (0.00,20.55) | 0.38 (0.01,13.24) | 0.65 (0.02,18.07) | 0.67 (0.02,24.01) | 0.67 (0.03,16.40) | 0.72 (0.02,22.63) | 0.67 (0.01,46.09) | 0.75 (0.02,24.16) | 1.00 (0.01,92.56) | Efpeglenatide | 1.07 (0.02,49.04) | 2.01 (0.02,185.63) | 2.68 (0.06,129.38) | 2.65 (0.05,128.17) |
| 0.16 (0.01,3.24) | 0.21 (0.00,9.53) | 0.21 (0.00,9.54) | 0.21 (0.00,9.52) | 0.35 (0.03,4.79) | 0.61 (0.06,5.95) | 0.63 (0.04,8.75) | 0.62 (0.08,5.07) | 0.67 (0.06,7.84) | 0.62 (0.02,20.15) | 0.70 (0.06,8.45) | 0.94 (0.02,42.91) | 0.93 (0.02,42.80) | Semaglutide | 1.88 (0.04,86.05) | 2.50 (0.12,51.82) | 2.48 (0.12,51.34) |
| 0.08 (0.00,4.02) | 0.11 (0.00,10.22) | 0.11 (0.00,10.22) | 0.11 (0.00,10.21) | 0.19 (0.01,6.58) | 0.32 (0.01,8.98) | 0.33 (0.01,11.93) | 0.33 (0.01,8.15) | 0.36 (0.01,11.25) | 0.33 (0.00,22.91) | 0.37 (0.01,12.00) | 0.50 (0.01,46.01) | 0.50 (0.01,45.89) | 0.53 (0.01,24.37) | Vildagliptin | 1.33 (0.03,64.30) | 1.32 (0.03,63.70) |
| 0.06 (0.00,1.38) | 0.08 (0.00,4.02) | 0.08 (0.00,4.02) | 0.08 (0.00,4.02) | 0.14 (0.01,2.07) | 0.24 (0.02,2.60) | 0.25 (0.02,3.78) | 0.25 (0.03,2.23) | 0.27 (0.02,3.40) | 0.25 (0.01,8.55) | 0.28 (0.02,3.67) | 0.37 (0.01,18.10) | 0.37 (0.01,18.06) | 0.40 (0.02,8.29) | 0.75 (0.02,36.30) | Dulaglutide | 0.99 (0.04,21.96) |
| 0.06 (0.00,1.40) | 0.08 (0.00,4.06) | 0.08 (0.00,4.06) | 0.08 (0.00,4.06) | 0.14 (0.01,2.09) | 0.25 (0.02,2.62) | 0.25 (0.02,3.81) | 0.25 (0.03,2.25) | 0.27 (0.02,3.44) | 0.25 (0.01,8.63) | 0.28 (0.02,3.70) | 0.38 (0.01,18.28) | 0.38 (0.01,18.23) | 0.40 (0.02,8.37) | 0.76 (0.02,36.65) | 1.01 (0.05,22.38) | Saxagliptin |

Data presents RR [95%CIs]. Network meta-analysis results are presented as estimate effect sizes for the outcome of chronic lymphocytic leukemia risk. Interventions are reported in order of mean ranking of beneficially prophylactic effect on chronic lymphocytic leukemia risk, and outcomes are expressed as risk ratio (RR) (95% confidence intervals) (95%CIs). For the upper-right portion, RR of less than 1 indicates that the treatment specified in the row got more beneficial effect than that specified in the column. For the lower-left portion, RR of less than 1 indicates that the treatment specified in the column has more beneficial effect than that specified in the row. Bold results marked with * indicate statistical significance.

**eTable 6F: League table of the primary outcome: chronic myeloid leukemia**

| Vildagliptin | 1.00 (0.01,92.02) | 1.26 (0.03,57.51) | 1.75 (0.04,83.91) | 2.78 (0.07,113.82) | 2.97 (0.04,204.46) | 2.98 (0.04,205.22) | 2.99 (0.12,73.33) | 3.00 (0.04,206.93) | 2.99 (0.06,150.57) | 3.05 (0.03,281.04) | 3.10 (0.10,100.28) | 6.30 (0.13,316.81) | 8.98 (0.10,829.45) | 8.97 (0.10,828.09) |
| --- | --- | --- | --- | --- | --- | --- | --- | --- | --- | --- | --- | --- | --- | --- |
| 1.00 (0.01,92.57) | Semaglutide | 1.26 (0.03,57.71) | 1.76 (0.04,84.19) | 2.79 (0.07,114.21) | 2.98 (0.04,205.14) | 2.99 (0.04,205.91) | 3.00 (0.12,73.59) | 3.01 (0.04,207.62) | 3.00 (0.06,151.07) | 3.06 (0.03,281.98) | 3.11 (0.10,100.62) | 6.32 (0.13,317.88) | 9.01 (0.10,832.20) | 9.00 (0.10,830.83) |
| 0.80 (0.02,36.47) | 0.79 (0.02,36.37) | Canagliflozin | 1.40 (0.07,28.58) | 2.21 (0.13,37.01) | 2.36 (0.07,76.24) | 2.37 (0.07,76.52) | 2.38 (0.29,19.35) | 2.39 (0.07,77.16) | 2.38 (0.11,52.01) | 2.43 (0.05,111.08) | 2.47 (0.20,30.00) | 5.01 (0.23,109.43) | 7.15 (0.16,327.89) | 7.14 (0.16,327.35) |
| 0.57 (0.01,27.25) | 0.57 (0.01,27.17) | 0.72 (0.03,14.63) | Sitagliptin | 1.58 (0.09,28.07) | 1.69 (0.05,57.20) | 1.70 (0.05,57.41) | 1.70 (0.19,14.97) | 1.71 (0.05,57.89) | 1.70 (0.07,39.26) | 1.74 (0.04,82.99) | 1.77 (0.14,22.92) | 3.59 (0.16,82.59) | 5.12 (0.11,244.97) | 5.11 (0.11,244.57) |
| 0.36 (0.01,14.73) | 0.36 (0.01,14.69) | 0.45 (0.03,7.55) | 0.63 (0.04,11.19) | Liraglutide | 1.07 (0.04,30.43) | 1.07 (0.04,30.55) | 1.08 (0.16,7.07) | 1.08 (0.04,30.80) | 1.08 (0.06,20.43) | 1.10 (0.03,44.87) | 1.12 (0.11,11.38) | 2.27 (0.12,42.97) | 3.23 (0.08,132.44) | 3.23 (0.08,132.22) |
| 0.34 (0.00,23.23) | 0.34 (0.00,23.17) | 0.42 (0.01,13.66) | 0.59 (0.02,20.02) | 0.94 (0.03,26.73) | Saxagliptin | 1.00 (0.02,50.57) | 1.01 (0.06,16.12) | 1.01 (0.02,51.00) | 1.01 (0.03,36.10) | 1.03 (0.01,70.77) | 1.04 (0.05,22.91) | 2.12 (0.06,75.96) | 3.03 (0.04,208.89) | 3.02 (0.04,208.54) |
| 0.34 (0.00,23.14) | 0.33 (0.00,23.08) | 0.42 (0.01,13.61) | 0.59 (0.02,19.94) | 0.93 (0.03,26.61) | 1.00 (0.02,50.18) | Linagliptin | 1.00 (0.06,16.05) | 1.01 (0.02,50.78) | 1.00 (0.03,35.95) | 1.02 (0.01,70.48) | 1.04 (0.05,22.81) | 2.11 (0.06,75.64) | 3.02 (0.04,208.02) | 3.01 (0.04,207.68) |
| 0.33 (0.01,8.20) | 0.33 (0.01,8.18) | 0.42 (0.05,3.41) | 0.59 (0.07,5.15) | 0.93 (0.14,6.11) | 0.99 (0.06,15.85) | 1.00 (0.06,15.91) | Placebo_or_Control | 1.00 (0.06,16.05) | 1.00 (0.10,9.61) | 1.02 (0.04,24.97) | 1.04 (0.27,4.04) | 2.11 (0.22,20.21) | 3.00 (0.12,73.71) | 3.00 (0.12,73.59) |
| 0.33 (0.00,22.96) | 0.33 (0.00,22.89) | 0.42 (0.01,13.50) | 0.58 (0.02,19.78) | 0.93 (0.03,26.41) | 0.99 (0.02,49.78) | 0.99 (0.02,49.97) | 1.00 (0.06,15.92) | Exenatide | 1.00 (0.03,35.67) | 1.01 (0.01,69.92) | 1.03 (0.05,22.63) | 2.10 (0.06,75.05) | 2.99 (0.04,206.38) | 2.99 (0.04,206.04) |
| 0.33 (0.01,16.83) | 0.33 (0.01,16.78) | 0.42 (0.02,9.17) | 0.59 (0.03,13.51) | 0.93 (0.05,17.64) | 0.99 (0.03,35.51) | 1.00 (0.03,35.64) | 1.00 (0.10,9.61) | 1.00 (0.03,35.94) | Empagliflozin | 1.02 (0.02,51.26) | 1.04 (0.07,14.53) | 2.11 (0.09,51.62) | 3.00 (0.06,151.30) | 3.00 (0.06,151.06) |
| 0.33 (0.00,30.29) | 0.33 (0.00,30.21) | 0.41 (0.01,18.88) | 0.58 (0.01,27.54) | 0.91 (0.02,37.36) | 0.97 (0.01,67.12) | 0.98 (0.01,67.37) | 0.98 (0.04,24.07) | 0.99 (0.01,67.93) | 0.98 (0.02,49.43) | Tirzepatide | 1.02 (0.03,32.92) | 2.07 (0.04,104.00) | 2.95 (0.03,272.28) | 2.94 (0.03,271.83) |
| 0.32 (0.01,10.44) | 0.32 (0.01,10.41) | 0.41 (0.03,4.93) | 0.57 (0.04,7.35) | 0.90 (0.09,9.15) | 0.96 (0.04,20.98) | 0.96 (0.04,21.06) | 0.96 (0.25,3.76) | 0.97 (0.04,21.23) | 0.97 (0.07,13.53) | 0.98 (0.03,31.79) | Dapagliflozin | 2.03 (0.15,28.45) | 2.90 (0.09,93.85) | 2.89 (0.09,93.69) |
| 0.16 (0.00,7.99) | 0.16 (0.00,7.97) | 0.20 (0.01,4.35) | 0.28 (0.01,6.41) | 0.44 (0.02,8.37) | 0.47 (0.01,16.86) | 0.47 (0.01,16.92) | 0.47 (0.05,4.56) | 0.48 (0.01,17.06) | 0.47 (0.02,11.65) | 0.48 (0.01,24.34) | 0.49 (0.04,6.89) | Sotagliflozin | 1.43 (0.03,71.83) | 1.42 (0.03,71.71) |
| 0.11 (0.00,10.28) | 0.11 (0.00,10.25) | 0.14 (0.00,6.41) | 0.20 (0.00,9.35) | 0.31 (0.01,12.68) | 0.33 (0.00,22.77) | 0.33 (0.00,22.86) | 0.33 (0.01,8.17) | 0.33 (0.00,23.05) | 0.33 (0.01,16.77) | 0.34 (0.00,31.30) | 0.35 (0.01,11.17) | 0.70 (0.01,35.29) | Dulaglutide | 1.00 (0.01,92.24) |
| 0.11 (0.00,10.29) | 0.11 (0.00,10.27) | 0.14 (0.00,6.42) | 0.20 (0.00,9.36) | 0.31 (0.01,12.70) | 0.33 (0.00,22.81) | 0.33 (0.00,22.90) | 0.33 (0.01,8.18) | 0.33 (0.00,23.09) | 0.33 (0.01,16.80) | 0.34 (0.00,31.36) | 0.35 (0.01,11.19) | 0.70 (0.01,35.35) | 1.00 (0.01,92.54) | Albiglutide |

Data presents RR [95%CIs]. Network meta-analysis results are presented as estimate effect sizes for the outcome of chronic myeloid leukemia risk. Interventions are reported in order of mean ranking of beneficially prophylactic effect on chronic myeloid leukemia risk, and outcomes are expressed as risk ratio (RR) (95% confidence intervals) (95%CIs). For the upper-right portion, RR of less than 1 indicates that the treatment specified in the row got more beneficial effect than that specified in the column. For the lower-left portion, RR of less than 1 indicates that the treatment specified in the column has more beneficial effect than that specified in the row. Bold results marked with * indicate statistical significance.

**eTable 6G: League table of the primary outcome: lymphoma**

| Tirzepatide | 2.03 (0.20,20.23) | 1.69 (0.05,60.99) | 3.36 (0.39,28.68) | 3.67 (0.65,20.64) | 3.77 (0.56,25.24) | ***5.07 (1.01,25.45)** | 5.61 (0.58,54.37) | ***6.11 (1.03,36.16)** | 6.38 (0.87,46.82) | ***6.41 (1.03,39.97)** | 6.30 (0.98,40.61) | 7.62 (0.21,274.24) | ***6.99 (1.10,44.61)** | 9.18 (0.75,112.62) | 10.67 (0.66,171.84) | ***8.61 (1.38,53.64)** | 12.64 (0.41,393.29) | 15.26 (0.42,549.62) | ***9.36 (1.59,54.99)** | 13.50 (0.62,294.13) | ***11.25 (1.81,69.87)** |
| --- | --- | --- | --- | --- | --- | --- | --- | --- | --- | --- | --- | --- | --- | --- | --- | --- | --- | --- | --- | --- | --- |
| 0.49 (0.05,4.92) | Lixisenatide | 0.84 (0.02,30.42) | 1.66 (0.19,14.42) | 1.81 (0.28,11.58) | 1.86 (0.27,12.72) | 2.50 (0.49,12.87) | 2.76 (0.28,27.30) | 3.01 (0.50,18.35) | 3.15 (0.42,23.58) | 3.16 (0.50,20.16) | 3.11 (0.47,20.47) | 3.76 (0.10,136.80) | 3.45 (0.53,22.49) | 4.53 (0.36,56.45) | 5.26 (0.32,86.00) | 4.25 (0.67,27.05) | 6.23 (0.20,196.28) | 7.53 (0.21,274.17) | 4.62 (0.77,27.75) | 6.66 (0.30,147.27) | 5.55 (0.87,35.23) |
| 0.59 (0.02,21.25) | 1.20 (0.03,43.57) | Vildagliptin | 1.98 (0.06,65.47) | 2.17 (0.08,59.65) | 2.23 (0.08,63.66) | 2.99 (0.12,73.33) | 3.31 (0.09,118.32) | 3.61 (0.13,96.68) | 3.76 (0.12,113.60) | 3.79 (0.14,104.04) | 3.72 (0.13,104.11) | 4.50 (0.05,414.92) | 4.13 (0.15,114.92) | 5.42 (0.13,225.96) | 6.30 (0.13,316.94) | 5.08 (0.18,139.64) | 7.46 (0.09,613.82) | 9.01 (0.10,831.42) | 5.53 (0.21,147.02) | 7.97 (0.13,500.32) | 6.64 (0.24,182.13) |
| 0.30 (0.03,2.54) | 0.60 (0.07,5.25) | 0.50 (0.02,16.66) | Canagliflozin | 1.09 (0.21,5.74) | 1.12 (0.20,6.35) | 1.51 (0.37,6.19) | 1.67 (0.20,14.09) | 1.82 (0.37,9.04) | 1.90 (0.30,11.89) | 1.91 (0.37,9.99) | 1.88 (0.35,10.18) | 2.27 (0.07,74.90) | 2.08 (0.39,11.17) | 2.73 (0.25,29.59) | 3.18 (0.22,45.73) | 2.56 (0.49,13.40) | 3.76 (0.13,107.02) | 4.54 (0.14,150.12) | 2.79 (0.57,13.65) | 4.02 (0.20,79.33) | 3.35 (0.64,17.45) |
| 0.27 (0.05,1.53) | 0.55 (0.09,3.54) | 0.46 (0.02,12.73) | 0.92 (0.17,4.81) | Semaglutide | 1.03 (0.27,3.89) | 1.38 (0.58,3.30) | 1.53 (0.25,9.45) | 1.67 (0.54,5.10) | 1.74 (0.40,7.47) | 1.75 (0.51,5.96) | 1.72 (0.48,6.15) | 2.08 (0.08,57.23) | 1.91 (0.54,6.73) | 2.50 (0.30,20.59) | 2.91 (0.26,32.87) | 2.35 (0.69,8.00) | 3.44 (0.15,81.07) | 4.16 (0.15,114.70) | 2.55 (0.82,7.95) | 3.68 (0.23,58.13) | 3.07 (0.90,10.40) |
| 0.27 (0.04,1.77) | 0.54 (0.08,3.68) | 0.45 (0.02,12.85) | 0.89 (0.16,5.04) | 0.97 (0.26,3.68) | Linagliptin | 1.34 (0.49,3.67) | 1.49 (0.22,9.82) | 1.62 (0.46,5.71) | 1.69 (0.36,7.91) | 1.70 (0.45,6.39) | 1.67 (0.42,6.58) | 2.02 (0.07,57.78) | 1.85 (0.48,7.19) | 2.43 (0.28,21.22) | 2.83 (0.24,33.63) | 2.28 (0.61,8.58) | 3.35 (0.14,82.00) | 4.05 (0.14,115.80) | 2.48 (0.72,8.59) | 3.58 (0.21,59.61) | 2.98 (0.80,11.16) |
| ***0.20 (0.04,0.99)** | 0.40 (0.08,2.06) | 0.33 (0.01,8.20) | 0.66 (0.16,2.72) | 0.72 (0.30,1.73) | 0.74 (0.27,2.03) | Placebo_or_  Control | 1.11 (0.22,5.47) | 1.21 (0.56,2.58) | 1.26 (0.39,4.06) | 1.27 (0.53,3.00) | 1.24 (0.49,3.16) | 1.50 (0.06,36.86) | 1.38 (0.56,3.43) | 1.81 (0.27,12.34) | 2.11 (0.22,20.23) | 1.70 (0.72,4.02) | 2.49 (0.12,51.90) | 3.01 (0.12,73.88) | 1.85 (0.89,3.83) | 2.66 (0.19,36.85) | 2.22 (0.94,5.22) |
| 0.18 (0.02,1.73) | 0.36 (0.04,3.57) | 0.30 (0.01,10.81) | 0.60 (0.07,5.06) | 0.65 (0.11,4.04) | 0.67 (0.10,4.45) | 0.90 (0.18,4.47) | Sotagliflozin | 1.09 (0.19,6.40) | 1.14 (0.16,8.26) | 1.14 (0.19,7.04) | 1.12 (0.18,7.15) | 1.36 (0.04,48.60) | 1.25 (0.20,7.86) | 1.64 (0.13,19.90) | 1.90 (0.12,30.39) | 1.54 (0.25,9.44) | 2.25 (0.07,69.67) | 2.72 (0.08,97.39) | 1.67 (0.29,9.68) | 2.41 (0.11,52.16) | 2.01 (0.33,12.30) |
| ***0.16 (0.03,0.97)** | 0.33 (0.05,2.02) | 0.28 (0.01,7.43) | 0.55 (0.11,2.73) | 0.60 (0.20,1.84) | 0.62 (0.18,2.17) | 0.83 (0.39,1.77) | 0.92 (0.16,5.39) | Sitagliptin | 1.04 (0.27,4.06) | 1.05 (0.33,3.31) | 1.03 (0.31,3.43) | 1.25 (0.05,33.40) | 1.14 (0.35,3.74) | 1.50 (0.19,11.82) | 1.75 (0.16,18.99) | 1.41 (0.45,4.44) | 2.07 (0.09,47.25) | 2.50 (0.09,66.94) | 1.53 (0.53,4.39) | 2.21 (0.16,30.25) | 1.84 (0.59,5.77) |
| 0.16 (0.02,1.15) | 0.32 (0.04,2.38) | 0.27 (0.01,8.01) | 0.53 (0.08,3.30) | 0.58 (0.13,2.47) | 0.59 (0.13,2.77) | 0.79 (0.25,2.56) | 0.88 (0.12,6.38) | 0.96 (0.25,3.73) | Ertugliflozin | 1.01 (0.23,4.30) | 0.99 (0.22,4.41) | 1.19 (0.04,36.04) | 1.10 (0.25,4.83) | 1.44 (0.15,13.62) | 1.67 (0.13,21.37) | 1.35 (0.32,5.78) | 1.98 (0.08,51.28) | 2.39 (0.08,72.23) | 1.47 (0.37,5.83) | 2.12 (0.15,29.00) | 1.76 (0.41,7.52) |
| ***0.16 (0.03,0.97)** | 0.32 (0.05,2.01) | 0.26 (0.01,7.26) | 0.52 (0.10,2.74) | 0.57 (0.17,1.95) | 0.59 (0.16,2.21) | 0.79 (0.33,1.87) | 0.87 (0.14,5.38) | 0.95 (0.30,3.01) | 0.99 (0.23,4.26) | Liraglutide | 0.98 (0.28,3.50) | 1.19 (0.04,32.64) | 1.09 (0.31,3.82) | 1.43 (0.17,11.73) | 1.66 (0.15,18.73) | 1.34 (0.40,4.54) | 1.97 (0.08,46.24) | 2.38 (0.09,65.43) | 1.46 (0.47,4.51) | 2.10 (0.13,33.42) | 1.75 (0.52,5.91) |
| 0.16 (0.02,1.02) | 0.32 (0.05,2.12) | 0.27 (0.01,7.53) | 0.53 (0.10,2.90) | 0.58 (0.16,2.09) | 0.60 (0.15,2.36) | 0.80 (0.32,2.04) | 0.89 (0.14,5.67) | 0.97 (0.29,3.23) | 1.01 (0.23,4.52) | 1.02 (0.29,3.63) | Empagliflozin | 1.21 (0.04,33.88) | 1.11 (0.30,4.09) | 1.46 (0.17,12.30) | 1.69 (0.15,19.57) | 1.37 (0.38,4.86) | 2.01 (0.08,48.03) | 2.42 (0.09,67.90) | 1.49 (0.46,4.85) | 2.14 (0.13,34.81) | 1.79 (0.50,6.33) |
| 0.13 (0.00,4.72) | 0.27 (0.01,9.68) | 0.22 (0.00,20.50) | 0.44 (0.01,14.55) | 0.48 (0.02,13.26) | 0.49 (0.02,14.15) | 0.66 (0.03,16.30) | 0.74 (0.02,26.29) | 0.80 (0.03,21.48) | 0.84 (0.03,25.24) | 0.84 (0.03,23.12) | 0.83 (0.03,23.14) | Bexagliflozin | 0.92 (0.03,25.54) | 1.20 (0.03,50.21) | 1.40 (0.03,70.43) | 1.13 (0.04,31.03) | 1.66 (0.02,136.42) | 2.00 (0.02,184.77) | 1.23 (0.05,32.67) | 1.77 (0.03,111.19) | 1.48 (0.05,40.47) |
| ***0.14 (0.02,0.91)** | 0.29 (0.04,1.89) | 0.24 (0.01,6.74) | 0.48 (0.09,2.58) | 0.52 (0.15,1.85) | 0.54 (0.14,2.09) | 0.72 (0.29,1.80) | 0.80 (0.13,5.05) | 0.87 (0.27,2.86) | 0.91 (0.21,4.02) | 0.92 (0.26,3.21) | 0.90 (0.24,3.31) | 1.09 (0.04,30.32) | Exenatide | 1.31 (0.16,10.97) | 1.53 (0.13,17.48) | 1.23 (0.35,4.31) | 1.81 (0.08,42.98) | 2.18 (0.08,60.77) | 1.34 (0.42,4.29) | 1.93 (0.12,31.12) | 1.61 (0.46,5.61) |
| 0.11 (0.01,1.34) | 0.22 (0.02,2.75) | 0.18 (0.00,7.69) | 0.37 (0.03,3.96) | 0.40 (0.05,3.29) | 0.41 (0.05,3.58) | 0.55 (0.08,3.76) | 0.61 (0.05,7.42) | 0.67 (0.08,5.24) | 0.69 (0.07,6.57) | 0.70 (0.09,5.72) | 0.69 (0.08,5.79) | 0.83 (0.02,34.60) | 0.76 (0.09,6.37) | Albiglutide | 1.16 (0.06,22.57) | 0.94 (0.11,7.68) | 1.38 (0.04,49.92) | 1.66 (0.04,69.34) | 1.02 (0.13,7.94) | 1.47 (0.06,38.03) | 1.23 (0.15,10.01) |
| 0.09 (0.01,1.51) | 0.19 (0.01,3.11) | 0.16 (0.00,7.99) | 0.31 (0.02,4.53) | 0.34 (0.03,3.89) | 0.35 (0.03,4.20) | 0.47 (0.05,4.56) | 0.53 (0.03,8.39) | 0.57 (0.05,6.23) | 0.60 (0.05,7.64) | 0.60 (0.05,6.77) | 0.59 (0.05,6.82) | 0.71 (0.01,35.94) | 0.66 (0.06,7.51) | 0.86 (0.04,16.71) | Alogliptin | 0.81 (0.07,9.09) | 1.18 (0.03,52.22) | 1.43 (0.03,72.03) | 0.88 (0.08,9.45) | 1.27 (0.04,40.55) | 1.05 (0.09,11.84) |
| ***0.12 (0.02,0.72)** | 0.24 (0.04,1.50) | 0.20 (0.01,5.41) | 0.39 (0.07,2.04) | 0.43 (0.13,1.45) | 0.44 (0.12,1.64) | 0.59 (0.25,1.39) | 0.65 (0.11,4.00) | 0.71 (0.23,2.24) | 0.74 (0.17,3.17) | 0.74 (0.22,2.52) | 0.73 (0.21,2.60) | 0.89 (0.03,24.31) | 0.81 (0.23,2.84) | 1.07 (0.13,8.73) | 1.24 (0.11,13.95) | Saxagliptin | 1.47 (0.06,34.44) | 1.77 (0.06,48.73) | 1.09 (0.35,3.36) | 1.57 (0.10,24.89) | 1.31 (0.39,4.40) |
| 0.08 (0.00,2.46) | 0.16 (0.01,5.05) | 0.13 (0.00,11.03) | 0.27 (0.01,7.56) | 0.29 (0.01,6.83) | 0.30 (0.01,7.30) | 0.40 (0.02,8.35) | 0.44 (0.01,13.71) | 0.48 (0.02,11.06) | 0.50 (0.02,13.07) | 0.51 (0.02,11.91) | 0.50 (0.02,11.93) | 0.60 (0.01,49.63) | 0.55 (0.02,13.17) | 0.73 (0.02,26.35) | 0.84 (0.02,37.22) | 0.68 (0.03,15.99) | Efpeglenatide | 1.21 (0.01,99.44) | 0.74 (0.03,16.81) | 1.07 (0.02,59.19) | 0.89 (0.04,20.85) |
| 0.07 (0.00,2.36) | 0.13 (0.00,4.84) | 0.11 (0.00,10.25) | 0.22 (0.01,7.27) | 0.24 (0.01,6.63) | 0.25 (0.01,7.07) | 0.33 (0.01,8.15) | 0.37 (0.01,13.14) | 0.40 (0.01,10.74) | 0.42 (0.01,12.62) | 0.42 (0.02,11.56) | 0.41 (0.01,11.57) | 0.50 (0.01,46.09) | 0.46 (0.02,12.77) | 0.60 (0.01,25.10) | 0.70 (0.01,35.20) | 0.56 (0.02,15.51) | 0.83 (0.01,68.18) | Omarigliptin | 0.61 (0.02,16.33) | 0.88 (0.01,55.57) | 0.74 (0.03,20.23) |
| ***0.11 (0.02,0.63)** | 0.22 (0.04,1.30) | 0.18 (0.01,4.82) | 0.36 (0.07,1.76) | 0.39 (0.13,1.22) | 0.40 (0.12,1.39) | 0.54 (0.26,1.12) | 0.60 (0.10,3.47) | 0.65 (0.23,1.87) | 0.68 (0.17,2.71) | 0.69 (0.22,2.12) | 0.67 (0.21,2.20) | 0.81 (0.03,21.66) | 0.75 (0.23,2.40) | 0.98 (0.13,7.63) | 1.14 (0.11,12.28) | 0.92 (0.30,2.84) | 1.35 (0.06,30.63) | 1.63 (0.06,43.41) | Dapagliflozin | 1.44 (0.09,22.03) | 1.20 (0.39,3.69) |
| 0.07 (0.00,1.61) | 0.15 (0.01,3.32) | 0.13 (0.00,7.88) | 0.25 (0.01,4.91) | 0.27 (0.02,4.29) | 0.28 (0.02,4.65) | 0.38 (0.03,5.19) | 0.42 (0.02,8.99) | 0.45 (0.03,6.20) | 0.47 (0.03,6.47) | 0.48 (0.03,7.54) | 0.47 (0.03,7.58) | 0.56 (0.01,35.43) | 0.52 (0.03,8.35) | 0.68 (0.03,17.59) | 0.79 (0.02,25.32) | 0.64 (0.04,10.12) | 0.94 (0.02,51.85) | 1.13 (0.02,71.00) | 0.69 (0.05,10.59) | Ertugliflozin_  Sitagliptin | 0.83 (0.05,13.20) |
| ***0.09 (0.01,0.55)** | 0.18 (0.03,1.14) | 0.15 (0.01,4.13) | 0.30 (0.06,1.56) | 0.33 (0.10,1.11) | 0.34 (0.09,1.25) | 0.45 (0.19,1.06) | 0.50 (0.08,3.06) | 0.54 (0.17,1.71) | 0.57 (0.13,2.42) | 0.57 (0.17,1.92) | 0.56 (0.16,1.98) | 0.68 (0.02,18.58) | 0.62 (0.18,2.17) | 0.82 (0.10,6.67) | 0.95 (0.08,10.65) | 0.77 (0.23,2.58) | 1.12 (0.05,26.31) | 1.36 (0.05,37.24) | 0.83 (0.27,2.56) | 1.20 (0.08,19.01) | Dulaglutide |

Data presents RR [95%CIs]. Network meta-analysis results are presented as estimate effect sizes for the outcome of lymphoma risk. Interventions are reported in order of mean ranking of beneficially prophylactic effect on lymphoma risk, and outcomes are expressed as risk ratio (RR) (95% confidence intervals) (95%CIs). For the upper-right portion, RR of less than 1 indicates that the treatment specified in the row got more beneficial effect than that specified in the column. For the lower-left portion, RR of less than 1 indicates that the treatment specified in the column has more beneficial effect than that specified in the row. Bold results marked with * indicate statistical significance.

**eTable 6H: League table of the primary outcome: B cell non-Hodgkin's lymphoma**

| Tirzepatide | 1.98 (0.04,99.29) | 3.15 (0.11,90.24) | 1.98 (0.02,182.52) | 3.46 (0.09,132.71) | 4.18 (0.11,155.31) | 5.12 (0.17,150.62) | 5.39 (0.17,170.88) | 5.92 (0.24,145.00) | 6.26 (0.21,189.76) | 7.48 (0.23,245.62) | 8.91 (0.10,820.84) | 7.96 (0.22,293.75) | 8.86 (0.10,817.10) | 11.36 (0.18,737.46) | 9.71 (0.34,279.43) | 10.73 (0.26,446.89) | 10.41 (0.34,319.86) | 11.75 (0.31,438.83) | 17.79 (0.60,528.83) |
| --- | --- | --- | --- | --- | --- | --- | --- | --- | --- | --- | --- | --- | --- | --- | --- | --- | --- | --- | --- |
| 0.51 (0.01,25.45) | Lixisenatide | 1.59 (0.13,19.05) | 1.00 (0.02,50.47) | 1.75 (0.10,30.65) | 2.12 (0.13,35.56) | 2.59 (0.21,32.07) | 2.73 (0.20,37.31) | 3.00 (0.31,28.81) | 3.17 (0.25,40.82) | 3.79 (0.26,54.22) | 4.51 (0.09,226.94) | 4.03 (0.24,67.14) | 4.49 (0.09,225.93) | 5.75 (0.17,192.04) | 4.92 (0.41,59.05) | 5.43 (0.28,105.52) | 5.27 (0.40,69.14) | 5.95 (0.35,100.64) | 9.01 (0.72,113.02) |
| 0.32 (0.01,9.10) | 0.63 (0.05,7.50) | Semaglutide | 0.63 (0.02,18.06) | 1.10 (0.15,8.34) | 1.33 (0.19,9.51) | 1.63 (0.40,6.69) | 1.71 (0.33,8.93) | 1.88 (0.68,5.20) | 1.99 (0.42,9.50) | 2.38 (0.42,13.42) | 2.83 (0.10,81.22) | 2.53 (0.36,17.89) | 2.82 (0.10,80.87) | 3.61 (0.21,62.02) | 3.09 (0.73,13.11) | 3.41 (0.39,29.90) | 3.31 (0.67,16.29) | 3.73 (0.52,27.00) | ***5.65 (1.23,25.87)** |
| 0.50 (0.01,46.53) | 1.00 (0.02,50.20) | 1.59 (0.06,45.64) | Vildagliptin | 1.75 (0.05,67.10) | 2.11 (0.06,78.53) | 2.59 (0.09,76.17) | 2.72 (0.09,86.41) | 2.99 (0.12,73.33) | 3.16 (0.10,95.96) | 3.78 (0.11,124.20) | 4.50 (0.05,414.92) | 4.02 (0.11,148.53) | 4.47 (0.05,413.03) | 5.74 (0.09,372.81) | 4.90 (0.17,141.30) | 5.42 (0.13,225.96) | 5.25 (0.17,161.75) | 5.93 (0.16,221.89) | 8.98 (0.30,267.42) |
| 0.29 (0.01,11.07) | 0.57 (0.03,9.97) | 0.91 (0.12,6.89) | 0.57 (0.01,21.95) | Sotagliflozin | 1.21 (0.11,13.72) | 1.48 (0.19,11.69) | 1.56 (0.17,13.88) | 1.71 (0.30,9.86) | 1.81 (0.22,15.01) | 2.16 (0.23,20.35) | 2.57 (0.07,98.70) | 2.30 (0.20,25.87) | 2.56 (0.07,98.26) | 3.28 (0.13,80.68) | 2.81 (0.37,21.38) | 3.10 (0.23,41.63) | 3.00 (0.35,25.52) | 3.39 (0.30,38.88) | 5.14 (0.64,41.33) |
| 0.24 (0.01,8.87) | 0.47 (0.03,7.92) | 0.75 (0.11,5.38) | 0.47 (0.01,17.59) | 0.83 (0.07,9.40) | Canagliflozin | 1.22 (0.16,9.14) | 1.29 (0.15,10.89) | 1.42 (0.26,7.63) | 1.50 (0.19,11.75) | 1.79 (0.20,15.98) | 2.13 (0.06,79.11) | 1.90 (0.18,20.40) | 2.12 (0.06,78.77) | 2.72 (0.11,64.38) | 2.32 (0.32,16.70) | 2.56 (0.20,32.93) | 2.49 (0.31,19.99) | 2.81 (0.26,30.67) | 4.25 (0.56,32.33) |
| 0.20 (0.01,5.74) | 0.39 (0.03,4.77) | 0.61 (0.15,2.53) | 0.39 (0.01,11.38) | 0.68 (0.09,5.34) | 0.82 (0.11,6.10) | Sitagliptin | 1.05 (0.21,5.37) | 1.16 (0.39,3.47) | 1.22 (0.24,6.16) | 1.46 (0.25,8.66) | 1.74 (0.06,51.18) | 1.55 (0.21,11.48) | 1.73 (0.06,50.96) | 2.22 (0.16,31.57) | 1.90 (0.42,8.53) | 2.09 (0.23,19.10) | 2.03 (0.39,10.55) | 2.29 (0.30,17.31) | 3.47 (0.72,16.79) |
| 0.19 (0.01,5.88) | 0.37 (0.03,5.01) | 0.58 (0.11,3.05) | 0.37 (0.01,11.66) | 0.64 (0.07,5.73) | 0.78 (0.09,6.56) | 0.95 (0.19,4.85) | Ertugliflozin | 1.10 (0.30,4.08) | 1.16 (0.20,6.82) | 1.39 (0.20,9.46) | 1.65 (0.05,52.45) | 1.48 (0.18,12.36) | 1.64 (0.05,52.22) | 2.11 (0.15,30.01) | 1.80 (0.34,9.54) | 1.99 (0.19,20.34) | 1.93 (0.32,11.64) | 2.18 (0.26,18.62) | 3.30 (0.58,18.65) |
| 0.17 (0.01,4.13) | 0.33 (0.03,3.20) | 0.53 (0.19,1.47) | 0.33 (0.01,8.20) | 0.58 (0.10,3.37) | 0.71 (0.13,3.81) | 0.86 (0.29,2.59) | 0.91 (0.25,3.38) | Placebo_or_Control | 1.06 (0.32,3.47) | 1.26 (0.31,5.13) | 1.50 (0.06,36.86) | 1.34 (0.25,7.15) | 1.50 (0.06,36.70) | 1.92 (0.13,28.00) | 1.64 (0.59,4.59) | 1.81 (0.27,12.34) | 1.76 (0.51,6.00) | 1.98 (0.36,10.83) | 3.00 (0.97,9.31) |
| 0.16 (0.01,4.84) | 0.32 (0.02,4.06) | 0.50 (0.11,2.40) | 0.32 (0.01,9.60) | 0.55 (0.07,4.59) | 0.67 (0.09,5.24) | 0.82 (0.16,4.12) | 0.86 (0.15,5.05) | 0.95 (0.29,3.10) | Liraglutide | 1.20 (0.19,7.49) | 1.42 (0.05,43.15) | 1.27 (0.16,9.87) | 1.42 (0.05,42.96) | 1.81 (0.10,34.05) | 1.55 (0.32,7.46) | 1.71 (0.18,16.36) | 1.66 (0.30,9.17) | 1.88 (0.24,14.89) | 2.84 (0.55,14.64) |
| 0.13 (0.00,4.38) | 0.26 (0.02,3.78) | 0.42 (0.07,2.37) | 0.26 (0.01,8.70) | 0.46 (0.05,4.36) | 0.56 (0.06,5.00) | 0.68 (0.12,4.06) | 0.72 (0.11,4.91) | 0.79 (0.20,3.21) | 0.84 (0.13,5.25) | Empagliflozin | 1.19 (0.04,39.11) | 1.06 (0.12,9.41) | 1.18 (0.04,38.94) | 1.52 (0.07,31.25) | 1.30 (0.23,7.38) | 1.43 (0.13,15.42) | 1.39 (0.22,8.95) | 1.57 (0.17,14.17) | 2.38 (0.39,14.38) |
| 0.11 (0.00,10.34) | 0.22 (0.00,11.16) | 0.35 (0.01,10.14) | 0.22 (0.00,20.50) | 0.39 (0.01,14.91) | 0.47 (0.01,17.45) | 0.58 (0.02,16.93) | 0.61 (0.02,19.20) | 0.66 (0.03,16.30) | 0.70 (0.02,21.32) | 0.84 (0.03,27.60) | Bexagliflozin | 0.89 (0.02,33.01) | 0.99 (0.01,91.79) | 1.28 (0.02,82.85) | 1.09 (0.04,31.40) | 1.20 (0.03,50.21) | 1.17 (0.04,35.94) | 1.32 (0.04,49.31) | 2.00 (0.07,59.43) |
| 0.13 (0.00,4.63) | 0.25 (0.01,4.13) | 0.40 (0.06,2.80) | 0.25 (0.01,9.19) | 0.43 (0.04,4.90) | 0.53 (0.05,5.64) | 0.64 (0.09,4.75) | 0.68 (0.08,5.66) | 0.74 (0.14,3.96) | 0.79 (0.10,6.11) | 0.94 (0.11,8.32) | 1.12 (0.03,41.33) | Linagliptin | 1.11 (0.03,41.15) | 1.43 (0.06,33.60) | 1.22 (0.17,8.68) | 1.35 (0.11,17.16) | 1.31 (0.16,10.40) | 1.48 (0.14,15.97) | 2.23 (0.30,16.81) |
| 0.11 (0.00,10.40) | 0.22 (0.00,11.22) | 0.36 (0.01,10.20) | 0.22 (0.00,20.63) | 0.39 (0.01,15.00) | 0.47 (0.01,17.56) | 0.58 (0.02,17.03) | 0.61 (0.02,19.32) | 0.67 (0.03,16.40) | 0.71 (0.02,21.45) | 0.84 (0.03,27.77) | 1.01 (0.01,92.76) | 0.90 (0.02,33.21) | Efpeglenatide | 1.28 (0.02,83.35) | 1.10 (0.04,31.59) | 1.21 (0.03,50.52) | 1.17 (0.04,36.16) | 1.33 (0.04,49.61) | 2.01 (0.07,59.79) |
| 0.09 (0.00,5.71) | 0.17 (0.01,5.80) | 0.28 (0.02,4.76) | 0.17 (0.00,11.33) | 0.30 (0.01,7.49) | 0.37 (0.02,8.73) | 0.45 (0.03,6.42) | 0.47 (0.03,6.75) | 0.52 (0.04,7.61) | 0.55 (0.03,10.34) | 0.66 (0.03,13.56) | 0.78 (0.01,50.94) | 0.70 (0.03,16.50) | 0.78 (0.01,50.71) | Ertugliflozin_Sitagliptin | 0.85 (0.05,15.10) | 0.94 (0.03,25.52) | 0.92 (0.05,17.48) | 1.03 (0.04,24.69) | 1.57 (0.09,28.73) |
| 0.10 (0.00,2.96) | 0.20 (0.02,2.44) | 0.32 (0.08,1.38) | 0.20 (0.01,5.87) | 0.36 (0.05,2.72) | 0.43 (0.06,3.10) | 0.53 (0.12,2.37) | 0.55 (0.10,2.94) | 0.61 (0.22,1.71) | 0.64 (0.13,3.10) | 0.77 (0.14,4.38) | 0.92 (0.03,26.41) | 0.82 (0.12,5.83) | 0.91 (0.03,26.30) | 1.17 (0.07,20.66) | Dapagliflozin | 1.10 (0.13,9.74) | 1.07 (0.22,5.32) | 1.21 (0.17,8.80) | 1.83 (0.40,8.45) |
| 0.09 (0.00,3.88) | 0.18 (0.01,3.57) | 0.29 (0.03,2.57) | 0.18 (0.00,7.69) | 0.32 (0.02,4.33) | 0.39 (0.03,5.01) | 0.48 (0.05,4.35) | 0.50 (0.05,5.13) | 0.55 (0.08,3.76) | 0.58 (0.06,5.57) | 0.70 (0.06,7.50) | 0.83 (0.02,34.60) | 0.74 (0.06,9.44) | 0.83 (0.02,34.45) | 1.06 (0.04,28.60) | 0.91 (0.10,7.98) | Albiglutide | 0.97 (0.10,9.46) | 1.09 (0.08,14.18) | 1.66 (0.18,15.37) |
| 0.10 (0.00,2.95) | 0.19 (0.01,2.49) | 0.30 (0.06,1.49) | 0.19 (0.01,5.86) | 0.33 (0.04,2.83) | 0.40 (0.05,3.23) | 0.49 (0.09,2.56) | 0.52 (0.09,3.12) | 0.57 (0.17,1.94) | 0.60 (0.11,3.32) | 0.72 (0.11,4.63) | 0.86 (0.03,26.35) | 0.77 (0.10,6.09) | 0.85 (0.03,26.23) | 1.09 (0.06,20.84) | 0.93 (0.19,4.63) | 1.03 (0.11,10.06) | Exenatide | 1.13 (0.14,9.17) | 1.71 (0.32,9.08) |
| 0.09 (0.00,3.18) | 0.17 (0.01,2.84) | 0.27 (0.04,1.94) | 0.17 (0.00,6.30) | 0.29 (0.03,3.38) | 0.36 (0.03,3.89) | 0.44 (0.06,3.29) | 0.46 (0.05,3.92) | 0.50 (0.09,2.75) | 0.53 (0.07,4.23) | 0.64 (0.07,5.75) | 0.76 (0.02,28.35) | 0.68 (0.06,7.33) | 0.75 (0.02,28.22) | 0.97 (0.04,23.09) | 0.83 (0.11,6.01) | 0.91 (0.07,11.83) | 0.89 (0.11,7.19) | Saxagliptin | 1.51 (0.20,11.64) |
| 0.06 (0.00,1.67) | 0.11 (0.01,1.39) | ***0.18 (0.04,0.81)** | 0.11 (0.00,3.31) | 0.19 (0.02,1.57) | 0.24 (0.03,1.79) | 0.29 (0.06,1.39) | 0.30 (0.05,1.71) | 0.33 (0.11,1.03) | 0.35 (0.07,1.81) | 0.42 (0.07,2.54) | 0.50 (0.02,14.90) | 0.45 (0.06,3.37) | 0.50 (0.02,14.83) | 0.64 (0.03,11.72) | 0.55 (0.12,2.52) | 0.60 (0.07,5.59) | 0.58 (0.11,3.11) | 0.66 (0.09,5.08) | Dulaglutide |

Data presents RR [95%CIs]. Network meta-analysis results are presented as estimate effect sizes for the outcome of B cell non-Hodgkin's lymphoma risk. Interventions are reported in order of mean ranking of beneficially prophylactic effect on B cell non-Hodgkin's lymphoma risk, and outcomes are expressed as risk ratio (RR) (95% confidence intervals) (95%CIs). For the upper-right portion, RR of less than 1 indicates that the treatment specified in the row got more beneficial effect than that specified in the column. For the lower-left portion, RR of less than 1 indicates that the treatment specified in the column has more beneficial effect than that specified in the row. Bold results marked with * indicate statistical significance.

**eTable 6I: League table of the primary outcome: T cell non-Hodgkin's lymphoma**

| Sitagliptin | 7.03 (0.16,317.24) | 6.99 (0.27,182.42) | 6.98 (0.12,415.16) | 10.46 (0.09,1231.34) | 21.06 (0.18,2479.28) |
| --- | --- | --- | --- | --- | --- |
| 0.14 (0.00,6.42) | Dapagliflozin | 1.00 (0.14,7.13) | 0.99 (0.04,23.21) | 1.49 (0.03,81.03) | 3.00 (0.06,163.16) |
| 0.14 (0.01,3.73) | 1.01 (0.14,7.20) | Placebo_or_Control | 1.00 (0.09,11.69) | 1.50 (0.05,48.48) | 3.01 (0.09,97.62) |
| 0.14 (0.00,8.51) | 1.01 (0.04,23.49) | 1.00 (0.09,11.71) | Empagliflozin | 1.50 (0.02,106.05) | 3.01 (0.04,213.54) |
| 0.10 (0.00,11.25) | 0.67 (0.01,36.56) | 0.67 (0.02,21.66) | 0.67 (0.01,47.27) | Efpeglenatide | 2.01 (0.01,275.55) |
| 0.05 (0.00,5.59) | 0.33 (0.01,18.17) | 0.33 (0.01,10.76) | 0.33 (0.00,23.50) | 0.50 (0.00,68.01) | Exenatide |

Data presents RR [95%CIs]. Network meta-analysis results are presented as estimate effect sizes for the outcome of T cell non-Hodgkin's lymphoma risk. Interventions are reported in order of mean ranking of beneficially prophylactic effect on T cell non-Hodgkin's lymphoma risk, and outcomes are expressed as risk ratio (RR) (95% confidence intervals) (95%CIs). For the upper-right portion, RR of less than 1 indicates that the treatment specified in the row got more beneficial effect than that specified in the column. For the lower-left portion, RR of less than 1 indicates that the treatment specified in the column has more beneficial effect than that specified in the row. Bold results marked with * indicate statistical significance.

**eTable 6J: League table of the primary outcome: myeloma**

| Tirzepatide | 2.37 (0.06,93.69) | 2.94 (0.06,148.14) | 3.85 (0.12,128.53) | 4.86 (0.15,154.68) | 4.46 (0.09,224.38) | 5.53 (0.19,160.13) | 7.01 (0.21,228.57) | 6.85 (0.20,232.73) | 8.84 (0.36,216.65) | 9.44 (0.21,431.76) | 9.48 (0.31,285.78) | 10.29 (0.33,324.71) | 26.61 (0.29,2456.33) | 26.50 (0.29,2446.49) | 26.52 (0.29,2448.09) | 26.30 (0.28,2427.27) | 22.93 (0.69,762.76) |
| --- | --- | --- | --- | --- | --- | --- | --- | --- | --- | --- | --- | --- | --- | --- | --- | --- | --- |
| 0.42 (0.01,16.72) | Sitagliptin | 1.24 (0.07,22.61) | 1.63 (0.16,16.48) | 2.05 (0.22,19.35) | 1.88 (0.10,34.23) | 2.33 (0.29,18.97) | 2.96 (0.40,22.09) | 2.89 (0.28,30.11) | 3.73 (0.61,22.92) | 3.99 (0.25,63.63) | 4.00 (0.46,34.66) | 4.35 (0.47,40.40) | 11.24 (0.28,445.10) | 11.19 (0.28,443.34) | 11.20 (0.28,443.62) | 11.11 (0.28,439.84) | 9.68 (0.96,97.64) |
| 0.34 (0.01,17.10) | 0.80 (0.04,14.63) | Canagliflozin | 1.31 (0.09,19.11) | 1.65 (0.12,22.66) | 1.52 (0.06,37.16) | 1.88 (0.16,22.73) | 2.38 (0.17,33.74) | 2.33 (0.16,34.79) | 3.00 (0.31,28.86) | 3.21 (0.15,69.94) | 3.22 (0.25,41.11) | 3.50 (0.26,47.42) | 9.04 (0.18,455.39) | 9.00 (0.18,453.58) | 9.01 (0.18,453.87) | 8.93 (0.18,450.01) | 7.79 (0.54,113.29) |
| 0.26 (0.01,8.66) | 0.61 (0.06,6.22) | 0.76 (0.05,11.15) | Linagliptin | 1.26 (0.18,8.88) | 1.16 (0.08,16.89) | 1.43 (0.24,8.50) | 1.82 (0.25,13.36) | 1.78 (0.23,14.01) | 2.29 (0.54,9.66) | 2.45 (0.19,31.05) | 2.46 (0.39,15.69) | 2.67 (0.39,18.50) | 6.91 (0.21,230.67) | 6.88 (0.21,229.76) | 6.88 (0.21,229.90) | 6.83 (0.20,227.94) | 5.95 (0.78,45.24) |
| 0.21 (0.01,6.56) | 0.49 (0.05,4.60) | 0.61 (0.04,8.32) | 0.79 (0.11,5.59) | Liraglutide | 0.92 (0.07,12.60) | 1.14 (0.21,6.14) | 1.44 (0.21,9.75) | 1.41 (0.19,10.26) | 1.82 (0.49,6.81) | 1.94 (0.16,23.09) | 1.95 (0.38,9.95) | 2.12 (0.33,13.47) | 5.48 (0.17,174.66) | 5.46 (0.17,173.97) | 5.46 (0.17,174.08) | 5.41 (0.17,172.59) | 4.72 (0.67,33.08) |
| 0.22 (0.00,11.27) | 0.53 (0.03,9.64) | 0.66 (0.03,16.17) | 0.86 (0.06,12.59) | 1.09 (0.08,14.93) | Ertugliflozin | 1.24 (0.10,14.98) | 1.57 (0.11,22.24) | 1.54 (0.10,22.93) | 1.98 (0.21,19.01) | 2.11 (0.10,46.10) | 2.13 (0.17,27.09) | 2.31 (0.17,31.25) | 5.97 (0.12,300.21) | 5.94 (0.12,299.02) | 5.94 (0.12,299.21) | 5.89 (0.12,296.66) | 5.14 (0.35,74.66) |
| 0.18 (0.01,5.24) | 0.43 (0.05,3.48) | 0.53 (0.04,6.44) | 0.70 (0.12,4.13) | 0.88 (0.16,4.74) | 0.81 (0.07,9.76) | Dapagliflozin | 1.27 (0.22,7.18) | 1.24 (0.20,7.60) | 1.60 (0.56,4.56) | 1.71 (0.16,17.74) | 1.72 (0.36,8.24) | 1.86 (0.35,9.84) | 4.82 (0.17,139.60) | 4.79 (0.17,139.05) | 4.80 (0.17,139.13) | 4.76 (0.16,137.95) | 4.15 (0.70,24.42) |
| 0.14 (0.00,4.66) | 0.34 (0.05,2.52) | 0.42 (0.03,5.95) | 0.55 (0.07,4.04) | 0.69 (0.10,4.69) | 0.64 (0.04,9.01) | 0.79 (0.14,4.46) | Semaglutide | 0.98 (0.13,7.41) | 1.26 (0.32,5.02) | 1.35 (0.11,16.54) | 1.35 (0.22,8.26) | 1.47 (0.22,9.76) | 3.80 (0.12,123.96) | 3.78 (0.12,123.47) | 3.78 (0.12,123.55) | 3.75 (0.11,122.50) | 3.27 (0.45,23.91) |
| 0.15 (0.00,4.96) | 0.35 (0.03,3.59) | 0.43 (0.03,6.42) | 0.56 (0.07,4.43) | 0.71 (0.10,5.15) | 0.65 (0.04,9.72) | 0.81 (0.13,4.95) | 1.02 (0.13,7.75) | Saxagliptin | 1.29 (0.29,5.67) | 1.38 (0.11,17.88) | 1.38 (0.21,9.12) | 1.50 (0.21,10.74) | 3.88 (0.11,131.99) | 3.87 (0.11,131.47) | 3.87 (0.11,131.55) | 3.84 (0.11,130.43) | 3.35 (0.43,26.22) |
| 0.11 (0.00,2.77) | 0.27 (0.04,1.64) | 0.33 (0.03,3.20) | 0.44 (0.10,1.84) | 0.55 (0.15,2.06) | 0.50 (0.05,4.85) | 0.63 (0.22,1.78) | 0.79 (0.20,3.16) | 0.78 (0.18,3.41) | Placebo_or_Control | 1.07 (0.13,8.66) | 1.07 (0.33,3.45) | 1.16 (0.32,4.25) | 3.01 (0.12,73.88) | 3.00 (0.12,73.59) | 3.00 (0.12,73.64) | 2.98 (0.12,73.01) | 2.59 (0.62,10.85) |
| 0.11 (0.00,4.85) | 0.25 (0.02,4.01) | 0.31 (0.01,6.81) | 0.41 (0.03,5.18) | 0.51 (0.04,6.12) | 0.47 (0.02,10.31) | 0.59 (0.06,6.09) | 0.74 (0.06,9.12) | 0.73 (0.06,9.43) | 0.94 (0.12,7.60) | Sotagliflozin | 1.00 (0.09,11.05) | 1.09 (0.09,12.79) | 2.82 (0.06,129.14) | 2.81 (0.06,128.63) | 2.81 (0.06,128.71) | 2.79 (0.06,127.62) | 2.43 (0.19,30.68) |
| 0.11 (0.00,3.18) | 0.25 (0.03,2.16) | 0.31 (0.02,3.96) | 0.41 (0.06,2.59) | 0.51 (0.10,2.61) | 0.47 (0.04,6.00) | 0.58 (0.12,2.80) | 0.74 (0.12,4.51) | 0.72 (0.11,4.76) | 0.93 (0.29,3.00) | 1.00 (0.09,10.94) | Exenatide | 1.09 (0.19,6.21) | 2.81 (0.09,84.66) | 2.80 (0.09,84.33) | 2.80 (0.09,84.38) | 2.77 (0.09,83.66) | 2.42 (0.38,15.33) |
| 0.10 (0.00,3.06) | 0.23 (0.02,2.14) | 0.29 (0.02,3.88) | 0.37 (0.05,2.59) | 0.47 (0.07,3.00) | 0.43 (0.03,5.87) | 0.54 (0.10,2.84) | 0.68 (0.10,4.52) | 0.67 (0.09,4.76) | 0.86 (0.24,3.13) | 0.92 (0.08,10.74) | 0.92 (0.16,5.27) | Empagliflozin | 2.59 (0.08,81.59) | 2.57 (0.08,81.27) | 2.58 (0.08,81.32) | 2.55 (0.08,80.62) | 2.23 (0.32,15.33) |
| 0.04 (0.00,3.47) | 0.09 (0.00,3.52) | 0.11 (0.00,5.57) | 0.14 (0.00,4.83) | 0.18 (0.01,5.82) | 0.17 (0.00,8.44) | 0.21 (0.01,6.02) | 0.26 (0.01,8.59) | 0.26 (0.01,8.75) | 0.33 (0.01,8.15) | 0.35 (0.01,16.23) | 0.36 (0.01,10.75) | 0.39 (0.01,12.21) | Omarigliptin | 1.00 (0.01,91.98) | 1.00 (0.01,92.04) | 0.99 (0.01,91.25) | 0.86 (0.03,28.68) |
| 0.04 (0.00,3.48) | 0.09 (0.00,3.54) | 0.11 (0.00,5.59) | 0.15 (0.00,4.85) | 0.18 (0.01,5.84) | 0.17 (0.00,8.47) | 0.21 (0.01,6.05) | 0.26 (0.01,8.63) | 0.26 (0.01,8.79) | 0.33 (0.01,8.18) | 0.36 (0.01,16.31) | 0.36 (0.01,10.79) | 0.39 (0.01,12.26) | 1.00 (0.01,92.76) | Albiglutide | 1.00 (0.01,92.45) | 0.99 (0.01,91.66) | 0.87 (0.03,28.81) |
| 0.04 (0.00,3.48) | 0.09 (0.00,3.54) | 0.11 (0.00,5.59) | 0.15 (0.00,4.85) | 0.18 (0.01,5.84) | 0.17 (0.00,8.47) | 0.21 (0.01,6.04) | 0.26 (0.01,8.63) | 0.26 (0.01,8.78) | 0.33 (0.01,8.18) | 0.36 (0.01,16.29) | 0.36 (0.01,10.78) | 0.39 (0.01,12.25) | 1.00 (0.01,92.68) | 1.00 (0.01,92.31) | Lixisenatide | 0.99 (0.01,91.58) | 0.86 (0.03,28.78) |
| 0.04 (0.00,3.51) | 0.09 (0.00,3.57) | 0.11 (0.00,5.64) | 0.15 (0.00,4.89) | 0.18 (0.01,5.89) | 0.17 (0.00,8.54) | 0.21 (0.01,6.09) | 0.27 (0.01,8.70) | 0.26 (0.01,8.86) | 0.34 (0.01,8.25) | 0.36 (0.01,16.43) | 0.36 (0.01,10.88) | 0.39 (0.01,12.36) | 1.01 (0.01,93.47) | 1.01 (0.01,93.09) | 1.01 (0.01,93.15) | Alogliptin | 0.87 (0.03,29.03) |
| 0.04 (0.00,1.45) | 0.10 (0.01,1.04) | 0.13 (0.01,1.87) | 0.17 (0.02,1.28) | 0.21 (0.03,1.48) | 0.19 (0.01,2.83) | 0.24 (0.04,1.42) | 0.31 (0.04,2.23) | 0.30 (0.04,2.34) | 0.39 (0.09,1.61) | 0.41 (0.03,5.20) | 0.41 (0.07,2.62) | 0.45 (0.07,3.09) | 1.16 (0.03,38.64) | 1.16 (0.03,38.49) | 1.16 (0.03,38.52) | 1.15 (0.03,38.19) | Dulaglutide |

Data presents RR [95%CIs]. Network meta-analysis results are presented as estimate effect sizes for the outcome of myeloma risk. Interventions are reported in order of mean ranking of beneficially prophylactic effect on myeloma risk, and outcomes are expressed as risk ratio (RR) (95% confidence intervals) (95%CIs). For the upper-right portion, RR of less than 1 indicates that the treatment specified in the row got more beneficial effect than that specified in the column. For the lower-left portion, RR of less than 1 indicates that the treatment specified in the column has more beneficial effect than that specified in the row. Bold results marked with * indicate statistical significance.

**eTable 6K: League table of the primary outcome: plasma cell myeloma**

| Tirzepatide | 2.37 (0.06,93.69) | 2.94 (0.06,148.14) | 3.85 (0.12,128.53) | 2.89 (0.03,265.87) | 4.57 (0.15,135.03) | 4.46 (0.09,224.38) | 5.31 (0.16,176.57) | 7.01 (0.21,228.57) | 8.87 (0.29,274.16) | 8.84 (0.36,216.65) | 9.44 (0.21,431.76) | 10.29 (0.33,324.71) | 26.30 (0.28,2427.27) | 26.50 (0.29,2446.49) | 26.52 (0.29,2448.09) | 26.61 (0.29,2456.33) | 26.54 (0.74,949.39) |
| --- | --- | --- | --- | --- | --- | --- | --- | --- | --- | --- | --- | --- | --- | --- | --- | --- | --- |
| 0.42 (0.01,16.72) | Sitagliptin | 1.24 (0.07,22.61) | 1.63 (0.16,16.48) | 1.22 (0.03,48.14) | 1.93 (0.23,16.19) | 1.88 (0.10,34.23) | 2.24 (0.22,22.60) | 2.96 (0.40,22.09) | 3.75 (0.42,33.73) | 3.73 (0.61,22.92) | 3.99 (0.25,63.63) | 4.35 (0.47,40.40) | 11.11 (0.28,439.84) | 11.19 (0.28,443.34) | 11.20 (0.28,443.62) | 11.24 (0.28,445.10) | 11.21 (1.00,125.98) |
| 0.34 (0.01,17.10) | 0.80 (0.04,14.63) | Canagliflozin | 1.31 (0.09,19.11) | 0.98 (0.02,49.26) | 1.55 (0.12,19.30) | 1.52 (0.06,37.16) | 1.80 (0.12,26.23) | 2.38 (0.17,33.74) | 3.01 (0.23,39.77) | 3.00 (0.31,28.86) | 3.21 (0.15,69.94) | 3.50 (0.26,47.42) | 8.93 (0.18,450.01) | 9.00 (0.18,453.58) | 9.01 (0.18,453.87) | 9.04 (0.18,455.39) | 9.02 (0.56,144.11) |
| 0.26 (0.01,8.66) | 0.61 (0.06,6.22) | 0.76 (0.05,11.15) | Linagliptin | 0.75 (0.02,24.94) | 1.19 (0.19,7.29) | 1.16 (0.08,16.89) | 1.38 (0.18,10.47) | 1.82 (0.25,13.36) | 2.30 (0.35,15.37) | 2.29 (0.54,9.66) | 2.45 (0.19,31.05) | 2.67 (0.39,18.50) | 6.83 (0.20,227.94) | 6.88 (0.21,229.76) | 6.88 (0.21,229.90) | 6.91 (0.21,230.67) | 6.89 (0.80,59.21) |
| 0.35 (0.00,31.81) | 0.82 (0.02,32.29) | 1.02 (0.02,51.06) | 1.33 (0.04,44.28) | Saxagliptin | 1.58 (0.05,46.52) | 1.54 (0.03,77.34) | 1.84 (0.06,60.83) | 2.42 (0.07,78.75) | 3.07 (0.10,94.45) | 3.06 (0.13,74.62) | 3.26 (0.07,148.81) | 3.56 (0.11,111.87) | 9.10 (0.10,837.04) | 9.17 (0.10,843.67) | 9.17 (0.10,844.22) | 9.21 (0.10,847.06) | 9.18 (0.26,327.13) |
| 0.22 (0.01,6.46) | 0.52 (0.06,4.34) | 0.64 (0.05,8.00) | 0.84 (0.14,5.18) | 0.63 (0.02,18.61) | Dapagliflozin | 0.98 (0.08,12.11) | 1.16 (0.19,7.09) | 1.53 (0.26,9.01) | 1.94 (0.37,10.23) | 1.93 (0.64,5.86) | 2.06 (0.19,22.06) | 2.25 (0.41,12.38) | 5.75 (0.19,170.12) | 5.80 (0.20,171.48) | 5.80 (0.20,171.59) | 5.82 (0.20,172.16) | 5.81 (0.83,40.67) |
| 0.22 (0.00,11.27) | 0.53 (0.03,9.64) | 0.66 (0.03,16.17) | 0.86 (0.06,12.59) | 0.65 (0.01,32.48) | 1.02 (0.08,12.71) | Ertugliflozin | 1.19 (0.08,17.28) | 1.57 (0.11,22.24) | 1.99 (0.15,26.21) | 1.98 (0.21,19.01) | 2.11 (0.10,46.10) | 2.31 (0.17,31.25) | 5.89 (0.12,296.66) | 5.94 (0.12,299.02) | 5.94 (0.12,299.21) | 5.97 (0.12,300.21) | 5.95 (0.37,94.97) |
| 0.19 (0.01,6.27) | 0.45 (0.04,4.50) | 0.55 (0.04,8.07) | 0.73 (0.10,5.52) | 0.54 (0.02,18.05) | 0.86 (0.14,5.26) | 0.84 (0.06,12.21) | Liraglutide | 1.32 (0.18,9.65) | 1.67 (0.25,11.10) | 1.67 (0.40,6.96) | 1.78 (0.14,22.45) | 1.94 (0.28,13.36) | 4.96 (0.15,164.98) | 4.99 (0.15,166.30) | 5.00 (0.15,166.40) | 5.01 (0.15,166.96) | 5.00 (0.58,42.78) |
| 0.14 (0.00,4.66) | 0.34 (0.05,2.52) | 0.42 (0.03,5.95) | 0.55 (0.07,4.04) | 0.41 (0.01,13.40) | 0.65 (0.11,3.83) | 0.64 (0.04,9.01) | 0.76 (0.10,5.53) | Semaglutide | 1.27 (0.20,8.10) | 1.26 (0.32,5.02) | 1.35 (0.11,16.54) | 1.47 (0.22,9.76) | 3.75 (0.11,122.50) | 3.78 (0.12,123.47) | 3.78 (0.12,123.55) | 3.80 (0.12,123.96) | 3.79 (0.46,31.36) |
| 0.11 (0.00,3.48) | 0.27 (0.03,2.40) | 0.33 (0.03,4.38) | 0.43 (0.07,2.90) | 0.33 (0.01,10.03) | 0.52 (0.10,2.72) | 0.50 (0.04,6.63) | 0.60 (0.09,3.97) | 0.79 (0.12,5.05) | Exenatide | 1.00 (0.29,3.44) | 1.06 (0.09,12.12) | 1.16 (0.19,6.96) | 2.96 (0.10,91.68) | 2.99 (0.10,92.41) | 2.99 (0.10,92.47) | 3.00 (0.10,92.78) | 2.99 (0.40,22.64) |
| 0.11 (0.00,2.77) | 0.27 (0.04,1.64) | 0.33 (0.03,3.20) | 0.44 (0.10,1.84) | 0.33 (0.01,7.99) | 0.52 (0.17,1.57) | 0.50 (0.05,4.85) | 0.60 (0.14,2.51) | 0.79 (0.20,3.16) | 1.00 (0.29,3.47) | Placebo_or_Control | 1.07 (0.13,8.66) | 1.16 (0.32,4.25) | 2.98 (0.12,73.01) | 3.00 (0.12,73.59) | 3.00 (0.12,73.64) | 3.01 (0.12,73.88) | 3.00 (0.61,14.87) |
| 0.11 (0.00,4.85) | 0.25 (0.02,4.01) | 0.31 (0.01,6.81) | 0.41 (0.03,5.18) | 0.31 (0.01,13.97) | 0.48 (0.05,5.18) | 0.47 (0.02,10.31) | 0.56 (0.04,7.10) | 0.74 (0.06,9.12) | 0.94 (0.08,10.71) | 0.94 (0.12,7.60) | Sotagliflozin | 1.09 (0.09,12.79) | 2.79 (0.06,127.62) | 2.81 (0.06,128.63) | 2.81 (0.06,128.71) | 2.82 (0.06,129.14) | 2.81 (0.20,39.22) |
| 0.10 (0.00,3.06) | 0.23 (0.02,2.14) | 0.29 (0.02,3.88) | 0.37 (0.05,2.59) | 0.28 (0.01,8.82) | 0.44 (0.08,2.44) | 0.43 (0.03,5.87) | 0.52 (0.07,3.55) | 0.68 (0.10,4.52) | 0.86 (0.14,5.17) | 0.86 (0.24,3.13) | 0.92 (0.08,10.74) | Empagliflozin | 2.55 (0.08,80.62) | 2.57 (0.08,81.27) | 2.58 (0.08,81.32) | 2.59 (0.08,81.59) | 2.58 (0.33,20.19) |
| 0.04 (0.00,3.51) | 0.09 (0.00,3.57) | 0.11 (0.00,5.64) | 0.15 (0.00,4.89) | 0.11 (0.00,10.12) | 0.17 (0.01,5.14) | 0.17 (0.00,8.54) | 0.20 (0.01,6.72) | 0.27 (0.01,8.70) | 0.34 (0.01,10.43) | 0.34 (0.01,8.25) | 0.36 (0.01,16.43) | 0.39 (0.01,12.36) | Alogliptin | 1.01 (0.01,93.09) | 1.01 (0.01,93.15) | 1.01 (0.01,93.47) | 1.01 (0.03,36.13) |
| 0.04 (0.00,3.48) | 0.09 (0.00,3.54) | 0.11 (0.00,5.59) | 0.15 (0.00,4.85) | 0.11 (0.00,10.04) | 0.17 (0.01,5.10) | 0.17 (0.00,8.47) | 0.20 (0.01,6.67) | 0.26 (0.01,8.63) | 0.33 (0.01,10.36) | 0.33 (0.01,8.18) | 0.36 (0.01,16.31) | 0.39 (0.01,12.26) | 0.99 (0.01,91.66) | Albiglutide | 1.00 (0.01,92.45) | 1.00 (0.01,92.76) | 1.00 (0.03,35.86) |
| 0.04 (0.00,3.48) | 0.09 (0.00,3.54) | 0.11 (0.00,5.59) | 0.15 (0.00,4.85) | 0.11 (0.00,10.03) | 0.17 (0.01,5.10) | 0.17 (0.00,8.47) | 0.20 (0.01,6.66) | 0.26 (0.01,8.63) | 0.33 (0.01,10.35) | 0.33 (0.01,8.18) | 0.36 (0.01,16.29) | 0.39 (0.01,12.25) | 0.99 (0.01,91.58) | 1.00 (0.01,92.31) | Lixisenatide | 1.00 (0.01,92.68) | 1.00 (0.03,35.83) |
| 0.04 (0.00,3.47) | 0.09 (0.00,3.52) | 0.11 (0.00,5.57) | 0.14 (0.00,4.83) | 0.11 (0.00,10.00) | 0.17 (0.01,5.08) | 0.17 (0.00,8.44) | 0.20 (0.01,6.64) | 0.26 (0.01,8.59) | 0.33 (0.01,10.31) | 0.33 (0.01,8.15) | 0.35 (0.01,16.23) | 0.39 (0.01,12.21) | 0.99 (0.01,91.25) | 1.00 (0.01,91.98) | 1.00 (0.01,92.04) | Omarigliptin | 1.00 (0.03,35.70) |
| 0.04 (0.00,1.35) | 0.09 (0.01,1.00) | 0.11 (0.01,1.77) | 0.15 (0.02,1.25) | 0.11 (0.00,3.88) | 0.17 (0.02,1.21) | 0.17 (0.01,2.68) | 0.20 (0.02,1.71) | 0.26 (0.03,2.19) | 0.33 (0.04,2.53) | 0.33 (0.07,1.65) | 0.36 (0.03,4.96) | 0.39 (0.05,3.04) | 0.99 (0.03,35.46) | 1.00 (0.03,35.74) | 1.00 (0.03,35.76) | 1.00 (0.03,35.88) | Dulaglutide |

Data presents RR [95%CIs]. Network meta-analysis results are presented as estimate effect sizes for the outcome of plasma cell myeloma risk. Interventions are reported in order of mean ranking of beneficially prophylactic effect on plasma cell myeloma risk, and outcomes are expressed as risk ratio (RR) (95% confidence intervals) (95%CIs). For the upper-right portion, RR of less than 1 indicates that the treatment specified in the row got more beneficial effect than that specified in the column. For the lower-left portion, RR of less than 1 indicates that the treatment specified in the column has more beneficial effect than that specified in the row. Bold results marked with * indicate statistical significance.

**eTable 6L: League table of NMA of the acceptability: drop-out rate**

| Tirzepatide | 1.12 (0.85,1.47) | 1.22 (0.89,1.66) | 1.23 (0.94,1.61) | 1.26 (0.92,1.72) | 1.25 (0.76,2.04) | ***1.37 (1.05,1.78)** | ***1.36 (1.06,1.75)** | 1.38 (0.93,2.05) | ***1.39 (1.09,1.76)** | ***1.40 (1.03,1.90)** | 1.40 (0.77,2.54) | ***1.43 (1.01,2.02)** | ***1.44 (1.07,1.94)** | 1.46 (0.97,2.20) | ***1.46 (1.09,1.96)** | ***1.50 (1.11,2.04)** | ***1.51 (1.14,2.00)** | ***1.56 (1.07,2.27)** | ***1.56 (1.09,2.22)** | ***1.61 (1.11,2.34)** | ***1.59 (1.30,1.94)** |
| --- | --- | --- | --- | --- | --- | --- | --- | --- | --- | --- | --- | --- | --- | --- | --- | --- | --- | --- | --- | --- | --- |
| 0.90 (0.68,1.18) | Liraglutide | 1.09 (0.80,1.49) | 1.10 (0.83,1.47) | 1.13 (0.83,1.54) | 1.12 (0.69,1.82) | 1.23 (0.95,1.58) | 1.22 (0.96,1.55) | 1.24 (0.84,1.83) | 1.24 (0.99,1.57) | 1.25 (0.93,1.68) | 1.25 (0.69,2.27) | 1.28 (0.94,1.74) | 1.29 (0.96,1.72) | 1.31 (0.89,1.93) | 1.31 (0.98,1.75) | 1.35 (0.99,1.82) | ***1.35 (1.02,1.78)** | 1.40 (0.96,2.02) | 1.39 (0.98,1.98) | 1.44 (1.00,2.08) | ***1.42 (1.18,1.72)** |
| 0.82 (0.60,1.13) | 0.92 (0.67,1.25) | Canagliflozin | 1.01 (0.73,1.40) | 1.04 (0.74,1.47) | 1.02 (0.61,1.71) | 1.13 (0.83,1.52) | 1.12 (0.84,1.50) | 1.14 (0.75,1.73) | 1.14 (0.86,1.51) | 1.15 (0.82,1.61) | 1.15 (0.62,2.12) | 1.17 (0.81,1.71) | 1.18 (0.85,1.65) | 1.20 (0.78,1.85) | 1.20 (0.87,1.67) | 1.24 (0.88,1.74) | 1.24 (0.90,1.70) | 1.28 (0.86,1.92) | 1.28 (0.87,1.88) | 1.32 (0.88,1.97) | ***1.31 (1.02,1.67)** |
| 0.81 (0.62,1.06) | 0.91 (0.68,1.21) | 0.99 (0.71,1.37) | Semaglutide | 1.03 (0.74,1.42) | 1.01 (0.62,1.66) | 1.11 (0.85,1.46) | 1.11 (0.86,1.42) | 1.12 (0.75,1.68) | 1.13 (0.88,1.45) | 1.14 (0.83,1.55) | 1.14 (0.62,2.07) | 1.16 (0.81,1.66) | 1.17 (0.86,1.59) | 1.19 (0.78,1.80) | 1.19 (0.88,1.61) | 1.22 (0.89,1.68) | 1.23 (0.91,1.64) | 1.27 (0.86,1.86) | 1.26 (0.88,1.82) | 1.31 (0.89,1.91) | ***1.29 (1.04,1.60)** |
| 0.79 (0.58,1.08) | 0.88 (0.65,1.20) | 0.96 (0.68,1.36) | 0.97 (0.71,1.35) | Dapagliflozin | 0.99 (0.59,1.65) | 1.08 (0.81,1.46) | 1.08 (0.81,1.44) | 1.09 (0.72,1.66) | 1.10 (0.83,1.45) | 1.11 (0.79,1.55) | 1.11 (0.60,2.04) | 1.13 (0.78,1.64) | 1.14 (0.82,1.58) | 1.16 (0.75,1.78) | 1.16 (0.84,1.60) | 1.19 (0.85,1.67) | 1.19 (0.87,1.64) | 1.24 (0.83,1.84) | 1.23 (0.84,1.81) | 1.27 (0.85,1.90) | 1.26 (0.99,1.60) |
| 0.80 (0.49,1.31) | 0.90 (0.55,1.46) | 0.98 (0.58,1.63) | 0.99 (0.60,1.62) | 1.01 (0.61,1.69) | Ertugliflozin_  Sitagliptin | 1.10 (0.68,1.78) | 1.09 (0.70,1.71) | 1.11 (0.63,1.95) | 1.12 (0.70,1.78) | 1.12 (0.73,1.73) | 1.12 (0.55,2.30) | 1.15 (0.67,1.95) | 1.16 (0.70,1.91) | 1.17 (0.66,2.08) | 1.17 (0.71,1.93) | 1.21 (0.73,2.00) | 1.21 (0.74,1.98) | 1.25 (0.72,2.17) | 1.25 (0.73,2.14) | 1.29 (0.74,2.23) | 1.28 (0.81,2.00) |
| ***0.73 (0.56,0.95)** | 0.82 (0.63,1.05) | 0.89 (0.66,1.20) | 0.90 (0.68,1.18) | 0.92 (0.69,1.24) | 0.91 (0.56,1.47) | Saxagliptin | 0.99 (0.79,1.25) | 1.01 (0.69,1.48) | 1.02 (0.82,1.26) | 1.02 (0.77,1.36) | 1.02 (0.57,1.84) | 1.04 (0.75,1.45) | 1.05 (0.80,1.39) | 1.07 (0.72,1.58) | 1.07 (0.81,1.41) | 1.10 (0.82,1.47) | 1.10 (0.85,1.43) | 1.14 (0.79,1.64) | 1.14 (0.81,1.60) | 1.17 (0.82,1.68) | 1.16 (0.98,1.38) |
| ***0.73 (0.57,0.95)** | 0.82 (0.64,1.04) | 0.89 (0.67,1.20) | 0.90 (0.70,1.16) | 0.93 (0.70,1.24) | 0.92 (0.58,1.43) | 1.01 (0.80,1.26) | Sitagliptin | 1.02 (0.70,1.47) | 1.02 (0.84,1.25) | 1.03 (0.80,1.33) | 1.03 (0.57,1.84) | 1.05 (0.76,1.45) | 1.06 (0.81,1.39) | 1.07 (0.73,1.58) | 1.08 (0.83,1.40) | 1.10 (0.83,1.47) | 1.11 (0.86,1.43) | 1.15 (0.80,1.63) | 1.14 (0.82,1.60) | 1.18 (0.83,1.68) | 1.17 (1.00,1.36) |
| 0.72 (0.49,1.07) | 0.81 (0.55,1.19) | 0.88 (0.58,1.34) | 0.89 (0.60,1.33) | 0.91 (0.60,1.39) | 0.90 (0.51,1.58) | 0.99 (0.68,1.45) | 0.98 (0.68,1.43) | Vildagliptin | 1.01 (0.70,1.45) | 1.01 (0.67,1.52) | 1.01 (0.53,1.95) | 1.03 (0.66,1.61) | 1.04 (0.69,1.56) | 1.06 (0.64,1.73) | 1.06 (0.71,1.58) | 1.09 (0.72,1.64) | 1.09 (0.73,1.62) | 1.13 (0.71,1.80) | 1.13 (0.72,1.76) | 1.16 (0.73,1.85) | 1.15 (0.82,1.61) |
| ***0.72 (0.57,0.91)** | 0.80 (0.64,1.01) | 0.87 (0.66,1.16) | 0.89 (0.69,1.14) | 0.91 (0.69,1.20) | 0.90 (0.56,1.43) | 0.98 (0.79,1.22) | 0.98 (0.80,1.20) | 0.99 (0.69,1.43) | Empagliflozin | 1.01 (0.77,1.31) | 1.01 (0.57,1.79) | 1.03 (0.75,1.41) | 1.04 (0.80,1.34) | 1.05 (0.72,1.54) | 1.05 (0.82,1.36) | 1.08 (0.82,1.42) | 1.08 (0.85,1.38) | 1.12 (0.79,1.59) | 1.12 (0.81,1.55) | 1.16 (0.82,1.63) | 1.14 (1.00,1.30) |
| ***0.71 (0.53,0.97)** | 0.80 (0.59,1.07) | 0.87 (0.62,1.22) | 0.88 (0.64,1.20) | 0.90 (0.65,1.26) | 0.89 (0.58,1.37) | 0.98 (0.74,1.30) | 0.97 (0.75,1.26) | 0.99 (0.66,1.49) | 0.99 (0.76,1.29) | Ertugliflozin | 1.00 (0.55,1.83) | 1.02 (0.71,1.47) | 1.03 (0.75,1.41) | 1.04 (0.68,1.59) | 1.05 (0.76,1.43) | 1.07 (0.77,1.49) | 1.08 (0.79,1.46) | 1.11 (0.75,1.65) | 1.11 (0.76,1.61) | 1.15 (0.78,1.70) | 1.13 (0.90,1.43) |
| 0.71 (0.39,1.30) | 0.80 (0.44,1.44) | 0.87 (0.47,1.60) | 0.88 (0.48,1.60) | 0.90 (0.49,1.66) | 0.89 (0.43,1.83) | 0.98 (0.54,1.76) | 0.97 (0.54,1.74) | 0.99 (0.51,1.90) | 0.99 (0.56,1.77) | 1.00 (0.55,1.83) | Bexagliflozin | 1.02 (0.54,1.91) | 1.03 (0.56,1.88) | 1.04 (0.54,2.03) | 1.05 (0.57,1.91) | 1.07 (0.58,1.97) | 1.08 (0.59,1.96) | 1.11 (0.58,2.13) | 1.11 (0.59,2.10) | 1.15 (0.60,2.19) | 1.14 (0.65,1.99) |
| ***0.70 (0.50,0.99)** | 0.78 (0.57,1.06) | 0.85 (0.58,1.24) | 0.86 (0.60,1.23) | 0.88 (0.61,1.28) | 0.87 (0.51,1.48) | 0.96 (0.69,1.33) | 0.95 (0.69,1.32) | 0.97 (0.62,1.51) | 0.97 (0.71,1.33) | 0.98 (0.68,1.41) | 0.98 (0.52,1.84) | Exenatide | 1.01 (0.70,1.44) | 1.02 (0.65,1.61) | 1.02 (0.72,1.46) | 1.05 (0.73,1.52) | 1.06 (0.75,1.49) | 1.09 (0.71,1.67) | 1.09 (0.72,1.64) | 1.12 (0.74,1.72) | 1.11 (0.84,1.48) |
| ***0.69 (0.52,0.94)** | 0.78 (0.58,1.04) | 0.84 (0.61,1.18) | 0.85 (0.63,1.16) | 0.88 (0.63,1.22) | 0.87 (0.52,1.43) | 0.95 (0.72,1.26) | 0.95 (0.72,1.24) | 0.96 (0.64,1.44) | 0.97 (0.75,1.25) | 0.97 (0.71,1.34) | 0.97 (0.53,1.78) | 0.99 (0.69,1.42) | Alogliptin | 1.01 (0.67,1.54) | 1.02 (0.75,1.39) | 1.04 (0.76,1.44) | 1.05 (0.78,1.41) | 1.08 (0.73,1.60) | 1.08 (0.75,1.56) | 1.12 (0.76,1.64) | 1.10 (0.88,1.38) |
| 0.69 (0.46,1.03) | 0.77 (0.52,1.13) | 0.83 (0.54,1.29) | 0.84 (0.56,1.28) | 0.87 (0.56,1.33) | 0.85 (0.48,1.52) | 0.94 (0.63,1.39) | 0.93 (0.63,1.37) | 0.95 (0.58,1.55) | 0.95 (0.65,1.39) | 0.96 (0.63,1.47) | 0.96 (0.49,1.87) | 0.98 (0.62,1.54) | 0.99 (0.65,1.50) | Lixisenatide | 1.00 (0.66,1.52) | 1.03 (0.67,1.58) | 1.03 (0.69,1.56) | 1.07 (0.66,1.73) | 1.07 (0.67,1.70) | 1.10 (0.68,1.78) | 1.09 (0.76,1.56) |
| ***0.68 (0.51,0.92)** | 0.76 (0.57,1.02) | 0.83 (0.60,1.15) | 0.84 (0.62,1.14) | 0.86 (0.62,1.19) | 0.85 (0.52,1.40) | 0.93 (0.71,1.23) | 0.93 (0.71,1.21) | 0.94 (0.63,1.41) | 0.95 (0.74,1.22) | 0.96 (0.70,1.31) | 0.96 (0.52,1.74) | 0.98 (0.68,1.39) | 0.98 (0.72,1.34) | 1.00 (0.66,1.51) | Sotagliflozin | 1.03 (0.75,1.41) | 1.03 (0.77,1.38) | 1.07 (0.72,1.57) | 1.06 (0.74,1.53) | 1.10 (0.75,1.61) | 1.09 (0.88,1.35) |
| ***0.67 (0.49,0.90)** | 0.74 (0.55,1.01) | 0.81 (0.58,1.14) | 0.82 (0.60,1.13) | 0.84 (0.60,1.18) | 0.83 (0.50,1.38) | 0.91 (0.68,1.22) | 0.91 (0.68,1.20) | 0.92 (0.61,1.39) | 0.92 (0.71,1.21) | 0.93 (0.67,1.29) | 0.93 (0.51,1.71) | 0.95 (0.66,1.37) | 0.96 (0.69,1.32) | 0.97 (0.63,1.49) | 0.97 (0.71,1.34) | Linagliptin | 1.00 (0.74,1.37) | 1.04 (0.70,1.54) | 1.04 (0.71,1.51) | 1.07 (0.72,1.59) | 1.06 (0.83,1.34) |
| ***0.66 (0.50,0.88)** | ***0.74 (0.56,0.98)** | 0.81 (0.59,1.11) | 0.82 (0.61,1.10) | 0.84 (0.61,1.15) | 0.83 (0.51,1.35) | 0.91 (0.70,1.18) | 0.90 (0.70,1.16) | 0.92 (0.62,1.36) | 0.92 (0.72,1.17) | 0.93 (0.69,1.26) | 0.93 (0.51,1.69) | 0.95 (0.67,1.34) | 0.96 (0.71,1.29) | 0.97 (0.64,1.46) | 0.97 (0.72,1.30) | 1.00 (0.73,1.36) | Albiglutide | 1.03 (0.71,1.51) | 1.03 (0.72,1.48) | 1.07 (0.73,1.55) | 1.05 (0.86,1.29) |
| ***0.64 (0.44,0.93)** | 0.72 (0.49,1.04) | 0.78 (0.52,1.17) | 0.79 (0.54,1.16) | 0.81 (0.54,1.21) | 0.80 (0.46,1.39) | 0.88 (0.61,1.26) | 0.87 (0.61,1.24) | 0.89 (0.56,1.41) | 0.89 (0.63,1.26) | 0.90 (0.61,1.33) | 0.90 (0.47,1.71) | 0.92 (0.60,1.40) | 0.92 (0.63,1.36) | 0.94 (0.58,1.51) | 0.94 (0.64,1.38) | 0.96 (0.65,1.43) | 0.97 (0.66,1.41) | Efpeglenatide | 1.00 (0.65,1.54) | 1.03 (0.66,1.62) | 1.02 (0.74,1.40) |
| ***0.64 (0.45,0.92)** | 0.72 (0.51,1.02) | 0.78 (0.53,1.15) | 0.79 (0.55,1.14) | 0.81 (0.55,1.19) | 0.80 (0.47,1.37) | 0.88 (0.63,1.24) | 0.87 (0.63,1.22) | 0.89 (0.57,1.39) | 0.89 (0.65,1.24) | 0.90 (0.62,1.31) | 0.90 (0.48,1.70) | 0.92 (0.61,1.38) | 0.93 (0.64,1.34) | 0.94 (0.59,1.49) | 0.94 (0.65,1.36) | 0.97 (0.66,1.41) | 0.97 (0.68,1.39) | 1.00 (0.65,1.55) | Dulaglutide | 1.03 (0.67,1.59) | 1.02 (0.76,1.37) |
| ***0.62 (0.43,0.90)** | 0.69 (0.48,1.00) | 0.76 (0.51,1.13) | 0.77 (0.52,1.12) | 0.79 (0.53,1.17) | 0.78 (0.45,1.34) | 0.85 (0.59,1.22) | 0.85 (0.60,1.20) | 0.86 (0.54,1.37) | 0.86 (0.61,1.22) | 0.87 (0.59,1.29) | 0.87 (0.46,1.66) | 0.89 (0.58,1.36) | 0.90 (0.61,1.32) | 0.91 (0.56,1.46) | 0.91 (0.62,1.34) | 0.94 (0.63,1.39) | 0.94 (0.64,1.37) | 0.97 (0.62,1.52) | 0.97 (0.63,1.49) | Omarigliptin | 0.99 (0.72,1.36) |
| ***0.63 (0.52,0.77)** | ***0.70 (0.58,0.85)** | ***0.77 (0.60,0.98)** | ***0.77 (0.63,0.96)** | 0.79 (0.62,1.01) | 0.78 (0.50,1.23) | 0.86 (0.73,1.02) | 0.86 (0.73,1.00) | 0.87 (0.62,1.22) | 0.87 (0.77,1.00) | 0.88 (0.70,1.11) | 0.88 (0.50,1.54) | 0.90 (0.68,1.19) | 0.91 (0.73,1.13) | 0.92 (0.64,1.31) | 0.92 (0.74,1.14) | 0.95 (0.75,1.20) | 0.95 (0.78,1.16) | 0.98 (0.71,1.35) | 0.98 (0.73,1.32) | 1.01 (0.74,1.39) | Placebo_or_  Control |

Data presents RR [95%CIs]. Network meta-analysis results are presented as estimate effect sizes for the outcome of drop-out rate. Interventions are reported in order of mean ranking of acceptability, and outcomes are expressed as risk ratio (RR) (95% confidence intervals) (95%CIs). For the upper-right portion, RR of less than 1 indicates that the treatment specified in the row got better acceptability than that specified in the column. For the lower-left portion, RR of less than 1 indicates that the treatment specified in the column has better acceptability than that specified in the row. Bold results marked with * indicate statistical significance.

*Abbreviation: 95%CIs: 95% confidence intervals; DPP4 inhibitor: dipeptidyl-peptidase 4 inhibitor; GLP-1 agonist: glucagon-like peptide-1 agonist; NMA: network meta-analysis; RR: risk ratio; RCT: randomized controlled trial; SGLT2 inhibitor: sodium–glucose cotransporter 2 inhibitor*

**eTable 7A SUCRA for primary outcome: overall hematologic malignancy risk**

| Treatment | SUCRA | PrBest | MeanRank |
| --- | --- | --- | --- |
| Tirzepatide | 94.7 | 49.2 | 2.1 |
| Linagliptin | 83.6 | 2.3 | 4.4 |
| Canagliflozin | 76.5 | 2.7 | 5.9 |
| Lixisenatide | 73.8 | 6.2 | 6.5 |
| Vildagliptin | 71.5 | 23.3 | 7.0 |
| Semaglutide | 68.8 | 0.1 | 7.5 |
| Liraglutide | 63.0 | 0.2 | 8.8 |
| Sitagliptin | 55.0 | 0.0 | 10.5 |
| Placebo_or_Control | 54.6 | 0.0 | 10.5 |
| Exenatide | 49.7 | 0.1 | 11.6 |
| Saxagliptin | 46.9 | 0.0 | 12.2 |
| Dapagliflozin | 45.9 | 0.0 | 12.4 |
| Sotagliflozin | 44.9 | 0.2 | 12.6 |
| Bexagliflozin | 43.2 | 8.8 | 12.9 |
| Ertugliflozin | 43.1 | 0.0 | 12.9 |
| Albiglutide | 33.2 | 0.2 | 15.0 |
| Ertugliflozin_Sitagliptin | 31.2 | 2.0 | 15.4 |
| Empagliflozin | 30.3 | 0.0 | 15.6 |
| Efpeglenatide | 27.1 | 1.7 | 16.3 |
| Alogliptin | 24.7 | 0.9 | 16.8 |
| Dulaglutide | 19.6 | 0.0 | 17.9 |
| Omarigliptin | 18.7 | 2.1 | 18.1 |

**eTable 7B SUCRA for primary outcome: leukemia risk**

| Treatment | SUCRA | PrBest | MeanRank |
| --- | --- | --- | --- |
| Tirzepatide | 74.6 | 22.9 | 5.3 |
| Canagliflozin | 74.4 | 11.9 | 5.3 |
| Linagliptin | 74.3 | 6.8 | 5.4 |
| Lixisenatide | 66.9 | 19.2 | 6.6 |
| Liraglutide | 66.5 | 3.6 | 6.7 |
| Semaglutide | 60.7 | 2.2 | 7.7 |
| Saxagliptin | 58.5 | 2.1 | 8.1 |
| Exenatide | 56.1 | 1.1 | 8.5 |
| Vildagliptin | 48.6 | 14.6 | 9.7 |
| Dapagliflozin | 44.2 | 0.0 | 10.5 |
| Placebo_or_Control | 44.1 | 0.0 | 10.5 |
| Sotagliflozin | 42.1 | 2.3 | 10.8 |
| Efpeglenatide | 38.5 | 10.4 | 11.5 |
| Albiglutide | 38.0 | 1.0 | 11.5 |
| Sitagliptin | 34.5 | 0.2 | 12.1 |
| Dulaglutide | 28.5 | 0.4 | 13.2 |
| Empagliflozin | 27.3 | 0.2 | 13.4 |
| Ertugliflozin | 22.3 | 1.1 | 14.2 |

**eTable 7C SUCRA for primary outcome: acute lymphocytic leukemia risk**

| Treatment | SUCRA | PrBest | MeanRank |
| --- | --- | --- | --- |
| Ertugliflozin | 77.5 | 40.6 | 2.1 |
| Linagliptin | 67.9 | 28.6 | 2.6 |
| Semaglutide | 67.3 | 25.8 | 2.6 |
| Placebo_or_Control | 43.5 | 0.9 | 3.8 |
| Sotagliflozin | 22.9 | 3.1 | 4.9 |
| Empagliflozin | 20.8 | 1.0 | 5.0 |

**eTable 7D SUCRA for primary outcome: acute myeloid leukemia risk**

| Treatment | SUCRA | PrBest | MeanRank |
| --- | --- | --- | --- |
| Linagliptin | 75.6 | 16.6 | 4.4 |
| Exenatide | 71.8 | 11.9 | 5.0 |
| Canagliflozin | 65.5 | 8.7 | 5.8 |
| Dulaglutide | 64.1 | 17.0 | 6.0 |
| Tirzepatide | 63.2 | 15.6 | 6.2 |
| Lixisenatide | 62.5 | 19.7 | 6.3 |
| Semaglutide | 60.8 | 4.7 | 6.5 |
| Saxagliptin | 57.4 | 3.9 | 7.0 |
| Dapagliflozin | 44.8 | 0.1 | 8.7 |
| Liraglutide | 39.5 | 0.4 | 9.5 |
| Placebo_or_Control | 37.1 | 0.0 | 9.8 |
| Empagliflozin | 29.0 | 0.2 | 10.9 |
| Sitagliptin | 28.3 | 0.3 | 11.0 |
| Albiglutide | 26.2 | 0.1 | 11.3 |
| Ertugliflozin | 24.3 | 0.8 | 11.6 |

**eTable 7E SUCRA for primary outcome: chronic lymphocytic leukemia risk**

| Treatment | SUCRA | PrBest | MeanRank |
| --- | --- | --- | --- |
| Liraglutide | 80.6 | 18.2 | 4.1 |
| Sotagliflozin | 72.7 | 22.1 | 5.4 |
| Albiglutide | 71.1 | 18.0 | 5.6 |
| Canagliflozin | 70.7 | 21.3 | 5.7 |
| Linagliptin | 65.6 | 4.1 | 6.5 |
| Dapagliflozin | 51.5 | 0.0 | 8.8 |
| Exenatide | 51.0 | 0.8 | 8.8 |
| Placebo_or_Control | 50.4 | 0.0 | 8.9 |
| Sitagliptin | 48.2 | 0.4 | 9.3 |
| Lixisenatide | 48.0 | 4.7 | 9.3 |
| Empagliflozin | 47.2 | 0.1 | 9.4 |
| Ertugliflozin | 43.6 | 3.8 | 10.0 |
| Efpeglenatide | 43.4 | 3.9 | 10.1 |
| Semaglutide | 38.2 | 0.8 | 10.9 |
| Vildagliptin | 28.6 | 1.8 | 12.4 |
| Dulaglutide | 20.3 | 0.0 | 13.7 |
| Saxagliptin | 19.1 | 0.0 | 13.9 |

**eTable 7F SUCRA for primary outcome: chronic myeloid leukemia risk**

| Treatment | SUCRA | PrBest | MeanRank |
| --- | --- | --- | --- |
| Vildagliptin | 70.1 | 22.6 | 5.2 |
| Semaglutide | 69.7 | 24.5 | 5.2 |
| Canagliflozin | 69.0 | 10.4 | 5.3 |
| Sitagliptin | 62.0 | 6.9 | 6.3 |
| Liraglutide | 51.1 | 2.5 | 7.8 |
| Saxagliptin | 49.5 | 5.6 | 8.1 |
| Linagliptin | 48.9 | 5.9 | 8.2 |
| Placebo_or_Control | 48.5 | 0.0 | 8.2 |
| Exenatide | 48.5 | 6.1 | 8.2 |
| Empagliflozin | 48.3 | 3.2 | 8.2 |
| Tirzepatide | 48.2 | 6.9 | 8.2 |
| Dapagliflozin | 48.0 | 0.9 | 8.3 |
| Sotagliflozin | 31.0 | 0.4 | 10.7 |
| Dulaglutide | 29.1 | 2.5 | 10.9 |
| Albiglutide | 28.0 | 1.6 | 11.1 |

**eTable 7G SUCRA for primary outcome: lymphoma risk**

| Treatment | SUCRA | PrBest | MeanRank |
| --- | --- | --- | --- |
| Tirzepatide | 92.4 | 36.2 | 2.6 |
| Lixisenatide | 80.7 | 12.1 | 5.0 |
| Vildagliptin | 75.2 | 31.8 | 6.2 |
| Canagliflozin | 69.8 | 2.5 | 7.3 |
| Semaglutide | 69.5 | 0.3 | 7.4 |
| Linagliptin | 68.1 | 0.5 | 7.7 |
| Placebo_or_Control | 58.7 | 0.0 | 9.7 |
| Sotagliflozin | 52.8 | 1.3 | 10.9 |
| Sitagliptin | 48.0 | 0.0 | 11.9 |
| Ertugliflozin | 47.8 | 0.1 | 12.0 |
| Liraglutide | 47.1 | 0.1 | 12.1 |
| Empagliflozin | 46.5 | 0.0 | 12.2 |
| Bexagliflozin | 43.8 | 6.5 | 12.8 |
| Exenatide | 43.5 | 0.0 | 12.9 |
| Albiglutide | 36.0 | 0.8 | 14.4 |
| Alogliptin | 35.4 | 1.4 | 14.6 |
| Saxagliptin | 35.3 | 0.0 | 14.6 |
| Efpeglenatide | 35.0 | 2.3 | 14.7 |
| Omarigliptin | 30.8 | 2.1 | 15.5 |
| Dapagliflozin | 29.7 | 0.0 | 15.8 |
| Ertugliflozin_Sitagliptin | 29.3 | 2.0 | 15.9 |
| Dulaglutide | 24.5 | 0.0 | 16.9 |

**eTable 7H SUCRA for primary outcome: non-Hodgkin's lymphoma risk**

| Treatment | SUCRA | PrBest | MeanRank |
| --- | --- | --- | --- |
| Tirzepatide | 92.6 | 39.7 | 2.5 |
| Lixisenatide | 74.4 | 10.2 | 6.4 |
| Vildagliptin | 74.1 | 27.2 | 6.4 |
| Semaglutide | 68.9 | 0.7 | 7.5 |
| Canagliflozin | 66.2 | 3.0 | 8.1 |
| Linagliptin | 60.0 | 0.8 | 9.4 |
| Placebo_or_Control | 56.3 | 0.0 | 10.2 |
| Ertugliflozin | 55.1 | 0.6 | 10.4 |
| Sitagliptin | 51.8 | 0.0 | 11.1 |
| Sotagliflozin | 51.3 | 0.7 | 11.2 |
| Empagliflozin | 50.4 | 0.0 | 11.4 |
| Liraglutide | 49.2 | 0.1 | 11.7 |
| Bexagliflozin | 43.2 | 6.5 | 12.9 |
| Saxagliptin | 41.4 | 0.0 | 13.3 |
| Exenatide | 40.3 | 0.2 | 13.5 |
| Dapagliflozin | 37.3 | 0.0 | 14.2 |
| Albiglutide | 35.0 | 0.8 | 14.6 |
| Ertugliflozin_Sitagliptin | 34.3 | 2.9 | 14.8 |
| Efpeglenatide | 34.2 | 2.7 | 14.8 |
| Alogliptin | 32.5 | 1.4 | 15.2 |
| Omarigliptin | 30.2 | 2.5 | 15.7 |
| Dulaglutide | 21.3 | 0.0 | 17.5 |

**eTable 7I SUCRA for primary outcome: B cell non-Hodgkin's lymphoma risk**

| Treatment | SUCRA | PrBest | MeanRank |
| --- | --- | --- | --- |
| Tirzepatide | 81.8 | 37.5 | 4.4 |
| Lixisenatide | 77.5 | 16.9 | 5.3 |
| Semaglutide | 73.3 | 2.2 | 6.1 |
| Vildagliptin | 70.8 | 22.5 | 6.5 |
| Sotagliflozin | 67.1 | 3.8 | 7.3 |
| Canagliflozin | 61.2 | 2.4 | 8.4 |
| Sitagliptin | 56.5 | 0.1 | 9.3 |
| Ertugliflozin | 54.1 | 0.6 | 9.7 |
| Placebo_or_Control | 52.5 | 0.0 | 10.0 |
| Liraglutide | 51.0 | 0.2 | 10.3 |
| Empagliflozin | 44.5 | 0.3 | 11.5 |
| Bexagliflozin | 42.8 | 5.8 | 11.9 |
| Linagliptin | 42.4 | 0.4 | 11.9 |
| Efpeglenatide | 41.2 | 4.8 | 12.2 |
| Ertugliflozin_Sitagliptin | 35.2 | 1.7 | 13.3 |
| Dapagliflozin | 34.3 | 0.0 | 13.5 |
| Albiglutide | 33.2 | 0.6 | 13.7 |
| Exenatide | 31.9 | 0.0 | 13.9 |
| Saxagliptin | 30.7 | 0.2 | 14.2 |
| Dulaglutide | 17.9 | 0.0 | 16.6 |

**eTable 7J SUCRA for primary outcome: T cell non-Hodgkin's lymphoma risk**

| Treatment | SUCRA | PrBest | MeanRank |
| --- | --- | --- | --- |
| Sitagliptin | 86.7 | 66.6 | 1.7 |
| Dapagliflozin | 48.7 | 7.6 | 3.6 |
| Placebo_or_Control | 48.3 | 1.0 | 3.6 |
| Empagliflozin | 47.3 | 8.4 | 3.6 |
| Efpeglenatide | 39.4 | 9.5 | 4.0 |
| Exenatide | 29.5 | 6.9 | 4.5 |

**eTable 7K SUCRA for primary outcome: myeloma risk**

| Treatment | SUCRA | PrBest | MeanRank |
| --- | --- | --- | --- |
| Tirzepatide | 85.7 | 50.5 | 3.4 |
| Sitagliptin | 79.4 | 14.3 | 4.5 |
| Canagliflozin | 72.0 | 11.1 | 5.8 |
| Linagliptin | 70.8 | 4.0 | 6.0 |
| Liraglutide | 64.9 | 1.4 | 7.0 |
| Ertugliflozin | 64.1 | 7.3 | 7.1 |
| Dapagliflozin | 60.6 | 1.6 | 7.7 |
| Semaglutide | 51.9 | 0.4 | 9.2 |
| Saxagliptin | 50.8 | 0.3 | 9.4 |
| Placebo_or_Control | 44.0 | 0.0 | 10.5 |
| Sotagliflozin | 43.8 | 1.5 | 10.6 |
| Exenatide | 42.0 | 0.0 | 10.9 |
| Empagliflozin | 41.2 | 0.2 | 11.0 |
| Omarigliptin | 28.5 | 2.0 | 13.2 |
| Albiglutide | 27.4 | 2.1 | 13.3 |
| Lixisenatide | 26.2 | 1.7 | 13.5 |
| Alogliptin | 25.9 | 1.6 | 13.6 |
| Dulaglutide | 20.9 | 0.0 | 14.4 |

**eTable 7L SUCRA for primary outcome: plasma cell myeloma risk**

| Treatment | SUCRA | PrBest | MeanRank |
| --- | --- | --- | --- |
| Tirzepatide | 84.3 | 41.5 | 3.7 |
| Sitagliptin | 77.4 | 9.6 | 4.8 |
| Canagliflozin | 70.5 | 9.4 | 6.0 |
| Linagliptin | 68.7 | 2.4 | 6.3 |
| Saxagliptin | 68.5 | 22.0 | 6.4 |
| Dapagliflozin | 64.0 | 1.6 | 7.1 |
| Ertugliflozin | 62.4 | 5.9 | 7.4 |
| Liraglutide | 59.8 | 0.8 | 7.8 |
| Semaglutide | 50.7 | 0.4 | 9.4 |
| Exenatide | 43.1 | 0.0 | 10.7 |
| Placebo_or_Control | 42.9 | 0.0 | 10.7 |
| Sotagliflozin | 42.8 | 0.3 | 10.7 |
| Empagliflozin | 40.5 | 0.1 | 11.1 |
| Alogliptin | 28.3 | 1.8 | 13.2 |
| Albiglutide | 27.0 | 1.6 | 13.4 |
| Lixisenatide | 25.9 | 1.6 | 13.6 |
| Omarigliptin | 23.8 | 1.0 | 14.0 |
| Dulaglutide | 19.4 | 0.0 | 14.7 |

*Abbreviation: 95%CIs: 95% confidence intervals; DPP4 inhibitor: dipeptidyl-peptidase 4 inhibitor; GLP-1 agonist: glucagon-like peptide-1 agonist; NMA: network meta-analysis; RR: risk ratio; RCT: randomized controlled trial; SGLT2 inhibitor: sodium–glucose cotransporter 2 inhibitor*

**eTable 8A Heterogeneity for primary outcome: overall hematologic malignancy risk**

|  | Heterogeneity statistic | degrees of freedom | *p* | *I squared* | *Tau-squared* | Treatments used |  |
| --- | --- | --- | --- | --- | --- | --- | --- |
| C - A | 1.11 | 3 | 0.775 | 0.00% | 0 | A: | Placebo_or_Control |
| D - A | 3.76 | 3 | 0.289 | 20.20% | 0.2608 | B: | Liraglutide |
| F - A | 0 | 0 | . | .% | 0 | C: | Albiglutide |
| H - A | 2.38 | 6 | 0.881 | 0.00% | 0 | D: | Canagliflozin |
| I - A | 0.01 | 2 | 0.993 | 0.00% | 0 | E: | Ertugliflozin_Sitagliptin |
| K - A | 9.64 | 6 | 0.141 | 37.80% | 0.2524 | F: | Efpeglenatide |
| L - A | 0.13 | 2 | 0.939 | 0.00% | 0 | G: | Omarigliptin |
| B - A | 0.71 | 2 | 0.701 | 0.00% | 0 | H: | Empagliflozin |
| O - A | 0 | 0 | . | .% | 0 | I: | Ertugliflozin |
| R - A | 0 | 0 | . | .% | 0 | J: | Vildagliptin |
| P - A | 3.68 | 5 | 0.596 | 0.00% | 0 | K: | Dapagliflozin |
| N - A | 2.66 | 3 | 0.447 | 0.00% | 0 | L: | Dulaglutide |
| U - P | 0 | 0 | . | .% | 0 | M: | Linagliptin |
| O - B | 0 | 0 | . | .% | 0 | N: | Sotagliflozin |
| R - B | 0 | 0 | . | .% | 0 | O: | Exenatide |
| S - A | 0.48 | 4 | 0.976 | 0.00% | 0 | P: | Semaglutide |
| S - P | 0 | 0 | . | .% | 0 | Q: | Alogliptin |
| V - A | 0 | 0 | . | .% | 0 | R: | Lixisenatide |
| U - A | 2.06 | 6 | 0.914 | 0.00% | 0 | S: | Tirzepatide |
| U - H | 0 | 0 | . | .% | 0 | T: | Saxagliptin |
| Q - A | 0.29 | 1 | 0.593 | 0.00% | 0 | U: | Sitagliptin |
| M - A | 0.85 | 1 | 0.358 | 0.00% | 0 | V: | Bexagliflozin |
| G - A | 0 | 0 | . | .% | 0 |  |  |
| T - A | 1.35 | 4 | 0.854 | 0.00% | 0 |  |  |
| I - E | 0 | 0 | . | .% | 0 |  |  |
| U - E | 0 | 0 | . | .% | 0 |  |  |
| U - I | 0 | 0 | . | .% | 0 |  |  |
| J - A | 0 | 0 | . | .% | 0 |  |  |

**eTable 8B Heterogeneity for primary outcome: leukemia risk**

|  | Heterogeneity statistic | degrees of freedom | *p* | *I squared* | *Tau-squared* | Treatments used |  |
| --- | --- | --- | --- | --- | --- | --- | --- |
| C - A | 0.89 | 2 | 0.642 | 0.00% | 0 | A: | Placebo_or_Control |
| D - A | 0.64 | 3 | 0.888 | 0.00% | 0 | B: | Liraglutide |
| F - A | 0 | 0 | . | .% | 0 | C: | Albiglutide |
| H - A | 2.99 | 6 | 0.81 | 0.00% | 0 | D: | Canagliflozin |
| I - A | 0 | 0 | . | .% | 0 | E: | Saxagliptin |
| K - A | 7.7 | 5 | 0.173 | 35.10% | 0.3901 | F: | Efpeglenatide |
| L - A | 0 | 0 | . | .% | 0 | G: | Sitagliptin |
| B - A | 1.08 | 1 | 0.298 | 7.50% | 0.1118 | H: | Empagliflozin |
| O - A | 0 | 0 | . | .% | 0 | I: | Ertugliflozin |
| R - A | 0 | 0 | . | .% | 0 | J: | Vildagliptin |
| P - A | 2.88 | 4 | 0.578 | 0.00% | 0 | K: | Dapagliflozin |
| N - A | 0.04 | 1 | 0.837 | 0.00% | 0 | L: | Dulaglutide |
| R - B | 0 | 0 | . | .% | 0 | M: | Linagliptin |
| Q - A | 0.62 | 2 | 0.734 | 0.00% | 0 | N: | Sotagliflozin |
| G - A | 0.75 | 3 | 0.86 | 0.00% | 0 | O: | Exenatide |
| H - G | 0 | 0 | . | .% | 0 | P: | Semaglutide |
| M - A | 1.17 | 1 | 0.28 | 14.50% | 0.2157 | Q: | Tirzepatide |
| E - A | 0.84 | 1 | 0.36 | 0.00% | 0 | R: | Lixisenatide |
| J - A | 0 | 0 | . | .% | 0 |  |  |

**eTable 8C Heterogeneity for primary outcome: acute lymphocytic leukemia risk**

|  | Heterogeneity statistic | degrees of freedom | *p* | *I squared* | *Tau-squared* | Treatments used |  |
| --- | --- | --- | --- | --- | --- | --- | --- |
| C - A | 0 | 0 | . | .% | 0 | A: | Placebo_or_Control |
| E - A | 0.29 | 1 | 0.592 | 0.00% | 0 | B: | Semaglutide |
| F - A | 0 | 0 | . | .% | 0 | C: | Ertugliflozin |
| B - A | 0 | 0 | . | .% | 0 | D: | Linagliptin |
| D - A | 0 | 0 | . | .% | 0 | E: | Empagliflozin |
|  |  |  |  |  |  | F: | Sotagliflozin |

**eTable 8D Heterogeneity for primary outcome: acute myeloid leukemia risk**

|  | Heterogeneity statistic | degrees of freedom | *p* | *I squared* | *Tau-squared* | Treatments used |  |
| --- | --- | --- | --- | --- | --- | --- | --- |
| C - A | 1.55 | 2 | 0.461 | 0.00% | 0 | A: | Placebo_or_Control |
| D - A | 0 | 1 | 1 | 0.00% | 0 | B: | Liraglutide |
| I - A | 0 | 0 | . | .% | 0 | C: | Albiglutide |
| K - A | 3.1 | 3 | 0.377 | 3.20% | 0.0536 | D: | Canagliflozin |
| L - A | 0 | 0 | . | .% | 0 | E: | Semaglutide |
| B - A | 0 | 0 | . | .% | 0 | F: | Lixisenatide |
| O - A | 0 | 0 | . | .% | 0 | G: | Saxagliptin |
| H - A | 1.43 | 2 | 0.489 | 0.00% | 0 | H: | Empagliflozin |
| E - A | 0.04 | 1 | 0.843 | 0.00% | 0 | I: | Ertugliflozin |
| F - B | 0 | 0 | . | .% | 0 | J: | Tirzepatide |
| M - A | 0.14 | 1 | 0.704 | 0.00% | 0 | K: | Dapagliflozin |
| G - A | 2.24 | 1 | 0.134 | 55.40% | 3.0319 | L: | Dulaglutide |
| N - A | 0.24 | 1 | 0.622 | 0.00% | 0 | M: | Linagliptin |
| J - A | 0 | 0 | . | .% | 0 | N: | Sitagliptin |
|  |  |  |  |  |  | O: | Exenatide |

**eTable 8E Heterogeneity for primary outcome: chronic lymphocytic leukemia risk**

|  | Heterogeneity statistic | degrees of freedom | *p* | *I squared* | *Tau-squared* | Treatments used |  |
| --- | --- | --- | --- | --- | --- | --- | --- |
| C - A | 0 | 0 | . | .% | 0 | A: | Placebo_or_Control |
| D - A | 0 | 0 | . | .% | 0 | B: | Liraglutide |
| F - A | 0 | 0 | . | .% | 0 | C: | Albiglutide |
| H - A | 1.62 | 4 | 0.805 | 0.00% | 0 | D: | Canagliflozin |
| I - A | 0 | 0 | . | .% | 0 | E: | Vildagliptin |
| K - A | 3.33 | 3 | 0.343 | 10.00% | 0.1451 | F: | Efpeglenatide |
| L - A | 0 | 0 | . | .% | 0 | G: | Sitagliptin |
| B - A | 0 | 0 | . | .% | 0 | H: | Empagliflozin |
| O - A | 0 | 0 | . | .% | 0 | I: | Ertugliflozin |
| Q - A | 0 | 0 | . | .% | 0 | J: | Semaglutide |
| N - A | 0 | 0 | . | .% | 0 | K: | Dapagliflozin |
| J - A | 0.26 | 1 | 0.611 | 0.00% | 0 | L: | Dulaglutide |
| G - A | 0 | 1 | 1 | 0.00% | 0 | M: | Linagliptin |
| H - G | 0 | 0 | . | .% | 0 | N: | Sotagliflozin |
| M - A | 0.14 | 1 | 0.712 | 0.00% | 0 | O: | Exenatide |
| P - A | 0 | 0 | . | .% | 0 | P: | Saxagliptin |
| E - A | 0 | 0 | . | .% | 0 | Q: | Lixisenatide |

**eTable 8F Heterogeneity for primary outcome: chronic myeloid leukemia risk**

|  | Heterogeneity statistic | degrees of freedom | *p* | *I squared* | *Tau-squared* | Treatments used |  |
| --- | --- | --- | --- | --- | --- | --- | --- |
| C - A | 0 | 0 | . | .% | 0 | A: | Placebo_or_Control |
| D - A | 0.04 | 1 | 0.851 | 0.00% | 0 | B: | Liraglutide |
| H - A | 0.91 | 1 | 0.341 | 0.00% | 0 | C: | Albiglutide |
| K - A | 0.65 | 2 | 0.724 | 0.00% | 0 | D: | Canagliflozin |
| L - A | 0 | 0 | . | .% | 0 | E: | Semaglutide |
| B - A | 0.66 | 1 | 0.415 | 0.00% | 0 | F: | Tirzepatide |
| O - A | 0 | 0 | . | .% | 0 | G: | Vildagliptin |
| E - A | 0 | 0 | . | .% | 0 | H: | Empagliflozin |
| N - A | 0.09 | 1 | 0.76 | 0.00% | 0 | I: | Saxagliptin |
| F - A | 0 | 0 | . | .% | 0 | J: | Sitagliptin |
| M - A | 0 | 0 | . | .% | 0 | K: | Dapagliflozin |
| I - A | 0 | 0 | . | .% | 0 | L: | Dulaglutide |
| J - A | 1.89 | 1 | 0.17 | 47.00% | 2.1886 | M: | Linagliptin |
| G - A | 0 | 0 | . | .% | 0 | N: | Sotagliflozin |
|  |  |  |  |  |  | O: | Exenatide |

**eTable 8G Heterogeneity for primary outcome: lymphoma risk**

|  | Heterogeneity statistic | degrees of freedom | *p* | *I squared* | *Tau-squared* | Treatments used |  |
| --- | --- | --- | --- | --- | --- | --- | --- |
| C - A | 0.02 | 1 | 0.894 | 0.00% | 0 | A: | Placebo_or_Control |
| D - A | 2.87 | 2 | 0.239 | 30.20% | 0.6912 | B: | Liraglutide |
| F - A | 0 | 0 | . | .% | 0 | C: | Albiglutide |
| H - A | 3.55 | 3 | 0.314 | 15.60% | 0.1741 | D: | Canagliflozin |
| I - A | 0 | 1 | 0.952 | 0.00% | 0 | E: | Omarigliptin |
| K - A | 2.74 | 2 | 0.255 | 26.90% | 0.1691 | F: | Efpeglenatide |
| L - A | 0.06 | 1 | 0.801 | 0.00% | 0 | G: | Ertugliflozin_Sitagliptin |
| B - A | 0.42 | 2 | 0.812 | 0.00% | 0 | H: | Empagliflozin |
| O - A | 0 | 0 | . | .% | 0 | I: | Ertugliflozin |
| R - A | 0 | 0 | . | .% | 0 | J: | Alogliptin |
| P - A | 1 | 4 | 0.91 | 0.00% | 0 | K: | Dapagliflozin |
| N - A | 2.73 | 2 | 0.255 | 26.80% | 0.7804 | L: | Dulaglutide |
| U - P | 0 | 0 | . | .% | 0 | M: | Linagliptin |
| S - A | 0.12 | 2 | 0.943 | 0.00% | 0 | N: | Sotagliflozin |
| S - P | 0 | 0 | . | .% | 0 | O: | Exenatide |
| V - A | 0 | 0 | . | .% | 0 | P: | Semaglutide |
| J - A | 0.09 | 1 | 0.764 | 0.00% | 0 | Q: | Vildagliptin |
| M - A | 1.21 | 1 | 0.271 | 17.50% | 0.1675 | R: | Lixisenatide |
| E - A | 0 | 0 | . | .% | 0 | S: | Tirzepatide |
| T - A | 0.25 | 2 | 0.884 | 0.00% | 0 | T: | Saxagliptin |
| U - A | 0.98 | 3 | 0.807 | 0.00% | 0 | U: | Sitagliptin |
| I - G | 0 | 0 | . | .% | 0 | V: | Bexagliflozin |
| U - G | 0 | 0 | . | .% | 0 |  |  |
| U - I | 0 | 0 | . | .% | 0 |  |  |
| Q - A | 0 | 0 | . | .% | 0 |  |  |

**eTable 8H Heterogeneity for primary outcome: non-Hodgkin's lymphoma risk**

|  | Heterogeneity statistic | degrees of freedom | *p* | *I squared* | *Tau-squared* | Treatments used |  |
| --- | --- | --- | --- | --- | --- | --- | --- |
| C - A | 0.02 | 1 | 0.894 | 0.00% | 0 | A: | Placebo_or_Control |
| D - A | 2.05 | 2 | 0.36 | 2.20% | 0.0369 | B: | Liraglutide |
| F - A | 0 | 0 | . | .% | 0 | C: | Albiglutide |
| H - A | 3.85 | 3 | 0.278 | 22.10% | 0.2902 | D: | Canagliflozin |
| I - A | 0.05 | 1 | 0.825 | 0.00% | 0 | E: | Omarigliptin |
| K - A | 1.78 | 2 | 0.411 | 0.00% | 0 | F: | Efpeglenatide |
| L - A | 0.1 | 1 | 0.746 | 0.00% | 0 | G: | Alogliptin |
| B - A | 0.4 | 1 | 0.529 | 0.00% | 0 | H: | Empagliflozin |
| O - A | 0 | 0 | . | .% | 0 | I: | Ertugliflozin |
| R - A | 0 | 0 | . | .% | 0 | J: | Ertugliflozin_Sitagliptin |
| P - A | 2.02 | 3 | 0.568 | 0.00% | 0 | K: | Dapagliflozin |
| N - A | 2.73 | 2 | 0.255 | 26.80% | 0.7804 | L: | Dulaglutide |
| T - P | 0 | 0 | . | .% | 0 | M: | Linagliptin |
| S - A | 0.12 | 2 | 0.943 | 0.00% | 0 | N: | Sotagliflozin |
| S - P | 0 | 0 | . | .% | 0 | O: | Exenatide |
| V - A | 0 | 0 | . | .% | 0 | P: | Semaglutide |
| G - A | 0.09 | 1 | 0.764 | 0.00% | 0 | Q: | Saxagliptin |
| M - A | 1 | 1 | 0.317 | 0.10% | 0.0008 | R: | Lixisenatide |
| E - A | 0 | 0 | . | .% | 0 | S: | Tirzepatide |
| Q - A | 0.06 | 1 | 0.801 | 0.00% | 0 | T: | Sitagliptin |
| T - A | 0.45 | 2 | 0.797 | 0.00% | 0 | U: | Vildagliptin |
| J - I | 0 | 0 | . | .% | 0 | V: | Bexagliflozin |
| T - I | 0 | 0 | . | .% | 0 |  |  |
| T - J | 0 | 0 | . | .% | 0 |  |  |
| U - A | 0 | 0 | . | .% | 0 |  |  |

**eTable 8I Heterogeneity for primary outcome: B cell non-Hodgkin's lymphoma risk**

|  | Heterogeneity statistic | degrees of freedom | *p* | *I squared* | *Tau-squared* | Treatments used |  |
| --- | --- | --- | --- | --- | --- | --- | --- |
| C - A | 0.02 | 1 | 0.894 | 0.00% | 0 | A: | Placebo_or_Control |
| D - A | 1.84 | 2 | 0.398 | 0.00% | 0 | B: | Liraglutide |
| F - A | 0 | 0 | . | .% | 0 | C: | Albiglutide |
| H - A | 2.5 | 3 | 0.475 | 0.00% | 0 | D: | Canagliflozin |
| I - A | 0 | 0 | . | .% | 0 | E: | Saxagliptin |
| K - A | 0.51 | 2 | 0.776 | 0.00% | 0 | F: | Efpeglenatide |
| L - A | 0 | 0 | . | .% | 0 | G: | Sitagliptin |
| B - A | 0.34 | 1 | 0.558 | 0.00% | 0 | H: | Empagliflozin |
| O - A | 0 | 0 | . | .% | 0 | I: | Ertugliflozin |
| R - A | 0 | 0 | . | .% | 0 | J: | Ertugliflozin_Sitagliptin |
| P - A | 1.68 | 3 | 0.641 | 0.00% | 0 | K: | Dapagliflozin |
| N - A | 1.48 | 2 | 0.476 | 0.00% | 0 | L: | Dulaglutide |
| P - G | 0 | 0 | . | .% | 0 | M: | Linagliptin |
| S - A | 0 | 0 | . | .% | 0 | N: | Sotagliflozin |
| T - A | 0 | 0 | . | .% | 0 | O: | Exenatide |
| M - A | 0.33 | 1 | 0.565 | 0.00% | 0 | P: | Semaglutide |
| E - A | 0 | 0 | . | .% | 0 | Q: | Vildagliptin |
| G - A | 0.95 | 2 | 0.623 | 0.00% | 0 | R: | Lixisenatide |
| I - G | 0 | 0 | . | .% | 0 | S: | Tirzepatide |
| J - G | 0 | 0 | . | .% | 0 | T: | Bexagliflozin |
| J - I | 0 | 0 | . | .% | 0 |  |  |
| Q - A | 0 | 0 | . | .% | 0 |  |  |

**eTable 8J Heterogeneity for primary outcome: T cell non-Hodgkin's lymphoma risk**

|  | Heterogeneity statistic | degrees of freedom | *p* | *I squared* | *Tau-squared* | Treatments used |  |
| --- | --- | --- | --- | --- | --- | --- | --- |
| F - A | 0 | 0 | . | .% | 0 | A: | Placebo_or_Control |
| C - A | 0.91 | 1 | 0.341 | 0.00% | 0 | B: | Sitagliptin |
| E - A | 2.56 | 2 | 0.278 | 21.90% | 0.7097 | C: | Empagliflozin |
| D - A | 0 | 0 | . | .% | 0 | D: | Exenatide |
| B - A | 0 | 0 | . | .% | 0 | E: | Dapagliflozin |
|  |  |  |  |  |  | F: | Efpeglenatide |

**eTable 8K Heterogeneity for primary outcome: myeloma risk**

|  | Heterogeneity statistic | degrees of freedom | *p* | *I squared* | *Tau-squared* | Treatments used |  |
| --- | --- | --- | --- | --- | --- | --- | --- |
| C - A | 0 | 0 | . | .% | 0 | A: | Placebo_or_Control |
| D - A | 0 | 1 | 1 | 0.00% | 0 | B: | Liraglutide |
| H - A | 0.84 | 3 | 0.84 | 0.00% | 0 | C: | Albiglutide |
| I - A | 0.92 | 1 | 0.337 | 0.00% | 0 | D: | Canagliflozin |
| K - A | 2.86 | 4 | 0.581 | 0.00% | 0 | E: | Sitagliptin |
| L - A | 0.16 | 1 | 0.688 | 0.00% | 0 | F: | Semaglutide |
| B - A | 0 | 0 | . | .% | 0 | G: | Alogliptin |
| O - A | 0 | 0 | . | .% | 0 | H: | Empagliflozin |
| R - A | 0 | 0 | . | .% | 0 | I: | Ertugliflozin |
| F - A | 0.91 | 2 | 0.636 | 0.00% | 0 | J: | Tirzepatide |
| N - A | 0.7 | 1 | 0.403 | 0.00% | 0 | K: | Dapagliflozin |
| F - E | 0 | 0 | . | .% | 0 | L: | Dulaglutide |
| O - B | 0 | 0 | . | .% | 0 | M: | Linagliptin |
| J - A | 0 | 0 | . | .% | 0 | N: | Sotagliflozin |
| G - A | 0 | 0 | . | .% | 0 | O: | Exenatide |
| M - A | 1.49 | 1 | 0.223 | 32.70% | 0.5256 | P: | Omarigliptin |
| P - A | 0 | 0 | . | .% | 0 | Q: | Saxagliptin |
| Q - A | 0.36 | 2 | 0.836 | 0.00% | 0 | R: | Lixisenatide |
| E - A | 0 | 0 | . | .% | 0 |  |  |

**eTable 8L Heterogeneity for primary outcome: plasma cell myeloma risk**

|  | Heterogeneity statistic | degrees of freedom | *p* | *I squared* | *Tau-squared* | Treatments used |  |
| --- | --- | --- | --- | --- | --- | --- | --- |
| C - A | 0 | 0 | . | .% | 0 | A: | Placebo_or_Control |
| D - A | 0 | 1 | 1 | 0.00% | 0 | B: | Liraglutide |
| H - A | 0.84 | 3 | 0.84 | 0.00% | 0 | C: | Albiglutide |
| I - A | 0.92 | 1 | 0.337 | 0.00% | 0 | D: | Canagliflozin |
| K - A | 1.81 | 3 | 0.612 | 0.00% | 0 | E: | Sitagliptin |
| L - A | 0 | 0 | . | .% | 0 | F: | Semaglutide |
| B - A | 0 | 0 | . | .% | 0 | G: | Tirzepatide |
| O - A | 0 | 0 | . | .% | 0 | H: | Empagliflozin |
| R - A | 0 | 0 | . | .% | 0 | I: | Ertugliflozin |
| F - A | 0.91 | 2 | 0.636 | 0.00% | 0 | J: | Saxagliptin |
| N - A | 0.7 | 1 | 0.403 | 0.00% | 0 | K: | Dapagliflozin |
| F - E | 0 | 0 | . | .% | 0 | L: | Dulaglutide |
| G - A | 0 | 0 | . | .% | 0 | M: | Linagliptin |
| P - A | 0 | 0 | . | .% | 0 | N: | Sotagliflozin |
| M - A | 1.49 | 1 | 0.223 | 32.70% | 0.5256 | O: | Exenatide |
| Q - A | 0 | 0 | . | .% | 0 | P: | Alogliptin |
| J - A | 0 | 0 | . | .% | 0 | Q: | Omarigliptin |
| E - A | 0 | 0 | . | .% | 0 | R: | Lixisenatide |

*Abbreviation: 95%CIs: 95% confidence intervals; DPP4 inhibitor: dipeptidyl-peptidase 4 inhibitor; GLP-1 agonist: glucagon-like peptide-1 agonist; NMA: network meta-analysis; RR: risk ratio; RCT: randomized controlled trial; SGLT2 inhibitor: sodium–glucose cotransporter 2 inhibitor*

**eTable 9A Side-splitting model inconsistency for primary outcome: overall hematologic malignancy risk**

| Side | Direct |  | Indirect |  | Difference |  |  | tau |  |  |
| --- | --- | --- | --- | --- | --- | --- | --- | --- | --- | --- |
|  | Coef. | Std. Err. | Coef. | Std. Err. | Coef. | Std. Err. | P>z |  | Treatments used |  |
| A B | -0.1590046 | 0.3070055 | -0.2340399 | 1.2043 | 0.0750353 | 1.242816 | 0.952 | 7.41E-07 | A: | Placebo_or_Control |
| A C | . | . | . | . | . | . | . | . | B: | Liraglutide |
| A D | . | . | . | . | . | . | . | . | C: | Albiglutide |
| A F | . | . | . | . | . | . | . | . | D: | Canagliflozin |
| A G | . | . | . | . | . | . | . | . | E: | Ertugliflozin_Sitagliptin |
| A H * | 0.4656337 | 0.3152946 | -1.06684 | 3.203331 | 1.532473 | 3.234185 | 0.636 | 7.47E-07 | F: | Efpeglenatide |
| A I | 0.2868878 | 0.4783308 | -0.741906 | 2.017069 | 1.028794 | 2.07301 | 0.62 | 2.21E-08 | G: | Omarigliptin |
| A J | . | . | . | . | . | . | . | . | H: | Empagliflozin |
| A K | . | . | . | . | . | . | . | . | I: | Ertugliflozin |
| A L | . | . | . | . | . | . | . | . | J: | Vildagliptin |
| A M | . | . | . | . | . | . | . | . | K: | Dapagliflozin |
| A N | . | . | . | . | . | . | . | . | L: | Dulaglutide |
| A O | 0.0482571 | 0.29776 | 0.9777706 | 1.658187 | -0.9295135 | 1.684709 | 0.581 | 2.53E-06 | M: | Linagliptin |
| A P | -0.3702241 | 0.3441432 | 0.6670016 | 1.177207 | -1.037226 | 1.226479 | 0.398 | 0.0000233 | N: | Sotagliflozin |
| A Q | . | . | . | . | . | . | . | . | O: | Exenatide |
| A R | -0.4051352 | 0.644986 | -1.287612 | 1.657691 | 0.8824768 | 1.778748 | 0.62 | 6.37E-08 | P: | Semaglutide |
| A S | -1.335172 | 0.6992995 | -2.527034 | 1.666389 | 1.191861 | 1.807172 | 0.51 | 7.39E-07 | Q: | Alogliptin |
| A T | . | . | . | . | . | . | . | . | R: | Lixisenatide |
| A U | -0.0405444 | 0.2788168 | 0.2345326 | 1.134428 | -0.275077 | 1.168189 | 0.814 | 0.0000489 | S: | Tirzepatide |
| A V | . | . | . | . | . | . | . | . | T: | Saxagliptin |
| B O | 1.111406 | 1.63038 | 0.1818918 | 0.4243918 | 0.9295138 | 1.684709 | 0.581 | 3.20E-07 | U: | Sitagliptin |
| B R | -1.098612 | 1.629974 | -0.2161354 | 0.7121323 | -0.8824769 | 1.778749 | 0.62 | 7.37E-06 | V: | Bexagliflozin |
| E I * | -1.120903 | 1.631752 | 0.9366845 | 3.443851 | -2.057587 | 4.14602 | 0.62 | 2.18E-06 |  |  |
| E U * | -0.4217256 | 1.63113 | -2.479321 | 3.444734 | 2.057595 | 4.14602 | 0.62 | 6.79E-06 |  |  |
| H U | 0.6106272 | 1.577232 | -0.5475739 | 0.4227331 | 1.158201 | 1.634989 | 0.479 | 2.65E-07 |  |  |
| I U * | 0.6991773 | 1.99849 | -0.3296164 | 0.550823 | 1.028794 | 2.07301 | 0.62 | 7.51E-07 |  |  |
| P S | -2.197225 | 1.632124 | -1.005363 | 0.775913 | -1.191861 | 1.807172 | 0.51 | 2.64E-07 |  |  |
| P U | -0.5144009 | 1.548271 | 0.3252573 | 0.4355015 | -0.8396582 | 1.608354 | 0.602 | 9.09E-07 |  |  |

**eTable 9B Side-splitting model inconsistency for primary outcome: leukemia risk**

| Side | Direct |  | Indirect |  | Difference |  |  | tau |  |  |
| --- | --- | --- | --- | --- | --- | --- | --- | --- | --- | --- |
|  | Coef. | Std. Err. | Coef. | Std. Err. | Coef. | Std. Err. | P>z |  | Treatments used |  |
| A B | -0.5843209 | 0.5599456 | 1.098942 | 2.157812 | -1.683263 | 2.22928 | 0.45 | 4.67E-07 | A: | Placebo_or_Control |
| A C | . | . | . | . | . | . | . | . | B: | Liraglutide |
| A D | . | . | . | . | . | . | . | . | C: | Albiglutide |
| A E | . | . | . | . | . | . | . | . | D: | Canagliflozin |
| A F | . | . | . | . | . | . | . | . | E: | Saxagliptin |
| A G * | 0.1642174 | 0.4467931 | 2.274574 | 2.831612 | -2.110357 | 2.866645 | 0.462 | 4.33E-07 | F: | Efpeglenatide |
| A H * | 0.5113837 | 0.5543467 | -0.6498788 | 3.294218 | 1.161263 | 3.386126 | 0.732 | 2.10E-08 | G: | Sitagliptin |
| A I | . | . | . | . | . | . | . | . | H: | Empagliflozin |
| A J | . | . | . | . | . | . | . | . | I: | Ertugliflozin |
| A K | . | . | . | . | . | . | . | . | J: | Vildagliptin |
| A L | . | . | . | . | . | . | . | . | K: | Dapagliflozin |
| A M | . | . | . | . | . | . | . | . | L: | Dulaglutide |
| A N | . | . | . | . | . | . | . | . | M: | Linagliptin |
| A O | . | . | . | . | . | . | . | . | N: | Sotagliflozin |
| A P | . | . | . | . | . | . | . | . | O: | Exenatide |
| A Q | . | . | . | . | . | . | . | . | P: | Semaglutide |
| A R | 0.0003299 | 1.41398 | -1.682933 | 1.723471 | 1.683263 | 2.229281 | 0.45 | 4.61E-07 | Q: | Tirzepatide |
| B R | -1.098612 | 1.629974 | 0.5846508 | 1.520815 | -1.683263 | 2.229281 | 0.45 | 1.83E-07 | R: | Lixisenatide |
| G H | -0.6493627 | 1.57898 | 0.4542167 | 0.745724 | -1.103579 | 1.755525 | 0.53 | 1.45E-07 |  |  |

**eTable 9C Side-splitting model inconsistency for primary outcome: acute lymphocytic leukemia risk**

| Side | Direct |  | Indirect |  | Difference |  |  | tau | Treatments used |  |
| --- | --- | --- | --- | --- | --- | --- | --- | --- | --- | --- |
|  | Coef. | Std. Err. | Coef. | Std. Err. | Coef. | Std. Err. | P>z |  | A: | Placebo_or_Control |
| A B | . | . | . | . | . | . | . | . | B: | Semaglutide |
| A C | . | . | . | . | . | . | . | . | C: | Ertugliflozin |
| A D | . | . | . | . | . | . | . | . | D: | Linagliptin |
| A E | . | . | . | . | . | . | . | . | E: | Empagliflozin |
| A F | . | . | . | . | . | . | . | . | F: | Sotagliflozin |

**eTable 9D Side-splitting model inconsistency for primary outcome: acute myeloid leukemia risk**

| Side | Direct |  | Indirect |  | Difference |  |  | tau | Treatments used |  |
| --- | --- | --- | --- | --- | --- | --- | --- | --- | --- | --- |
|  | Coef. | Std. Err. | Coef. | Std. Err. | Coef. | Std. Err. | P>z |  | A: | Placebo_or_Control |
| A B * | 0.0008565 | 0.9997858 | 0.4825931 | 5859.209 | -0.4817366 | 5859.209 | 1 | 6.38E-06 | B: | Liraglutide |
| A C | . | . | . | . | . | . | . | . | C: | Albiglutide |
| A D | . | . | . | . | . | . | . | . | D: | Canagliflozin |
| A E | . | . | . | . | . | . | . | . | E: | Semaglutide |
| A G | . | . | . | . | . | . | . | . | F: | Lixisenatide |
| A H | . | . | . | . | . | . | . | . | G: | Saxagliptin |
| A I | . | . | . | . | . | . | . | . | H: | Empagliflozin |
| A J | . | . | . | . | . | . | . | . | I: | Ertugliflozin |
| A K | . | . | . | . | . | . | . | . | J: | Tirzepatide |
| A L | . | . | . | . | . | . | . | . | K: | Dapagliflozin |
| A M | . | . | . | . | . | . | . | . | L: | Dulaglutide |
| A N | . | . | . | . | . | . | . | . | M: | Linagliptin |
| A O | . | . | . | . | . | . | . | . | N: | Sitagliptin |
| B F * | -1.098613 | 1.629974 | 4.715392 | 7404.851 | -5.814005 | 7404.851 | 0.999 | 0.0000293 | O: | Exenatide |

**eTable 9E Side-splitting model inconsistency for primary outcome: chronic lymphocytic leukemia risk**

| Side | Direct |  | Indirect |  | Difference |  |  | tau |  |  |
| --- | --- | --- | --- | --- | --- | --- | --- | --- | --- | --- |
|  | Coef. | Std. Err. | Coef. | Std. Err. | Coef. | Std. Err. | P>z |  | Treatments used |  |
| A B | . | . | . | . | . | . | . | . | A: | Placebo_or_Control |
| A C | . | . | . | . | . | . | . | . | B: | Liraglutide |
| A D | . | . | . | . | . | . | . | . | C: | Albiglutide |
| A E | . | . | . | . | . | . | . | . | D: | Canagliflozin |
| A F | . | . | . | . | . | . | . | . | E: | Vildagliptin |
| A G * | 0.0009774 | 0.666245 | 1.663751 | 3.030857 | -1.662774 | 3.10322 | 0.592 | 3.89E-10 | F: | Efpeglenatide |
| A H * | 0.1761073 | 0.7178136 | -0.826196 | 3.473009 | 1.002303 | 3.618183 | 0.782 | 4.04E-10 | G: | Sitagliptin |
| A I | . | . | . | . | . | . | . | . | H: | Empagliflozin |
| A J | . | . | . | . | . | . | . | . | I: | Ertugliflozin |
| A K | . | . | . | . | . | . | . | . | J: | Semaglutide |
| A L | . | . | . | . | . | . | . | . | K: | Dapagliflozin |
| A M | . | . | . | . | . | . | . | . | L: | Dulaglutide |
| A N | . | . | . | . | . | . | . | . | M: | Linagliptin |
| A O | . | . | . | . | . | . | . | . | N: | Sotagliflozin |
| A P | . | . | . | . | . | . | . | . | O: | Exenatide |
| A Q | . | . | . | . | . | . | . | . | P: | Saxagliptin |
| G H | -0.5878861 | 1.581438 | 0.3268251 | 1.066622 | -0.9147113 | 1.915766 | 0.633 | 1.45E-09 | Q: | Lixisenatide |

**eTable 9F Side-splitting model inconsistency for primary outcome: chronic myeloid leukemia risk**

| Side | Direct |  | Indirect |  | Difference |  |  | tau | Treatments used |  |
| --- | --- | --- | --- | --- | --- | --- | --- | --- | --- | --- |
|  | Coef. | Std. Err. | Coef. | Std. Err. | Coef. | Std. Err. | P>z |  | A: | Placebo_or_Control |
| A B | . | . | . | . | . | . | . | . | B: | Liraglutide |
| A C | . | . | . | . | . | . | . | . | C: | Albiglutide |
| A D | . | . | . | . | . | . | . | . | D: | Canagliflozin |
| A E | . | . | . | . | . | . | . | . | E: | Semaglutide |
| A F | . | . | . | . | . | . | . | . | F: | Tirzepatide |
| A G | . | . | . | . | . | . | . | . | G: | Vildagliptin |
| A H | . | . | . | . | . | . | . | . | H: | Empagliflozin |
| A I | . | . | . | . | . | . | . | . | I: | Saxagliptin |
| A J | . | . | . | . | . | . | . | . | J: | Sitagliptin |
| A K | . | . | . | . | . | . | . | . | K: | Dapagliflozin |
| A L | . | . | . | . | . | . | . | . | L: | Dulaglutide |
| A M | . | . | . | . | . | . | . | . | M: | Linagliptin |
| A N | . | . | . | . | . | . | . | . | N: | Sotagliflozin |
| A O | . | . | . | . | . | . | . | . | O: | Exenatide |

**eTable 9G Side-splitting model inconsistency for primary outcome: lymphoma risk**

| Side | Direct |  | Indirect |  | Difference |  |  | tau |  |  |
| --- | --- | --- | --- | --- | --- | --- | --- | --- | --- | --- |
|  | Coef. | Std. Err. | Coef. | Std. Err. | Coef. | Std. Err. | P>z |  | Treatments used |  |
| A B | . | . | . | . | . | . | . | . | A: | Placebo_or_Control |
| A C | . | . | . | . | . | . | . | . | B: | Liraglutide |
| A D | . | . | . | . | . | . | . | . | C: | Albiglutide |
| A E | . | . | . | . | . | . | . | . | D: | Canagliflozin |
| A F | . | . | . | . | . | . | . | . | E: | Omarigliptin |
| A H | . | . | . | . | . | . | . | . | F: | Efpeglenatide |
| A I | 0.3026424 | 0.6249045 | -0.5405884 | 2.037006 | 0.8432308 | 2.130704 | 0.692 | 9.69E-06 | G: | Ertugliflozin_Sitagliptin |
| A J | . | . | . | . | . | . | . | . | H: | Empagliflozin |
| A K | . | . | . | . | . | . | . | . | I: | Ertugliflozin |
| A L | . | . | . | . | . | . | . | . | J: | Alogliptin |
| A M | . | . | . | . | . | . | . | . | K: | Dapagliflozin |
| A N | . | . | . | . | . | . | . | . | L: | Dulaglutide |
| A O | . | . | . | . | . | . | . | . | M: | Linagliptin |
| A P | -0.4431612 | 0.4758355 | 0.5102344 | 1.254001 | -0.9533956 | 1.341245 | 0.477 | 4.25E-07 | N: | Sotagliflozin |
| A Q | . | . | . | . | . | . | . | . | O: | Exenatide |
| A R | . | . | . | . | . | . | . | . | P: | Semaglutide |
| A S | -1.323792 | 0.9420589 | -2.591014 | 1.69512 | 1.267222 | 1.939306 | 0.513 | 5.73E-07 | Q: | Vildagliptin |
| A T | . | . | . | . | . | . | . | . | R: | Lixisenatide |
| A U | 0.1886568 | 0.405351 | 0.1747899 | 1.317955 | 0.0138669 | 1.378882 | 0.992 | 2.25E-08 | S: | Tirzepatide |
| A V | . | . | . | . | . | . | . | . | T: | Saxagliptin |
| G I * | -1.120903 | 1.631752 | 0.5655587 | 3.581931 | -1.686462 | 4.261409 | 0.692 | 0.0000302 | U: | Sitagliptin |
| G U * | -0.4217256 | 1.63113 | -2.108187 | 3.582781 | 1.686462 | 4.261409 | 0.692 | 0.0000131 | V: | Bexagliflozin |
| I U * | 0.6991773 | 1.99849 | -0.1440535 | 0.7388756 | 0.8432308 | 2.130704 | 0.692 | 0.0000258 |  |  |
| P S | -2.197225 | 1.632124 | -0.9300029 | 1.047415 | -1.267222 | 1.939306 | 0.513 | 3.20E-07 |  |  |
| P U | -0.0035753 | 1.632118 | 0.5822802 | 0.6092809 | -0.5858554 | 1.742134 | 0.737 | 1.11E-06 |  |  |

**eTable 9H Side-splitting model inconsistency for primary outcome: non-Hodgkin's lymphoma risk**

| Side | Direct |  | Indirect |  | Difference |  |  | tau |  |  |
| --- | --- | --- | --- | --- | --- | --- | --- | --- | --- | --- |
|  | Coef. | Std. Err. | Coef. | Std. Err. | Coef. | Std. Err. | P>z |  | Treatments used |  |
| A B | . | . | . | . | . | . | . | . | A: | Placebo_or_Control |
| A C | . | . | . | . | . | . | . | . | B: | Liraglutide |
| A D | . | . | . | . | . | . | . | . | C: | Albiglutide |
| A E | . | . | . | . | . | . | . | . | D: | Canagliflozin |
| A F | . | . | . | . | . | . | . | . | E: | Omarigliptin |
| A G | . | . | . | . | . | . | . | . | F: | Efpeglenatide |
| A H | . | . | . | . | . | . | . | . | G: | Alogliptin |
| A I | 0.0616687 | 0.6484016 | -0.6212521 | 2.042678 | 0.6829208 | 2.143118 | 0.75 | 8.43E-08 | H: | Empagliflozin |
| A K | . | . | . | . | . | . | . | . | I: | Ertugliflozin |
| A L | . | . | . | . | . | . | . | . | J: | Ertugliflozin_Sitagliptin |
| A M | . | . | . | . | . | . | . | . | K: | Dapagliflozin |
| A N | . | . | . | . | . | . | . | . | L: | Dulaglutide |
| A O | . | . | . | . | . | . | . | . | M: | Linagliptin |
| A P | -0.5003783 | 0.5188703 | 0.4663353 | 1.256957 | -0.9667136 | 1.359841 | 0.477 | 2.22E-07 | N: | Sotagliflozin |
| A Q | . | . | . | . | . | . | . | . | O: | Exenatide |
| A R | . | . | . | . | . | . | . | . | P: | Semaglutide |
| A S | -1.323792 | 0.9420589 | -2.642257 | 1.705811 | 1.318465 | 1.948657 | 0.499 | 1.19E-06 | Q: | Saxagliptin |
| A T | 0.1095895 | 0.4361471 | 0.0572924 | 1.324887 | 0.0522972 | 1.394829 | 0.97 | 2.68E-07 | R: | Lixisenatide |
| A U | . | . | . | . | . | . | . | . | S: | Tirzepatide |
| A V | . | . | . | . | . | . | . | . | T: | Sitagliptin |
| I J * | 1.120903 | 1.631752 | -0.2449388 | 3.611434 | 1.365842 | 4.286237 | 0.75 | 2.94E-06 | U: | Vildagliptin |
| I T * | 0.6991773 | 1.99849 | 0.0162563 | 0.7739459 | 0.682921 | 2.143118 | 0.75 | 5.31E-06 | V: | Bexagliflozin |
| J T * | -0.4217256 | 1.63113 | -1.787567 | 3.612276 | 1.365842 | 4.286237 | 0.75 | 8.87E-07 |  |  |
| P S | -2.197225 | 1.632124 | -0.8787598 | 1.064629 | -1.318465 | 1.948657 | 0.499 | 6.31E-07 |  |  |
| P T | -0.0035753 | 1.632118 | 0.540062 | 0.6577387 | -0.5436373 | 1.759667 | 0.757 | 0.0000202 |  |  |

**eTable 9I Side-splitting model inconsistency for primary outcome: B cell non-Hodgkin's lymphoma risk**

| Side | Direct |  | Indirect |  | Difference |  |  | tau |  |  |
| --- | --- | --- | --- | --- | --- | --- | --- | --- | --- | --- |
|  | Coef. | Std. Err. | Coef. | Std. Err. | Coef. | Std. Err. | P>z |  | Treatments used |  |
| A B | . | . | . | . | . | . | . | . | A: | Placebo_or_Control |
| A C | . | . | . | . | . | . | . | . | B: | Liraglutide |
| A D | . | . | . | . | . | . | . | . | C: | Albiglutide |
| A E | . | . | . | . | . | . | . | . | D: | Canagliflozin |
| A F | . | . | . | . | . | . | . | . | E: | Saxagliptin |
| A G | -0.146566 | 0.6169382 | -0.1386996 | 1.335797 | -0.0078664 | 1.471383 | 0.996 | 5.30E-07 | F: | Efpeglenatide |
| A H | . | . | . | . | . | . | . | . | G: | Sitagliptin |
| A I | -0.0005463 | 0.7067203 | -0.9076908 | 2.081155 | 0.9071445 | 2.197876 | 0.68 | 2.29E-07 | H: | Empagliflozin |
| A K | . | . | . | . | . | . | . | . | I: | Ertugliflozin |
| A L | . | . | . | . | . | . | . | . | J: | Ertugliflozin_Sitagliptin |
| A M | . | . | . | . | . | . | . | . | K: | Dapagliflozin |
| A N | . | . | . | . | . | . | . | . | L: | Dulaglutide |
| A O | . | . | . | . | . | . | . | . | M: | Linagliptin |
| A P | -0.6866416 | 0.5439031 | -0.0769893 | 1.736289 | -0.6096523 | 1.819486 | 0.738 | 4.20E-07 | N: | Sotagliflozin |
| A Q | . | . | . | . | . | . | . | . | O: | Exenatide |
| A R | . | . | . | . | . | . | . | . | P: | Semaglutide |
| A S | . | . | . | . | . | . | . | . | Q: | Vildagliptin |
| A T | . | . | . | . | . | . | . | . | R: | Lixisenatide |
| G I * | -0.6991773 | 1.99849 | 0.2079672 | 0.9147112 | -0.9071445 | 2.197876 | 0.68 | 8.19E-07 | S: | Tirzepatide |
| G J * | 0.4217256 | 1.63113 | 2.236015 | 3.741572 | -1.814289 | 4.395753 | 0.68 | 1.08E-06 | T: | Bexagliflozin |
| G P | 0.0035753 | 1.632118 | -0.6060771 | 0.8041898 | 0.6096523 | 1.819486 | 0.738 | 1.31E-07 |  |  |
| I J * | 1.120903 | 1.631752 | -0.693386 | 3.740758 | 1.814289 | 4.395752 | 0.68 | 1.45E-07 |  |  |

**eTable 9J Side-splitting model inconsistency for primary outcome: T cell non-Hodgkin's lymphoma risk**

| Side | Direct |  | Indirect |  | Difference |  |  | tau | Treatments used |  |
| --- | --- | --- | --- | --- | --- | --- | --- | --- | --- | --- |
|  | Coef. | Std. Err. | Coef. | Std. Err. | Coef. | Std. Err. | P>z |  | A: | Placebo_or_Control |
| A B | . | . | . | . | . | . | . | . | B: | Sitagliptin |
| A C | . | . | . | . | . | . | . | . | C: | Empagliflozin |
| A D | . | . | . | . | . | . | . | . | D: | Exenatide |
| A E | . | . | . | . | . | . | . | . | E: | Dapagliflozin |
| A F | . | . | . | . | . | . | . | . | F: | Efpeglenatide |

**eTable 9K Side-splitting model inconsistency for primary outcome: myeloma risk**

| Side | Direct |  | Indirect |  | Difference |  |  | tau |  |  |
| --- | --- | --- | --- | --- | --- | --- | --- | --- | --- | --- |
|  | Coef. | Std. Err. | Coef. | Std. Err. | Coef. | Std. Err. | P>z |  | Treatments used |  |
| A B | -0.5099691 | 0.7300035 | -1.1076 | 1.748675 | 0.5976311 | 1.894933 | 0.752 | 1.96E-07 | A: | Placebo_or_Control |
| A C | . | . | . | . | . | . | . | . | B: | Liraglutide |
| A D | . | . | . | . | . | . | . | . | C: | Albiglutide |
| A E | -1.790659 | 1.079996 | -0.0037651 | 1.798542 | -1.786894 | 2.097891 | 0.394 | 1.21E-06 | D: | Canagliflozin |
| A F | -0.0001898 | 0.7556093 | -1.787084 | 1.95709 | 1.786894 | 2.097891 | 0.394 | 1.21E-06 | E: | Sitagliptin |
| A G | . | . | . | . | . | . | . | . | F: | Semaglutide |
| A H | . | . | . | . | . | . | . | . | G: | Alogliptin |
| A I | . | . | . | . | . | . | . | . | H: | Empagliflozin |
| A J | . | . | . | . | . | . | . | . | I: | Ertugliflozin |
| A K | . | . | . | . | . | . | . | . | J: | Tirzepatide |
| A L | . | . | . | . | . | . | . | . | K: | Dapagliflozin |
| A M | . | . | . | . | . | . | . | . | L: | Dulaglutide |
| A N | . | . | . | . | . | . | . | . | M: | Linagliptin |
| A O | 0.0038054 | 0.6322406 | 0.6014365 | 1.786349 | -0.5976311 | 1.894933 | 0.752 | 1.96E-07 | N: | Sotagliflozin |
| A P | . | . | . | . | . | . | . | . | O: | Exenatide |
| A Q | . | . | . | . | . | . | . | . | P: | Omarigliptin |
| A R | . | . | . | . | . | . | . | . | Q: | Saxagliptin |
| B O | 1.111406 | 1.63038 | 0.5137745 | 0.9657294 | 0.5976312 | 1.894933 | 0.752 | 1.96E-07 | R: | Lixisenatide |
| E F | 0.0035753 | 1.632118 | 1.790469 | 1.318081 | -1.786894 | 2.097891 | 0.394 | 1.21E-06 |  |  |

**eTable 9L Side-splitting model inconsistency for primary outcome: plasma cell myeloma risk**

| Side | Direct |  | Indirect |  | Difference |  |  | tau |  |  |
| --- | --- | --- | --- | --- | --- | --- | --- | --- | --- | --- |
|  | Coef. | Std. Err. | Coef. | Std. Err. | Coef. | Std. Err. | P>z |  | Treatments used |  |
| A B | . | . | . | . | . | . | . | . | A: | Placebo_or_Control |
| A C | . | . | . | . | . | . | . | . | B: | Liraglutide |
| A D | . | . | . | . | . | . | . | . | C: | Albiglutide |
| A E | -1.790659 | 1.079996 | -0.0037651 | 1.798542 | -1.786894 | 2.097891 | 0.394 | 3.88E-12 | D: | Canagliflozin |
| A F | -0.0001898 | 0.7556093 | -1.787084 | 1.95709 | 1.786894 | 2.097891 | 0.394 | 1.99E-13 | E: | Sitagliptin |
| A G | . | . | . | . | . | . | . | . | F: | Semaglutide |
| A H | . | . | . | . | . | . | . | . | G: | Tirzepatide |
| A I | . | . | . | . | . | . | . | . | H: | Empagliflozin |
| A J | . | . | . | . | . | . | . | . | I: | Ertugliflozin |
| A K | . | . | . | . | . | . | . | . | J: | Saxagliptin |
| A L | . | . | . | . | . | . | . | . | K: | Dapagliflozin |
| A M | . | . | . | . | . | . | . | . | L: | Dulaglutide |
| A N | . | . | . | . | . | . | . | . | M: | Linagliptin |
| A O | . | . | . | . | . | . | . | . | N: | Sotagliflozin |
| A P | . | . | . | . | . | . | . | . | O: | Exenatide |
| A Q | . | . | . | . | . | . | . | . | P: | Alogliptin |
| A R | . | . | . | . | . | . | . | . | Q: | Omarigliptin |
| E F | 0.0035753 | 1.632118 | 1.790469 | 1.318081 | -1.786894 | 2.097891 | 0.394 | 5.22E-12 | R: | Lixisenatide |

**eTable 9M Design-by-treatment model and loop inconsistency for all primary outcomes**

| Inconsistency model | chi^2^ | *p* value of Prob>chi^2^ |
| --- | --- | --- |
| Primary outcome: overall hematologic malignancy risk | | |
| design-by-treatment | 2.09 | 0.9549 |
| loop inconsistency | 1.50 | 0.9126 |
| Primary outcome: leukemia risk | | |
| design-by-treatment | 1.12 | 0.7724 |
| loop inconsistency | 0.57 | 0.4502 |
| Primary outcome: acute lymphocytic leukemia risk | | |
| design-by-treatment | 0.45 | 0.5014 |
| loop inconsistency | 0.45 | 0.5014 |
| Primary outcome: acute myeloid leukemia risk | | |
| design-by-treatment | 0.45 | 0.5003 |
| loop inconsistency | 0.45 | 0.5003 |
| Primary outcome: chronic lymphocytic leukemia risk | | |
| design-by-treatment | 0.29 | 0.8663 |
| loop inconsistency | 0.00 | 0.9998 |
| Primary outcome: chronic myeloid leukemia risk | | |
| design-by-treatment | 0.00 | 0.9979 |
| loop inconsistency | 0.00 | 0.9979 |
| Primary outcome: lymphoma risk | | |
| design-by-treatment | 0.72 | 0.8691 |
| loop inconsistency | 0.72 | 0.8691 |
| Primary outcome: non-Hodgkin's lymphoma risk | | |
| design-by-treatment | 0.68 | 0.8775 |
| loop inconsistency | 0.68 | 0.8775 |
| Primary outcome: B cell non-Hodgkin's lymphoma risk | | |
| design-by-treatment | 0.26 | 0.8786 |
| loop inconsistency | 0.26 | 0.8786 |
| Primary outcome: T cell non-Hodgkin's lymphoma risk | | |
| design-by-treatment | 0.05 | 0.8204 |
| loop inconsistency | 0.05 | 0.8204 |
| Primary outcome: myeloma risk | | |
| design-by-treatment | 0.82 | 0.6620 |
| loop inconsistency | 0.82 | 0.6620 |
| Primary outcome: plasma cell myeloma risk | | |
| design-by-treatment | 0.73 | 0.3943 |
| loop inconsistency | 0.73 | 0.3943 |

*Abbreviation: 95%CIs: 95% confidence intervals; DPP4 inhibitor: dipeptidyl-peptidase 4 inhibitor; GLP-1 agonist: glucagon-like peptide-1 agonist; NMA: network meta-analysis; RR: risk ratio; RCT: randomized controlled trial; SGLT2 inhibitor: sodium–glucose cotransporter 2 inhibitor*

**eTable 10 GRADE for primary outcome: overall hematologic malignancy risk**

|  | Comparison | Study limitations | Imprecision | Inconsistency | Indirectness | Publication bias | GRADE |
| --- | --- | --- | --- | --- | --- | --- | --- |
| 1 | Albiglutide:Alogliptin | No downgrade | No downgrade | No downgrade | No downgrade | No downgrade | MODERATE |
| 2 | Albiglutide:Bexagliflozin | No downgrade | No downgrade | No downgrade | No downgrade | No downgrade | MODERATE |
| 3 | Albiglutide:Canagliflozin | No downgrade | No downgrade | No downgrade | No downgrade | No downgrade | MODERATE |
| 4 | Albiglutide:Dapagliflozin | No downgrade | No downgrade | No downgrade | No downgrade | No downgrade | MODERATE |
| 5 | Albiglutide:Dulaglutide | No downgrade | No downgrade | No downgrade | No downgrade | No downgrade | MODERATE |
| 6 | Albiglutide:Efpeglenatide | No downgrade | No downgrade | No downgrade | No downgrade | No downgrade | MODERATE |
| 7 | Albiglutide:Empagliflozin | No downgrade | No downgrade | No downgrade | No downgrade | No downgrade | MODERATE |
| 8 | Albiglutide:Ertugliflozin | No downgrade | No downgrade | No downgrade | No downgrade | No downgrade | MODERATE |
| 9 | Albiglutide:Ertugliflozin_Sitagliptin | No downgrade | No downgrade | No downgrade | No downgrade | No downgrade | MODERATE |
| 10 | Albiglutide:Exenatide | No downgrade | No downgrade | No downgrade | No downgrade | No downgrade | MODERATE |
| 11 | Albiglutide:Linagliptin | No downgrade | No downgrade | No downgrade | No downgrade | No downgrade | MODERATE |
| 12 | Albiglutide:Liraglutide | No downgrade | No downgrade | No downgrade | No downgrade | No downgrade | MODERATE |
| 13 | Albiglutide:Lixisenatide | No downgrade | No downgrade | No downgrade | No downgrade | No downgrade | MODERATE |
| 14 | Albiglutide:Omarigliptin | No downgrade | No downgrade | No downgrade | No downgrade | No downgrade | MODERATE |
| 15 | Albiglutide:Placebo_or_Control | No downgrade | No downgrade | No downgrade | No downgrade | No downgrade | MODERATE |
| 16 | Albiglutide:Saxagliptin | No downgrade | No downgrade | No downgrade | No downgrade | No downgrade | MODERATE |
| 17 | Albiglutide:Semaglutide | No downgrade | No downgrade | No downgrade | No downgrade | No downgrade | MODERATE |
| 18 | Albiglutide:Sitagliptin | No downgrade | No downgrade | No downgrade | No downgrade | No downgrade | MODERATE |
| 19 | Albiglutide:Sotagliflozin | No downgrade | No downgrade | No downgrade | No downgrade | No downgrade | MODERATE |
| 20 | Albiglutide:Tirzepatide | No downgrade | Upgrade due to large effect size | No downgrade | No downgrade | No downgrade | HIGH |
| 21 | Albiglutide:Vildagliptin | No downgrade | Downgrade because the opposite limit exceed | No downgrade | No downgrade | No downgrade | LOW |
| 22 | Alogliptin:Bexagliflozin | No downgrade | No downgrade | No downgrade | No downgrade | No downgrade | MODERATE |
| 23 | Alogliptin:Canagliflozin | No downgrade | No downgrade | No downgrade | No downgrade | No downgrade | MODERATE |
| 24 | Alogliptin:Dapagliflozin | No downgrade | No downgrade | No downgrade | No downgrade | No downgrade | MODERATE |
| 25 | Alogliptin:Dulaglutide | No downgrade | No downgrade | No downgrade | No downgrade | No downgrade | MODERATE |
| 26 | Alogliptin:Efpeglenatide | No downgrade | No downgrade | No downgrade | No downgrade | No downgrade | MODERATE |
| 27 | Alogliptin:Empagliflozin | No downgrade | No downgrade | No downgrade | No downgrade | No downgrade | MODERATE |
| 28 | Alogliptin:Ertugliflozin | No downgrade | No downgrade | No downgrade | No downgrade | No downgrade | MODERATE |
| 29 | Alogliptin:Ertugliflozin_Sitagliptin | No downgrade | No downgrade | No downgrade | No downgrade | No downgrade | MODERATE |
| 30 | Alogliptin:Exenatide | No downgrade | No downgrade | No downgrade | No downgrade | No downgrade | MODERATE |
| 31 | Alogliptin:Linagliptin | No downgrade | No downgrade | No downgrade | No downgrade | No downgrade | MODERATE |
| 32 | Alogliptin:Liraglutide | No downgrade | No downgrade | No downgrade | No downgrade | No downgrade | MODERATE |
| 33 | Alogliptin:Lixisenatide | No downgrade | No downgrade | No downgrade | No downgrade | No downgrade | MODERATE |
| 34 | Alogliptin:Omarigliptin | No downgrade | No downgrade | No downgrade | No downgrade | No downgrade | MODERATE |
| 35 | Alogliptin:Placebo_or_Control | Downgrade because risk of bias | No downgrade | No downgrade | No downgrade | No downgrade | LOW |
| 36 | Alogliptin:Saxagliptin | No downgrade | No downgrade | No downgrade | No downgrade | No downgrade | MODERATE |
| 37 | Alogliptin:Semaglutide | No downgrade | No downgrade | No downgrade | No downgrade | No downgrade | MODERATE |
| 38 | Alogliptin:Sitagliptin | No downgrade | No downgrade | No downgrade | No downgrade | No downgrade | MODERATE |
| 39 | Alogliptin:Sotagliflozin | No downgrade | No downgrade | No downgrade | No downgrade | No downgrade | MODERATE |
| 40 | Alogliptin:Tirzepatide | No downgrade | Upgrade due to large effect size | No downgrade | No downgrade | No downgrade | HIGH |
| 41 | Alogliptin:Vildagliptin | No downgrade | No downgrade | No downgrade | No downgrade | No downgrade | MODERATE |
| 42 | Bexagliflozin:Canagliflozin | No downgrade | Downgrade because the opposite limit exceed | No downgrade | No downgrade | No downgrade | LOW |
| 43 | Bexagliflozin:Dapagliflozin | No downgrade | Downgrade because the opposite limit exceed | No downgrade | No downgrade | No downgrade | LOW |
| 44 | Bexagliflozin:Dulaglutide | No downgrade | Downgrade because the opposite limit exceed | No downgrade | No downgrade | No downgrade | LOW |
| 45 | Bexagliflozin:Efpeglenatide | No downgrade | Downgrade because the opposite limit exceed | No downgrade | No downgrade | No downgrade | LOW |
| 46 | Bexagliflozin:Empagliflozin | No downgrade | Downgrade because the opposite limit exceed | No downgrade | No downgrade | No downgrade | LOW |
| 47 | Bexagliflozin:Ertugliflozin | No downgrade | Downgrade because the opposite limit exceed | No downgrade | No downgrade | No downgrade | LOW |
| 48 | Bexagliflozin:Ertugliflozin_Sitagliptin | No downgrade | Downgrade because the opposite limit exceed | No downgrade | No downgrade | No downgrade | LOW |
| 49 | Bexagliflozin:Exenatide | No downgrade | Downgrade because the opposite limit exceed | No downgrade | No downgrade | No downgrade | LOW |
| 50 | Bexagliflozin:Linagliptin | No downgrade | Downgrade because the opposite limit exceed | No downgrade | No downgrade | No downgrade | LOW |
| 51 | Bexagliflozin:Liraglutide | No downgrade | Downgrade because the opposite limit exceed | No downgrade | No downgrade | No downgrade | LOW |
| 52 | Bexagliflozin:Lixisenatide | No downgrade | Downgrade because the opposite limit exceed | No downgrade | No downgrade | No downgrade | LOW |
| 53 | Bexagliflozin:Omarigliptin | No downgrade | Downgrade because the opposite limit exceed | No downgrade | No downgrade | No downgrade | LOW |
| 54 | Bexagliflozin:Placebo_or_Control | Downgrade because risk of bias | No downgrade | No downgrade | No downgrade | No downgrade | LOW |
| 55 | Bexagliflozin:Saxagliptin | No downgrade | Downgrade because the opposite limit exceed | No downgrade | No downgrade | No downgrade | LOW |
| 56 | Bexagliflozin:Semaglutide | No downgrade | Downgrade because the opposite limit exceed | No downgrade | No downgrade | No downgrade | LOW |
| 57 | Bexagliflozin:Sitagliptin | No downgrade | Downgrade because the opposite limit exceed | No downgrade | No downgrade | No downgrade | LOW |
| 58 | Bexagliflozin:Sotagliflozin | No downgrade | Downgrade because the opposite limit exceed | No downgrade | No downgrade | No downgrade | LOW |
| 59 | Bexagliflozin:Tirzepatide | No downgrade | Downgrade because the opposite limit exceed | No downgrade | No downgrade | No downgrade | LOW |
| 60 | Bexagliflozin:Vildagliptin | No downgrade | Downgrade because the opposite limit exceed | No downgrade | No downgrade | No downgrade | LOW |
| 61 | Canagliflozin:Dapagliflozin | No downgrade | No downgrade | No downgrade | No downgrade | No downgrade | MODERATE |
| 62 | Canagliflozin:Dulaglutide | No downgrade | Upgrade due to large effect size | No downgrade | No downgrade | No downgrade | HIGH |
| 63 | Canagliflozin:Efpeglenatide | No downgrade | No downgrade | No downgrade | No downgrade | No downgrade | MODERATE |
| 64 | Canagliflozin:Empagliflozin | No downgrade | No downgrade | No downgrade | No downgrade | No downgrade | MODERATE |
| 65 | Canagliflozin:Ertugliflozin | No downgrade | No downgrade | No downgrade | No downgrade | No downgrade | MODERATE |
| 66 | Canagliflozin:Ertugliflozin_Sitagliptin | No downgrade | No downgrade | No downgrade | No downgrade | No downgrade | MODERATE |
| 67 | Canagliflozin:Exenatide | No downgrade | No downgrade | No downgrade | No downgrade | No downgrade | MODERATE |
| 68 | Canagliflozin:Linagliptin | No downgrade | No downgrade | No downgrade | No downgrade | No downgrade | MODERATE |
| 69 | Canagliflozin:Liraglutide | No downgrade | No downgrade | No downgrade | No downgrade | No downgrade | MODERATE |
| 70 | Canagliflozin:Lixisenatide | No downgrade | No downgrade | No downgrade | No downgrade | No downgrade | MODERATE |
| 71 | Canagliflozin:Omarigliptin | No downgrade | No downgrade | No downgrade | No downgrade | No downgrade | MODERATE |
| 72 | Canagliflozin:Placebo_or_Control | No downgrade | No downgrade | No downgrade | No downgrade | No downgrade | MODERATE |
| 73 | Canagliflozin:Saxagliptin | No downgrade | No downgrade | No downgrade | No downgrade | No downgrade | MODERATE |
| 74 | Canagliflozin:Semaglutide | No downgrade | No downgrade | No downgrade | No downgrade | No downgrade | MODERATE |
| 75 | Canagliflozin:Sitagliptin | No downgrade | No downgrade | No downgrade | No downgrade | No downgrade | MODERATE |
| 76 | Canagliflozin:Sotagliflozin | No downgrade | No downgrade | No downgrade | No downgrade | No downgrade | MODERATE |
| 77 | Canagliflozin:Tirzepatide | No downgrade | No downgrade | No downgrade | No downgrade | No downgrade | MODERATE |
| 78 | Canagliflozin:Vildagliptin | No downgrade | Downgrade because the opposite limit exceed | No downgrade | No downgrade | No downgrade | LOW |
| 79 | Dapagliflozin:Dulaglutide | No downgrade | No downgrade | No downgrade | No downgrade | No downgrade | MODERATE |
| 80 | Dapagliflozin:Efpeglenatide | No downgrade | No downgrade | No downgrade | No downgrade | No downgrade | MODERATE |
| 81 | Dapagliflozin:Empagliflozin | No downgrade | No downgrade | No downgrade | No downgrade | No downgrade | MODERATE |
| 82 | Dapagliflozin:Ertugliflozin | No downgrade | No downgrade | No downgrade | No downgrade | No downgrade | MODERATE |
| 83 | Dapagliflozin:Ertugliflozin_Sitagliptin | No downgrade | No downgrade | No downgrade | No downgrade | No downgrade | MODERATE |
| 84 | Dapagliflozin:Exenatide | No downgrade | No downgrade | No downgrade | No downgrade | No downgrade | MODERATE |
| 85 | Dapagliflozin:Linagliptin | No downgrade | Upgrade due to large effect size | No downgrade | No downgrade | No downgrade | HIGH |
| 86 | Dapagliflozin:Liraglutide | No downgrade | No downgrade | No downgrade | No downgrade | No downgrade | MODERATE |
| 87 | Dapagliflozin:Lixisenatide | No downgrade | No downgrade | No downgrade | No downgrade | No downgrade | MODERATE |
| 88 | Dapagliflozin:Omarigliptin | No downgrade | No downgrade | No downgrade | No downgrade | No downgrade | MODERATE |
| 89 | Dapagliflozin:Placebo_or_Control | No downgrade | No downgrade | No downgrade | No downgrade | No downgrade | MODERATE |
| 90 | Dapagliflozin:Saxagliptin | No downgrade | No downgrade | No downgrade | No downgrade | No downgrade | MODERATE |
| 91 | Dapagliflozin:Semaglutide | No downgrade | No downgrade | No downgrade | No downgrade | No downgrade | MODERATE |
| 92 | Dapagliflozin:Sitagliptin | No downgrade | No downgrade | No downgrade | No downgrade | No downgrade | MODERATE |
| 93 | Dapagliflozin:Sotagliflozin | No downgrade | No downgrade | No downgrade | No downgrade | No downgrade | MODERATE |
| 94 | Dapagliflozin:Tirzepatide | No downgrade | Upgrade due to large effect size | No downgrade | No downgrade | No downgrade | HIGH |
| 95 | Dapagliflozin:Vildagliptin | No downgrade | Downgrade because the opposite limit exceed | No downgrade | No downgrade | No downgrade | LOW |
| 96 | Dulaglutide:Efpeglenatide | No downgrade | No downgrade | No downgrade | No downgrade | No downgrade | MODERATE |
| 97 | Dulaglutide:Empagliflozin | No downgrade | No downgrade | No downgrade | No downgrade | No downgrade | MODERATE |
| 98 | Dulaglutide:Ertugliflozin | No downgrade | No downgrade | No downgrade | No downgrade | No downgrade | MODERATE |
| 99 | Dulaglutide:Ertugliflozin_Sitagliptin | No downgrade | No downgrade | No downgrade | No downgrade | No downgrade | MODERATE |
| 100 | Dulaglutide:Exenatide | No downgrade | No downgrade | No downgrade | No downgrade | No downgrade | MODERATE |
| 101 | Dulaglutide:Linagliptin | No downgrade | Upgrade due to large effect size | No downgrade | No downgrade | No downgrade | HIGH |
| 102 | Dulaglutide:Liraglutide | No downgrade | Upgrade due to large effect size | No downgrade | No downgrade | No downgrade | HIGH |
| 103 | Dulaglutide:Lixisenatide | No downgrade | No downgrade | No downgrade | No downgrade | No downgrade | MODERATE |
| 104 | Dulaglutide:Omarigliptin | No downgrade | No downgrade | No downgrade | No downgrade | No downgrade | MODERATE |
| 105 | Dulaglutide:Placebo_or_Control | No downgrade | Upgrade due to large effect size | No downgrade | No downgrade | No downgrade | HIGH |
| 106 | Dulaglutide:Saxagliptin | No downgrade | No downgrade | No downgrade | No downgrade | No downgrade | MODERATE |
| 107 | Dulaglutide:Semaglutide | No downgrade | Upgrade due to large effect size | No downgrade | No downgrade | No downgrade | HIGH |
| 108 | Dulaglutide:Sitagliptin | No downgrade | No downgrade | No downgrade | No downgrade | No downgrade | MODERATE |
| 109 | Dulaglutide:Sotagliflozin | No downgrade | No downgrade | No downgrade | No downgrade | No downgrade | MODERATE |
| 110 | Dulaglutide:Tirzepatide | No downgrade | Upgrade due to large effect size | No downgrade | No downgrade | No downgrade | HIGH |
| 111 | Dulaglutide:Vildagliptin | No downgrade | Downgrade because the opposite limit exceed | No downgrade | No downgrade | No downgrade | LOW |
| 112 | Efpeglenatide:Empagliflozin | No downgrade | No downgrade | No downgrade | No downgrade | No downgrade | MODERATE |
| 113 | Efpeglenatide:Ertugliflozin | No downgrade | No downgrade | No downgrade | No downgrade | No downgrade | MODERATE |
| 114 | Efpeglenatide:Ertugliflozin_Sitagliptin | No downgrade | No downgrade | No downgrade | No downgrade | No downgrade | MODERATE |
| 115 | Efpeglenatide:Exenatide | No downgrade | No downgrade | No downgrade | No downgrade | No downgrade | MODERATE |
| 116 | Efpeglenatide:Linagliptin | No downgrade | No downgrade | No downgrade | No downgrade | No downgrade | MODERATE |
| 117 | Efpeglenatide:Liraglutide | No downgrade | No downgrade | No downgrade | No downgrade | No downgrade | MODERATE |
| 118 | Efpeglenatide:Lixisenatide | No downgrade | No downgrade | No downgrade | No downgrade | No downgrade | MODERATE |
| 119 | Efpeglenatide:Omarigliptin | No downgrade | No downgrade | No downgrade | No downgrade | No downgrade | MODERATE |
| 120 | Efpeglenatide:Placebo_or_Control | No downgrade | No downgrade | No downgrade | No downgrade | No downgrade | MODERATE |
| 121 | Efpeglenatide:Saxagliptin | No downgrade | No downgrade | No downgrade | No downgrade | No downgrade | MODERATE |
| 122 | Efpeglenatide:Semaglutide | No downgrade | No downgrade | No downgrade | No downgrade | No downgrade | MODERATE |
| 123 | Efpeglenatide:Sitagliptin | No downgrade | No downgrade | No downgrade | No downgrade | No downgrade | MODERATE |
| 124 | Efpeglenatide:Sotagliflozin | No downgrade | No downgrade | No downgrade | No downgrade | No downgrade | MODERATE |
| 125 | Efpeglenatide:Tirzepatide | No downgrade | No downgrade | No downgrade | No downgrade | No downgrade | MODERATE |
| 126 | Efpeglenatide:Vildagliptin | No downgrade | Downgrade because the opposite limit exceed | No downgrade | No downgrade | No downgrade | LOW |
| 127 | Empagliflozin:Ertugliflozin | No downgrade | No downgrade | No downgrade | No downgrade | No downgrade | MODERATE |
| 128 | Empagliflozin:Ertugliflozin_Sitagliptin | No downgrade | No downgrade | No downgrade | No downgrade | No downgrade | MODERATE |
| 129 | Empagliflozin:Exenatide | No downgrade | No downgrade | No downgrade | No downgrade | No downgrade | MODERATE |
| 130 | Empagliflozin:Linagliptin | No downgrade | Upgrade due to large effect size | No downgrade | No downgrade | No downgrade | HIGH |
| 131 | Empagliflozin:Liraglutide | No downgrade | No downgrade | No downgrade | No downgrade | No downgrade | MODERATE |
| 132 | Empagliflozin:Lixisenatide | No downgrade | No downgrade | No downgrade | No downgrade | No downgrade | MODERATE |
| 133 | Empagliflozin:Omarigliptin | No downgrade | No downgrade | No downgrade | No downgrade | No downgrade | MODERATE |
| 134 | Empagliflozin:Placebo_or_Control | No downgrade | No downgrade | No downgrade | No downgrade | No downgrade | MODERATE |
| 135 | Empagliflozin:Saxagliptin | No downgrade | No downgrade | No downgrade | No downgrade | No downgrade | MODERATE |
| 136 | Empagliflozin:Semaglutide | No downgrade | No downgrade | No downgrade | No downgrade | No downgrade | MODERATE |
| 137 | Empagliflozin:Sitagliptin | No downgrade | No downgrade | No downgrade | No downgrade | No downgrade | MODERATE |
| 138 | Empagliflozin:Sotagliflozin | No downgrade | No downgrade | No downgrade | No downgrade | No downgrade | MODERATE |
| 139 | Empagliflozin:Tirzepatide | No downgrade | Upgrade due to large effect size | No downgrade | No downgrade | No downgrade | HIGH |
| 140 | Empagliflozin:Vildagliptin | No downgrade | Downgrade because the opposite limit exceed | No downgrade | No downgrade | No downgrade | LOW |
| 141 | Ertugliflozin:Ertugliflozin_Sitagliptin | No downgrade | No downgrade | No downgrade | No downgrade | No downgrade | MODERATE |
| 142 | Ertugliflozin:Exenatide | No downgrade | No downgrade | No downgrade | No downgrade | No downgrade | MODERATE |
| 143 | Ertugliflozin:Linagliptin | No downgrade | No downgrade | No downgrade | No downgrade | No downgrade | MODERATE |
| 144 | Ertugliflozin:Liraglutide | No downgrade | No downgrade | No downgrade | No downgrade | No downgrade | MODERATE |
| 145 | Ertugliflozin:Lixisenatide | No downgrade | No downgrade | No downgrade | No downgrade | No downgrade | MODERATE |
| 146 | Ertugliflozin:Omarigliptin | No downgrade | No downgrade | No downgrade | No downgrade | No downgrade | MODERATE |
| 147 | Ertugliflozin:Placebo_or_Control | No downgrade | No downgrade | No downgrade | No downgrade | No downgrade | MODERATE |
| 148 | Ertugliflozin:Saxagliptin | No downgrade | No downgrade | No downgrade | No downgrade | No downgrade | MODERATE |
| 149 | Ertugliflozin:Semaglutide | No downgrade | No downgrade | No downgrade | No downgrade | No downgrade | MODERATE |
| 150 | Ertugliflozin:Sitagliptin | No downgrade | No downgrade | No downgrade | No downgrade | No downgrade | MODERATE |
| 151 | Ertugliflozin:Sotagliflozin | No downgrade | No downgrade | No downgrade | No downgrade | No downgrade | MODERATE |
| 152 | Ertugliflozin:Tirzepatide | No downgrade | Upgrade due to large effect size | No downgrade | No downgrade | No downgrade | HIGH |
| 153 | Ertugliflozin:Vildagliptin | No downgrade | Downgrade because the opposite limit exceed | No downgrade | No downgrade | No downgrade | LOW |
| 154 | Ertugliflozin_Sitagliptin:Exenatide | No downgrade | No downgrade | No downgrade | No downgrade | No downgrade | MODERATE |
| 155 | Ertugliflozin_Sitagliptin:Linagliptin | No downgrade | No downgrade | No downgrade | No downgrade | No downgrade | MODERATE |
| 156 | Ertugliflozin_Sitagliptin:Liraglutide | No downgrade | No downgrade | No downgrade | No downgrade | No downgrade | MODERATE |
| 157 | Ertugliflozin_Sitagliptin:Lixisenatide | No downgrade | No downgrade | No downgrade | No downgrade | No downgrade | MODERATE |
| 158 | Ertugliflozin_Sitagliptin:Omarigliptin | No downgrade | No downgrade | No downgrade | No downgrade | No downgrade | MODERATE |
| 159 | Ertugliflozin_Sitagliptin:Placebo_or_Control | No downgrade | No downgrade | No downgrade | No downgrade | No downgrade | MODERATE |
| 160 | Ertugliflozin_Sitagliptin:Saxagliptin | No downgrade | No downgrade | No downgrade | No downgrade | No downgrade | MODERATE |
| 161 | Ertugliflozin_Sitagliptin:Semaglutide | No downgrade | No downgrade | No downgrade | No downgrade | No downgrade | MODERATE |
| 162 | Ertugliflozin_Sitagliptin:Sitagliptin | No downgrade | No downgrade | No downgrade | No downgrade | No downgrade | MODERATE |
| 163 | Ertugliflozin_Sitagliptin:Sotagliflozin | No downgrade | No downgrade | No downgrade | No downgrade | No downgrade | MODERATE |
| 164 | Ertugliflozin_Sitagliptin:Tirzepatide | No downgrade | No downgrade | No downgrade | No downgrade | No downgrade | MODERATE |
| 165 | Ertugliflozin_Sitagliptin:Vildagliptin | No downgrade | Downgrade because the opposite limit exceed | No downgrade | No downgrade | No downgrade | LOW |
| 166 | Exenatide:Linagliptin | No downgrade | No downgrade | No downgrade | No downgrade | No downgrade | MODERATE |
| 167 | Exenatide:Liraglutide | Downgrade because risk of bias | No downgrade | No downgrade | No downgrade | No downgrade | LOW |
| 168 | Exenatide:Lixisenatide | No downgrade | No downgrade | No downgrade | No downgrade | No downgrade | MODERATE |
| 169 | Exenatide:Omarigliptin | No downgrade | No downgrade | No downgrade | No downgrade | No downgrade | MODERATE |
| 170 | Exenatide:Placebo_or_Control | No downgrade | No downgrade | No downgrade | No downgrade | No downgrade | MODERATE |
| 171 | Exenatide:Saxagliptin | No downgrade | No downgrade | No downgrade | No downgrade | No downgrade | MODERATE |
| 172 | Exenatide:Semaglutide | No downgrade | No downgrade | No downgrade | No downgrade | No downgrade | MODERATE |
| 173 | Exenatide:Sitagliptin | No downgrade | No downgrade | No downgrade | No downgrade | No downgrade | MODERATE |
| 174 | Exenatide:Sotagliflozin | No downgrade | No downgrade | No downgrade | No downgrade | No downgrade | MODERATE |
| 175 | Exenatide:Tirzepatide | No downgrade | Upgrade due to large effect size | No downgrade | No downgrade | No downgrade | HIGH |
| 176 | Exenatide:Vildagliptin | No downgrade | Downgrade because the opposite limit exceed | No downgrade | No downgrade | No downgrade | LOW |
| 177 | Linagliptin:Liraglutide | No downgrade | No downgrade | No downgrade | No downgrade | No downgrade | MODERATE |
| 178 | Linagliptin:Lixisenatide | No downgrade | No downgrade | No downgrade | No downgrade | No downgrade | MODERATE |
| 179 | Linagliptin:Omarigliptin | No downgrade | No downgrade | No downgrade | No downgrade | No downgrade | MODERATE |
| 180 | Linagliptin:Placebo_or_Control | No downgrade | No downgrade | No downgrade | No downgrade | No downgrade | MODERATE |
| 181 | Linagliptin:Saxagliptin | No downgrade | No downgrade | No downgrade | No downgrade | No downgrade | MODERATE |
| 182 | Linagliptin:Semaglutide | No downgrade | No downgrade | No downgrade | No downgrade | No downgrade | MODERATE |
| 183 | Linagliptin:Sitagliptin | No downgrade | No downgrade | No downgrade | No downgrade | No downgrade | MODERATE |
| 184 | Linagliptin:Sotagliflozin | No downgrade | No downgrade | No downgrade | No downgrade | No downgrade | MODERATE |
| 185 | Linagliptin:Tirzepatide | No downgrade | No downgrade | No downgrade | No downgrade | No downgrade | MODERATE |
| 186 | Linagliptin:Vildagliptin | No downgrade | Downgrade because the opposite limit exceed | No downgrade | No downgrade | No downgrade | LOW |
| 187 | Liraglutide:Lixisenatide | No downgrade | No downgrade | No downgrade | No downgrade | No downgrade | MODERATE |
| 188 | Liraglutide:Omarigliptin | No downgrade | No downgrade | No downgrade | No downgrade | No downgrade | MODERATE |
| 189 | Liraglutide:Placebo_or_Control | No downgrade | Upgrade due to large effect size | No downgrade | No downgrade | No downgrade | HIGH |
| 190 | Liraglutide:Saxagliptin | No downgrade | No downgrade | No downgrade | No downgrade | No downgrade | MODERATE |
| 191 | Liraglutide:Semaglutide | No downgrade | No downgrade | No downgrade | No downgrade | No downgrade | MODERATE |
| 192 | Liraglutide:Sitagliptin | No downgrade | No downgrade | No downgrade | No downgrade | No downgrade | MODERATE |
| 193 | Liraglutide:Sotagliflozin | No downgrade | No downgrade | No downgrade | No downgrade | No downgrade | MODERATE |
| 194 | Liraglutide:Tirzepatide | No downgrade | No downgrade | No downgrade | No downgrade | No downgrade | MODERATE |
| 195 | Liraglutide:Vildagliptin | No downgrade | Downgrade because the opposite limit exceed | No downgrade | No downgrade | No downgrade | LOW |
| 196 | Lixisenatide:Omarigliptin | No downgrade | No downgrade | No downgrade | No downgrade | No downgrade | MODERATE |
| 197 | Lixisenatide:Placebo_or_Control | No downgrade | No downgrade | No downgrade | No downgrade | No downgrade | MODERATE |
| 198 | Lixisenatide:Saxagliptin | No downgrade | No downgrade | No downgrade | No downgrade | No downgrade | MODERATE |
| 199 | Lixisenatide:Semaglutide | No downgrade | No downgrade | No downgrade | No downgrade | No downgrade | MODERATE |
| 200 | Lixisenatide:Sitagliptin | No downgrade | No downgrade | No downgrade | No downgrade | No downgrade | MODERATE |
| 201 | Lixisenatide:Sotagliflozin | No downgrade | No downgrade | No downgrade | No downgrade | No downgrade | MODERATE |
| 202 | Lixisenatide:Tirzepatide | No downgrade | No downgrade | No downgrade | No downgrade | No downgrade | MODERATE |
| 203 | Lixisenatide:Vildagliptin | No downgrade | Downgrade because the opposite limit exceed | No downgrade | No downgrade | No downgrade | LOW |
| 204 | Omarigliptin:Placebo_or_Control | No downgrade | No downgrade | No downgrade | No downgrade | No downgrade | MODERATE |
| 205 | Omarigliptin:Saxagliptin | No downgrade | No downgrade | No downgrade | No downgrade | No downgrade | MODERATE |
| 206 | Omarigliptin:Semaglutide | No downgrade | No downgrade | No downgrade | No downgrade | No downgrade | MODERATE |
| 207 | Omarigliptin:Sitagliptin | No downgrade | No downgrade | No downgrade | No downgrade | No downgrade | MODERATE |
| 208 | Omarigliptin:Sotagliflozin | No downgrade | No downgrade | No downgrade | No downgrade | No downgrade | MODERATE |
| 209 | Omarigliptin:Tirzepatide | No downgrade | No downgrade | No downgrade | No downgrade | No downgrade | MODERATE |
| 210 | Omarigliptin:Vildagliptin | No downgrade | Downgrade because the opposite limit exceed | No downgrade | No downgrade | No downgrade | LOW |
| 211 | Saxagliptin:Placebo_or_Control | No downgrade | No downgrade | No downgrade | No downgrade | No downgrade | MODERATE |
| 212 | Semaglutide:Placebo_or_Control | No downgrade | No downgrade | No downgrade | No downgrade | No downgrade | MODERATE |
| 213 | Sitagliptin:Placebo_or_Control | No downgrade | No downgrade | No downgrade | No downgrade | No downgrade | MODERATE |
| 214 | Sotagliflozin:Placebo_or_Control | Downgrade because risk of bias | No downgrade | No downgrade | No downgrade | No downgrade | LOW |
| 215 | Tirzepatide:Placebo_or_Control | No downgrade | Upgrade due to large effect size | No downgrade | No downgrade | No downgrade | HIGH |
| 216 | Vildagliptin:Placebo_or_Control | No downgrade | Downgrade because the opposite limit exceed | No downgrade | No downgrade | No downgrade | LOW |
| 217 | Saxagliptin:Semaglutide | No downgrade | No downgrade | No downgrade | No downgrade | No downgrade | MODERATE |
| 218 | Saxagliptin:Sitagliptin | No downgrade | No downgrade | No downgrade | No downgrade | No downgrade | MODERATE |
| 219 | Saxagliptin:Sotagliflozin | No downgrade | No downgrade | No downgrade | No downgrade | No downgrade | MODERATE |
| 220 | Saxagliptin:Tirzepatide | No downgrade | Upgrade due to large effect size | No downgrade | No downgrade | No downgrade | HIGH |
| 221 | Saxagliptin:Vildagliptin | No downgrade | Downgrade because the opposite limit exceed | No downgrade | No downgrade | No downgrade | LOW |
| 222 | Semaglutide:Sitagliptin | No downgrade | No downgrade | No downgrade | No downgrade | No downgrade | MODERATE |
| 223 | Semaglutide:Sotagliflozin | No downgrade | No downgrade | No downgrade | No downgrade | No downgrade | MODERATE |
| 224 | Semaglutide:Tirzepatide | No downgrade | No downgrade | No downgrade | No downgrade | No downgrade | MODERATE |
| 225 | Semaglutide:Vildagliptin | No downgrade | Downgrade because the opposite limit exceed | No downgrade | No downgrade | No downgrade | LOW |
| 226 | Sitagliptin:Sotagliflozin | No downgrade | No downgrade | No downgrade | No downgrade | No downgrade | MODERATE |
| 227 | Sitagliptin:Tirzepatide | No downgrade | Upgrade due to large effect size | No downgrade | No downgrade | No downgrade | HIGH |
| 228 | Sitagliptin:Vildagliptin | No downgrade | Downgrade because the opposite limit exceed | No downgrade | No downgrade | No downgrade | LOW |
| 229 | Sotagliflozin:Tirzepatide | No downgrade | Upgrade due to large effect size | No downgrade | No downgrade | No downgrade | HIGH |
| 230 | Sotagliflozin:Vildagliptin | No downgrade | Downgrade because the opposite limit exceed | No downgrade | No downgrade | No downgrade | LOW |
| 231 | Tirzepatide:Vildagliptin | No downgrade | Downgrade because the opposite limit exceed | No downgrade | No downgrade | No downgrade | LOW |

*Abbreviation: 95%CIs: 95% confidence intervals; DPP4 inhibitor: dipeptidyl-peptidase 4 inhibitor; GLP-1 agonist: glucagon-like peptide-1 agonist; NMA: network meta-analysis; RR: risk ratio; RCT: randomized controlled trial; SGLT2 inhibitor: sodium–glucose cotransporter 2 inhibitor*

**Reference list of supplement tables:**

1. Peryer G, Golder S, Junqueira D, Vohra S, Loke YK, Group CAEM. Chapter 19: adverse effects. In: Higgins JPT, Thomas J, Chandler J, Cumpston M, Li T, Page MJ, et al., editors. Cochrane Handbook for Systematic Reviews of Interventions, Cochrane: Cochrane; 2023.

2. Hutton B, Salanti G, Caldwell DM, Chaimani A, Schmid CH, Cameron C, et al. The PRISMA extension statement for reporting of systematic reviews incorporating network meta-analyses of health care interventions: checklist and explanations. Ann Intern Med. 2015;162(11):777-84.

3. Phillips R, Hazell L, Sauzet O, Cornelius V. Analysis and reporting of adverse events in randomised controlled trials: a review. BMJ Open. 2019;9(2):e024537.

4. Sterne JAC, Savovic J, Page MJ, Elbers RG, Blencowe NS, Boutron I, et al. RoB 2: a revised tool for assessing risk of bias in randomised trials. Bmj. 2019;366:l4898.

5. Keykhaei M, Masinaei M, Mohammadi E, Azadnajafabad S, Rezaei N, Saeedi Moghaddam S, et al. A global, regional, and national survey on burden and Quality of Care Index (QCI) of hematologic malignancies; global burden of disease systematic analysis 1990-2017. Exp Hematol Oncol. 2021;10(1):11.

6. Alaggio R, Amador C, Anagnostopoulos I, Attygalle AD, Araujo IBO, Berti E, et al. The 5th edition of the World Health Organization Classification of Haematolymphoid Tumours: Lymphoid Neoplasms. Leukemia. 2022;36(7):1720-48.

7. Tseng PT, Zeng BS, Hung CM, Liang CS, Stubbs B, Carvalho AF, et al. Assessment of Noninvasive Brain Stimulation Interventions for Negative Symptoms of Schizophrenia: A Systematic Review and Network Meta-analysis. JAMA psychiatry. 2022;79(8):770-9.

8. Tseng PT, Yang CP, Su KP, Chen TY, Wu YC, Tu YK, et al. The association between melatonin and episodic migraine: A pilot network meta-analysis of randomized controlled trials to compare the prophylactic effects with exogenous melatonin supplementation and pharmacotherapy. Journal of pineal research. 2020;69(2):e12663.

9. Anker SD, Butler J, Filippatos G, Ferreira JP, Bocchi E, Bohm M, et al. Empagliflozin in Heart Failure with a Preserved Ejection Fraction. N Engl J Med. 2021;385(16):1451-61.

10. Arjona Ferreira JC, Marre M, Barzilai N, Guo H, Golm GT, Sisk CM, et al. Efficacy and safety of sitagliptin versus glipizide in patients with type 2 diabetes and moderate-to-severe chronic renal insufficiency. Diabetes Care. 2013;36(5):1067-73.

11. Aronne LJ, Sattar N, Horn DB, Bays HE, Wharton S, Lin WY, et al. Continued Treatment With Tirzepatide for Maintenance of Weight Reduction in Adults With Obesity: The SURMOUNT-4 Randomized Clinical Trial. Jama. 2024;331(1):38-48.

12. Bailey CJ, Gross JL, Pieters A, Bastien A, List JF. Effect of dapagliflozin in patients with type 2 diabetes who have inadequate glycaemic control with metformin: a randomised, double-blind, placebo-controlled trial. Lancet. 2010;375(9733):2223-33.

13. Bhatt DL, Szarek M, Pitt B, Cannon CP, Leiter LA, McGuire DK, et al. Sotagliflozin in Patients with Diabetes and Chronic Kidney Disease. N Engl J Med. 2021;384(2):129-39.

14. Buse JB, Rosenstock J, Sesti G, Schmidt WE, Montanya E, Brett JH, et al. Liraglutide once a day versus exenatide twice a day for type 2 diabetes: a 26-week randomised, parallel-group, multinational, open-label trial (LEAD-6). Lancet. 2009;374(9683):39-47.

15. Cannon CP, Pratley R, Dagogo-Jack S, Mancuso J, Huyck S, Masiukiewicz U, et al. Cardiovascular Outcomes with Ertugliflozin in Type 2 Diabetes. N Engl J Med. 2020;383(15):1425-35.

16. Charbonnel B, Karasik A, Liu J, Wu M, Meininger G, Sitagliptin Study G. Efficacy and safety of the dipeptidyl peptidase-4 inhibitor sitagliptin added to ongoing metformin therapy in patients with type 2 diabetes inadequately controlled with metformin alone. Diabetes Care. 2006;29(12):2638-43.

17. Cherney DZI, Ferrannini E, Umpierrez GE, Peters AL, Rosenstock J, Powell DR, et al. Efficacy and safety of sotagliflozin in patients with type 2 diabetes and stage 3 chronic kidney disease. Diabetes Obes Metab. 2023;25(6):1646-57.

18. Danne T, Cariou B, Banks P, Brandle M, Brath H, Franek E, et al. HbA(1c) and Hypoglycemia Reductions at 24 and 52 Weeks With Sotagliflozin in Combination With Insulin in Adults With Type 1 Diabetes: The European inTandem2 Study. Diabetes Care. 2018;41(9):1981-90.

19. Davies M, Faerch L, Jeppesen OK, Pakseresht A, Pedersen SD, Perreault L, et al. Semaglutide 2.4 mg once a week in adults with overweight or obesity, and type 2 diabetes (STEP 2): a randomised, double-blind, double-dummy, placebo-controlled, phase 3 trial. Lancet. 2021;397(10278):971-84.

20. DeFronzo RA, Hissa MN, Garber AJ, Luiz Gross J, Yuyan Duan R, Ravichandran S, et al. The efficacy and safety of saxagliptin when added to metformin therapy in patients with inadequately controlled type 2 diabetes with metformin alone. Diabetes Care. 2009;32(9):1649-55.

21. Del Prato S, Fleck P, Wilson C, Chaudhari P. Comparison of alogliptin and glipizide for composite endpoint of glycated haemoglobin reduction, no hypoglycaemia and no weight gain in type 2 diabetes mellitus. Diabetes Obes Metab. 2016;18(6):623-7.

22. Del Prato S, Kahn SE, Pavo I, Weerakkody GJ, Yang Z, Doupis J, et al. Tirzepatide versus insulin glargine in type 2 diabetes and increased cardiovascular risk (SURPASS-4): a randomised, open-label, parallel-group, multicentre, phase 3 trial. Lancet. 2021;398(10313):1811-24.

23. Dou J, Ma J, Liu J, Wang C, Johnsson E, Yao H, et al. Efficacy and safety of saxagliptin in combination with metformin as initial therapy in Chinese patients with type 2 diabetes: Results from the START study, a multicentre, randomized, double-blind, active-controlled, phase 3 trial. Diabetes Obes Metab. 2018;20(3):590-8.

24. Frias JP, Auerbach P, Bajaj HS, Fukushima Y, Lingvay I, Macura S, et al. Efficacy and safety of once-weekly semaglutide 2.0 mg versus 1.0 mg in patients with type 2 diabetes (SUSTAIN FORTE): a double-blind, randomised, phase 3B trial. Lancet Diabetes Endocrinol. 2021;9(9):563-74.

25. Frias JP, Davies MJ, Rosenstock J, Perez Manghi FC, Fernandez Lando L, Bergman BK, et al. Tirzepatide versus Semaglutide Once Weekly in Patients with Type 2 Diabetes. N Engl J Med. 2021;385(6):503-15.

26. Gallo S, Charbonnel B, Goldman A, Shi H, Huyck S, Darekar A, et al. Long-term efficacy and safety of ertugliflozin in patients with type 2 diabetes mellitus inadequately controlled with metformin monotherapy: 104-week VERTIS MET trial. Diabetes Obes Metab. 2019;21(4):1027-36.

27. Gantz I, Chen M, Suryawanshi S, Ntabadde C, Shah S, O'Neill EA, et al. A randomized, placebo-controlled study of the cardiovascular safety of the once-weekly DPP-4 inhibitor omarigliptin in patients with type 2 diabetes mellitus. Cardiovasc Diabetol. 2017;16(1):112.

28. Garvey WT, Frias JP, Jastreboff AM, le Roux CW, Sattar N, Aizenberg D, et al. Tirzepatide once weekly for the treatment of obesity in people with type 2 diabetes (SURMOUNT-2): a double-blind, randomised, multicentre, placebo-controlled, phase 3 trial. Lancet. 2023;402(10402):613-26.

29. Gerstein HC, Colhoun HM, Dagenais GR, Diaz R, Lakshmanan M, Pais P, et al. Dulaglutide and cardiovascular outcomes in type 2 diabetes (REWIND): a double-blind, randomised placebo-controlled trial. Lancet. 2019;394(10193):121-30.

30. Gerstein HC, Sattar N, Rosenstock J, Ramasundarahettige C, Pratley R, Lopes RD, et al. Cardiovascular and Renal Outcomes with Efpeglenatide in Type 2 Diabetes. N Engl J Med. 2021;385(10):896-907.

31. Giorgino F, Benroubi M, Sun JH, Zimmermann AG, Pechtner V. Efficacy and Safety of Once-Weekly Dulaglutide Versus Insulin Glargine in Patients With Type 2 Diabetes on Metformin and Glimepiride (AWARD-2). Diabetes Care. 2015;38(12):2241-9.

32. Goke B, Gallwitz B, Eriksson JG, Hellqvist A, Gause-Nilsson I. Saxagliptin vs. glipizide as add-on therapy in patients with type 2 diabetes mellitus inadequately controlled on metformin alone: long-term (52-week) extension of a 52-week randomised controlled trial. International journal of clinical practice. 2013;67(4):307-16.

33. Green JB, Bethel MA, Armstrong PW, Buse JB, Engel SS, Garg J, et al. Effect of Sitagliptin on Cardiovascular Outcomes in Type 2 Diabetes. N Engl J Med. 2015;373(3):232-42.

34. Grunberger G, Camp S, Johnson J, Huyck S, Terra SG, Mancuso JP, et al. Ertugliflozin in Patients with Stage 3 Chronic Kidney Disease and Type 2 Diabetes Mellitus: The VERTIS RENAL Randomized Study. Diabetes Ther. 2018;9(1):49-66.

35. Hadjadj S, Rosenstock J, Meinicke T, Woerle HJ, Broedl UC. Initial Combination of Empagliflozin and Metformin in Patients With Type 2 Diabetes. Diabetes Care. 2016;39(10):1718-28.

36. Heerspink HJL, Stefansson BV, Correa-Rotter R, Chertow GM, Greene T, Hou FF, et al. Dapagliflozin in Patients with Chronic Kidney Disease. N Engl J Med. 2020;383(15):1436-46.

37. Hernandez AF, Green JB, Janmohamed S, D'Agostino RB, Sr., Granger CB, Jones NP, et al. Albiglutide and cardiovascular outcomes in patients with type 2 diabetes and cardiovascular disease (Harmony Outcomes): a double-blind, randomised placebo-controlled trial. Lancet. 2018;392(10157):1519-29.

38. Holman RR, Bethel MA, Mentz RJ, Thompson VP, Lokhnygina Y, Buse JB, et al. Effects of Once-Weekly Exenatide on Cardiovascular Outcomes in Type 2 Diabetes. N Engl J Med. 2017;377(13):1228-39.

39. Husain M, Birkenfeld AL, Donsmark M, Dungan K, Eliaschewitz FG, Franco DR, et al. Oral Semaglutide and Cardiovascular Outcomes in Patients with Type 2 Diabetes. N Engl J Med. 2019;381(9):841-51.

40. Jastreboff AM, Aronne LJ, Ahmad NN, Wharton S, Connery L, Alves B, et al. Tirzepatide Once Weekly for the Treatment of Obesity. N Engl J Med. 2022;387(3):205-16.

41. Kaku K, Yamada Y, Watada H, Abiko A, Nishida T, Zacho J, et al. Safety and efficacy of once-weekly semaglutide vs additional oral antidiabetic drugs in Japanese people with inadequately controlled type 2 diabetes: A randomized trial. Diabetes Obes Metab. 2018;20(5):1202-12.

42. Lincoff AM, Brown-Frandsen K, Colhoun HM, Deanfield J, Emerson SS, Esbjerg S, et al. Semaglutide and Cardiovascular Outcomes in Obesity without Diabetes. N Engl J Med. 2023;389(24):2221-32.

43. Lock JP. Bexagliflozin Efficacy and Safety Trial (BEST), <https://clinicaltrials.gov/study/NCT02558296?cond=NCT02558296&rank=1>; 2021 [accessed 2024/10/28 2024].

44. Marso SP, Bain SC, Consoli A, Eliaschewitz FG, Jodar E, Leiter LA, et al. Semaglutide and Cardiovascular Outcomes in Patients with Type 2 Diabetes. N Engl J Med. 2016;375(19):1834-44.

45. Marso SP, Daniels GH, Brown-Frandsen K, Kristensen P, Mann JF, Nauck MA, et al. Liraglutide and Cardiovascular Outcomes in Type 2 Diabetes. N Engl J Med. 2016;375(4):311-22.

46. Matthews DR, Paldanius PM, Proot P, Chiang Y, Stumvoll M, Del Prato S, et al. Glycaemic durability of an early combination therapy with vildagliptin and metformin versus sequential metformin monotherapy in newly diagnosed type 2 diabetes (VERIFY): a 5-year, multicentre, randomised, double-blind trial. Lancet. 2019;394(10208):1519-29.

47. McMurray JJV, Solomon SD, Inzucchi SE, Kober L, Kosiborod MN, Martinez FA, et al. Dapagliflozin in Patients with Heart Failure and Reduced Ejection Fraction. N Engl J Med. 2019;381(21):1995-2008.

48. Nassif ME, Windsor SL, Tang F, Khariton Y, Husain M, Inzucchi SE, et al. Dapagliflozin Effects on Biomarkers, Symptoms, and Functional Status in Patients With Heart Failure With Reduced Ejection Fraction: The DEFINE-HF Trial. Circulation. 2019;140(18):1463-76.

49. Nauck M, Frid A, Hermansen K, Shah NS, Tankova T, Mitha IH, et al. Efficacy and safety comparison of liraglutide, glimepiride, and placebo, all in combination with metformin, in type 2 diabetes: the LEAD (liraglutide effect and action in diabetes)-2 study. Diabetes Care. 2009;32(1):84-90.

50. Nauck M, Rizzo M, Johnson A, Bosch-Traberg H, Madsen J, Cariou B. Once-Daily Liraglutide Versus Lixisenatide as Add-on to Metformin in Type 2 Diabetes: A 26-Week Randomized Controlled Clinical Trial. Diabetes Care. 2016;39(9):1501-9.

51. Nauck MA, Meininger G, Sheng D, Terranella L, Stein PP, Sitagliptin Study G. Efficacy and safety of the dipeptidyl peptidase-4 inhibitor, sitagliptin, compared with the sulfonylurea, glipizide, in patients with type 2 diabetes inadequately controlled on metformin alone: a randomized, double-blind, non-inferiority trial. Diabetes Obes Metab. 2007;9(2):194-205.

52. Nauck MA, Stewart MW, Perkins C, Jones-Leone A, Yang F, Perry C, et al. Efficacy and safety of once-weekly GLP-1 receptor agonist albiglutide (HARMONY 2): 52 week primary endpoint results from a randomised, placebo-controlled trial in patients with type 2 diabetes mellitus inadequately controlled with diet and exercise. Diabetologia. 2016;59(2):266-74.

53. Neal B, Perkovic V, Mahaffey KW, de Zeeuw D, Fulcher G, Erondu N, et al. Canagliflozin and Cardiovascular and Renal Events in Type 2 Diabetes. N Engl J Med. 2017;377(7):644-57.

54. Packer M, Anker SD, Butler J, Filippatos G, Pocock SJ, Carson P, et al. Cardiovascular and Renal Outcomes with Empagliflozin in Heart Failure. N Engl J Med. 2020;383(15):1413-24.

55. Packer M, Zile MR, Kramer CM, Baum SJ, Litwin SE, Menon V, et al. Tirzepatide for Heart Failure with Preserved Ejection Fraction and Obesity. N Engl J Med. 2025;392(5):427-37.

56. Perkovic V, Jardine MJ, Neal B, Bompoint S, Heerspink HJL, Charytan DM, et al. Canagliflozin and Renal Outcomes in Type 2 Diabetes and Nephropathy. N Engl J Med. 2019;380(24):2295-306.

57. Perkovic V, Tuttle KR, Rossing P, Mahaffey KW, Mann JFE, Bakris G, et al. Effects of Semaglutide on Chronic Kidney Disease in Patients with Type 2 Diabetes. N Engl J Med. 2024;391(2):109-21.

58. Pfeffer MA, Claggett B, Diaz R, Dickstein K, Gerstein HC, Kober LV, et al. Lixisenatide in Patients with Type 2 Diabetes and Acute Coronary Syndrome. N Engl J Med. 2015;373(23):2247-57.

59. Pi-Sunyer X, Astrup A, Fujioka K, Greenway F, Halpern A, Krempf M, et al. A Randomized, Controlled Trial of 3.0 mg of Liraglutide in Weight Management. N Engl J Med. 2015;373(1):11-22.

60. Pratley RE, Eldor R, Raji A, Golm G, Huyck SB, Qiu Y, et al. Ertugliflozin plus sitagliptin versus either individual agent over 52 weeks in patients with type 2 diabetes mellitus inadequately controlled with metformin: The VERTIS FACTORIAL randomized trial. Diabetes Obes Metab. 2018;20(5):1111-20.

61. Raz I, Chen Y, Wu M, Hussain S, Kaufman KD, Amatruda JM, et al. Efficacy and safety of sitagliptin added to ongoing metformin therapy in patients with type 2 diabetes. Curr Med Res Opin. 2008;24(2):537-50.

62. Ridderstrale M, Andersen KR, Zeller C, Kim G, Woerle HJ, Broedl UC, et al. Comparison of empagliflozin and glimepiride as add-on to metformin in patients with type 2 diabetes: a 104-week randomised, active-controlled, double-blind, phase 3 trial. Lancet Diabetes Endocrinol. 2014;2(9):691-700.

63. Rosenstock J, Allison D, Birkenfeld AL, Blicher TM, Deenadayalan S, Jacobsen JB, et al. Effect of Additional Oral Semaglutide vs Sitagliptin on Glycated Hemoglobin in Adults With Type 2 Diabetes Uncontrolled With Metformin Alone or With Sulfonylurea: The PIONEER 3 Randomized Clinical Trial. Jama. 2019;321(15):1466-80.

64. Rosenstock J, Fonseca VA, Gross JL, Ratner RE, Ahren B, Chow FC, et al. Advancing basal insulin replacement in type 2 diabetes inadequately controlled with insulin glargine plus oral agents: a comparison of adding albiglutide, a weekly GLP-1 receptor agonist, versus thrice-daily prandial insulin lispro. Diabetes Care. 2014;37(8):2317-25.

65. Rosenstock J, Kahn SE, Johansen OE, Zinman B, Espeland MA, Woerle HJ, et al. Effect of Linagliptin vs Glimepiride on Major Adverse Cardiovascular Outcomes in Patients With Type 2 Diabetes: The CAROLINA Randomized Clinical Trial. Jama. 2019;322(12):1155-66.

66. Rosenstock J, Perkovic V, Johansen OE, Cooper ME, Kahn SE, Marx N, et al. Effect of Linagliptin vs Placebo on Major Cardiovascular Events in Adults With Type 2 Diabetes and High Cardiovascular and Renal Risk: The CARMELINA Randomized Clinical Trial. Jama. 2019;321(1):69-79.

67. Rosenstock J, Seman LJ, Jelaska A, Hantel S, Pinnetti S, Hach T, et al. Efficacy and safety of empagliflozin, a sodium glucose cotransporter 2 (SGLT2) inhibitor, as add-on to metformin in type 2 diabetes with mild hyperglycaemia. Diabetes Obes Metab. 2013;15(12):1154-60.

68. Rubino D, Abrahamsson N, Davies M, Hesse D, Greenway FL, Jensen C, et al. Effect of Continued Weekly Subcutaneous Semaglutide vs Placebo on Weight Loss Maintenance in Adults With Overweight or Obesity: The STEP 4 Randomized Clinical Trial. Jama. 2021;325(14):1414-25.

69. Schernthaner G, Duran-Garcia S, Hanefeld M, Langslet G, Niskanen L, Ostgren CJ, et al. Efficacy and tolerability of saxagliptin compared with glimepiride in elderly patients with type 2 diabetes: a randomized, controlled study (GENERATION). Diabetes Obes Metab. 2015;17(7):630-8.

70. Scirica BM, Bhatt DL, Braunwald E, Steg PG, Davidson J, Hirshberg B, et al. Saxagliptin and cardiovascular outcomes in patients with type 2 diabetes mellitus. N Engl J Med. 2013;369(14):1317-26.

71. Solomon SD, McMurray JJV, Claggett B, de Boer RA, DeMets D, Hernandez AF, et al. Dapagliflozin in Heart Failure with Mildly Reduced or Preserved Ejection Fraction. N Engl J Med. 2022;387(12):1089-98.

72. Terauchi Y, Yamada Y, Ishida H, Ohsugi M, Kitaoka M, Satoh J, et al. Efficacy and safety of sitagliptin as compared with glimepiride in Japanese patients with type 2 diabetes mellitus aged >/= 60 years (START-J trial). Diabetes Obes Metab. 2017;19(8):1188-92.

73. The E-KCG, Herrington WG, Staplin N, Wanner C, Green JB, Hauske SJ, et al. Empagliflozin in Patients with Chronic Kidney Disease. N Engl J Med. 2023;388(2):117-27.

74. Umpierrez G, Tofe Povedano S, Perez Manghi F, Shurzinske L, Pechtner V. Efficacy and safety of dulaglutide monotherapy versus metformin in type 2 diabetes in a randomized controlled trial (AWARD-3). Diabetes Care. 2014;37(8):2168-76.

75. Wada T, Mori-Anai K, Takahashi A, Matsui T, Inagaki M, Iida M, et al. Effect of canagliflozin on the decline of estimated glomerular filtration rate in chronic kidney disease patients with type 2 diabetes mellitus: A multicenter, randomized, double-blind, placebo-controlled, parallel-group, phase III study in Japan. J Diabetes Investig. 2022;13(12):1981-9.

76. Wason S. Efficacy and Safety of Sotagliflozin Versus Placebo in Participants With Type 2 Diabetes Mellitus Who Have Inadequate Glycemic Control While Taking Insulin Alone or With Other Oral Antidiabetic Agents (SOTA-INS), <https://clinicaltrials.gov/study/NCT03285594?cond=NCT03285594&rank=1>; 2021 [accessed 2024/10/28 2024].

77. Weissman PN, Carr MC, Ye J, Cirkel DT, Stewart M, Perry C, et al. HARMONY 4: randomised clinical trial comparing once-weekly albiglutide and insulin glargine in patients with type 2 diabetes inadequately controlled with metformin with or without sulfonylurea. Diabetologia. 2014;57(12):2475-84.

78. White WB, Cannon CP, Heller SR, Nissen SE, Bergenstal RM, Bakris GL, et al. Alogliptin after acute coronary syndrome in patients with type 2 diabetes. N Engl J Med. 2013;369(14):1327-35.

79. Wilding JP, Woo V, Soler NG, Pahor A, Sugg J, Rohwedder K, et al. Long-term efficacy of dapagliflozin in patients with type 2 diabetes mellitus receiving high doses of insulin: a randomized trial. Ann Intern Med. 2012;156(6):405-15.

80. Wilding JPH, Batterham RL, Calanna S, Davies M, Van Gaal LF, Lingvay I, et al. Once-Weekly Semaglutide in Adults with Overweight or Obesity. N Engl J Med. 2021;384(11):989-1002.

81. Wiviott SD, Raz I, Bonaca MP, Mosenzon O, Kato ET, Cahn A, et al. Dapagliflozin and Cardiovascular Outcomes in Type 2 Diabetes. N Engl J Med. 2019;380(4):347-57.

82. Zinman B, Wanner C, Lachin JM, Fitchett D, Bluhmki E, Hantel S, et al. Empagliflozin, Cardiovascular Outcomes, and Mortality in Type 2 Diabetes. N Engl J Med. 2015;373(22):2117-28.

83. Cadarette SM, Maclure M, Delaney JAC, Whitaker HJ, Hayes KN, Wang SV, et al. Control yourself: ISPE-endorsed guidance in the application of self-controlled study designs in pharmacoepidemiology. Pharmacoepidemiol Drug Saf. 2021;30(6):671-84.

84. International Non-Hodgkin's Lymphoma Prognostic Factors P. A predictive model for aggressive non-Hodgkin's lymphoma. N Engl J Med. 1993;329(14):987-94.

85. Chaimani A, Caldwell DM, Li T, Higgins JPT, Salanti G. Chapter 11: Undertaking network meta-analyses. In: Higgins JPT, Thomas J, Chandler J, Cumpston MS, Li T, Page M, et al., eds. *Cochrane Handbook for Systematic Reviews of Interventions.* London: Cochrane; 2018:7.

86. White IR. Network meta-analysis. Stata J. 2015;15:951-85.

87. Tu YK. Use of generalized linear mixed models for network meta-analysis. Medical decision making : an international journal of the Society for Medical Decision Making. 2014;34(7):911-8.

88. Salanti G, Ades AE, Ioannidis JP. Graphical methods and numerical summaries for presenting results from multiple-treatment meta-analysis: an overview and tutorial. J Clin Epidemiol. 2011;64(2):163-71.

89. Higgins JP, Del Giovane C, Chaimani A, Caldwell DM, Salanti G. Evaluating the Quality of Evidence from a Network Meta-Analysis. Value Health. 2014;17(7):A324.

90. Puhan MA, Schunemann HJ, Murad MH, Li T, Brignardello-Petersen R, Singh JA, et al. A GRADE Working Group approach for rating the quality of treatment effect estimates from network meta-analysis. Bmj. 2014;349:g5630.

91. Borenstein M, Hedges LV, Higgins JP, Rothstein HR. A basic introduction to fixed-effect and random-effects models for meta-analysis. Res Synth Methods. 2010;1(2):97-111.

92. Owen RK, Bradbury N, Xin Y, Cooper N, Sutton A. MetaInsight: An interactive web-based tool for analyzing, interrogating, and visualizing network meta-analyses using R-shiny and netmeta. Res Synth Methods. 2019;10(4):569-81.

93. Cheng J, Pullenayegum E, Marshall JK, Iorio A, Thabane L. Impact of including or excluding both-armed zero-event studies on using standard meta-analysis methods for rare event outcome: a simulation study. BMJ Open. 2016;6(8):e010983.

94. Brockhaus AC, Bender R, Skipka G. The Peto odds ratio viewed as a new effect measure. Stat Med. 2014;33(28):4861-74.

95. Yao M, Deng K, Wang Y, Mei F, Zou K, Li L, et al. Random-effects meta-analysis models for pooling rare events data: a comparison between frequentist and bayesian methods. BMC medical research methodology. 2025;25(1):228.

96. Sweeting MJ, Sutton AJ, Lambert PC. What to add to nothing? Use and avoidance of continuity corrections in meta-analysis of sparse data. Stat Med. 2004;23(9):1351-75.

97. Phillippo DM. multinma: Bayesian Network Meta-Analysis of Individual and Aggregate Data. R package version 0.8.1, <https://dmphillippo.github.io/multinma/>; 2024 [accessed 2025/05/15 2025].

98. Salika T, Turner RM, Fisher D, Tierney JF, White IR. Implications of analysing time-to-event outcomes as binary in meta-analysis: empirical evidence from the Cochrane Database of Systematic Reviews. BMC medical research methodology. 2022;22(1):73.

99. Bradburn MJ, Deeks JJ, Berlin JA, Russell Localio A. Much ado about nothing: a comparison of the performance of meta-analytical methods with rare events. Stat Med. 2007;26(1):53-77.

100. Page MJ, McKenzie JE, Bossuyt PM, Boutron I, Hoffmann TC, Mulrow CD, et al. The PRISMA 2020 statement: an updated guideline for reporting systematic reviews. Bmj. 2021;372:n71.

101. Mattii L, Moscato S, Ippolito C, Polizzi E, Novo G, Zucchi R, et al. Empagliflozin mitigates ponatinib-induced cardiotoxicity by restoring the connexin 43-autophagy pathway. Biomed Pharmacother. 2024;178:117278.

102. Adil M, Kandhare AD, Dalvi G, Ghosh P, Venkata S, Raygude KS, et al. Ameliorative effect of berberine against gentamicin-induced nephrotoxicity in rats via attenuation of oxidative stress, inflammation, apoptosis and mitochondrial dysfunction. Renal failure. 2016;38(6):996-1006.

103. Leiter LA, Cefalu WT, de Bruin TW, Xu J, Parikh S, Johnsson E, et al. Long-term maintenance of efficacy of dapagliflozin in patients with type 2 diabetes mellitus and cardiovascular disease. Diabetes Obes Metab. 2016;18(8):766-74.

104. Nagendra L, Bg H, Sharma M, Dutta D. Semaglutide and cancer: A systematic review and meta-analysis. Diabetes Metab Syndr. 2023;17(9):102834.

105. Zhang L, Xue B, Yu F, Yin Y, Jin S. Deciphering the Causal Relationship between Sodium-glucose Cotransporter 2 Inhibition and Cancer Risks: A Comprehensive Mendelian Randomization Study. J Cancer. 2024;15(12):3903-12.

106. Guo W, Zhao L, Huang W, Chen J, Zhong T, Yan S, et al. Sodium-glucose cotransporter 2 inhibitors, inflammation, and heart failure: a two-sample Mendelian randomization study. Cardiovasc Diabetol. 2024;23(1):118.

107. Wolff Sagy Y, Ramot N, Battat E, Arbel R, Reges O, Dicker D, et al. Glucagon-like peptide-1 receptor agonists compared with bariatric metabolic surgery and the risk of obesity-related cancer: an observational, retrospective cohort study. EClinicalMedicine. 2025;83:103213.

108. Moon S, Choi JW, Park JH, Kim DS, Ahn Y, Kim Y, et al. Association of Appendicular Skeletal Muscle Mass Index and Insulin Resistance With Mortality in Multi-Nationwide Cohorts. Journal of cachexia, sarcopenia and muscle. 2025;16(2):e13811.

109. Bliddal H, Bays H, Czernichow S, Udden Hemmingsson J, Hjelmesaeth J, Hoffmann Morville T, et al. Once-Weekly Semaglutide in Persons with Obesity and Knee Osteoarthritis. N Engl J Med. 2024;391(17):1573-83.

110. Docherty KF, Buendia Lopez R, Folkvaljon F, de Boer RA, Cowie MR, Hammarstedt A, et al. Effect of Dapagliflozin on Accelerometer-Based Measures of Physical Activity in Patients With Heart Failure: An Analysis of the DETERMINE Trials. Circ Heart Fail. 2024;17(10):e012349.

111. Ji L, Agesen RM, Bain SC, Fu F, Gabery S, Geng J, et al. Efficacy and safety of oral semaglutide vs sitagliptin in a predominantly Chinese population with type 2 diabetes uncontrolled with metformin: PIONEER 12, a double-blind, Phase IIIa, randomised trial. Diabetologia. 2024;67(9):1800-16.

112. Lee BW, Cho YM, Kim SG, Ko SH, Lim S, Dahaoui A, et al. Efficacy and Safety of Once-Weekly Semaglutide Versus Once-Daily Sitagliptin as Metformin Add-on in a Korean Population with Type 2 Diabetes. Diabetes Ther. 2024;15(2):547-63.

113. McGowan BM, Bruun JM, Capehorn M, Pedersen SD, Pietilainen KH, Muniraju HAK, et al. Efficacy and safety of once-weekly semaglutide 2.4 mg versus placebo in people with obesity and prediabetes (STEP 10): a randomised, double-blind, placebo-controlled, multicentre phase 3 trial. Lancet Diabetes Endocrinol. 2024;12(9):631-42.

114. McMurray JJV, Docherty KF, de Boer RA, Hammarstedt A, Kitzman DW, Kosiborod MN, et al. Effect of Dapagliflozin Versus Placebo on Symptoms and 6-Minute Walk Distance in Patients With Heart Failure: The DETERMINE Randomized Clinical Trials. Circulation. 2024;149(11):825-38.

115. Mu Y, Bao X, Eliaschewitz FG, Hansen MR, Kim BT, Koroleva A, et al. Efficacy and safety of once weekly semaglutide 2.4 mg for weight management in a predominantly east Asian population with overweight or obesity (STEP 7): a double-blind, multicentre, randomised controlled trial. Lancet Diabetes Endocrinol. 2024;12(3):184-95.

116. Natale P, Tunnicliffe DJ, Toyama T, Palmer SC, Saglimbene VM, Ruospo M, et al. Sodium-glucose co-transporter protein 2 (SGLT2) inhibitors for people with chronic kidney disease and diabetes. The Cochrane database of systematic reviews. 2024;5(5):CD015588.

117. SURMOUNT-J. A Study of Tirzepatide (LY3298176) in Participants With Obesity Disease (SURMOUNT-J), <https://clinicaltrials.gov/study/NCT04844918?cond=NCT04844918&rank=1>; 2024 [accessed 2024/10/28 2024].

118. Tuttle KR, Hauske SJ, Canziani ME, Caramori ML, Cherney D, Cronin L, et al. Efficacy and safety of aldosterone synthase inhibition with and without empagliflozin for chronic kidney disease: a randomised, controlled, phase 2 trial. Lancet. 2024;403(10424):379-90.

119. Wang W, Bain SC, Bian F, Chen R, Gabery S, Huang S, et al. Efficacy and safety of oral semaglutide monotherapy vs placebo in a predominantly Chinese population with type 2 diabetes (PIONEER 11): a double-blind, Phase IIIa, randomised trial. Diabetologia. 2024;67(9):1783-99.

120. Zhao L, Cheng Z, Lu Y, Liu M, Chen H, Zhang M, et al. Tirzepatide for Weight Reduction in Chinese Adults With Obesity: The SURMOUNT-CN Randomized Clinical Trial. Jama. 2024;332(7):551-60.

121. Aroda VR, Frias JP, Ji L, Niemoeller E, Nguyen-Pascal ML, Denkel K, et al. Efficacy and safety of once-weekly efpeglenatide in people with suboptimally controlled type 2 diabetes: The AMPLITUDE-D, AMPLITUDE-L and AMPLITUDE-S randomized controlled trials. Diabetes Obes Metab. 2023;25(8):2084-95.

122. Aroda VR, Aberle J, Bardtrum L, Christiansen E, Knop FK, Gabery S, et al. Efficacy and safety of once-daily oral semaglutide 25 mg and 50 mg compared with 14 mg in adults with type 2 diabetes (PIONEER PLUS): a multicentre, randomised, phase 3b trial. Lancet. 2023;402(10403):693-704.

123. Buse JB, Nordahl Christensen H, Harty BJ, Mitchell J, Soule BP, Zacherle E, et al. Study design and baseline profile for adults with type 2 diabetes in the once-weekly subcutaneous SEmaglutide randomized PRAgmatic (SEPRA) trial. BMJ Open Diabetes Res Care. 2023;11(3).

124. (SURPASS-CN-INS) EL. A Study of Tirzepatide (LY3298176) in Chinese Participants With Type 2 Diabetes (SURPASS-CN-INS) (NCT05691712), <https://clinicaltrials.gov/study/NCT05691712?cond=NCT05691712&rank=1>; 2023 [accessed 2025/9/1 2025].

125. Feng P, Sheng X, Ji Y, Urva S, Wang F, Miller S, et al. A Phase 1 Multiple Dose Study of Tirzepatide in Chinese Patients with Type 2 Diabetes. Adv Ther. 2023;40(8):3434-45.

126. Frias JP, Hsia S, Eyde S, Liu R, Ma X, Konig M, et al. Efficacy and safety of oral orforglipron in patients with type 2 diabetes: a multicentre, randomised, dose-response, phase 2 study. Lancet. 2023;402(10400):472-83.

127. Gao L, Lee BW, Chawla M, Kim J, Huo L, Du L, et al. Tirzepatide versus insulin glargine as second-line or third-line therapy in type 2 diabetes in the Asia-Pacific region: the SURPASS-AP-Combo trial. Nat Med. 2023;29(6):1500-10.

128. Jastreboff AM, Kaplan LM, Frias JP, Wu Q, Du Y, Gurbuz S, et al. Triple-Hormone-Receptor Agonist Retatrutide for Obesity - A Phase 2 Trial. N Engl J Med. 2023;389(6):514-26.

129. Ji L, Lu Y, Li Q, Fu L, Luo Y, Lei T, et al. Efficacy and safety of empagliflozin in combination with insulin in Chinese patients with type 2 diabetes and insufficient glycaemic control: A phase III, randomized, double-blind, placebo-controlled, parallel study. Diabetes Obes Metab. 2023;25(7):1839-48.

130. Kosiborod MN, Abildstrom SZ, Borlaug BA, Butler J, Rasmussen S, Davies M, et al. Semaglutide in Patients with Heart Failure with Preserved Ejection Fraction and Obesity. N Engl J Med. 2023;389(12):1069-84.

131. Loomba R, Abdelmalek MF, Armstrong MJ, Jara M, Kjaer MS, Krarup N, et al. Semaglutide 2.4 mg once weekly in patients with non-alcoholic steatohepatitis-related cirrhosis: a randomised, placebo-controlled phase 2 trial. Lancet Gastroenterol Hepatol. 2023;8(6):511-22.

132. Ramos EL, Dayan CM, Chatenoud L, Sumnik Z, Simmons KM, Szypowska A, et al. Teplizumab and beta-Cell Function in Newly Diagnosed Type 1 Diabetes. N Engl J Med. 2023;389(23):2151-61.

133. Rosenstock J, Frias J, Jastreboff AM, Du Y, Lou J, Gurbuz S, et al. Retatrutide, a GIP, GLP-1 and glucagon receptor agonist, for people with type 2 diabetes: a randomised, double-blind, placebo and active-controlled, parallel-group, phase 2 trial conducted in the USA. Lancet. 2023;402(10401):529-44.

134. Rosenstock J, Frias JP, Rodbard HW, Tofe S, Sears E, Huh R, et al. Tirzepatide vs Insulin Lispro Added to Basal Insulin in Type 2 Diabetes: The SURPASS-6 Randomized Clinical Trial. Jama. 2023;330(17):1631-40.

135. Wadden TA, Chao AM, Machineni S, Kushner R, Ard J, Srivastava G, et al. Tirzepatide after intensive lifestyle intervention in adults with overweight or obesity: the SURMOUNT-3 phase 3 trial. Nat Med. 2023;29(11):2909-18.

136. Dahl D, Onishi Y, Norwood P, Huh R, Bray R, Patel H, et al. Effect of Subcutaneous Tirzepatide vs Placebo Added to Titrated Insulin Glargine on Glycemic Control in Patients With Type 2 Diabetes: The SURPASS-5 Randomized Clinical Trial. Jama. 2022;327(6):534-45.

137. Fox CK, Clark JM, Rudser KD, Ryder JR, Gross AC, Nathan BM, et al. Exenatide for weight-loss maintenance in adolescents with severe obesity: A randomized, placebo-controlled trial. Obesity (Silver Spring). 2022;30(5):1105-15.

138. Frias JP, Choi J, Rosenstock J, Popescu L, Niemoeller E, Muehlen-Bartmer I, et al. Efficacy and Safety of Once-Weekly Efpeglenatide Monotherapy Versus Placebo in Type 2 Diabetes: The AMPLITUDE-M Randomized Controlled Trial. Diabetes Care. 2022;45(7):1592-600.

139. Garvey WT, Batterham RL, Bhatta M, Buscemi S, Christensen LN, Frias JP, et al. Two-year effects of semaglutide in adults with overweight or obesity: the STEP 5 trial. Nat Med. 2022;28(10):2083-91.

140. Heise T, Mari A, DeVries JH, Urva S, Li J, Pratt EJ, et al. Effects of subcutaneous tirzepatide versus placebo or semaglutide on pancreatic islet function and insulin sensitivity in adults with type 2 diabetes: a multicentre, randomised, double-blind, parallel-arm, phase 1 clinical trial. Lancet Diabetes Endocrinol. 2022;10(6):418-29.

141. Inagaki N, Takeuchi M, Oura T, Imaoka T, Seino Y. Efficacy and safety of tirzepatide monotherapy compared with dulaglutide in Japanese patients with type 2 diabetes (SURPASS J-mono): a double-blind, multicentre, randomised, phase 3 trial. Lancet Diabetes Endocrinol. 2022;10(9):623-33.

142. Kadowaki T, Isendahl J, Khalid U, Lee SY, Nishida T, Ogawa W, et al. Semaglutide once a week in adults with overweight or obesity, with or without type 2 diabetes in an east Asian population (STEP 6): a randomised, double-blind, double-dummy, placebo-controlled, phase 3a trial. Lancet Diabetes Endocrinol. 2022;10(3):193-206.

143. Kadowaki T, Chin R, Ozeki A, Imaoka T, Ogawa Y. Safety and efficacy of tirzepatide as an add-on to single oral antihyperglycaemic medication in patients with type 2 diabetes in Japan (SURPASS J-combo): a multicentre, randomised, open-label, parallel-group, phase 3 trial. Lancet Diabetes Endocrinol. 2022;10(9):634-44.

144. Kellerer M, Kaltoft MS, Lawson J, Nielsen LL, Strojek K, Tabak O, et al. Effect of once-weekly semaglutide versus thrice-daily insulin aspart, both as add-on to metformin and optimized insulin glargine treatment in participants with type 2 diabetes (SUSTAIN 11): A randomized, open-label, multinational, phase 3b trial. Diabetes Obes Metab. 2022;24(9):1788-99.

145. Nordisk N. Research Study Investigating How Well Semaglutide Works in People From Thailand and South Korea Living With Obesity, <https://clinicaltrials.gov/study/NCT04998136?cond=NCT04998136&rank=1>; 2022 [accessed 2026/01/31 2026].

146. Rubino DM, Greenway FL, Khalid U, O'Neil PM, Rosenstock J, Sorrig R, et al. Effect of Weekly Subcutaneous Semaglutide vs Daily Liraglutide on Body Weight in Adults With Overweight or Obesity Without Diabetes: The STEP 8 Randomized Clinical Trial. Jama. 2022;327(2):138-50.

147. Spertus JA, Birmingham MC, Nassif M, Damaraju CV, Abbate A, Butler J, et al. The SGLT2 inhibitor canagliflozin in heart failure: the CHIEF-HF remote, patient-centered randomized trial. Nat Med. 2022;28(4):809-13.

148. Tuttle KR, Levin A, Nangaku M, Kadowaki T, Agarwal R, Hauske SJ, et al. Safety of Empagliflozin in Patients With Type 2 Diabetes and Chronic Kidney Disease: Pooled Analysis of Placebo-Controlled Clinical Trials. Diabetes Care. 2022;45(6):1445-52.

149. Voors AA, Angermann CE, Teerlink JR, Collins SP, Kosiborod M, Biegus J, et al. The SGLT2 inhibitor empagliflozin in patients hospitalized for acute heart failure: a multinational randomized trial. Nat Med. 2022;28(3):568-74.

150. Anker SD, Ponikowski P, Wanner C, Pfarr E, Hauske S, Peil B, et al. Kidney Function After Initiation and Discontinuation of Empagliflozin in Patients With Heart Failure With and Without Type 2 Diabetes: Insights From the EMPERIAL Trials. Circulation. 2021;144(15):1265-7.

151. Bhatt DL, Szarek M, Steg PG, Cannon CP, Leiter LA, McGuire DK, et al. Sotagliflozin in Patients with Diabetes and Recent Worsening Heart Failure. N Engl J Med. 2021;384(2):117-28.

152. Kosiborod MN, Esterline R, Furtado RHM, Oscarsson J, Gasparyan SB, Koch GG, et al. Dapagliflozin in patients with cardiometabolic risk factors hospitalised with COVID-19 (DARE-19): a randomised, double-blind, placebo-controlled, phase 3 trial. Lancet Diabetes Endocrinol. 2021;9(9):586-94.

153. Ludvik B, Giorgino F, Jodar E, Frias JP, Fernandez Lando L, Brown K, et al. Once-weekly tirzepatide versus once-daily insulin degludec as add-on to metformin with or without SGLT2 inhibitors in patients with type 2 diabetes (SURPASS-3): a randomised, open-label, parallel-group, phase 3 trial. Lancet. 2021;398(10300):583-98.

154. Nassif ME, Spertus JA, Tang F, Windsor SL, Jones P, Thomas M, et al. Association Between Change in Ambulatory Hemodynamic Pressures and Symptoms of Heart Failure. Circ Heart Fail. 2021;14(11):e008446.

155. Nassif ME, Windsor SL, Borlaug BA, Kitzman DW, Shah SJ, Tang F, et al. The SGLT2 inhibitor dapagliflozin in heart failure with preserved ejection fraction: a multicenter randomized trial. Nat Med. 2021;27(11):1954-60.

156. Rodgers M, Migdal AL, Rodriguez TG, Chen ZZ, Nath AK, Gerszten RE, et al. Weight Loss Outcomes Among Early High Responders to Exenatide Treatment: A Randomized, Placebo Controlled Study in Overweight and Obese Women. Front Endocrinol (Lausanne). 2021;12:742873.

157. Rosenstock J, Wysham C, Frias JP, Kaneko S, Lee CJ, Fernandez Lando L, et al. Efficacy and safety of a novel dual GIP and GLP-1 receptor agonist tirzepatide in patients with type 2 diabetes (SURPASS-1): a double-blind, randomised, phase 3 trial. Lancet. 2021;398(10295):143-55.

158. Santos-Gallego CG, Vargas-Delgado AP, Requena-Ibanez JA, Garcia-Ropero A, Mancini D, Pinney S, et al. Randomized Trial of Empagliflozin in Nondiabetic Patients With Heart Failure and Reduced Ejection Fraction. J Am Coll Cardiol. 2021;77(3):243-55.

159. Stack AG, Han D, Goldwater R, Johansson S, Dronamraju N, Oscarsson J, et al. Dapagliflozin Added to Verinurad Plus Febuxostat Further Reduces Serum Uric Acid in Hyperuricemia: The QUARTZ Study. J Clin Endocrinol Metab. 2021;106(5):e2347-e56.

160. Wadden TA, Bailey TS, Billings LK, Davies M, Frias JP, Koroleva A, et al. Effect of Subcutaneous Semaglutide vs Placebo as an Adjunct to Intensive Behavioral Therapy on Body Weight in Adults With Overweight or Obesity: The STEP 3 Randomized Clinical Trial. Jama. 2021;325(14):1403-13.

161. Wason S. Efficacy and Bone Safety of Sotagliflozin 400 and 200 mg Versus Placebo in Participants With Type 2 Diabetes Mellitus Who Have Inadequate Glycemic Control (SOTA-BONE), <https://clinicaltrials.gov/study/NCT03386344?cond=NCT03386344&rank=1>; 2021 [accessed 2024/10/28 2024].

162. Yang W, Xu X, Lei T, Ma J, Li L, Shen J, et al. Efficacy and safety of linagliptin as add-on therapy to insulin in Chinese patients with type 2 diabetes mellitus: A randomized, double-blind, placebo-controlled trial. Diabetes Obes Metab. 2021;23(2):642-7.

163. Capehorn MS, Catarig AM, Furberg JK, Janez A, Price HC, Tadayon S, et al. Efficacy and safety of once-weekly semaglutide 1.0mg vs once-daily liraglutide 1.2mg as add-on to 1-3 oral antidiabetic drugs in subjects with type 2 diabetes (SUSTAIN 10). Diabetes Metab. 2020;46(2):100-9.

164. Aroda VR, Rosenstock J, Terauchi Y, Altuntas Y, Lalic NM, Morales Villegas EC, et al. PIONEER 1: Randomized Clinical Trial of the Efficacy and Safety of Oral Semaglutide Monotherapy in Comparison With Placebo in Patients With Type 2 Diabetes. Diabetes Care. 2019;42(9):1724-32.

165. Herold KC, Bundy BN, Long SA, Bluestone JA, DiMeglio LA, Dufort MJ, et al. An Anti-CD3 Antibody, Teplizumab, in Relatives at Risk for Type 1 Diabetes. N Engl J Med. 2019;381(7):603-13.

166. Ledesma G, Umpierrez GE, Morley JE, Lewis-D'Agostino D, Keller A, Meinicke T, et al. Efficacy and safety of linagliptin to improve glucose control in older people with type 2 diabetes on stable insulin therapy: A randomized trial. Diabetes Obes Metab. 2019;21(11):2465-73.

167. Lingvay I, Catarig AM, Frias JP, Kumar H, Lausvig NL, le Roux CW, et al. Efficacy and safety of once-weekly semaglutide versus daily canagliflozin as add-on to metformin in patients with type 2 diabetes (SUSTAIN 8): a double-blind, phase 3b, randomised controlled trial. Lancet Diabetes Endocrinol. 2019;7(11):834-44.

168. Mosenzon O, Blicher TM, Rosenlund S, Eriksson JW, Heller S, Hels OH, et al. Efficacy and safety of oral semaglutide in patients with type 2 diabetes and moderate renal impairment (PIONEER 5): a placebo-controlled, randomised, phase 3a trial. Lancet Diabetes Endocrinol. 2019;7(7):515-27.

169. Mullins RJ, Mustapic M, Chia CW, Carlson O, Gulyani S, Tran J, et al. A Pilot Study of Exenatide Actions in Alzheimer's Disease. Curr Alzheimer Res. 2019;16(8):741-52.

170. Pieber TR, Bode B, Mertens A, Cho YM, Christiansen E, Hertz CL, et al. Efficacy and safety of oral semaglutide with flexible dose adjustment versus sitagliptin in type 2 diabetes (PIONEER 7): a multicentre, open-label, randomised, phase 3a trial. Lancet Diabetes Endocrinol. 2019;7(7):528-39.

171. Pollock C, Stefansson B, Reyner D, Rossing P, Sjostrom CD, Wheeler DC, et al. Albuminuria-lowering effect of dapagliflozin alone and in combination with saxagliptin and effect of dapagliflozin and saxagliptin on glycaemic control in patients with type 2 diabetes and chronic kidney disease (DELIGHT): a randomised, double-blind, placebo-controlled trial. Lancet Diabetes Endocrinol. 2019;7(6):429-41.

172. Pratley R, Amod A, Hoff ST, Kadowaki T, Lingvay I, Nauck M, et al. Oral semaglutide versus subcutaneous liraglutide and placebo in type 2 diabetes (PIONEER 4): a randomised, double-blind, phase 3a trial. Lancet. 2019;394(10192):39-50.

173. Rodbard HW, Rosenstock J, Canani LH, Deerochanawong C, Gumprecht J, Lindberg SO, et al. Oral Semaglutide Versus Empagliflozin in Patients With Type 2 Diabetes Uncontrolled on Metformin: The PIONEER 2 Trial. Diabetes Care. 2019;42(12):2272-81.

174. Rosenstock J, Perl S, Johnsson E, Garcia-Sanchez R, Jacob S. Triple therapy with low-dose dapagliflozin plus saxagliptin versus dual therapy with each monocomponent, all added to metformin, in uncontrolled type 2 diabetes. Diabetes Obes Metab. 2019;21(9):2152-62.

175. Wang J, Li HQ, Xu XH, Kong XC, Sun R, Jing T, et al. The Effects of Once-Weekly Dulaglutide and Insulin Glargine on Glucose Fluctuation in Poorly Oral-Antidiabetic Controlled Patients with Type 2 Diabetes Mellitus. Biomed Res Int. 2019;2019:2682657.

176. Zinman B, Aroda VR, Buse JB, Cariou B, Harris SB, Hoff ST, et al. Efficacy, Safety, and Tolerability of Oral Semaglutide Versus Placebo Added to Insulin With or Without Metformin in Patients With Type 2 Diabetes: The PIONEER 8 Trial. Diabetes Care. 2019;42(12):2262-71.

177. Ahmann AJ, Capehorn M, Charpentier G, Dotta F, Henkel E, Lingvay I, et al. Efficacy and Safety of Once-Weekly Semaglutide Versus Exenatide ER in Subjects With Type 2 Diabetes (SUSTAIN 3): A 56-Week, Open-Label, Randomized Clinical Trial. Diabetes Care. 2018;41(2):258-66.

178. Aronson R, Frias J, Goldman A, Darekar A, Lauring B, Terra SG. Long-term efficacy and safety of ertugliflozin monotherapy in patients with inadequately controlled T2DM despite diet and exercise: VERTIS MONO extension study. Diabetes Obes Metab. 2018;20(6):1453-60.

179. Buse JB, Garg SK, Rosenstock J, Bailey TS, Banks P, Bode BW, et al. Sotagliflozin in Combination With Optimized Insulin Therapy in Adults With Type 1 Diabetes: The North American inTandem1 Study. Diabetes Care. 2018;41(9):1970-80.

180. Chen Y, Liu X, Li Q, Ma J, Lv X, Guo L, et al. Saxagliptin add-on therapy in Chinese patients with type 2 diabetes inadequately controlled by insulin with or without metformin: Results from the SUPER study, a randomized, double-blind, placebo-controlled trial. Diabetes Obes Metab. 2018;20(4):1044-9.

181. Coskun T, Sloop KW, Loghin C, Alsina-Fernandez J, Urva S, Bokvist KB, et al. LY3298176, a novel dual GIP and GLP-1 receptor agonist for the treatment of type 2 diabetes mellitus: From discovery to clinical proof of concept. Mol Metab. 2018;18:3-14.

182. Frias JP, Nauck MA, Van J, Kutner ME, Cui X, Benson C, et al. Efficacy and safety of LY3298176, a novel dual GIP and GLP-1 receptor agonist, in patients with type 2 diabetes: a randomised, placebo-controlled and active comparator-controlled phase 2 trial. Lancet. 2018;392(10160):2180-93.

183. Iwamoto N, Matsui A, Kazama H, Oura T. Subgroup Analysis Stratified by Baseline Pancreatic beta-cell Function in a Japanese Study of Dulaglutide in Patients with Type 2 Diabetes. Diabetes Ther. 2018;9(1):383-94.

184. Ludvik B, Frias JP, Tinahones FJ, Wainstein J, Jiang H, Robertson KE, et al. Dulaglutide as add-on therapy to SGLT2 inhibitors in patients with inadequately controlled type 2 diabetes (AWARD-10): a 24-week, randomised, double-blind, placebo-controlled trial. Lancet Diabetes Endocrinol. 2018;6(5):370-81.

185. Muller-Wieland D, Kellerer M, Cypryk K, Skripova D, Rohwedder K, Johnsson E, et al. Efficacy and safety of dapagliflozin or dapagliflozin plus saxagliptin versus glimepiride as add-on to metformin in patients with type 2 diabetes. Diabetes Obes Metab. 2018;20(11):2598-607.

186. O'Neil PM, Birkenfeld AL, McGowan B, Mosenzon O, Pedersen SD, Wharton S, et al. Efficacy and safety of semaglutide compared with liraglutide and placebo for weight loss in patients with obesity: a randomised, double-blind, placebo and active controlled, dose-ranging, phase 2 trial. Lancet. 2018;392(10148):637-49.

187. Pratley RE, Aroda VR, Lingvay I, Ludemann J, Andreassen C, Navarria A, et al. Semaglutide versus dulaglutide once weekly in patients with type 2 diabetes (SUSTAIN 7): a randomised, open-label, phase 3b trial. Lancet Diabetes Endocrinol. 2018;6(4):275-86.

188. Rodbard HW, Lingvay I, Reed J, de la Rosa R, Rose L, Sugimoto D, et al. Semaglutide Added to Basal Insulin in Type 2 Diabetes (SUSTAIN 5): A Randomized, Controlled Trial. J Clin Endocrinol Metab. 2018;103(6):2291-301.

189. Scott R, Morgan J, Zimmer Z, Lam RLH, O'Neill EA, Kaufman KD, et al. A randomized clinical trial of the efficacy and safety of sitagliptin compared with dapagliflozin in patients with type 2 diabetes mellitus and mild renal insufficiency: The CompoSIT-R study. Diabetes Obes Metab. 2018;20(12):2876-84.

190. Seino Y, Terauchi Y, Osonoi T, Yabe D, Abe N, Nishida T, et al. Safety and efficacy of semaglutide once weekly vs sitagliptin once daily, both as monotherapy in Japanese people with type 2 diabetes. Diabetes Obes Metab. 2018;20(2):378-88.

191. Tuttle KR, Lakshmanan MC, Rayner B, Busch RS, Zimmermann AG, Woodward DB, et al. Dulaglutide versus insulin glargine in patients with type 2 diabetes and moderate-to-severe chronic kidney disease (AWARD-7): a multicentre, open-label, randomised trial. Lancet Diabetes Endocrinol. 2018;6(8):605-17.

192. Zhu D, Gan S, Liu Y, Ma J, Dong X, Song W, et al. Dorzagliatin monotherapy in Chinese patients with type 2 diabetes: a dose-ranging, randomised, double-blind, placebo-controlled, phase 2 study. Lancet Diabetes Endocrinol. 2018;6(8):627-36.

193. Ahren B, Masmiquel L, Kumar H, Sargin M, Karsbol JD, Jacobsen SH, et al. Efficacy and safety of once-weekly semaglutide versus once-daily sitagliptin as an add-on to metformin, thiazolidinediones, or both, in patients with type 2 diabetes (SUSTAIN 2): a 56-week, double-blind, phase 3a, randomised trial. Lancet Diabetes Endocrinol. 2017;5(5):341-54.

194. Aroda VR, Bain SC, Cariou B, Piletic M, Rose L, Axelsen M, et al. Efficacy and safety of once-weekly semaglutide versus once-daily insulin glargine as add-on to metformin (with or without sulfonylureas) in insulin-naive patients with type 2 diabetes (SUSTAIN 4): a randomised, open-label, parallel-group, multicentre, multinational, phase 3a trial. Lancet Diabetes Endocrinol. 2017;5(5):355-66.

195. Ba J, Han P, Yuan G, Mo Z, Pan C, Wu F, et al. Randomized trial assessing the safety and efficacy of sitagliptin in Chinese patients with type 2 diabetes mellitus inadequately controlled on sulfonylurea alone or combined with metformin. J Diabetes. 2017;9(7):667-76.

196. Davies M, Pieber TR, Hartoft-Nielsen ML, Hansen OKH, Jabbour S, Rosenstock J. Effect of Oral Semaglutide Compared With Placebo and Subcutaneous Semaglutide on Glycemic Control in Patients With Type 2 Diabetes: A Randomized Clinical Trial. Jama. 2017;318(15):1460-70.

197. Du J, Liang L, Fang H, Xu F, Li W, Shen L, et al. Efficacy and safety of saxagliptin compared with acarbose in Chinese patients with type 2 diabetes mellitus uncontrolled on metformin monotherapy: Results of a Phase IV open-label randomized controlled study (the SMART study). Diabetes Obes Metab. 2017;19(11):1513-20.

198. Gadde KM, Vetter ML, Iqbal N, Hardy E, Ohman P, investigators D-N-s. Efficacy and safety of autoinjected exenatide once-weekly suspension versus sitagliptin or placebo with metformin in patients with type 2 diabetes: The DURATION-NEO-2 randomized clinical study. Diabetes Obes Metab. 2017;19(7):979-88.

199. Gantz I, Okamoto T, Ito Y, Okuyama K, O'Neill EA, Kaufman KD, et al. A randomized, placebo- and sitagliptin-controlled trial of the safety and efficacy of omarigliptin, a once-weekly dipeptidyl peptidase-4 inhibitor, in Japanese patients with type 2 diabetes. Diabetes Obes Metab. 2017;19(11):1602-9.

200. Groop PH, Cooper ME, Perkovic V, Hocher B, Kanasaki K, Haneda M, et al. Linagliptin and its effects on hyperglycaemia and albuminuria in patients with type 2 diabetes and renal dysfunction: the randomized MARLINA-T2D trial. Diabetes Obes Metab. 2017;19(11):1610-9.

201. Handelsman Y, Lauring B, Gantz I, Iredale C, O'Neill EA, Wei Z, et al. A randomized, double-blind, non-inferiority trial evaluating the efficacy and safety of omarigliptin, a once-weekly DPP-4 inhibitor, or glimepiride in patients with type 2 diabetes inadequately controlled on metformin monotherapy. Curr Med Res Opin. 2017;33(10):1861-8.

202. Home PD, Ahren B, Reusch JEB, Rendell M, Weissman PN, Cirkel DT, et al. Three-year data from 5 HARMONY phase 3 clinical trials of albiglutide in type 2 diabetes mellitus: Long-term efficacy with or without rescue therapy. Diabetes Res Clin Pract. 2017;131:49-60.

203. Januzzi JL, Jr., Butler J, Jarolim P, Sattar N, Vijapurkar U, Desai M, et al. Effects of Canagliflozin on Cardiovascular Biomarkers in Older Adults With Type 2 Diabetes. J Am Coll Cardiol. 2017;70(6):704-12.

204. Lee SH, Gantz I, Round E, Latham M, O'Neill EA, Ceesay P, et al. A randomized, placebo-controlled clinical trial evaluating the safety and efficacy of the once-weekly DPP-4 inhibitor omarigliptin in patients with type 2 diabetes mellitus inadequately controlled by glimepiride and metformin. BMC Endocr Disord. 2017;17(1):70.

205. Meneilly GS, Roy-Duval C, Alawi H, Dailey G, Bellido D, Trescoli C, et al. Lixisenatide Therapy in Older Patients With Type 2 Diabetes Inadequately Controlled on Their Current Antidiabetic Treatment: The GetGoal-O Randomized Trial. Diabetes Care. 2017;40(4):485-93.

206. Mu Y, Pan C, Fan B, Hehnke U, Zhang X, Zhang X, et al. Efficacy and safety of linagliptin/metformin single-pill combination as initial therapy in drug-naive Asian patients with type 2 diabetes. Diabetes Res Clin Pract. 2017;124:48-56.

207. Pan C, Han P, Ji Q, Li C, Lu J, Yang J, et al. Efficacy and safety of alogliptin in patients with type 2 diabetes mellitus: A multicentre randomized double-blind placebo-controlled Phase 3 study in mainland China, Taiwan, and Hong Kong. J Diabetes. 2017;9(4):386-95.

208. Shankar RR, Inzucchi SE, Scarabello V, Gantz I, Kaufman KD, Lai E, et al. A randomized clinical trial evaluating the efficacy and safety of the once-weekly dipeptidyl peptidase-4 inhibitor omarigliptin in patients with type 2 diabetes inadequately controlled on metformin monotherapy. Curr Med Res Opin. 2017;33(10):1853-60.

209. Sorli C, Harashima SI, Tsoukas GM, Unger J, Karsbol JD, Hansen T, et al. Efficacy and safety of once-weekly semaglutide monotherapy versus placebo in patients with type 2 diabetes (SUSTAIN 1): a double-blind, randomised, placebo-controlled, parallel-group, multinational, multicentre phase 3a trial. Lancet Diabetes Endocrinol. 2017;5(4):251-60.

210. Tinahones FJ, Gallwitz B, Nordaby M, Gotz S, Maldonado-Lutomirsky M, Woerle HJ, et al. Linagliptin as add-on to empagliflozin and metformin in patients with type 2 diabetes: Two 24-week randomized, double-blind, double-dummy, parallel-group trials. Diabetes Obes Metab. 2017;19(2):266-74.

211. Wang W, Ning G, Ma J, Liu X, Zheng S, Wu F, et al. A randomized clinical trial of the safety and efficacy of sitagliptin in patients with type 2 diabetes mellitus inadequately controlled by acarbose alone. Curr Med Res Opin. 2017;33(4):693-9.

212. Yu M, Brunt KV, Milicevic Z, Varnado O, Boye KS. Patient-reported Outcomes in Patients with Type 2 Diabetes Treated with Dulaglutide Added to Titrated Insulin Glargine (AWARD-9). Clin Ther. 2017;39(11):2284-95.

213. Davies MJ, Bain SC, Atkin SL, Rossing P, Scott D, Shamkhalova MS, et al. Efficacy and Safety of Liraglutide Versus Placebo as Add-on to Glucose-Lowering Therapy in Patients With Type 2 Diabetes and Moderate Renal Impairment (LIRA-RENAL): A Randomized Clinical Trial. Diabetes Care. 2016;39(2):222-30.

214. Dungan KM, Weitgasser R, Perez Manghi F, Pintilei E, Fahrbach JL, Jiang HH, et al. A 24-week study to evaluate the efficacy and safety of once-weekly dulaglutide added on to glimepiride in type 2 diabetes (AWARD-8). Diabetes Obes Metab. 2016;18(5):475-82.

215. Investigators F-ST. Glucose Variability in a 26-Week Randomized Comparison of Mealtime Treatment With Rapid-Acting Insulin Versus GLP-1 Agonist in Participants With Type 2 Diabetes at High Cardiovascular Risk. Diabetes Care. 2016;39(6):973-81.

216. Lepore JJ, Olson E, Demopoulos L, Haws T, Fang Z, Barbour AM, et al. Effects of the Novel Long-Acting GLP-1 Agonist, Albiglutide, on Cardiac Function, Cardiac Metabolism, and Exercise Capacity in Patients With Chronic Heart Failure and Reduced Ejection Fraction. JACC Heart Fail. 2016;4(7):559-66.

217. Margulies KB, Hernandez AF, Redfield MM, Givertz MM, Oliveira GH, Cole R, et al. Effects of Liraglutide on Clinical Stability Among Patients With Advanced Heart Failure and Reduced Ejection Fraction: A Randomized Clinical Trial. Jama. 2016;316(5):500-8.

218. Mellander A, Billger M, Johnsson E, Traff AK, Yoshida S, Johnsson K. Hypersensitivity Events, Including Potentially Hypersensitivity-Related Skin Events, with Dapagliflozin in Patients with Type 2 Diabetes Mellitus: A Pooled Analysis. Clinical drug investigation. 2016;36(11):925-33.

219. Moses RG, Round E, Shentu Y, Golm GT, O'Neill E A, Gantz I, et al. A randomized clinical trial evaluating the safety and efficacy of sitagliptin added to the combination of sulfonylurea and metformin in patients with type 2 diabetes mellitus and inadequate glycemic control. J Diabetes. 2016;8(5):701-11.

220. Wang W, Yang J, Yang G, Gong Y, Patel S, Zhang C, et al. Efficacy and safety of linagliptin in Asian patients with type 2 diabetes mellitus inadequately controlled by metformin: A multinational 24-week, randomized clinical trial. J Diabetes. 2016;8(2):229-37.

221. Zang L, Liu Y, Geng J, Luo Y, Bian F, Lv X, et al. Efficacy and safety of liraglutide versus sitagliptin, both in combination with metformin, in Chinese patients with type 2 diabetes: a 26-week, open-label, randomized, active comparator clinical trial. Diabetes Obes Metab. 2016;18(8):803-11.

222. Blonde L, Jendle J, Gross J, Woo V, Jiang H, Fahrbach JL, et al. Once-weekly dulaglutide versus bedtime insulin glargine, both in combination with prandial insulin lispro, in patients with type 2 diabetes (AWARD-4): a randomised, open-label, phase 3, non-inferiority study. Lancet. 2015;385(9982):2057-66.

223. Cefalu WT, Leiter LA, de Bruin TW, Gause-Nilsson I, Sugg J, Parikh SJ. Dapagliflozin's Effects on Glycemia and Cardiovascular Risk Factors in High-Risk Patients With Type 2 Diabetes: A 24-Week, Multicenter, Randomized, Double-Blind, Placebo-Controlled Study With a 28-Week Extension. Diabetes Care. 2015;38(7):1218-27.

224. Davies MJ, Bergenstal R, Bode B, Kushner RF, Lewin A, Skjoth TV, et al. Efficacy of Liraglutide for Weight Loss Among Patients With Type 2 Diabetes: The SCALE Diabetes Randomized Clinical Trial. Jama. 2015;314(7):687-99.

225. DeFronzo RA, Lewin A, Patel S, Liu D, Kaste R, Woerle HJ, et al. Combination of empagliflozin and linagliptin as second-line therapy in subjects with type 2 diabetes inadequately controlled on metformin. Diabetes Care. 2015;38(3):384-93.

226. Hartley P, Shentu Y, Betz-Schiff P, Golm GT, Sisk CM, Engel SS, et al. Efficacy and Tolerability of Sitagliptin Compared with Glimepiride in Elderly Patients with Type 2 Diabetes Mellitus and Inadequate Glycemic Control: A Randomized, Double-Blind, Non-Inferiority Trial. Drugs Aging. 2015;32(6):469-76.

227. Hirose T, Suzuki M, Tsumiyama I. Efficacy and Safety of Vildagliptin as an Add-on to Insulin with or without Metformin in Japanese Patients with Type 2 Diabetes Mellitus: A 12-week, Double-Blind, Randomized Study. Diabetes Ther. 2015;6(4):559-71.

228. Ji L, Zinman B, Patel S, Ji J, Bailes Z, Thiemann S, et al. Efficacy and safety of linagliptin co-administered with low-dose metformin once daily versus high-dose metformin twice daily in treatment-naive patients with type 2 diabetes: a double-blind randomized trial. Adv Ther. 2015;32(3):201-15.

229. Kovacs CS, Seshiah V, Merker L, Christiansen AV, Roux F, Salsali A, et al. Empagliflozin as Add-on Therapy to Pioglitazone With or Without Metformin in Patients With Type 2 Diabetes Mellitus. Clin Ther. 2015;37(8):1773-88 e1.

230. Laakso M, Rosenstock J, Groop PH, Barnett AH, Gallwitz B, Hehnke U, et al. Treatment with the dipeptidyl peptidase-4 inhibitor linagliptin or placebo followed by glimepiride in patients with type 2 diabetes with moderate to severe renal impairment: a 52-week, randomized, double-blind clinical trial. Diabetes Care. 2015;38(2):e15-7.

231. Mathieu C, Ranetti AE, Li D, Ekholm E, Cook W, Hirshberg B, et al. Randomized, Double-Blind, Phase 3 Trial of Triple Therapy With Dapagliflozin Add-on to Saxagliptin Plus Metformin in Type 2 Diabetes. Diabetes Care. 2015;38(11):2009-17.

232. Mathieu C, Shankar RR, Lorber D, Umpierrez G, Wu F, Xu L, et al. A Randomized Clinical Trial to Evaluate the Efficacy and Safety of Co-Administration of Sitagliptin with Intensively Titrated Insulin Glargine. Diabetes Ther. 2015;6(2):127-42.

233. Matthaei S, Catrinoiu D, Celinski A, Ekholm E, Cook W, Hirshberg B, et al. Randomized, Double-Blind Trial of Triple Therapy With Saxagliptin Add-on to Dapagliflozin Plus Metformin in Patients With Type 2 Diabetes. Diabetes Care. 2015;38(11):2018-24.

234. Roden M, Merker L, Christiansen AV, Roux F, Salsali A, Kim G, et al. Safety, tolerability and effects on cardiometabolic risk factors of empagliflozin monotherapy in drug-naive patients with type 2 diabetes: a double-blind extension of a Phase III randomized controlled trial. Cardiovasc Diabetol. 2015;14:154.

235. Rosenstock J, Hansen L, Zee P, Li Y, Cook W, Hirshberg B, et al. Dual add-on therapy in type 2 diabetes poorly controlled with metformin monotherapy: a randomized double-blind trial of saxagliptin plus dapagliflozin addition versus single addition of saxagliptin or dapagliflozin to metformin. Diabetes Care. 2015;38(3):376-83.

236. Sheu WH, Gantz I, Chen M, Suryawanshi S, Mirza A, Goldstein BJ, et al. Safety and Efficacy of Omarigliptin (MK-3102), a Novel Once-Weekly DPP-4 Inhibitor for the Treatment of Patients With Type 2 Diabetes. Diabetes Care. 2015;38(11):2106-14.

237. Weinstock RS, Guerci B, Umpierrez G, Nauck MA, Skrivanek Z, Milicevic Z. Safety and efficacy of once-weekly dulaglutide versus sitagliptin after 2 years in metformin-treated patients with type 2 diabetes (AWARD-5): a randomized, phase III study. Diabetes Obes Metab. 2015;17(9):849-58.

238. Bajaj M, Gilman R, Patel S, Kempthorne-Rawson J, Lewis-D'Agostino D, Woerle HJ. Linagliptin improved glycaemic control without weight gain or hypoglycaemia in patients with type 2 diabetes inadequately controlled by a combination of metformin and pioglitazone: a 24-week randomized, double-blind study. Diabet Med. 2014;31(12):1505-14.

239. Barnett AH, Mithal A, Manassie J, Jones R, Rattunde H, Woerle HJ, et al. Efficacy and safety of empagliflozin added to existing antidiabetes treatment in patients with type 2 diabetes and chronic kidney disease: a randomised, double-blind, placebo-controlled trial. Lancet Diabetes Endocrinol. 2014;2(5):369-84.

240. Dungan KM, Povedano ST, Forst T, Gonzalez JG, Atisso C, Sealls W, et al. Once-weekly dulaglutide versus once-daily liraglutide in metformin-treated patients with type 2 diabetes (AWARD-6): a randomised, open-label, phase 3, non-inferiority trial. Lancet. 2014;384(9951):1349-57.

241. Henry RR, Staels B, Fonseca VA, Chou MZ, Teng R, Golm GT, et al. Efficacy and safety of initial combination treatment with sitagliptin and pioglitazone--a factorial study. Diabetes Obes Metab. 2014;16(3):223-30.

242. Kadowaki T, Kondo K. Efficacy and safety of teneligliptin added to glimepiride in Japanese patients with type 2 diabetes mellitus: a randomized, double-blind, placebo-controlled study with an open-label, long-term extension. Diabetes Obes Metab. 2014;16(5):418-25.

243. McGill JB, Barnett AH, Lewin AJ, Patel S, Neubacher D, von Eynatten M, et al. Linagliptin added to sulphonylurea in uncontrolled type 2 diabetes patients with moderate-to-severe renal impairment. Diab Vasc Dis Res. 2014;11(1):34-40.

244. Polidori D, Mari A, Ferrannini E. Canagliflozin, a sodium glucose co-transporter 2 inhibitor, improves model-based indices of beta cell function in patients with type 2 diabetes. Diabetologia. 2014;57(5):891-901.

245. Pratley RE, Fleck P, Wilson C. Efficacy and safety of initial combination therapy with alogliptin plus metformin versus either as monotherapy in drug-naive patients with type 2 diabetes: a randomized, double-blind, 6-month study. Diabetes Obes Metab. 2014;16(7):613-21.

246. Pratley RE, Nauck MA, Barnett AH, Feinglos MN, Ovalle F, Harman-Boehm I, et al. Once-weekly albiglutide versus once-daily liraglutide in patients with type 2 diabetes inadequately controlled on oral drugs (HARMONY 7): a randomised, open-label, multicentre, non-inferiority phase 3 study. Lancet Diabetes Endocrinol. 2014;2(4):289-97.

247. Stenlof K, Cefalu WT, Kim KA, Jodar E, Alba M, Edwards R, et al. Long-term efficacy and safety of canagliflozin monotherapy in patients with type 2 diabetes inadequately controlled with diet and exercise: findings from the 52-week CANTATA-M study. Curr Med Res Opin. 2014;30(2):163-75.

248. Van Gaal L, Souhami E, Zhou T, Aronson R. Efficacy and safety of the glucagon-like peptide-1 receptor agonist lixisenatide versus the dipeptidyl peptidase-4 inhibitor sitagliptin in young (<50 years) obese patients with type 2 diabetes mellitus. J Clin Transl Endocrinol. 2014;1(2):31-7.

249. White JL, Buchanan P, Li J, Frederich R. A randomized controlled trial of the efficacy and safety of twice-daily saxagliptin plus metformin combination therapy in patients with type 2 diabetes and inadequate glycemic control on metformin monotherapy. BMC Endocr Disord. 2014;14:17.

250. Wysham C, Blevins T, Arakaki R, Colon G, Garcia P, Atisso C, et al. Efficacy and safety of dulaglutide added onto pioglitazone and metformin versus exenatide in type 2 diabetes in a randomized controlled trial (AWARD-1). Diabetes Care. 2014;37(8):2159-67.

251. Alba M, Ahren B, Inzucchi SE, Guan Y, Mallick M, Xu L, et al. Sitagliptin and pioglitazone provide complementary effects on postprandial glucose and pancreatic islet cell function. Diabetes Obes Metab. 2013;15(12):1101-10.

252. Arjona Ferreira JC, Corry D, Mogensen CE, Sloan L, Xu L, Golm GT, et al. Efficacy and safety of sitagliptin in patients with type 2 diabetes and ESRD receiving dialysis: a 54-week randomized trial. Am J Kidney Dis. 2013;61(4):579-87.

253. Barnett AH, Huisman H, Jones R, von Eynatten M, Patel S, Woerle HJ. Linagliptin for patients aged 70 years or older with type 2 diabetes inadequately controlled with common antidiabetes treatments: a randomised, double-blind, placebo-controlled trial. Lancet. 2013;382(9902):1413-23.

254. Barnett AH, Charbonnel B, Li J, Donovan M, Fleming D, Iqbal N. Saxagliptin add-on therapy to insulin with or without metformin for type 2 diabetes mellitus: 52-week safety and efficacy. Clinical drug investigation. 2013;33(10):707-17.

255. Charbonnel B, Steinberg H, Eymard E, Xu L, Thakkar P, Prabhu V, et al. Efficacy and safety over 26 weeks of an oral treatment strategy including sitagliptin compared with an injectable treatment strategy with liraglutide in patients with type 2 diabetes mellitus inadequately controlled on metformin: a randomised clinical trial. Diabetologia. 2013;56(7):1503-11.

256. Dobs AS, Goldstein BJ, Aschner P, Horton ES, Umpierrez GE, Duran L, et al. Efficacy and safety of sitagliptin added to ongoing metformin and rosiglitazone combination therapy in a randomized placebo-controlled 54-week trial in patients with type 2 diabetes. J Diabetes. 2013;5(1):68-79.

257. Ferrannini E, Berk A, Hantel S, Pinnetti S, Hach T, Woerle HJ, et al. Long-term safety and efficacy of empagliflozin, sitagliptin, and metformin: an active-controlled, parallel-group, randomized, 78-week open-label extension study in patients with type 2 diabetes. Diabetes Care. 2013;36(12):4015-21.

258. Haak T, Meinicke T, Jones R, Weber S, von Eynatten M, Woerle HJ. Initial combination of linagliptin and metformin in patients with type 2 diabetes: efficacy and safety in a randomised, double-blind 1-year extension study. International journal of clinical practice. 2013;67(12):1283-93.

259. Herold KC, Gitelman SE, Ehlers MR, Gottlieb PA, Greenbaum CJ, Hagopian W, et al. Teplizumab (anti-CD3 mAb) treatment preserves C-peptide responses in patients with new-onset type 1 diabetes in a randomized controlled trial: metabolic and immunologic features at baseline identify a subgroup of responders. Diabetes. 2013;62(11):3766-74.

260. Herold KC, Gitelman SE, Willi SM, Gottlieb PA, Waldron-Lynch F, Devine L, et al. Teplizumab treatment may improve C-peptide responses in participants with type 1 diabetes after the new-onset period: a randomised controlled trial. Diabetologia. 2013;56(2):391-400.

261. Kadowaki T, Kondo K. Efficacy, safety and dose-response relationship of teneligliptin, a dipeptidyl peptidase-4 inhibitor, in Japanese patients with type 2 diabetes mellitus. Diabetes Obes Metab. 2013;15(9):810-8.

262. Kadowaki T, Kondo K. Efficacy and safety of teneligliptin in combination with pioglitazone in Japanese patients with type 2 diabetes mellitus. J Diabetes Investig. 2013;4(6):576-84.

263. Lavalle-Gonzalez FJ, Januszewicz A, Davidson J, Tong C, Qiu R, Canovatchel W, et al. Efficacy and safety of canagliflozin compared with placebo and sitagliptin in patients with type 2 diabetes on background metformin monotherapy: a randomised trial. Diabetologia. 2013;56(12):2582-92.

264. Philis-Tsimikas A, Del Prato S, Satman I, Bhargava A, Dharmalingam M, Skjoth TV, et al. Effect of insulin degludec versus sitagliptin in patients with type 2 diabetes uncontrolled on oral antidiabetic agents. Diabetes Obes Metab. 2013;15(8):760-6.

265. Roden M, Weng J, Eilbracht J, Delafont B, Kim G, Woerle HJ, et al. Empagliflozin monotherapy with sitagliptin as an active comparator in patients with type 2 diabetes: a randomised, double-blind, placebo-controlled, phase 3 trial. Lancet Diabetes Endocrinol. 2013;1(3):208-19.

266. Rosenstock J, Gross JL, Aguilar-Salinas C, Hissa M, Berglind N, Ravichandran S, et al. Long-term 4-year safety of saxagliptin in drug-naive and metformin-treated patients with Type 2 diabetes. Diabet Med. 2013;30(12):1472-6.

267. Rosenstock J, Raccah D, Koranyi L, Maffei L, Boka G, Miossec P, et al. Efficacy and safety of lixisenatide once daily versus exenatide twice daily in type 2 diabetes inadequately controlled on metformin: a 24-week, randomized, open-label, active-controlled study (GetGoal-X). Diabetes Care. 2013;36(10):2945-51.

268. Rosenstock J, Wilson C, Fleck P. Alogliptin versus glipizide monotherapy in elderly type 2 diabetes mellitus patients with mild hyperglycaemia: a prospective, double-blind, randomized, 1-year study. Diabetes Obes Metab. 2013;15(10):906-14.

269. Schernthaner G, Gross JL, Rosenstock J, Guarisco M, Fu M, Yee J, et al. Canagliflozin compared with sitagliptin for patients with type 2 diabetes who do not have adequate glycemic control with metformin plus sulfonylurea: a 52-week randomized trial. Diabetes Care. 2013;36(9):2508-15.

270. Yki-Jarvinen H, Rosenstock J, Duran-Garcia S, Pinnetti S, Bhattacharya S, Thiemann S, et al. Effects of adding linagliptin to basal insulin regimen for inadequately controlled type 2 diabetes: a >/=52-week randomized, double-blind study. Diabetes Care. 2013;36(12):3875-81.

271. Barnett AH, Patel S, Harper R, Toorawa R, Thiemann S, von Eynatten M, et al. Linagliptin monotherapy in type 2 diabetes patients for whom metformin is inappropriate: an 18-week randomized, double-blind, placebo-controlled phase III trial with a 34-week active-controlled extension. Diabetes Obes Metab. 2012;14(12):1145-54.

272. DeFronzo RA, Burant CF, Fleck P, Wilson C, Mekki Q, Pratley RE. Efficacy and tolerability of the DPP-4 inhibitor alogliptin combined with pioglitazone, in metformin-treated patients with type 2 diabetes. J Clin Endocrinol Metab. 2012;97(5):1615-22.

273. Frederich R, McNeill R, Berglind N, Fleming D, Chen R. The efficacy and safety of the dipeptidyl peptidase-4 inhibitor saxagliptin in treatment-naive patients with type 2 diabetes mellitus: a randomized controlled trial. Diabetol Metab Syndr. 2012;4(1):36.

274. Gallwitz B, Rosenstock J, Rauch T, Bhattacharya S, Patel S, von Eynatten M, et al. 2-year efficacy and safety of linagliptin compared with glimepiride in patients with type 2 diabetes inadequately controlled on metformin: a randomised, double-blind, non-inferiority trial. Lancet. 2012;380(9840):475-83.

275. Gallwitz B, Guzman J, Dotta F, Guerci B, Simo R, Basson BR, et al. Exenatide twice daily versus glimepiride for prevention of glycaemic deterioration in patients with type 2 diabetes with metformin failure (EUREXA): an open-label, randomised controlled trial. Lancet. 2012;379(9833):2270-8.

276. Haak T, Meinicke T, Jones R, Weber S, von Eynatten M, Woerle HJ. Initial combination of linagliptin and metformin improves glycaemic control in type 2 diabetes: a randomized, double-blind, placebo-controlled study. Diabetes Obes Metab. 2012;14(6):565-74.

277. Hermans MP, Delibasi T, Farmer I, Lohm L, Maheux P, Piatti P, et al. Effects of saxagliptin added to sub-maximal doses of metformin compared with uptitration of metformin in type 2 diabetes: the PROMPT study. Curr Med Res Opin. 2012;28(10):1635-45.

278. Kawamori R, Inagaki N, Araki E, Watada H, Hayashi N, Horie Y, et al. Linagliptin monotherapy provides superior glycaemic control versus placebo or voglibose with comparable safety in Japanese patients with type 2 diabetes: a randomized, placebo and active comparator-controlled, double-blind study. Diabetes Obes Metab. 2012;14(4):348-57.

279. Lewin AJ, Arvay L, Liu D, Patel S, von Eynatten M, Woerle HJ. Efficacy and tolerability of linagliptin added to a sulfonylurea regimen in patients with inadequately controlled type 2 diabetes mellitus: an 18-week, multicenter, randomized, double-blind, placebo-controlled trial. Clin Ther. 2012;34(9):1909-19 e15.

280. Pan CY, Yang W, Tou C, Gause-Nilsson I, Zhao J. Efficacy and safety of saxagliptin in drug-naive Asian patients with type 2 diabetes mellitus: a randomized controlled trial. Diabetes/metabolism research and reviews. 2012;28(3):268-75.

281. Rosenstock J, Aggarwal N, Polidori D, Zhao Y, Arbit D, Usiskin K, et al. Dose-ranging effects of canagliflozin, a sodium-glucose cotransporter 2 inhibitor, as add-on to metformin in subjects with type 2 diabetes. Diabetes Care. 2012;35(6):1232-8.

282. Ross SA, Rafeiro E, Meinicke T, Toorawa R, Weber-Born S, Woerle HJ. Efficacy and safety of linagliptin 2.5 mg twice daily versus 5 mg once daily in patients with type 2 diabetes inadequately controlled on metformin: a randomised, double-blind, placebo-controlled trial. Curr Med Res Opin. 2012;28(9):1465-74.

283. Russell-Jones D, Cuddihy RM, Hanefeld M, Kumar A, Gonzalez JG, Chan M, et al. Efficacy and safety of exenatide once weekly versus metformin, pioglitazone, and sitagliptin used as monotherapy in drug-naive patients with type 2 diabetes (DURATION-4): a 26-week double-blind study. Diabetes Care. 2012;35(2):252-8.

284. Seino Y, Hiroi S, Hirayama M, Kaku K. Efficacy and safety of alogliptin added to sulfonylurea in Japanese patients with type 2 diabetes: A randomized, double-blind, placebo-controlled trial with an open-label, long-term extension study. J Diabetes Investig. 2012;3(6):517-25.

285. Seino Y, Miyata Y, Hiroi S, Hirayama M, Kaku K. Efficacy and safety of alogliptin added to metformin in Japanese patients with type 2 diabetes: a randomized, double-blind, placebo-controlled trial with an open-label, long-term extension study. Diabetes Obes Metab. 2012;14(10):927-36.

286. Yang W, Guan Y, Shentu Y, Li Z, Johnson-Levonas AO, Engel SS, et al. The addition of sitagliptin to ongoing metformin therapy significantly improves glycemic control in Chinese patients with type 2 diabetes. J Diabetes. 2012;4(3):227-37.

287. Barzilai N, Guo H, Mahoney EM, Caporossi S, Golm GT, Langdon RB, et al. Efficacy and tolerability of sitagliptin monotherapy in elderly patients with type 2 diabetes: a randomized, double-blind, placebo-controlled trial. Curr Med Res Opin. 2011;27(5):1049-58.

288. Gallwitz B, Bohmer M, Segiet T, Molle A, Milek K, Becker B, et al. Exenatide twice daily versus premixed insulin aspart 70/30 in metformin-treated patients with type 2 diabetes: a randomized 26-week study on glycemic control and hypoglycemia. Diabetes Care. 2011;34(3):604-6.

289. Gomis R, Espadero RM, Jones R, Woerle HJ, Dugi KA. Efficacy and safety of initial combination therapy with linagliptin and pioglitazone in patients with inadequately controlled type 2 diabetes: a randomized, double-blind, placebo-controlled study. Diabetes Obes Metab. 2011;13(7):653-61.

290. Hollander PL, Li J, Frederich R, Allen E, Chen R, Investigators CV. Safety and efficacy of saxagliptin added to thiazolidinedione over 76 weeks in patients with type 2 diabetes mellitus. Diab Vasc Dis Res. 2011;8(2):125-35.

291. Nowicki M, Rychlik I, Haller H, Warren ML, Suchower L, Gause-Nilsson I, et al. Saxagliptin improves glycaemic control and is well tolerated in patients with type 2 diabetes mellitus and renal impairment. Diabetes Obes Metab. 2011;13(6):523-32.

292. Owens DR, Swallow R, Dugi KA, Woerle HJ. Efficacy and safety of linagliptin in persons with type 2 diabetes inadequately controlled by a combination of metformin and sulphonylurea: a 24-week randomized study. Diabet Med. 2011;28(11):1352-61.

293. Reasner C, Olansky L, Seck TL, Williams-Herman DE, Chen M, Terranella L, et al. The effect of initial therapy with the fixed-dose combination of sitagliptin and metformin compared with metformin monotherapy in patients with type 2 diabetes mellitus. Diabetes Obes Metab. 2011;13(7):644-52.

294. Seino Y, Fujita T, Hiroi S, Hirayama M, Kaku K. Alogliptin plus voglibose in Japanese patients with type 2 diabetes: a randomized, double-blind, placebo-controlled trial with an open-label, long-term extension. Curr Med Res Opin. 2011;27 Suppl 3:21-9.

295. Yang W, Pan CY, Tou C, Zhao J, Gause-Nilsson I. Efficacy and safety of saxagliptin added to metformin in Asian people with type 2 diabetes mellitus: a randomized controlled trial. Diabetes Res Clin Pract. 2011;94(2):217-24.

296. Bergenstal RM, Wysham C, Macconell L, Malloy J, Walsh B, Yan P, et al. Efficacy and safety of exenatide once weekly versus sitagliptin or pioglitazone as an adjunct to metformin for treatment of type 2 diabetes (DURATION-2): a randomised trial. Lancet. 2010;376(9739):431-9.

297. Trials GC. A Study of the Efficacy and Safety of Albiglutide in Subjects With Type 2 Diabetes With Renal Impairment (NCT01098539), <https://clinicaltrials.gov/study/NCT01098539?cond=NCT01098539&rank=1>; 2010 [accessed 2025/7/22 2025].

298. Iwamoto Y, Taniguchi T, Nonaka K, Okamoto T, Okuyama K, Arjona Ferreira JC, et al. Dose-ranging efficacy of sitagliptin, a dipeptidyl peptidase-4 inhibitor, in Japanese patients with type 2 diabetes mellitus. Endocr J. 2010;57(5):383-94.

299. Pratley RE, Nauck M, Bailey T, Montanya E, Cuddihy R, Filetti S, et al. Liraglutide versus sitagliptin for patients with type 2 diabetes who did not have adequate glycaemic control with metformin: a 26-week, randomised, parallel-group, open-label trial. Lancet. 2010;375(9724):1447-56.

300. Rosenstock J, Inzucchi SE, Seufert J, Fleck PR, Wilson CA, Mekki Q. Initial combination therapy with alogliptin and pioglitazone in drug-naive patients with type 2 diabetes. Diabetes Care. 2010;33(11):2406-8.

301. Vilsboll T, Rosenstock J, Yki-Jarvinen H, Cefalu WT, Chen Y, Luo E, et al. Efficacy and safety of sitagliptin when added to insulin therapy in patients with type 2 diabetes. Diabetes Obes Metab. 2010;12(2):167-77.

302. Chacra AR, Tan GH, Apanovitch A, Ravichandran S, List J, Chen R, et al. Saxagliptin added to a submaximal dose of sulphonylurea improves glycaemic control compared with uptitration of sulphonylurea in patients with type 2 diabetes: a randomised controlled trial. International journal of clinical practice. 2009;63(9):1395-406.

303. Lilly E. Protege Encore Study- Clinical Trial of Teplizumab (MGA031) in Children and Adults With Recent-Onset Type 1 Diabetes Mellitus (Protege Encore Study), <https://clinicaltrials.gov/study/NCT00920582?cond=NCT00920582&rank=1>; 2009 [accessed 2025/05/15 2025].

304. Garber A, Henry R, Ratner R, Garcia-Hernandez PA, Rodriguez-Pattzi H, Olvera-Alvarez I, et al. Liraglutide versus glimepiride monotherapy for type 2 diabetes (LEAD-3 Mono): a randomised, 52-week, phase III, double-blind, parallel-treatment trial. Lancet. 2009;373(9662):473-81.

305. Jadzinsky M, Pfutzner A, Paz-Pacheco E, Xu Z, Allen E, Chen R, et al. Saxagliptin given in combination with metformin as initial therapy improves glycaemic control in patients with type 2 diabetes compared with either monotherapy: a randomized controlled trial. Diabetes Obes Metab. 2009;11(6):611-22.

306. Mohan V, Yang W, Son HY, Xu L, Noble L, Langdon RB, et al. Efficacy and safety of sitagliptin in the treatment of patients with type 2 diabetes in China, India, and Korea. Diabetes Res Clin Pract. 2009;83(1):106-16.

307. Nauck MA, Ellis GC, Fleck PR, Wilson CA, Mekki Q, Alogliptin Study G. Efficacy and safety of adding the dipeptidyl peptidase-4 inhibitor alogliptin to metformin therapy in patients with type 2 diabetes inadequately controlled with metformin monotherapy: a multicentre, randomised, double-blind, placebo-controlled study. International journal of clinical practice. 2009;63(1):46-55.

308. Pratley RE, Kipnes MS, Fleck PR, Wilson C, Mekki Q, Alogliptin Study G. Efficacy and safety of the dipeptidyl peptidase-4 inhibitor alogliptin in patients with type 2 diabetes inadequately controlled by glyburide monotherapy. Diabetes Obes Metab. 2009;11(2):167-76.

309. Pratley RE, Reusch JE, Fleck PR, Wilson CA, Mekki Q, Alogliptin Study G. Efficacy and safety of the dipeptidyl peptidase-4 inhibitor alogliptin added to pioglitazone in patients with type 2 diabetes: a randomized, double-blind, placebo-controlled study. Curr Med Res Opin. 2009;25(10):2361-71.

310. Rosenstock J, Rendell MS, Gross JL, Fleck PR, Wilson CA, Mekki Q. Alogliptin added to insulin therapy in patients with type 2 diabetes reduces HbA(1C) without causing weight gain or increased hypoglycaemia. Diabetes Obes Metab. 2009;11(12):1145-52.

311. DeFronzo RA, Fleck PR, Wilson CA, Mekki Q, Alogliptin Study G. Efficacy and safety of the dipeptidyl peptidase-4 inhibitor alogliptin in patients with type 2 diabetes and inadequate glycemic control: a randomized, double-blind, placebo-controlled study. Diabetes Care. 2008;31(12):2315-7.

312. Goldstein BJ, Feinglos MN, Lunceford JK, Johnson J, Williams-Herman DE, Sitagliptin 036 Study G. Effect of initial combination therapy with sitagliptin, a dipeptidyl peptidase-4 inhibitor, and metformin on glycemic control in patients with type 2 diabetes. Diabetes Care. 2007;30(8):1979-87.

313. Rosenstock J, Brazg R, Andryuk PJ, Lu K, Stein P, Sitagliptin Study G. Efficacy and safety of the dipeptidyl peptidase-4 inhibitor sitagliptin added to ongoing pioglitazone therapy in patients with type 2 diabetes: a 24-week, multicenter, randomized, double-blind, placebo-controlled, parallel-group study. Clin Ther. 2006;28(10):1556-68.

314. Akasaka H, Sugimoto K, Shintani A, Taniuchi S, Yamamoto K, Iwakura K, et al. Effects of ipragliflozin on left ventricular diastolic function in patients with type 2 diabetes and heart failure with preserved ejection fraction: The EXCEED randomized controlled multicenter study. Geriatr Gerontol Int. 2022;22(4):298-304.

315. Arturi F, Succurro E, Miceli S, Cloro C, Ruffo M, Maio R, et al. Liraglutide improves cardiac function in patients with type 2 diabetes and chronic heart failure. Endocrine. 2017;57(3):464-73.

316. Bailey TS, Takacs R, Tinahones FJ, Rao PV, Tsoukas GM, Thomsen AB, et al. Efficacy and safety of switching from sitagliptin to liraglutide in subjects with type 2 diabetes (LIRA-SWITCH): a randomized, double-blind, double-dummy, active-controlled 26-week trial. Diabetes Obes Metab. 2016;18(12):1191-8.

317. Bergenstal RM, Forti A, Chiasson JL, Woloschak M, Boldrin M, Balena R. Efficacy and safety of taspoglutide versus sitagliptin for type 2 diabetes mellitus (T-emerge 4 trial). Diabetes Ther. 2012;3(1):13.

318. Blonde L, Dagogo-Jack S, Banerji MA, Pratley RE, Marcellari A, Braceras R, et al. Comparison of vildagliptin and thiazolidinedione as add-on therapy in patients inadequately controlled with metformin: results of the GALIANT trial--a primary care, type 2 diabetes study. Diabetes Obes Metab. 2009;11(10):978-86.

319. Bosi E, Camisasca RP, Collober C, Rochotte E, Garber AJ. Effects of vildagliptin on glucose control over 24 weeks in patients with type 2 diabetes inadequately controlled with metformin. Diabetes Care. 2007;30(4):890-5.

320. Bosi E, Dotta F, Jia Y, Goodman M. Vildagliptin plus metformin combination therapy provides superior glycaemic control to individual monotherapy in treatment-naive patients with type 2 diabetes mellitus. Diabetes Obes Metab. 2009;11(5):506-15.

321. Carbone S, Billingsley HE, Canada JM, Bressi E, Rotelli B, Kadariya D, et al. The effects of canagliflozin compared to sitagliptin on cardiorespiratory fitness in type 2 diabetes mellitus and heart failure with reduced ejection fraction: The CANA-HF study. Diabetes/metabolism research and reviews. 2020;36(8):e3335.

322. Chan JC, Scott R, Arjona Ferreira JC, Sheng D, Gonzalez E, Davies MJ, et al. Safety and efficacy of sitagliptin in patients with type 2 diabetes and chronic renal insufficiency. Diabetes Obes Metab. 2008;10(7):545-55.

323. Chow E, Wang K, Lim CKP, Tsoi STF, Fan B, Poon E, et al. Dorzagliatin, a Dual-Acting Glucokinase Activator, Increases Insulin Secretion and Glucose Sensitivity in Glucokinase Maturity-Onset Diabetes of the Young and Recent-Onset Type 2 Diabetes. Diabetes. 2023;72(2):299-308.

324. Dayem KA, Younis O, Zarif B, Attia S, AbdelSalam A. Impact of dapagliflozin on cardiac function following anterior myocardial infarction in non-diabetic patients - DACAMI (a randomized controlled clinical trial). Int J Cardiol. 2023;379:9-14.

325. de Boer SA, Heerspink HJL, Juarez Orozco LE, van Roon AM, Kamphuisen PW, Smit AJ, et al. Effect of linagliptin on pulse wave velocity in early type 2 diabetes: A randomized, double-blind, controlled 26-week trial (RELEASE). Diabetes Obes Metab. 2017;19(8):1147-54.

326. Dei Cas A, Micheli MM, Aldigeri R, Gardini S, Ferrari-Pellegrini F, Perini M, et al. Long-acting exenatide does not prevent cognitive decline in mild cognitive impairment: a proof-of-concept clinical trial. Journal of endocrinological investigation. 2024;47(9):2339-49.

327. Dubourg J, Perrimond-Dauchy S, Felices M, Bolze S, Voiriot P, Fouqueray P. Absence of QTc prolongation in a thorough QT study with imeglimin, a first in class oral agent for type 2 diabetes mellitus. Eur J Clin Pharmacol. 2020;76(10):1393-400.

328. Dubourg J, Ueki K, Grouin JM, Fouqueray P. Efficacy and safety of imeglimin in Japanese patients with type 2 diabetes: A 24-week, randomized, double-blind, placebo-controlled, dose-ranging phase 2b trial. Diabetes Obes Metab. 2021;23(3):800-10.

329. Dubourg J, Fouqueray P, Thang C, Grouin JM, Ueki K. Efficacy and Safety of Imeglimin Monotherapy Versus Placebo in Japanese Patients With Type 2 Diabetes (TIMES 1): A Double-Blind, Randomized, Placebo-Controlled, Parallel-Group, Multicenter Phase 3 Trial. Diabetes Care. 2021;44(4):952-9.

330. Ejiri K, Miyoshi T, Kihara H, Hata Y, Nagano T, Takaishi A, et al. Effect of Luseogliflozin on Heart Failure With Preserved Ejection Fraction in Patients With Diabetes Mellitus. J Am Heart Assoc. 2020;9(16):e015103.

331. Fonseca V, Schweizer A, Albrecht D, Baron MA, Chang I, Dejager S. Addition of vildagliptin to insulin improves glycaemic control in type 2 diabetes. Diabetologia. 2007;50(6):1148-55.

332. Fouqueray P, Pirags V, Inzucchi SE, Bailey CJ, Schernthaner G, Diamant M, et al. The efficacy and safety of imeglimin as add-on therapy in patients with type 2 diabetes inadequately controlled with metformin monotherapy. Diabetes Care. 2013;36(3):565-8.

333. Fouqueray P, Pirags V, Diamant M, Schernthaner G, Lebovitz HE, Inzucchi SE, et al. The efficacy and safety of imeglimin as add-on therapy in patients with type 2 diabetes inadequately controlled with sitagliptin monotherapy. Diabetes Care. 2014;37(7):1924-30.

334. Garber AJ, Schweizer A, Baron MA, Rochotte E, Dejager S. Vildagliptin in combination with pioglitazone improves glycaemic control in patients with type 2 diabetes failing thiazolidinedione monotherapy: a randomized, placebo-controlled study. Diabetes Obes Metab. 2007;9(2):166-74.

335. Garber AJ, Foley JE, Banerji MA, Ebeling P, Gudbjornsdottir S, Camisasca RP, et al. Effects of vildagliptin on glucose control in patients with type 2 diabetes inadequately controlled with a sulphonylurea. Diabetes Obes Metab. 2008;10(11):1047-56.

336. Gu T, Ma J, Zhang Q, Zhu L, Zhang H, Xu L, et al. Comparative effect of saxagliptin and glimepiride with a composite endpoint of adequate glycaemic control without hypoglycaemia and without weight gain in patients uncontrolled with metformin therapy: Results from the SPECIFY study, a 48-week, multi-centre, randomized, controlled trial. Diabetes Obes Metab. 2019;21(4):939-48.

337. Hagi K, Kochi K, Watada H, Kaku K, Ueki K. Effect of patient characteristics on the efficacy and safety of imeglimin monotherapy in Japanese patients with type 2 diabetes mellitus: A post-hoc analysis of two randomized, placebo-controlled trials. J Diabetes Investig. 2023;14(9):1101-9.

338. Hagi K, Kochi K, Watada H, Kaku K, Ueki K. Factors contributing to the clinical effectiveness of imeglimin monotherapy in Japanese patients with type 2 diabetes mellitus. J Diabetes Investig. 2024;15(9):1239-47.

339. Hagi K, Kochi K, Watada H, Kaku K, Ueki K. Differences in imeglimin response in subgroups of patients with type 2 diabetes stratified by data-driven cluster analysis: A post-hoc analysis of imeglimin clinical trial data. Diabetes Obes Metab. 2024;26(9):3732-42.

340. Hanefeld M, Herman GA, Wu M, Mickel C, Sanchez M, Stein PP, et al. Once-daily sitagliptin, a dipeptidyl peptidase-4 inhibitor, for the treatment of patients with type 2 diabetes. Curr Med Res Opin. 2007;23(6):1329-39.

341. Hao Z, Zhang Y. Different Doses of Empagliflozin in Patients with Heart Failure with Reduced Ejection Fraction. Int Heart J. 2022;63(5):852-6.

342. Henry RR, Smith SR, Schwartz SL, Mudaliar SR, Deacon CF, Holst JJ, et al. Effects of saxagliptin on beta-cell stimulation and insulin secretion in patients with type 2 diabetes. Diabetes Obes Metab. 2011;13(9):850-8.

343. Hermansen K, Kipnes M, Luo E, Fanurik D, Khatami H, Stein P, et al. Efficacy and safety of the dipeptidyl peptidase-4 inhibitor, sitagliptin, in patients with type 2 diabetes mellitus inadequately controlled on glimepiride alone or on glimepiride and metformin. Diabetes Obes Metab. 2007;9(5):733-45.

344. Hollander P, Li J, Allen E, Chen R, Investigators CV. Saxagliptin added to a thiazolidinedione improves glycemic control in patients with type 2 diabetes and inadequate control on thiazolidinedione alone. J Clin Endocrinol Metab. 2009;94(12):4810-9.

345. Ito J, Hagi K, Kochi K, Ueki K, Watada H, Kaku K. Gastrointestinal symptoms in patients receiving imeglimin in combination with metformin: A post-hoc analysis of imeglimin clinical trial data. J Diabetes Investig. 2025;16(4):629-38.

346. Ji L, Li L, Kuang J, Yang T, Kim DJ, Kadir AA, et al. Efficacy and safety of fixed-dose combination therapy, alogliptin plus metformin, in Asian patients with type 2 diabetes: A phase 3 trial. Diabetes Obes Metab. 2017;19(5):754-8.

347. Kaku K, Shimoda M, Osonoi T, Iwamoto M, Kaneto H. Efficacy and safety of imeglimin add-on to DPP-4 inhibitor therapy in Japanese patients with type 2 diabetes mellitus: An interim analysis of the randomised, double-blind FAMILIAR trial. Diabetes Obes Metab. 2025;27(6):3212-22.

348. Kanazawa I, Tanaka KI, Notsu M, Tanaka S, Kiyohara N, Koike S, et al. Long-term efficacy and safety of vildagliptin add-on therapy in type 2 diabetes mellitus with insulin treatment. Diabetes Res Clin Pract. 2017;123:9-17.

349. Katsuno T, Shiraiwa T, Iwasaki S, Park H, Watanabe N, Kaneko S, et al. Benefit of Early Add-on of Linagliptin to Insulin in Japanese Patients With Type 2 Diabetes Mellitus: Randomized-Controlled Open-Label Trial (TRUST2). Adv Ther. 2021;38(3):1514-35.

350. Kikuchi M, Abe N, Kato M, Terao S, Mimori N, Tachibana H. Vildagliptin dose-dependently improves glycemic control in Japanese patients with type 2 diabetes mellitus. Diabetes Res Clin Pract. 2009;83(2):233-40.

351. Kikuchi M, Haneda M, Koya D, Tobe K, Onishi Y, Couturier A, et al. Efficacy and tolerability of vildagliptin as an add-on to glimepiride in Japanese patients with Type 2 diabetes mellitus. Diabetes Res Clin Pract. 2010;89(3):216-23.

352. Kim MK, Rhee EJ, Han KA, Woo AC, Lee MK, Ku BJ, et al. Efficacy and safety of teneligliptin, a dipeptidyl peptidase-4 inhibitor, combined with metformin in Korean patients with type 2 diabetes mellitus: a 16-week, randomized, double-blind, placebo-controlled phase III trial. Diabetes Obes Metab. 2015;17(3):309-12.

353. Kothny W, Shao Q, Groop PH, Lukashevich V. One-year safety, tolerability and efficacy of vildagliptin in patients with type 2 diabetes and moderate or severe renal impairment. Diabetes Obes Metab. 2012;14(11):1032-9.

354. Kothny W, Foley J, Kozlovski P, Shao Q, Gallwitz B, Lukashevich V. Improved glycaemic control with vildagliptin added to insulin, with or without metformin, in patients with type 2 diabetes mellitus. Diabetes Obes Metab. 2013;15(3):252-7.

355. Lee MMY, Brooksbank KJM, Wetherall K, Mangion K, Roditi G, Campbell RT, et al. Effect of Empagliflozin on Left Ventricular Volumes in Patients With Type 2 Diabetes, or Prediabetes, and Heart Failure With Reduced Ejection Fraction (SUGAR-DM-HF). Circulation. 2021;143(6):516-25.

356. Lukashevich V, Del Prato S, Araga M, Kothny W. Efficacy and safety of vildagliptin in patients with type 2 diabetes mellitus inadequately controlled with dual combination of metformin and sulphonylurea. Diabetes Obes Metab. 2014;16(5):403-9.

357. Macauley M, Hollingsworth KG, Smith FE, Thelwall PE, Al-Mrabeh A, Schweizer A, et al. Effect of vildagliptin on hepatic steatosis. J Clin Endocrinol Metab. 2015;100(4):1578-85.

358. McGill JB, Sloan L, Newman J, Patel S, Sauce C, von Eynatten M, et al. Long-term efficacy and safety of linagliptin in patients with type 2 diabetes and severe renal impairment: a 1-year, randomized, double-blind, placebo-controlled study. Diabetes Care. 2013;36(2):237-44.

359. McMurray JJV, Ponikowski P, Bolli GB, Lukashevich V, Kozlovski P, Kothny W, et al. Effects of Vildagliptin on Ventricular Function in Patients With Type 2 Diabetes Mellitus and Heart Failure: A Randomized Placebo-Controlled Trial. JACC Heart Fail. 2018;6(1):8-17.

360. Mita T, Katakami N, Yoshii H, Onuma T, Kaneto H, Osonoi T, et al. Alogliptin, a Dipeptidyl Peptidase 4 Inhibitor, Prevents the Progression of Carotid Atherosclerosis in Patients With Type 2 Diabetes: The Study of Preventive Effects of Alogliptin on Diabetic Atherosclerosis (SPEAD-A). Diabetes Care. 2016;39(1):139-48.

361. Mita T, Katakami N, Shiraiwa T, Yoshii H, Onuma T, Kuribayashi N, et al. Sitagliptin Attenuates the Progression of Carotid Intima-Media Thickening in Insulin-Treated Patients With Type 2 Diabetes: The Sitagliptin Preventive Study of Intima-Media Thickness Evaluation (SPIKE): A Randomized Controlled Trial. Diabetes Care. 2016;39(3):455-64.

362. Neves JS, Vasques-Novoa F, Borges-Canha M, Leite AR, Sharma A, Carvalho D, et al. Risk of adverse events with liraglutide in heart failure with reduced ejection fraction: A post hoc analysis of the FIGHT trial. Diabetes Obes Metab. 2023;25(1):189-97.

363. Nielsen R, Jorsal A, Tougaard RS, Rasmussen JJ, Schou M, Videbaek L, et al. The impact of the glucagon-like peptide-1 receptor agonist liraglutide on natriuretic peptides in heart failure patients with reduced ejection fraction with and without type 2 diabetes. Diabetes Obes Metab. 2020;22(11):2141-50.

364. Ning G, Wang W, Li L, Ma J, Lv X, Yang M, et al. Vildagliptin as add-on therapy to insulin improves glycemic control without increasing risk of hypoglycemia in Asian, predominantly Chinese, patients with type 2 diabetes mellitus. J Diabetes. 2016;8(3):345-53.

365. Nomoto H, Takahashi A, Nakamura A, Kurihara H, Takeuchi J, Nagai S, et al. Add-on imeglimin versus metformin dose escalation regarding glycemic control in patients with type 2 diabetes treated with a dipeptidyl peptidase-4 inhibitor plus low-dose metformin: study protocol for a multicenter, prospective, randomized, open-label, parallel-group comparison study (MEGMI study). BMJ Open Diabetes Res Care. 2022;10(6).

366. Nordisk N. A Research Study to Look Into How Well Semaglutide Medicine Works at Different Doses in People With Type 2 Diabetes and Overweight, <https://clinicaltrials.gov/study/NCT05486065?cond=NCT05486065&rank=1>; 2022 [accessed 2025/05/20 2025].

367. Odawara M, Hamada I, Suzuki M. Efficacy and Safety of Vildagliptin as Add-on to Metformin in Japanese Patients with Type 2 Diabetes Mellitus. Diabetes Ther. 2014;5(1):169-81.

368. Olansky L, Reasner C, Seck TL, Williams-Herman DE, Chen M, Terranella L, et al. A treatment strategy implementing combination therapy with sitagliptin and metformin results in superior glycaemic control versus metformin monotherapy due to a low rate of addition of antihyperglycaemic agents. Diabetes Obes Metab. 2011;13(9):841-9.

369. Oyanagi T, Kawanabe S, Tsukiyama H, Nishine A, Nakamura Y, Nakagawa T, et al. The Effects of Imeglimin on Muscle Strength in Patients with Type 2 Diabetes: A Prospective Cohort Study. Diabetes Ther. 2024;15(11):2323-36.

370. Pacini G, Mari A, Fouqueray P, Bolze S, Roden M. Imeglimin increases glucose-dependent insulin secretion and improves beta-cell function in patients with type 2 diabetes. Diabetes Obes Metab. 2015;17(6):541-5.

371. Palau P, Amiguet M, Dominguez E, Sastre C, Mollar A, Seller J, et al. Short-term effects of dapagliflozin on maximal functional capacity in heart failure with reduced ejection fraction (DAPA-VO(2) ): a randomized clinical trial. Eur J Heart Fail. 2022;24(10):1816-26.

372. Peng XV, Marcinak JF, Raanan MG, Cao C. Combining the G-protein-coupled receptor 40 agonist fasiglifam with sitagliptin improves glycaemic control in patients with type 2 diabetes with or without metformin: A randomized, 12-week trial. Diabetes Obes Metab. 2017;19(8):1127-34.

373. Pirags V, Lebovitz H, Fouqueray P. Imeglimin, a novel glimin oral antidiabetic, exhibits a good efficacy and safety profile in type 2 diabetic patients. Diabetes Obes Metab. 2012;14(9):852-8.

374. Raz I, Hanefeld M, Xu L, Caria C, Williams-Herman D, Khatami H, et al. Efficacy and safety of the dipeptidyl peptidase-4 inhibitor sitagliptin as monotherapy in patients with type 2 diabetes mellitus. Diabetologia. 2006;49(11):2564-71.

375. Reilhac C, Dubourg J, Thang C, Grouin JM, Fouqueray P, Watada H. Efficacy and safety of imeglimin add-on to insulin monotherapy in Japanese patients with type 2 diabetes (TIMES 3): A randomized, double-blind, placebo-controlled phase 3 trial with a 36-week open-label extension period. Diabetes Obes Metab. 2022;24(5):838-48.

376. Rosenstock J, Foley JE, Rendell M, Landin-Olsson M, Holst JJ, Deacon CF, et al. Effects of the dipeptidyl peptidase-IV inhibitor vildagliptin on incretin hormones, islet function, and postprandial glycemia in subjects with impaired glucose tolerance. Diabetes Care. 2008;31(1):30-5.

377. Rosenstock J, Sankoh S, List JF. Glucose-lowering activity of the dipeptidyl peptidase-4 inhibitor saxagliptin in drug-naive patients with type 2 diabetes. Diabetes Obes Metab. 2008;10(5):376-86.

378. Saito D, Kanazawa A, Shigihara N, Sato F, Uchida T, Sato J, et al. Efficacy and Safety of Vildagliptin as an Add-On Therapy in Inadequately Controlled Type 2 Diabetes Patients Treated With Basal Insulin. J Clin Med Res. 2017;9(3):193-9.

379. Sanyal AJ, Kaplan LM, Frias JP, Brouwers B, Wu Q, Thomas MK, et al. Triple hormone receptor agonist retatrutide for metabolic dysfunction-associated steatotic liver disease: a randomized phase 2a trial. Nat Med. 2024;30(7):2037-48.

380. Scherbaum WA, Schweizer A, Mari A, Nilsson PM, Lalanne G, Jauffret S, et al. Efficacy and tolerability of vildagliptin in drug-naive patients with type 2 diabetes and mild hyperglycaemia*. Diabetes Obes Metab. 2008;10(8):675-82.

381. Strain WD, Lukashevich V, Kothny W, Hoellinger MJ, Paldanius PM. Individualised treatment targets for elderly patients with type 2 diabetes using vildagliptin add-on or lone therapy (INTERVAL): a 24 week, randomised, double-blind, placebo-controlled study. Lancet. 2013;382(9890):409-16.

382. Takahashi A, Nomoto H, Onishi K, Manda S, Miya A, Kameda H, et al. A comparative study of the effects of imeglimin add-on or metformin dose escalation on glycaemic variability in subjects with type 2 diabetes treated with low-dose metformin (MEGMI-CGM study). Diabetes Obes Metab. 2024;26(8):3471-4.

383. Takahashi A, Nomoto H, Yokoyama H, Yokozeki K, Furusawa S, Oe Y, et al. Efficacy of imeglimin treatment versus metformin dose escalation on glycemic control in subjects with type 2 diabetes treated with a dipeptidyl peptidase-4 inhibitor plus low-dose metformin: A multicenter, prospective, randomized, open-label, parallel-group comparison study (MEGMI study). Diabetes Obes Metab. 2025;27(3):1466-76.

384. Theurey P, Thang C, Pirags V, Mari A, Pacini G, Bolze S, et al. Phase 2 trial with imeglimin in patients with Type 2 diabetes indicates effects on insulin secretion and sensitivity. Endocrinol Diabetes Metab. 2022;5(6):e371.

385. Usui R, Hamamoto Y, Imura M, Omori Y, Yamazaki Y, Kuwata H, et al. Differential effects of imeglimin and metformin on insulin and incretin secretion-An exploratory randomized controlled trial. Diabetes Obes Metab. 2025;27(2):856-65.

386. Yang W, Xing X, Lv X, Li Y, Ma J, Yuan G, et al. Vildagliptin added to sulfonylurea improves glycemic control without hypoglycemia and weight gain in Chinese patients with type 2 diabetes mellitus. J Diabetes. 2015;7(2):174-81.

387. Yang W, Zhu D, Gan S, Dong X, Su J, Li W, et al. Dorzagliatin add-on therapy to metformin in patients with type 2 diabetes: a randomized, double-blind, placebo-controlled phase 3 trial. Nat Med. 2022;28(5):974-81.

388. Younis A, Eskenazi D, Goldkorn R, Leor J, Naftali-Shani N, Fisman EZ, et al. The addition of vildagliptin to metformin prevents the elevation of interleukin 1ss in patients with type 2 diabetes and coronary artery disease: a prospective, randomized, open-label study. Cardiovasc Diabetol. 2017;16(1):69.

389. Zhu D, Li X, Ma J, Zeng J, Gan S, Dong X, et al. Dorzagliatin in drug-naive patients with type 2 diabetes: a randomized, double-blind, placebo-controlled phase 3 trial. Nat Med. 2022;28(5):965-73.

390. Zhu XX, Zhu DL, Li XY, Li YL, Jin XW, Hu TX, et al. Dorzagliatin (HMS5552), a novel dual-acting glucokinase activator, improves glycaemic control and pancreatic beta-cell function in patients with type 2 diabetes: A 28-day treatment study using biomarker-guided patient selection. Diabetes Obes Metab. 2018;20(9):2113-20.

391. Simela C, Walker JM, Ghosh AK, Chen DH. SGLT2 inhibitors for prevention and management of cancer treatment-related cardiovascular toxicity: a review of potential mechanisms and clinical insights. Cardiooncology. 2025;11(1):15.

392. Sorum ME, Gang AO, Tholstrup DM, Gudbrandsdottir S, Kissow H, Kornblit B, et al. Semaglutide treatment for PRevention Of Toxicity in high-dosE Chemotherapy with autologous haematopoietic stem-cell Transplantation (PROTECT): study protocol for a randomised, double-blind, placebo-controlled, investigator-initiated study. BMJ Open. 2024;14(10):e089862.
